# Alignment-free method for DNA sequence clustering using Fuzzy integral similarity

**DOI:** 10.1038/s41598-019-40452-6

**Published:** 2019-03-06

**Authors:** Ajay Kumar Saw, Garima Raj, Manashi Das, Narayan Chandra Talukdar, Binod Chandra Tripathy, Soumyadeep Nandi

**Affiliations:** 1 Institute of Advanced Study in Science and Technology, Mathematical Sciences Division, Guwahati, 781035 India; 2 Institute of Advanced Study in Science and Technology, Life Science Division, Guwahati, 781035 India; 3 Tripura University, Department of Mathematics, Agartala, 799022 India

## Abstract

A larger amount of sequence data in private and public databases produced by next-generation sequencing put new challenges due to limitation associated with the alignment-based method for sequence comparison. So, there is a high need for faster sequence analysis algorithms. In this study, we developed an alignment-free algorithm for faster sequence analysis. The novelty of our approach is the inclusion of fuzzy integral with Markov chain for sequence analysis in the alignment-free model. The method estimate the parameters of a Markov chain by considering the frequencies of occurrence of all possible nucleotide pairs from each DNA sequence. These estimated Markov chain parameters were used to calculate similarity among all pairwise combinations of DNA sequences based on a fuzzy integral algorithm. This matrix is used as an input for the neighbor program in the PHYLIP package for phylogenetic tree construction. Our method was tested on eight benchmark datasets and on in-house generated datasets (18 s rDNA sequences from 11 arbuscular mycorrhizal fungi (AMF) and 16 s rDNA sequences of 40 bacterial isolates from plant interior). The results indicate that the fuzzy integral algorithm is an efficient and feasible alignment-free method for sequence analysis on the genomic scale.

## Introduction

Phylogenetic tree analysis and comparative studies of taxa are essential parts of modern molecular biology. Phylogenetic reconstruction and comparative sequence analysis traditionally depend on multiple or pairwise sequence alignments. However, various limitations are encountered when analyzing large datasets using an alignment based approach. Whole genome alignment of higher eukaryotes can exceed computational resources. Moreover, factors such as the combinatorics of genomic rearrangements and duplications make the alignment of entire genomes impossible. Therefore, the alignable homologous segments of the genomes under study have to be identified in the initial steps. Recently, large amounts of sequence data produced by next-generation sequencing techniques have become available in private and public databases, which has created new challenges due to the limitations associated with alignment based approaches. This plethora of sequence information increases the computation and time requirements for genome comparisons in computational biology. Therefore, there is a high need for faster sequence analysis algorithms. For this, various methods have been proposed to overcome the limitations of alignment based approach^1–3^, and is termed as alignment-free methods. The alignment-free methods are not only used in phylogenetic studies^4,5^, but also for metagenomics^6–11^, analysis of regulatory elements^12–14^, protein classification^15,16^, sequence assembly^17^, isoform quantification from transcriptome data^18^, and to identify biomarkers in diagnostic tests^19^. The alignment-free methods fall into two broad categories: methods based on k-mer or word frequency, and methods based on match length^20^. Methods based on k-mer or word frequency are quite popular and studied extensively. The k-mer based methods were developed to compare DNA sequences, in which it counts the frequencies of substrings with k letters occurring in respective sequences^21^. In recent past, a lot of k-mer based methods have been proposed and implemented in sequence analysis and phylogeny, such as, feature frequency profile (FFP)^22^, return time distribution (RTD)^23^, frequency chaos game representation (FCGR)^24^, an improved complete composition vector method (ICCV)^25^, composition vector (CV)^26^ and complete composition vector (CCV)^27^. For sequence comparison, ICCV method is more efficient and robust compared to CV and CCV methods. The other category of the alignment-free method is based on match lengths, where it employs the similarity of substrings between two sequences^28–31^. Examples of match length methods are, k-mismatch average common substring^32^, average common substring^28^, *K*_*r*_ – method^28^, etc. These methods are commonly used for string processing in computer science. In this study, we propose to use fuzzy integral^33^ to analyze DNA sequences based on a Markov chain^34^, which can be categorised as k-mer or word frequency method. The fuzzy integral similarity method^35,36^ assigns a similarity score between two DNA sequences based on the estimated parameters of a Markov chain. A DNA sequence consists of four characters (A, T, G and C). By taking the state space as **S** = {A, T, G, C}, we used the *k*-th step transition probability matrix, a fuzzy measure^37^ and fuzzy integral to describe the DNA sequences. We used the fuzzy integral similarity to obtain a distance matrix, which was used in the neighbor program in the PHYLIP package^38^ to construct a phylogenetic tree. The similar fuzzy integral similarity approach was taken by^36^. However in^36^, the method of feature vector extraction from the DNA sequences is different from our method. In both our method and^36^, the extracted features are used as an input for the fuzzy integral similarity analysis. The proposed method is tested on 18S rDNA sequences from 11 Arbuscular mycorrhizal fungi isolates and 16S rDNA sequences from 40 bacterial isolates, and also tested on the following benchmark datasets, 41 mammalian mitochondrial genomes, 59 ebolavirus complete genomes, 30 coronavirus whole genomes, 30 bacterial whole genomes, 48 Hepatitis E virus (HEV) whole genomes, 24 Eutherian mammals sequences, 58 genome datasets from different species and 29 Escherichia/Shigella complete genomes. The method was also tested on large mammalian dataset. In addition, we used receiver operating characteristic (ROC)^39–41^ curve for measuring the performance of our method to compare the other alignment-free methods from Alfree repository^2^. The consistency can also be seen from the statistical analysis such as AUC (area under the ROC) values, calculated from ROC curves provided in Supplementary Material.

## Materials and Methods

### Construction of a Markov chain for DNA sequence

Let *P* = [*p*_*ij*_] denote the transition probability matrix of a discrete-time Markov chain^34^. Each state transition probability *p*_*ij*_ is defined as follows:

1 $${p}_{ij}=p({X}_{n+1}={s}_{j}|{X}_{n}={s}_{i}),\,1\le i,j\le S,$$

where *X*_*n*_ indicates the actual state at time *n*(*n* = 1, 2, 3 …) and *s*_*i*_ is the *i*_*th*_ state of *S* distinct states. In the context of a DNA sequence, the number of states is *S* = 4 which corresponds to the four nucleotide symbol set ${\bf{S}}=\{A={s}_{1},T={s}_{2},G={s}_{3},C={s}_{4}\mathrm{\}}.$ The state transition probabilities are subject to

$${p}_{ij}\ge 0\,\forall i,j\,\,{\rm{and}}\,\,\sum _{j=1}^{S}\,{p}_{ij}=1\,\forall i.$$

Since the transition probabilities are unknown initially, they must be estimated based on the observed sequence. Here, we estimate the parameters of the Markov chain by taking the frequencies of occurrence of all possible nucleotide pairs for each sequence^42^. If the total number of each adjacent nucleotide pair (*s*_*i*_, *s*_*j*_) in the sequence is denoted by ${N}_{{s}_{i}{s}_{j}}$, then the 1^*st*^-step transition probability from state *s*_*i*_ to state *s*_*j*_ is estimated as

2 $${p}_{ij}=\frac{{N}_{{s}_{i}{s}_{j}}}{{N}_{{s}_{i}A}+{N}_{{s}_{i}T}+{N}_{{s}_{i}G}+{N}_{{s}_{i}C}},$$

where ${N}_{{s}_{i}{s}_{j}}$ represents the total number of each adjacent pair starting from nucleotide *s*_*i*_ and ending with nucleotide *s*_*j*_.

Presented above is the 1-step Markov chain. The k-step Markov chain can be calculated through the 1-step Markov chain, which is known as the Chapman-Kolmogorov process. Let ${P}^{k}=[{p}_{ij}^{k}]$ denote the transition probability matrix of a discrete-time Markov chain in state *j* after *k* steps from state *i*. Each state transition probability ${p}_{ij}^{k}$ is defined as follows:

3 $${p}_{ij}^{k}={p}^{k}({X}_{n+k}={s}_{j}|{X}_{n}={s}_{i}),\,1\le i,j\le S,$$

The state transition probabilities are subject to

$${p}_{ij}^{k}\ge 0\,\forall i,j\,\,{\rm{and}}\,\,\sum _{j=1}^{S}\,{p}_{ij}^{k}=1\,\forall i.$$

For any three events, *A*, *B* and *C*, the following identity is known: $p[A\cap B|C]=p[A|B\cap C]p[B|C].$ By interpreting *A* as *X*_*n*+*k*_ = *s*_*j*_, *B* as *X*_*n*+*t*_ = *s*_*r*_ and *C* as *X*_*n*_ = *s*_*i*_, we have

4 $$\begin{array}{rcl}{p}_{ij}^{k} & = & p[{X}_{n+k}={s}_{j}|{X}_{n}={s}_{i}]\\  & = & \sum _{{s}_{r}\in {\bf{S}}}\,p[{X}_{n+k}={s}_{j},{X}_{n+t}={s}_{r}|{X}_{n}={s}_{i}]\\  & = & \sum _{{s}_{r}\in {\bf{S}}}\,p[{X}_{n+k}={s}_{j}|{X}_{n+t}={s}_{r},{X}_{n}={s}_{i}]\times p[{X}_{n+t}={s}_{r}|{X}_{n}={s}_{i}]\\  & = & \sum _{{s}_{r}\in {\bf{S}}}\,p[{X}_{n+k}={s}_{j}|{X}_{n+t}={s}_{r}]\times p[{X}_{n+t}={s}_{r}|{X}_{n}={s}_{i}]\\  & = & \sum _{{s}_{r}\in {\bf{S}}}\,{p}_{rj}^{k-t}{p}_{ir}^{t},\end{array}$$

which is known as the Chapman-Kolmogorov equation.

Hence, the matrix with the elements ${p}_{ij}^{k}$ is $[{p}_{ij}^{k}]={P}^{k}$.

The selection of step *k* plays an important role in capturing rich evolutionary information from the DNA sequence. In the context of a DNA sequence, the *k*^*th*^-step transition probability can be written as:

5 $${P}^{k}=[\begin{array}{cccc}{p}_{11}^{k} & {p}_{12}^{k} & {p}_{13}^{k} & {p}_{14}^{k}\\ {p}_{21}^{k} & {p}_{22}^{k} & {p}_{23}^{k} & {p}_{24}^{k}\\ {p}_{31}^{k} & {p}_{32}^{k} & {p}_{33}^{k} & {p}_{34}^{k}\\ {p}_{41}^{k} & {p}_{42}^{k} & {p}_{43}^{k} & {p}_{44}^{k}\end{array}]=[\begin{array}{cccc}{p}_{AA}^{k} & {p}_{AT}^{k} & {p}_{AG}^{k} & {p}_{AC}^{k}\\ {p}_{TA}^{k} & {p}_{TT}^{k} & {p}_{TG}^{k} & {p}_{TC}^{k}\\ {p}_{GA}^{k} & {p}_{GT}^{k} & {p}_{GG}^{k} & {p}_{GC}^{k}\\ {p}_{CA}^{k} & {p}_{CT}^{k} & {p}_{CG}^{k} & {p}_{CC}^{k}\end{array}]$$

Which is subject to ${p}_{ij}^{k}\ge 0\,\forall i,\,j\in \{1,2,3,4\}$ and ${\sum }_{j=1}^{4}\,{p}_{ij}^{k}=1\,\forall i.$ The ${p}_{ij}^{k}$ can be calculated using the above Eqs (2 and 4).

### Fuzzy measure and fuzzy integral for the *k*^*th*^-step nucleotide sequence

Fuzzy set theory^43^ is particularly suitable for modelling imprecise data, whereas fuzzy integral is highly appropriate for representing the interaction among different information sources. The concept of fuzzy integral with respect to a fuzzy measure has been proposed by Sugeno in 1974^44^. In this section, we propose the use of the fuzzy integral incorporating with the transition probability matrix, where the elements of transition probability matrix are taken as fuzzy membership degree.

Let $F=\{{({s}_{i}{s}_{j})}^{k}={y}_{ij}|i,j\in \{1,2,3,4\}\}$ be the finite set of *k*^*th*^-step nucleotides starting from nucleotide *s*_*i*_ and ending with nucleotide *s*_*j*_ estimated from the observed sequence.

Let *X*, *Y* ⊆ *F* and *R*(*F*) be the power set of *F*. A fuzzy measure *μ* is a real valued function:

*μ*: *R*(*F*) → [0, 1] satisfies the given condition,

- *μ*(*ϕ*) = 0 and *μ*(*F*) = 1.
- *μ*(*X*) ≤ *μ*(*Y*) if *X* ⊆ *Y*.

For a fuzzy measure *μ* let $\mu ({y}_{ij})={\mu }^{ij}\,\forall {y}_{ij}\in F.$ The mapping ${y}_{ij}\to {\mu }^{ij}$ is termed a fuzzy density function. The fuzzy density function can be interpreted as the importance of element *y*_*ij*_ in determining the set *F*. By definition of the fuzzy measure *μ*, the measure of the union of two disjointed subsets cannot be directly computed from their disjointed component measures. In other words, the fuzzy measure value of a given subset is not simply the summation of the measures of its elements. Therefore, to define a fuzzy measure, we need to know the fuzzy densities of each element of the measured set and the measure of each combination. This measure can be provided by an expert or extracted from the problem definition. However, when dealing with a set of numerous elements, this job may become noisy, tedious or even unfeasible. A possible solution to this problem is to use a *λ* – fuzzy measure. A *λ* – fuzzy measure^45^ fulfills the criteria of a fuzzy measure, and has an additional property: for all $X\cap Y=\varphi ,\,X,Y\subseteq \{{y}_{i1},{y}_{i2},{y}_{i3},{y}_{i4}\}$ for fixed $i\in \{1,2,3,4\}$ and

6 $$\mu (X\cup Y)=\mu (X)+\mu (Y)+{\lambda }_{i}\mu (X)\mu (Y),\,{\rm{for}}\,{\rm{each}}\,{\lambda }_{i} > -\,1.$$

Furthermore, *λ*_*i*_ can be calculated by solving:

7 $${\lambda }_{i}+1=\prod _{j=1}^{4}\,\mathrm{(1}+{\lambda }_{i}{\mu }^{ij})\,{\rm{for}}\,{\rm{fixed}}\,\,i.$$

For solving Eqs (6 and 7), we only need to assemble information regarding the individual fuzzy densities of the elements ${\mu }^{ij}\,(i,j=1,2,3,4).$

Let $F=\{{({s}_{i}{s}_{j})}^{k}={y}_{ij}|i,\,j\in \{1,2,3,4\}\}$ be a finite set of information sources. Let *h*: *F* → [0, 1] represent a function that maps each element of *F* to its observed evidence. Suppose that $h({y}_{i1})\ge h({y}_{i2})\ge h({y}_{i3})\ge h({y}_{i4})$ for each fixed $i\in \{1,2,3,4\}$ If the decreasing order criterion is not fulfilled, then *F* should be reordered so that the decreasing order relationship holds, and further investigation will be based on the modified relationship. Let *μ*: *R*(*F*) → [0, 1] be a fuzzy measure. Then, the fuzzy integral of *h* with respect to the fuzzy measure *μ* is

8 $$I=max[ma{x}_{i=1}^{4}[mi{n}_{j=1}^{4}[h({y}_{ij}),\mu ({A}_{ij})]]],$$

9 $${\rm{where}}\,{A}_{ij}=\{{y}_{i1},{y}_{i2},\ldots ,{y}_{ij}\}\,{\rm{for}}\,{\rm{each}}\,{\rm{fixed}}\,i.$$

The fuzzy integral considers the significance provided by every element of a given set, and the importance of each subset of elements (i.e., the fuzzy measure) plays an important role in its decision-making process. The combination of the extracted information and the importance of the provided source convert the fuzzy integral to an appropriate form for information fusion. This theory has the potential to address uncertainties associated with issues related to data extraction and their processing procedures. Therefore, the theory has been widely implemented in feature extraction and classification^45,46^.

### Fuzzy integral similarity and distance matrix for sequence comparison

The fuzzy integral similarity is based on the distance of the *k*^*th*^-step nucleotide pair frequency with respect to the conservation level of the position between two sequences. In our case, the *k*^*th*^-step nucleotide pair frequency at all sixteen positions in the transition probability matrix is taken as the fuzzy membership degree.

Let ${P}_{1}^{k}$ and ${P}_{2}^{k}$ be two *k*^*th*^-step transition probability matrices. The fuzzy integral function find the similarity level of the nucleotide pairs between *k*^*th*^-step transition probability matrices. We constructed a fuzzy integral function *h*, which is given as:

10 $${h}^{{y}_{ij}}=1-|{({P}_{1}^{k})}^{{y}_{ij}}-{({P}_{2}^{k})}^{{y}_{ij}}|,$$

where *y*_*ij*_ ∈ {(*AA*)^*k*^, (*AT*)^*k*^, (*AG*)^*k*^, (*AC*)^*k*^, (*TA*)^*k*^, (*TT*)^*k*^, (*TG*)^*k*^, (*TC*)^*k*^, (*GA*)^*k*^, (*GT*)^*k*^, (*GG*)^*k*^, (*GC*)^*k*^, (*CA*)^*k*^, (*CT*)^*k*^, (*CG*)^*k*^, (*CC*)^*k*^}.

Additionally, the fuzzy measure function find the maximum level of conservation of the nucleotide pairs between *k*^*th*^-step transition probability matrices ${P}_{1}^{k}$ and ${P}_{2}^{k}$, which favours the importance of better conserved positions.

Taking advantage of the properties explained above, we can construct a *λ* – fuzzy measure *μ* using the fuzzy density of each element *μ*^*ij*^.

In this case,

11 $${\mu }^{ij}={\mu }^{{y}_{ij}}=max\{{({P}_{1}^{k})}^{{y}_{ij}},{({P}_{2}^{k})}^{{y}_{ij}}\},$$

where *y*_*ij*_ ∈ {(*AA*)^*k*^, (*AT*)^*k*^, (*AG*)^*k*^, (*AC*)^*k*^, (*TA*)^*k*^, (*TT*)^*k*^, (*TG*)^*k*^, (*TC*)^*k*^, (*GA*)^*k*^, (*GT*)^*k*^, (*GG*)^*k*^, (*GC*)^*k*^, (*CA*)^*k*^, (*CT*)^*k*^, (*CG*)^*k*^, (*CC*)^*k*^}. At this stage, we should apply Eq. (7) to find *λ* and apply the value of *λ* in Eq. (6) to finally obtain the fuzzy measure *μ*. The result generated by Eq. (6) satisfies the given criteria (*a*) and (*b*) of the fuzzy measure. After generating *h* and *μ*, we obtained the fuzzy integral similarity by applying Eq. (8). In fuzzy integral similarity, greater importance is given to the higher degree of membership which is calculated via the fuzzy integral with respect to the fuzzy measure. It is based on fuzzy technology and is intended to deal with the intrinsic uncertainty involved in sequence comparison tasks. Fuzzy integral similarity does not require any additional parameters, which makes it fully automated and robust.

The fuzzy integral similarity measure provides the similarity score between the two *k*^*th*^-step transition probability matrices. Next, we will define a distance measure between two *k*^*th*^-step transition probability matrices ${P}_{1}^{k}$ and ${P}_{2}^{k},$ which is given as follows:

12 $$D({P}_{1}^{k},{P}_{1}^{k})=1-I({P}_{1}^{k},{P}_{1}^{k}).$$

Similarly using Eq. (12), we can calculate the distance measure for all pairwise combinations taken from an *n* number of DNA sequences. Finally, a symmetric distance matrix is generated. This matrix is used as an input for the neighbor program in the PHYLIP package^38^ for phylogenetic tree construction.

### Algorithm

This section describes the algorithmic aspect of the proposed method. The entire algorithm contains three stages.

**Stage 1**: Calculation of the transition probability matrix through Markov chain:

**Algorithm 1:**
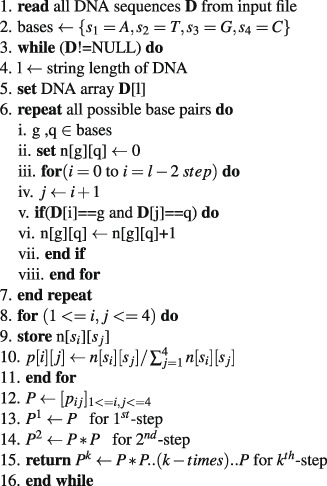
*k*^*th*^-step transition probability matrix.

**Stage 2**: Calculation of fuzzy integral similarity between two ${k}^{th}-$ step transition probability matrices:

**Algorithm 2:**
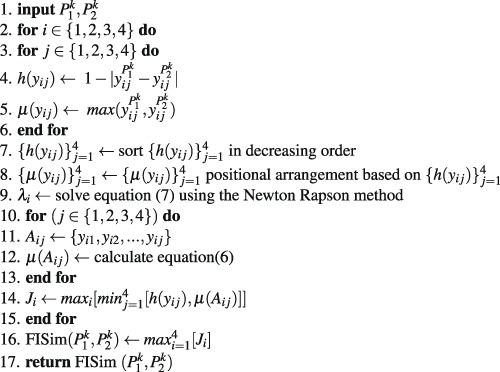
FISim $$({P}_{1}^{k},{P}_{2}^{k})$$.

**Stage 3**: We integrate stage(1) and stage(2) for phylogenetic construction:

**Algorithm 3:**
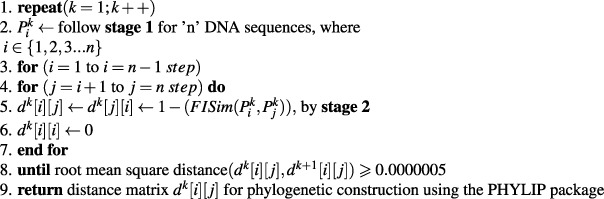
Construction of distance matrix.

### Time complexity of the proposed algorithm

To determine the time complexity of a given algorithm, we assume that all operations took the same unit of time. The whole computational process consists of three stages. In the first stage, we calculate the transition probability matrix from the raw DNA sequences. The time complexity of stage (1) is *O*(*m*^3^*nk* + *nl*), where *l* is the average length of the DNA sequences, *n* is the total number of DNA sequences, *m* is the number of bases and *k* is the *k*^*th*^-step transition probability matrix. In the second stage, we calculate the fuzzy integral similarity between the two *m* × *m* transition probability matrices generated in stage (1). The time complexity of stage (2) is *O*(*m*2^*m*^). In the third stage, we integrate stage (1) and stage (2) to generate a distance matrix. Let *k* = *h* be an optimal step that satisfies condition (8) in algorithm (3). Therefore, the total time complexity to generate the final distance matrix at the *h*^*th*^ optimal step is:

$$\begin{array}{c}=\,{h}^{th}-step\,{\rm{time}}\,{\rm{complexity}}\,{\rm{of}}\,{\rm{stage}}\,1+h((n(n-1))\mathrm{/2})\ast {\rm{time}}\,{\rm{complexity}}\,{\rm{of}}\,{\rm{stage}}\,2\\ =\,O({m}^{3}nh+nl)+h((n(n-\mathrm{1))/2)}\ast O(m{2}^{m})\\ =\,O({m}^{3}nh+nl)+O(h{n}^{2}m{2}^{m})\\ =\,O({m}^{3}nh+nl+h{n}^{2}m{2}^{m}).\end{array}$$

Since we are calculating the computational complexity for DNA sequences, the number of bases (A, T, G and C) is *m* = 4. Hence, the time complexity of our proposed algorithm are *O*(*nh* + *nl* + *hn*^2^).

## Results

To check the performance of the proposed method, it was tested on different datasets. Some datasets are small sized and others are medium sized. The length of sequences ranges from seven thousands to several millions base pairs. In order to compare and analyze various genomic data, we generated a distance matrix using Eq. (12) for each distinct step *k* using the method described above. We increased step *k* until we obtained the same distance matrix for two consecutive distinct *k*(suppose *k* = *h and h* + 1, where *h* is a fixed integer), (i.e., the root mean square distance^47^ between two distance matrices generated by step *h* and *h* + 1 should be zero). Therefore, we considered *k* = *h* an optimal step and generate the phylogenetic tree at step *k* = *h* using the PHYLIP package. Here, we use the UPGMA (Unweighted Pair Group Method with Arithmetic Mean) approach in the PHYLIP package^38^ to generate the phylogenetic tree. To test the effectiveness of our proposed approach, we selected ten sets of test data: (i) 18S rDNA sequences from 11 Arbuscular mycorrhizal fungi isolates, (ii) 16S rDNA sequences from 40 bacterial isolates, (iii) 41 mammalian mitochondrial genomes, (iv) 59 ebolavirus complete genomes, (v) 30 coronavirus whole genomes, (vi) 30 bacterial whole genomes, (vii) 48 Hepatitis E virus (HEV) whole genomes, (viii) 24 Eutherian mammals sequences, (ix) 58 genome datasets from different species and (x) 29 Escherichia/Shigella complete genomes. We compared our tree with the tree generated by the previously published method using same datasets.

### Phylogenetic tree analysis using 18S rDNA sequences from 11 arbuscular mycorrhizal fungi (AMF) isolates

**Figure 1:**
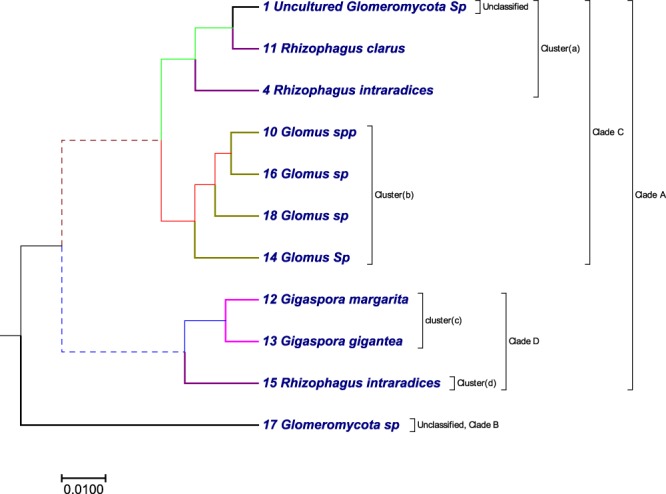
The phylogenetic tree of the 11 AMF sequences constructed using our method.

Arbuscular mycorrhizal fungi (which is also called an AM fungi (AMF) or endomycorrhiza) is a type of mycorrhiza in which the fungus infects vascular plants by penetrating the cortical cells of the root. Arbuscular mycorrhizas are characterized by the formation of unique structures (arbuscules) and vesicles, these fungi belong to phylum *glomeromycota*. Arbuscular mycorrhizas fungi help plants to capture nutrients, such as phosphorus, sulfur, nitrogen and micronutrients, from soil. The development of arbuscular mycorrhizal symbiosis is believed to have played a crucial role in the initial colonization of plants on land and in the evolution of vascular plants^48^. We built a phylogenetic tree (Fig. 1) using the optimal step *k* = 8 of 11 AMF sequences listed in Table S1. To compare our method with an alignment-based method, we also constructed the phylogenetic tree (Fig. S1) by ClustalW method using MEGA package^49^. We characterized 11 AMF sequences based on their families and genera. All *rhizophagus* genera belonging to family *glomeraceae* were clustered together in cluster (a), except one genus of *rhizophagus* (i.e., the “15 Rhi in” sequence belongs to cluster (d)). All *glomus* genera belonging to family *glomeraceae* were clustered in cluster (b). All *gigaspora* genera belonging to family *gigasporaceae* were clustered in cluster (c). While comparing the tree prepared by our method (Fig. 1) with the tree prepared by ClustalW method (Fig. S1) using the UPGMA approach, we found that, *glomus* genera were clustered together in Fig. 1 which was lacking in Fig. S1. An obvious flaw in both the phylogenetic trees (Figs 1 and S1) is, none of them clustered *rhizophagus* genera in the single clade.

### Phylogenetic tree analysis using 16S rDNA sequences from 40 bacterial isolates

**Figure 2:**
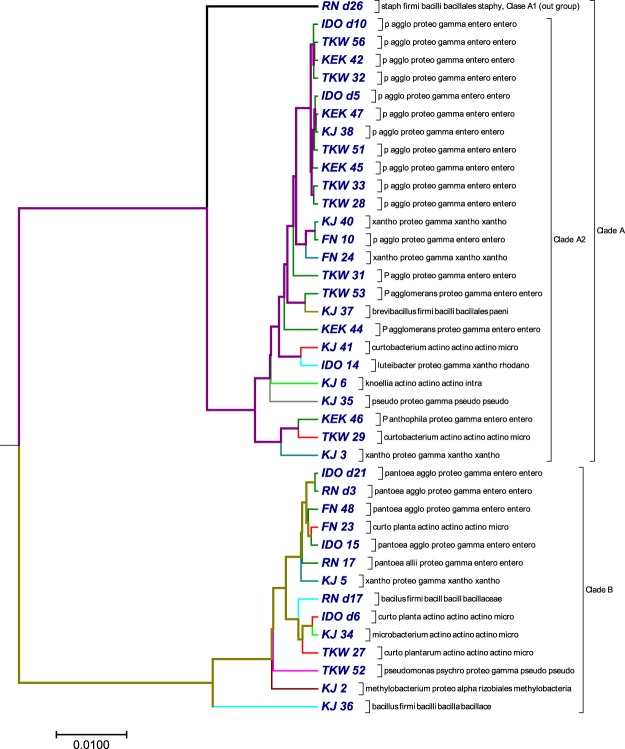
The phylogenetic tree constructed by our method using 16S rDNA sequences from 40 bacterial isolates.

Endophytic bacteria are an essential part of plant systems and play significant roles in plant growth and development^50^. The 40 bacterial sequences were obtained from pure cultures of endophytic bacteria isolated from the surface sterilized mature endosperms of six rice varieties. The rice seeds were collected from two different locations in north-east India: North-Lakhimpur, Assam, Aizawl and Mizoram. Genomic DNA was extracted from the pure cultures, and the full length 16S rDNA sequences were amplified using the primer pair 27f (5′-AGAGTTTGATYMTGGCTCAG) and 1492r (5′-TACCTTGTTAYGACTT). The amplicons were sequenced in an Applied Biosystems sequencer using the BigDye terminator method. To minimize sequencing error, we also used two internal primers 533f/805r along with 27 f/1492r. We assembled the contigs based on their phred scores (15) using the Codon Code aligner v7.0.1, BioEdit and SeqTrace v0.9 software^51^. The contigs were checked for the presence of any chimeras in mothur v1.35.1 and were aligned for identification against the NCBI reference rRNA database using the blastn algorithm. Information, including the accession numbers, phyla, classes, orders and families for the 40 bacterial isolates are listed in Table S2. With there 40 bacterial isolates, we generated a phylogenetic tree (Fig. 2) with our approach using the optimal step *k* = 6. The tree (Fig. 2) obtained by our method was compared with the tree (Fig. S2) obtained by ClustalW method using MEGA package^49^. Our algorithm separated the 40 bacterial sequences into two major clades: clade A (purple) and clade B (green) (Fig. 2). Clade A branched into two clades: clade A1 (bold black) and clade A2 (purple). Clade A1 contained only *Staphylococcus warneri*, which separated out as an outgroup from the sequences in clade A2. Clade A2 consisted of 25 sequences, of which one sequence represented phylum *firmicutes*, three sequences represented *actinobacteria*, and the remaining 21 sequences belonged to phylum *proteobacteria*. Our method successfully grouped sequences of genus pantoea together, but in one instance it placed pantoea and xanthomonas as sister groups. In the same clade, our method placed a third sequence belonging to xanthomonas as an outlier. Additionally, in clade A2, brevibacillus and pantoea were clustered as a sister group, which belong to phylum *firmicutes* and *proteobacteria* respectively and curtobacterium was grouped with luteibacter. None of the actinobacterial sequences were grouped together in this clade.

The second major cluster or clade B(green) consisted of 14 sequences. *Bacillus marisflavi* was an outlier from the remaining 13 sequences. In this cluster, *Curtobacterium plantarum* and *Pantoea agglomerans* were placed together as sister groups, which might indicate sequence similarity between the two species. Three actinobacterial species (two sequences of *C. plantarum* and one sequence of *Microbacterium proteolyticum*) were placed together in one clade. However, *C. plantarum* and *M. proteolyticum* were placed as sister groups, and the other *C. plantarum* sequence was positioned as an outlier. When we compared our method (Fig. 2) with ClustalW method (Fig. S2), we found that both methods clearly separated the 40 bacterial sequences into two major clades. Each clade contains the same type of bacterial sequences, but the order was interchanged. In clade A, our method failed to cluster xanthomonas together, which was grouped together in the result obtained by ClustalW.

### Phylogenetic tree analysis of 41 mammalian mitochondrial genomes

**Figure 3:**
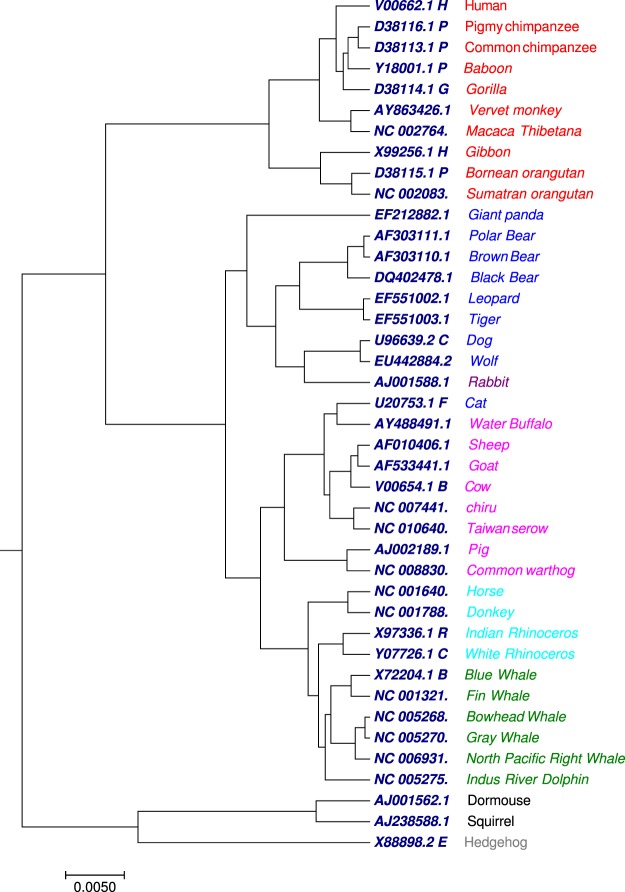
The phylogenetic tree of 41 mammalian mitochondrial genomes constructed using our method.

The proposed algorithm was tested on the benchmark mammalian dataset containing 41 complete mitochondrial genomes(mtDNA) with nearly 16500 nucleotides (Table S3). The tree generated by our approach (Fig. 3) using the optimal step *k* = 6, the 41 species were correctly divided into eight groups: *Primates* (red), *Cetacea* (green), *Artiodactyla* (pink), *Perissodactyla* (light green), *Rodentia* (black), *Lagomorpha* (dark red), *Carnivore* (blue), and *Erinaceomorpha* (grey). The cat species in our approach was clustered with the *Artiodactyla* group. We compared the phylogenetic tree (Fig. 3) generated by our approach with the phylogenetic tree (Figs S3 and S4) collected from previous work^52^. The Fig. S3 is generated by multiple encoding vector method^52^ and Fig. S4 is generated by FFP method^22^ using substrings length seven. In Fig. 3, the 10 primates (red) formed a cluster, also Vervet monkey and Macaca Thibetana of family *cercopithecidae* were clustered in a single clade as sister group which was not observed in Fig. S3. Moreover, species belong to *Artiodactyla* were grouped into a separate clade, which was lacking in Fig. S3. We have also compared our result with the phylogenetic tree (Fig. S4) generated by FFP method. As showed in Fig. S4, the eight groups were not classified well. The four species of *Perissodactyla* were distributed into two clades. Indus RiverDolphin from *Cetacea* was separated from other species of *Cetacea*. The *Primates*, *Artiodactyla* and *Carnivore* clades were all divided into more than one group. The phylogenetic tree (Fig. 3) generated by our approach shows a better clustering as compared to Figs S3 and S4.

### Phylogenetic tree analysis of 59 ebolavirus complete genomes

**Figure 4:**
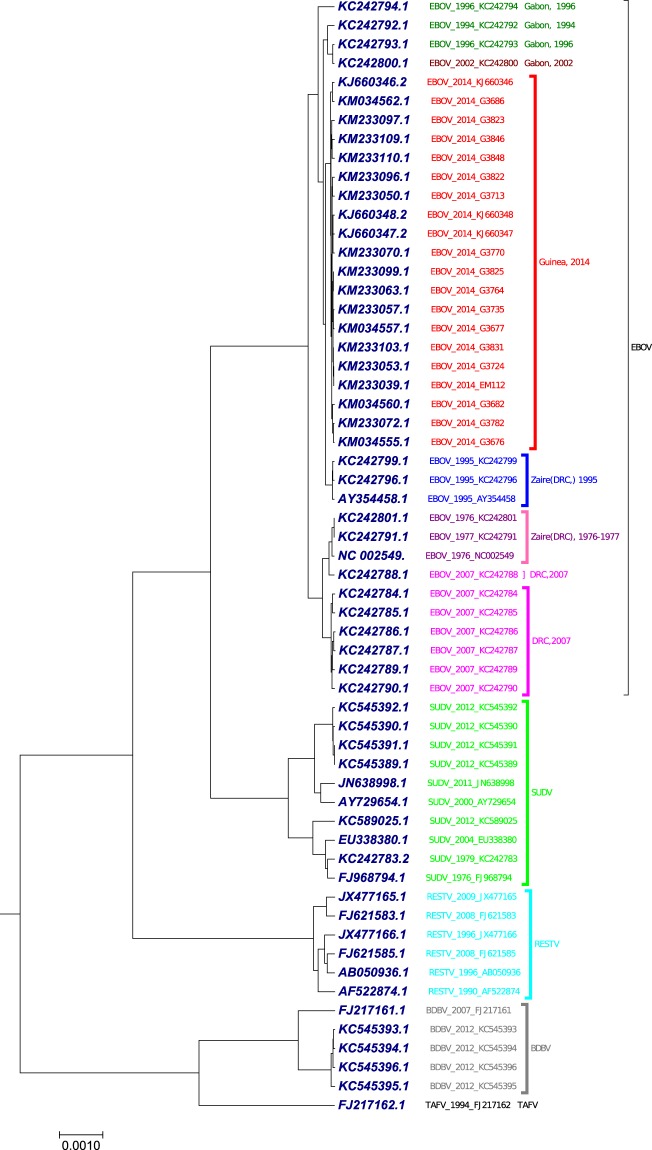
The phylogenetic tree of 59 ebolavirus complete genomes constructed using our method.

The benchmark dataset used in this study was 59 complete genomes of ebolavirus with nearly 18900 nucleotides (Table S4). The *Ebolavirus* genus includes five species: Bundibugyo virus (BDBV), Reston virus (RESTV), Ebola virus (formerly Zaire ebolavirus, EBOV), Sudan virus (SUDV), and Tai Forest virus (TAFV)^53^. Ebola viruses are single-strand negative sense RNA viruses. Each ebolavirus genome encodes seven proteins in which glycoprotein is the only viral protein on the surface of ebolavirus. The first case of human, infected by EBOV, was reported in 1976 in Zaire (currently the Democratic Republic of the Congo (DRC))^54^. We applied our proposed method to generate the phylogenetic tree (Fig. 4) using the optimal step *k* = 6 of 59 viruses in *Ebolavirus* genus. As shown in Fig. 4, the five species were correctly separated. The EBOV strains from the recognized pandemics build a lineage independent of the other four species in genus *Ebolavirus*. The EBOV strains in Zaire (DRC) pandemic in 1976–1977 were clustered together as a clade. The EBOV strains in DRC pandemic in 2007 were clustered together with the exception *EBOV*_2007_*KC*242788, which was clustered with Zaire (DRC) in 1976–1977. The EBOV strains in Guinea epidemic in 2014 were clustered together as a clade. The three EBOV strains from the 1995 outbreak in Zaire (DRC) formed a clade. SUDV and RESTV formed separate clades. BDBV and TAFV viruses were positioned together. Our result was in consensus with the result generated using multiple encoding vector method^52^ (Fig. S5) and FFP method^22^ (Fig. S6).

### Phylogenetic tree analysis of 30 coronavirus whole genomes

**Figure 5:**
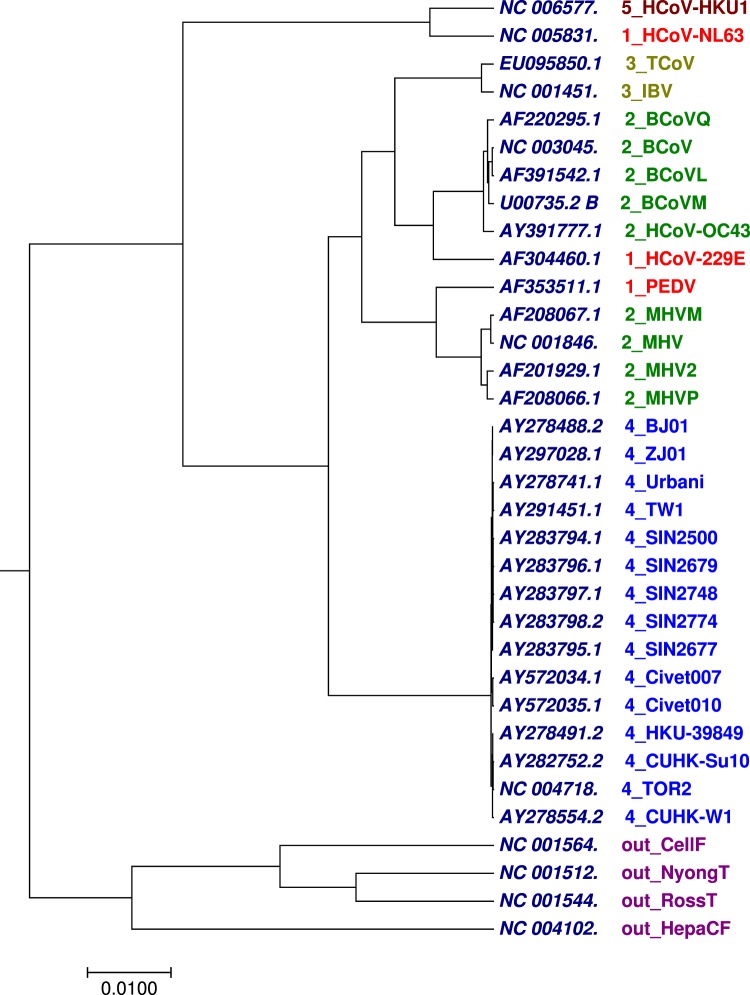
The phylogenetic tree of 30 coronavirus whole genomes constructed using our method.

The other benchmark dataset used for validation of the method was the 30 complete coronavirus genomes with nearly 25,000 to 32,000 nucleotides (Table S5). Coronaviruses^55^ are enveloped, single-stranded, positive-sense RNA viruses within the family *Coronaviridae*^56^. The coronaviruses are pleomorphic RNA viruses that are widespread among avians, bats, humans and other mammals. They are known to cause mild to severe respiratory diseases, gastroenterological, neurological and systemic conditions. This group of virus can easily cross species-barrier and infect new species^57^. As a result of pandemics from coronaviruses especially the SARS, the classification and evolutionary relationships among these viruses have been extensively investigated. We employed our method to analyse the 30 coronavirus whole genome sequences along with 4 non-coronaviruses as outgroups. The 30 coronavirus were classified into five groups according to their host type. As shown in Fig. 5 generated by our approach using the optimal step *k* = 7, we can observe that the 30 coronavirus along with 4 non-coronaviruses were correctly grouped according to their host type except group 1 (Table S5). We compared Fig. 5 generated by our approach with Figs S7, S8, S9 and S10 collected from previous work^52,58^. The limitation observed in our result (Fig. 5) is that, our method was unable to cluster group 1 as compared to Figs S7 and S8. While in Fig. S9 generated by *k*–mer^59^ method and Fig. S10 generated by FFP method using substrings length six, the four non-coronaviruses were not clustered together. Therefore, for this dataset, tree generated by our approach has advantage over *k*–mer and FFP methods using substrings length six.

### Phylogenetic tree analysis of 30 bacterial whole genomes

**Figure 6:**
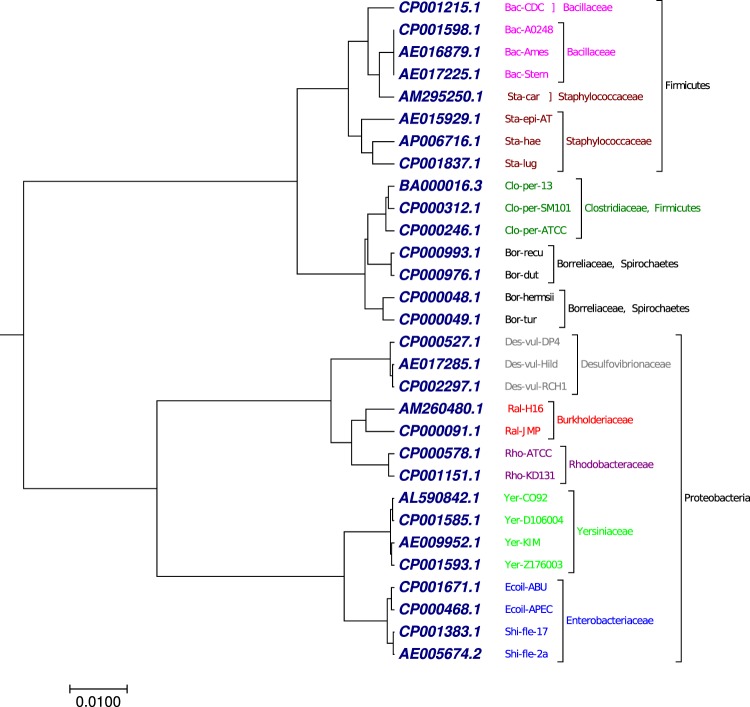
The phylogenetic tree of 30 bacterial whole genomes constructed using our method.

Another benchmark dataset used in this study was 30 complete bacterial genomes with more than 1 million nucleotides (Table S6). Methods based on multiple sequence alignment program cannot handle such large dataset. As shown in Fig. 6, generated by our approach using the optimal step *k* = 7, the 30 bacterial genomes were clustered into nine groups based on taxnomic family: *Burkholderiaceae*, *Rhodobacteriaceae*, *Enterobacteriaceae*, *Borreliaceae*, *Bacilleceae*, *Clostridiaceae*, *Desulfovibrionaceae*, *Yersiniaceae*, and *Staphylococcaceae*. Our result (Fig. 6) has similarity with the result (Fig. S11) generated by fourier power spectrum method at the taxnomic family level collected from previous work^58^. However, our phylogenetic tree (Fig. 6) has advantages at the phylum level which was lacking in Fig. S11. As shown in Fig. 6, the genomes were successfully clustered into three phylum, *Firmicutes*, *Proteobacteria*, and *Spirochaetes* as a separate clade, which was not observed in Fig. S11.

### Phylogenetic tree analysis of 48 Hepatitis E virus (HEV) whole genomes

**Figure 7:**
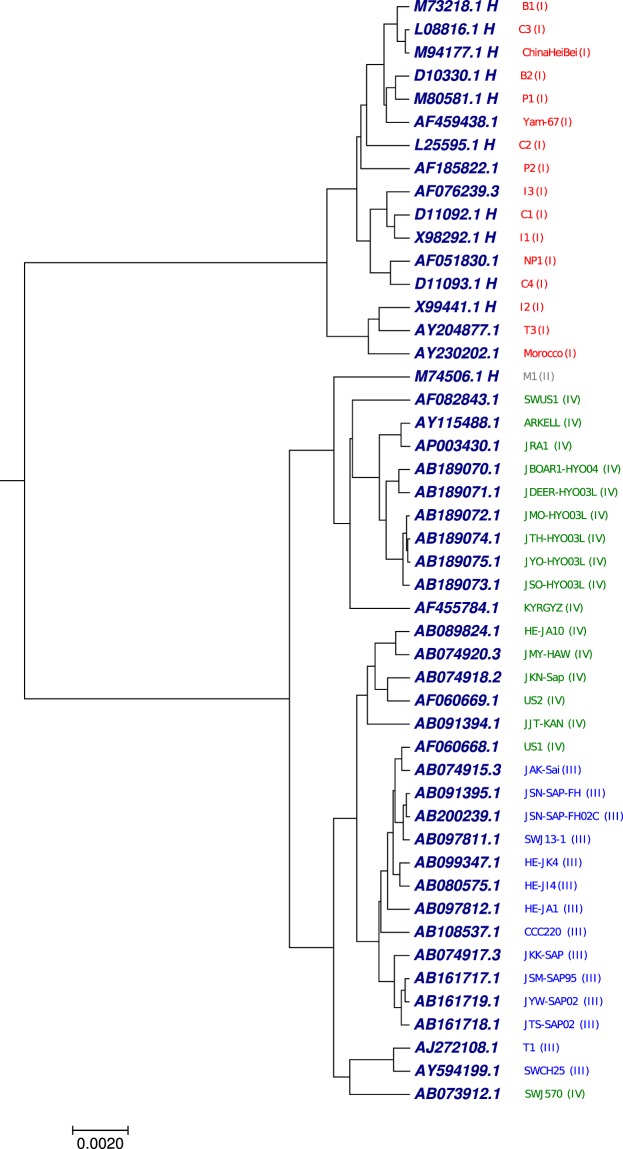
The phylogenetic tree of 48 Hepatitis E virus (HEV) whole genomes constructed using our method.

The other benchmark was 48 complete genomes of hepatitis E virus (HEV). This virus is characterized as non-enveloped, single-stranded RNA virus with nearly 7200 nucleotides (Table S7). The acute condition of the disease is caused by the hepatitis E virus. The difference between other known hepatitis viruses (A, B, C, D) and hepatitis E virus is that, the hepatitis E virus is the only animal-host disease hepatitis^60^. To understand the relationship between HEV sequences, we have applied our proposed method to generate the phylogenetic tree using the optimal step *k* = 6 of the 48 HEV whole genome sequences. As shown in Fig. 7 generated by our approach, the HEV genomes were divided into separate clades based on four genotypic^61^ category (I(red), II(grey), III(blue) and IV(green)) except few sequences. Phylogenetic tree (Fig. 7) generated by our approach shows better clade distribution based on the genotypic division as compared with Figs S12 and S13 collected from the previous work^62^.

### Phylogenetic tree analysis of 24 Eutherian mammal sequences

**Figure 8:**
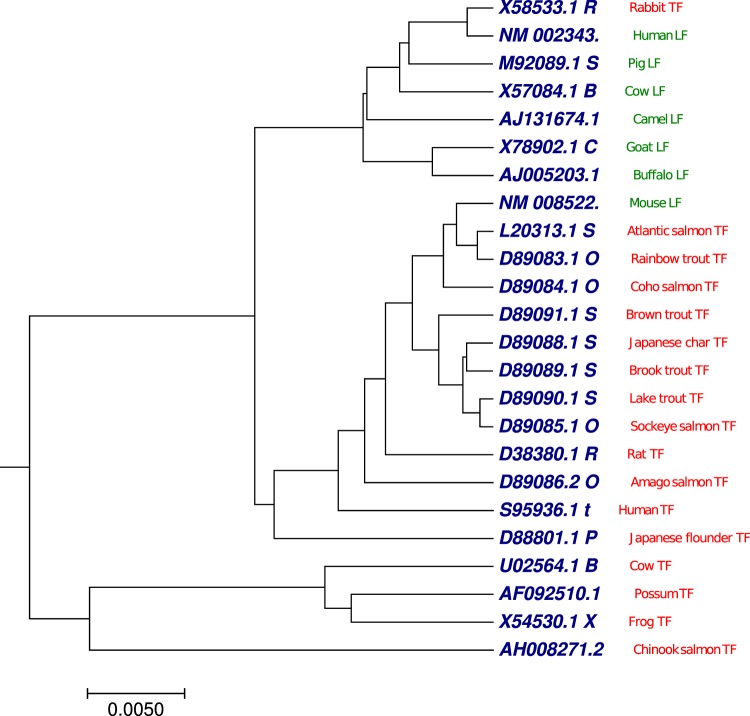
The phylogenetic tree of 24 Eutherian mammals sequences constructed using our method.

We selected transferrin (red) and lactoferrin (green) sequences from 24 vertebrates as a benchmark dataset^63^ (Table S8). Vertebrate transferrins and lactoferrins are iron-binding proteins found in blood serum, milk, egg whites, tears, and interstitial spaces. They can be involved in iron storage and resistance to bacterial disease. We have applied our proposed method to generate the phylogenetic tree (Fig. 8) using the optimal step *k* = 8 of the 24 Eutherian mammal sequences. As shown in Fig. 8, we can observe that all transferrin sequences (red) were clustered into two distinct clades, except rabbit transferrin sequence was grouped with lactoferrin class. Similarly, all lactoferrin sequences (green) were clustered together, except mouse lactoferrin sequence was grouped with transferrin class. Phylogenetic tree (Fig. 8) generated by our approach showed better clade distribution based on transferrin and lactoferrin categories compared with previous work^62^ which is shown in Figs S14 and S15.

### Phylogenetic tree analysis using 58 genome datasets from different species

**Figure 9:**
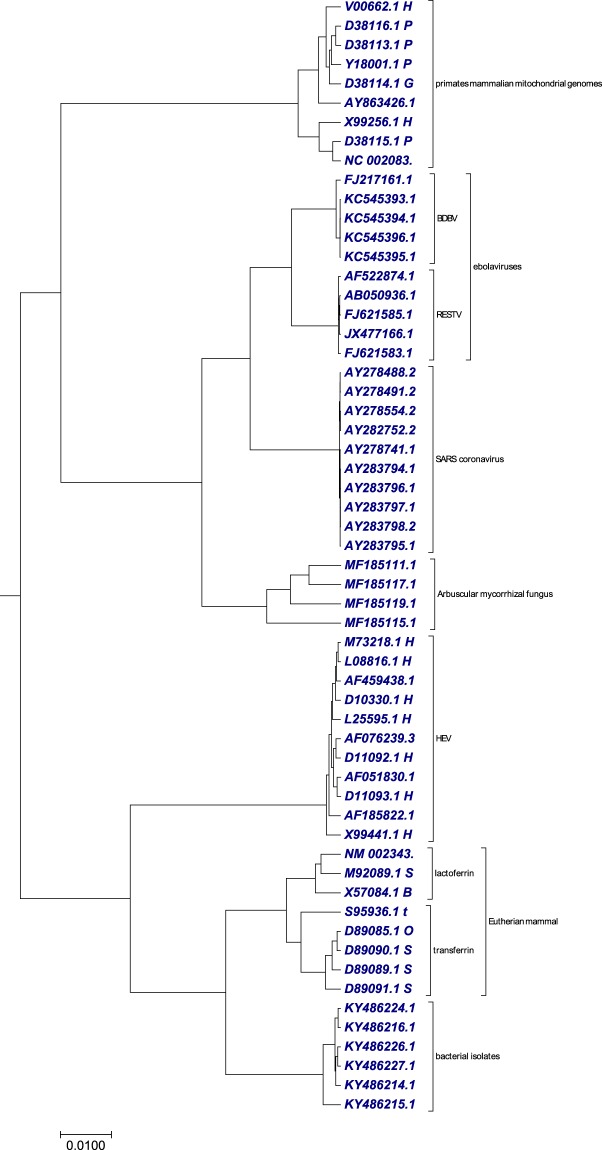
The phylogenetic tree of 58 genome datasets from different species constructed using our method.

To verify the clustering efficiency of our method on extremely divergent sequences from different organisms, we randomly collected genomes of varying length from different datasets from Tables S1, S2, S3, S4, S5, S7 and S8. The genomes included in this dataset were, four arbuscular mycorrhizal fungi, six bacterial isolates, nine primates mammalian mitochondrial genomes, ten ebolavirus (five reston virus (RESTV), five bundibugyo virus (BDBV)) complete genomes, ten SARS coronavirus, eleven hepatitis E virus and eight eutherian mammals. We applied our proposed method to generate the phylogenetic tree (Fig. 9) using the optimal step *k* = 8. As shown in Fig. 9, we observed that all the different species genomes were clustered separately. This result (Fig. 9) showed the efficiency of our method in clustering genomes irrespective of their size and divergence. Our result (Fig. 9) was in consensus with the result generated using ClustalW method (Fig. S16). The time taken by our method to generate the transition probability matrix was less than 1 second, while Clustalw has taken 3 hours and 12 minutes.

### Phylogenetic tree analysis using 29 Escherichia/Shigella complete genomes

**Figure 10:**
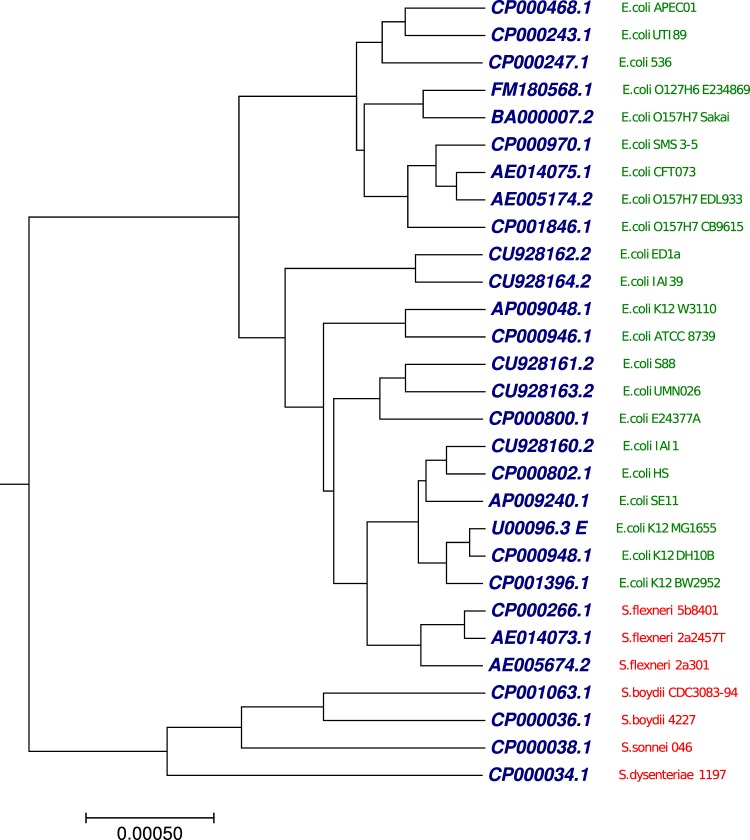
The phylogenetic tree of 29 Escherichia/Shigella whole genomes constructed using our method.

The other benchmark dataset used in this study was 29 complete genomes from the genera Escherichia/Shigella with more than 1 million nucleotides (Table S9). We applied our proposed method to generate the phylogenetic tree (Fig. 10) using the optimal step *k* = 7. As shown in Fig. 10, we observed that the genomes were clustered into distinct clades, Escherichia(green) and Shigella (red). We took the benchmark tree^64^ as a reference which is based on concatenated alignments of the 2034 core genes and used the maximum likelihood method to infer the phylogenetic relationships. We calculated Robinson-Foulds distance (RF-distance)^65^ of the tree produced by our method against the benchmark tree^64^. The RF-distance is often used to compare two trees of closely related species. Since, the species in this dataset (29 complete genomes from the genera Escherichia/Shigella) are closely related organisms, therefore, we employed the RF-distance, which evaluates the topological congruence between an inferred tree and a benchmark tree. We also collected the generated RF-distances from the previous study^66^. RF = 0 indicates that, the test-tree topology is completely similar to that of the benchmark tree, while similarity level decreases as the RF increases. As shown in Fig. S17, RF-distance generated by our approach to the reference tree was higher than RF-distance generated by rest of the methods to the reference tree. This result demonstrates that our proposed method has a limitation in clustering of the closely related organism.

## Conclusion

This study focused on fuzzy integral similarity technique based on Markov chain and applied this algorithm to phylogenetic tree analysis. Sequence comparison is one of the most useful and widely practiced methods in bioinformatics and computational biology. Alignment based methods perform well if the genetic sequences are homologous. High mutation rates and genetic recombination brings in a limitation of the alignment based method. Also at the genomic scale, alignment based methods become impractical due to their computational complexity. Alignment-free methods are of great value, because they reduce the technical constraints of alignments. We constructed a transition probability matrix using a Markov chain for each DNA sequences without performing prior alignment at the genomic scale. The fuzzy integral similarity technique is a method that can calculate similarity score between two transition probability matrices of DNA sequences. The main advantage of our approach is that, it does not require any additional parameters, which makes it fully automated and robust. We implemented and tested our method on suitable datasets.

All programs are implemented on a linux server with 384 GB RAM with 24 dual core processor. Our proposed approach is fast in computational speed (Table 1) compare to alignment-based method, ClustalW and also faster as compared to various alignment-free methods, which were discussed above. For the large datasets such as, 30 bacteria and 29 Escherichia/Shigella, which ClustalW can not handle, while our alignment-free method take only 3 seconds to produce transition probability matrices for both the datasets.

**Table 1:** Running time comparison.

Method	AMF isolates	Bacterial isolates	Mammals	Ebolavirus	Coronavirus	Bacteria	HEV	Eutherian mammals	Mixed genomes	Escherichia/Shigella
Our method	<1 s	<1 s	<1 s	<1 s	<1 s	3 s	<1 s	<1 s	<1 s	3 s
ClustalW	1 s	1 min 8 s	4 h 75 min	10 h 28 min	5 h 23 min	—	1 h 28 min	2 min 13 s	3 h 12 m	—
Multiple encoding vector method	—	—	0.12 s	6 min 42 s	0.34 s	—	—	—	—	—
Fourier power spectrum method	—	—	—	—	6 s	9 min 41 s	—	—	—	—

**Figure 11:**
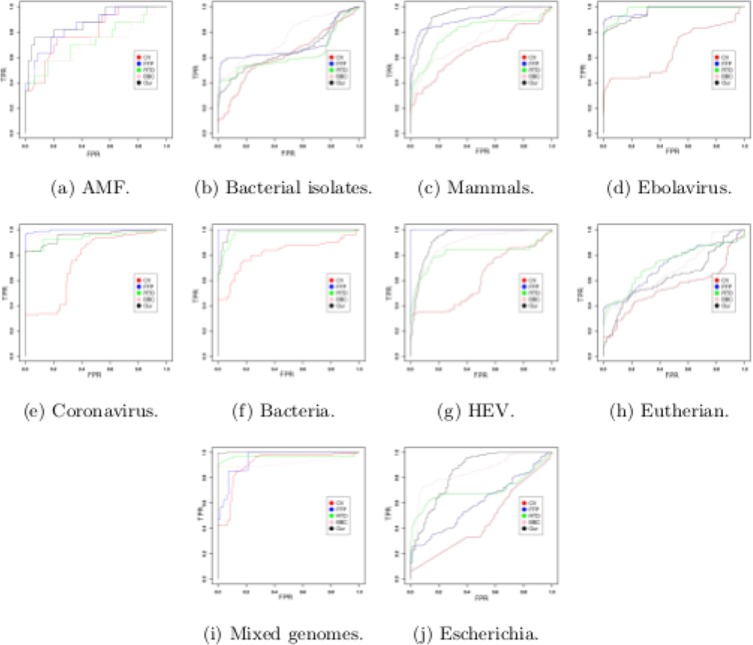
Receiver operating characteristic curve (ROC) on the given datasets.

In this study, we plotted ROC curve^39–41^ (Fig. 11) and calculate area under the ROC curve (AUC) using distance matrices generated by our method and other alignment-free methods from Alfree repository^2^. The detail discussion of the ROC results (Fig. 11) and AUC analysis for all benchmark datasets are given in ROC_Supplementary Material. It may be observed that, while we have similar AUC values as the other methods, the phylogenetic tree generated by our method outperforms the other existing methods. The result shows clear accuracy in terms of AUC of our method as the other methods and superiority in terms of phylogenetic clustering. Moreover, the superiority of our method can be observed from the execution time in Figs 30 and 48 (ROC_Supplementary Material) for the large sequence length data.

Our proposed method is faster and has the potential to build phylogenetic tree for large sized genomes, such as, mammalian genome. Mammalian genomes are divided into several chromosomes. In this study, we selected chromosome X to do the phylogenetic analysis, details are given in Table S10. Our dataset included the species: human (*Homo sapiens*), monkey (*Macaca mulatta*), chimpanzee (*Pan troglodytes*), gorilla (*Gorilla gorilla*), horse (*Equus caballus*), mouse (*Mus musculus*), dog (*Canis familiaris*), opossum (*Monodelphis domesticus*), and platypus (*Ornithorhynchus anatinus*). The length of the chromosomes X in these organisms ranges approximately from 6 to 147 Mb. Applying our method, we generated the phylogenetic tree (Fig. S18) of nine mammals using the optimal step *k* = 8. Our method took only 18 seconds for generating transition probability matrix. Phylogenetic tree (Fig. S19) generated by multiple encoding vector method^52^, mouse clustered with primates, while dog and horse came together in a clade. Figure S18 generated by our method formed three major clusters. Branch point in the first clade shows a divergence event of horse and mouse from the primates. A comparative radiation hybrid map of chromosome X of human, horse and mouse reveals many conserved syntenies between the three species^67,68^. This similarity may have placed horse and mouse in a sister group. Dog and oppossum formed a distinct clade, while platypus separated as an outgroup.

Based on the results generated by our developed method, we found that our method performed well on divergent sequences, rather than closely related sequences. Therefore, this approach would be beneficial for the users to generate hypothesis that can be investigated in further detail with subsequent analysis. Before continuing research for further development of our method, we must keep in mind that, this approach is a probabilistic measure in nature, and can be modified by incorporating more information, such as, nucleotides substitution, insertion, deletion, genetic recombination, Physicochemical Properties of nucleotides etc., in sequences. Overall, our goal in this study was to introduce a new methodology and a new tool to the comparative genomics research community. This proposed work can be used to guide the development of more powerful measures for sequence comparison.

## Supplementary information

Dataset 1

Dataset 2

Dataset 3

Dataset 4

Dataset 5

## Data Availability

The datasets used in this paper are available in the supplementary table and the C source code in this paper is freely available to the public upon request.

## References

[1] Vinga S, Almeida J (2003). Alignment-free sequence comparison—a review. Bioinforma..

[2] Zielezinski A, Vinga S, Almeida J, Karlowski WM (2017). Alignment-free sequence comparison: benefits, applications, and tools. Genome Biol..

[3] Bernard, G. *et al*. Alignment-free inference of hierarchical and reticulate phylogenomic relationships. *Briefings Bioinforma*. bbx067 (2017). 10.1093/bib/bbx067 PMC6433738 28673025

[4] Bromberg R, Grishin NV, Otwinowski Z (2016). Phylogeny reconstruction with alignment-free method that corrects for horizontal gene transfer. PLOS Comput. Biol..

[5] Didier G (2007). Comparing sequences without using alignments: application to hiv/siv subtyping. BMC Bioinforma..

[6] Chatterji, S., Yamazaki, I., Bai, Z. & Eisen, J. A. *Compostbin: A dna composition-based algorithm for binning environmental shotgun reads*. 17–28 (Springer Berlin Heidelberg, Berlin, Heidelberg, 2008).

[7] Meinicke P (2015). Uproc: tools for ultra-fast protein domain classification. Bioinforma..

[8] Tanaseichuk O, Borneman J, Jiang T (2012). Separating metagenomic short reads into genomes via clustering. Algorithms for Mol. Biol..

[9] Teeling H, Waldmann J, Lombardot T, Bauer M, Glöckner FO (2004). Tetra: a web-service and a stand-alone program for the analysis and comparison of tetranucleotide usage patterns in dna sequences. BMC Bioinforma..

[10] Wang Y, Leung HC, Yiu S, Chin FY (2012). Metacluster 5.0: a two-round binning approach for metagenomic data for low-abundance species in a noisy sample. Bioinforma..

[11] Wu Y-W, Ye Y (2011). A novel abundance-based algorithm for binning metagenomic sequences using l-tuples. J. Comput. Biol..

[12] Federico M, Leoncini M, Montangero M, Valente P (2012). Direct vs 2-stage approaches to structured motif finding. Algorithms for Mol. Biol..

[13] Kantorovitz MR, Robinson GE, Sinha S (2007). A statistical method for alignment-free comparison of regulatory sequences. Bioinforma..

[14] Leung G, Eisen MB (2009). Identifying cis-regulatory sequences by word profile similarity. Plos One.

[15] Lingner T, Meinicke P (2006). Remote homology detection based on oligomer distances. Bioinforma..

[16] Lingner T, Meinicke P (2008). Word correlation matrices for protein sequence analysis and remote homology detection. BMC Bioinforma..

[17] Zerbino DR, Birney E (2008). Velvet: Algorithms for de novo short read assembly using de bruijn graphs. Genome Res..

[18] Rob Patro SMM, Kingsford C (2014). Sailfish enables alignment-free isoform quantification from rna-seq reads using lightweight algorithms. Nat. Biotechnol..

[19] Drouin A (2016). Predictive computational phenotyping and biomarker discovery using reference-free genome comparisons. BMC Genomics.

[20] Haubold B (2014). Alignment-free phylogenetics and population genetics. Briefings Bioinforma..

[21] Blaisdell BE (1991). Average values of a dissimilarity measure not requiring sequence alignment are twice the averages of conventional mismatch counts requiring sequence alignment for a variety of computer-generated model systems. J. Mol. Evol..

[22] Sims GE, Jun S-R, Wu GA, Kim S-H (2009). Alignment-free genome comparison with feature frequency profiles (ffp) and optimal resolutions. Proc. Natl. Acad. Sci..

[23] Kolekar P, Kale M, Kulkarni-Kale U (2012). Alignment-free distance measure based on return time distribution for sequence analysis: Applications to clustering, molecular phylogeny and subtyping. Mol. Phylogenetics Evol..

[24] Hatje, K. & Kollmar, M. A phylogenetic analysis of the brassicales clade based on an alignment-free sequence comparison method. *Front Plant Sci*. **3** (2012). 10.3389/fpls.2012.00192 PMC3429886 22952468

[25] Lu G, Zhang S, Fang X (2008). An improved string composition method for sequence comparison. BMC Bioinforma..

[26] Gao L, Qi J (2007). Whole genome molecular phylogeny of large dsdna viruses using composition vector method. BMC Evol. Biol..

[27] Wu X, Wan X-F, Wu G, Xu D, Lin G (2006). Phylogenetic analysis using complete signature information of whole genomes and clustered neighbour-joining method. Int. J. Bioinforma. Res. Appl..

[28] Ulitsky I, Burstein D, Tuller T, Chor B (2006). The average common substring approach to phylogenomic reconstruction. J. Comput. Biol..

[29] Comin M, Verzotto D (2012). Alignment-free phylogeny of whole genomes using underlying subwords. Algorithms for Mol. Biol..

[30] Haubold B, Pierstorff N, Möller F, Wiehe T (2005). Genome comparison without alignment using shortest unique substrings. BMC Bioinforma..

[31] Thankachan SV, Chockalingam SP, Yongchao L, Alberto A, Srinivas A (2016). Alfred: A practical method for alignment-free distance computation. J. Comput. Biol..

[32] Leimeister C-A, Morgenstern B (2014). Kmacs: the k-mismatch average common substring approach to alignment-free sequence comparison. Bioinforma..

[33] Torra V, Narukawa Y (2006). The interpretation of fuzzy integrals and their application to fuzzy systems. Int. J. Approx. Reason..

[34] Medhi, J. *Stochastic Processes* (New Age Science, 2009).

[35] Garcia F, Lopez FJ, Cano C, Blanco A (2009). Fisim: A new similarity measure between transcription factor binding sites based on the fuzzy integral. BMC Bioinforma..

[36] Zhang S, Zhang Y, Gutman I (2013). Analysis of dna sequences based on the fuzzy integral. Match Commun. Math. Comput. Chem..

[37] Sims JR, Zhenyuan W (1990). Fuzzy measures and fuzzy integrals: An overview. Int. J. Gen. Syst..

[38] Felsenstein J (1989). Phylip–phylogeny inference package (version 3.2). Cladistics.

[39] Swets J (1988). Measuring the accuracy of diagnostic systems. Sci..

[40] Nemes S, Hartel T (2010). Summary measures for binary classification systems in animal ecology. North-Western J. Zool..

[41] Sonego P, Kocsor A, Pongor S (2008). Roc analysis: applications to the classification of biological sequences and 3d structures. Briefings Bioinforma..

[42] Durbin, R., Eddy, S. R., Krogh, A. & Mitchison, G. *Biological Sequence Analysis* (Cambridge University Press, Cambridge, 1998).

[43] Zadeh L (1965). Fuzzy sets. Inf. Control..

[44] Sugeno, M. Theory of Fuzzy Integrals and Its Applications (Doct. Thesis, Tokyo Institute of Technology, Tokyo, 1974).

[45] Sugeno, M. *Fuzzy measures and fuzzy integrals: A survey*, 89–102 (North Holland, New York, 1997).

[46] Chaira, T. *Fuzzy Measures in Image Processing*, 587–606 (Springer Berlin Heidelberg, Berlin, Heidelberg, 2008).

[47] Carugo O, Pongor S (2001). A normalized root-mean-spuare distance for comparing protein three-dimensional structures. Protein Sci..

[48] C. Brundrett M (2002). Coevolution of roots and mycorrhizas of land plants. New Phytol..

[49] Kumar S, Stecher G, Tamura K (2016). Mega7: Molecular evolutionary genetics analysis version 7.0 for bigger datasets. Mol. Biol. Evol..

[50] Bulgarelli D, Schlaeppi K, Spaepen S, van Themaat EVL, Schulze-Lefert P (2013). Structure and functions of the bacterial microbiota of plants. Annu. Rev. Plant Biol..

[51] Stucky BJ (2012). Seqtrace: A graphical tool for rapidly processing dna sequencing chromatograms. J. Biomol. Tech..

[52] Li, Y., He, L., He, R. L. & Yau, S. S.-T. A novel fast vector method for genetic sequence comparison. *Sci. Reports* **7** (2017). 10.1038/s41598-017-12493-2 PMC5610321 28939913

[53] Gire SK (2014). Genomic surveillance elucidates ebola virus origin and transmission during the 2014 outbreak. Sci..

[54] Holmes EC, Dudas G, Rambaut A, Andersen KG (2016). The evolution of ebola virus: Insights from the 2013–2016 epidemic. Nat..

[55] Leibowitz, J. L. *Coronaviruses: Molecular and Cellular Biology*, 693–694 (Caister Academic Press, 2008).

[56] King, M. Q., Adams, M. J., Carstens, E. B. & Lefkowitz, E. J. (eds). *Family - Coronaviridae*, 806–828 (Elsevier, San Diego, 2012).

[57] Greenwood, D., Barer, M., Slack, R. & Irving, W. (Elsevier, Churchill Livingstone, 2012).

[58] Hoang T (2015). A new method to cluster dna sequences using fourier power spectrum. J. Theor. Biol..

[59] Yang K, Zhang L (2008). Performance comparison between k -tuple distance and four model-based distances in phylogenetic tree reconstruction. Nucleic Acids Res..

[60] Meng XJ (2010). Recent advances in hepatitis e virus. J. Viral Hepat..

[61] Li L (2009). Full-genome nucleotide sequence and analysis of a chinese swine hepatitis e virus isolate of genotype 4 identified in the guangxi zhuang autonomous region: Evidence of zoonotic risk from swine to human in south china. Liver Int..

[62] Liu L, Li C, Bai F, Zhao Q, Wang Y (2015). An optimization approach and its application to compare dna sequences. J. Mol. Struct..

[63] Ford MJ (2001). Molecular evolution of transferrin: Evidence for positive selection in salmonids. Mol. Biol. Evol..

[64] Zhou Z (2010). Derivation of *escherichia coli* o157:h7 from its o55:h7 precursor. Plos One.

[65] Robinson D, Foulds L (1981). Comparison of phylogenetic trees. Math. Biosci..

[66] Morgenstern B, Zhu B, Horwege S, Leimeister CA (2015). Estimating evolutionary distances between genomic sequences from spaced-word matches. Algorithms for Mol. Biol..

[67] Chowdhary BP (2003). The first-generation whole-genome radiation hybrid map in the horse identifies conserved segments in human and mouse genomes. Genome Res..

[68] Raudsepp T (2004). Exceptional conservation of horse–human gene order on x chromosome revealed by high-resolution radiation hybrid mapping. Proc. Natl. Acad. Sci..

