# Supplementary material for: Alignment-free method for DNA sequence clustering using Fuzzy integral similarity
Source: Sci Rep. 2019 Mar 6;9:3753. doi: 10.1038/s41598-019-40452-6 (PMC6403383; doi:10.1038/s41598-019-40452-6)
Supplement: Supplementary file 1 — Dataset 1 [file 41598_2019_40452_MOESM1_ESM.pdf]

Alignment-free method for DNA sequence clustering using Fuzzy integral similarity

Ajay Kumar Saw[1], Garima Raj[2], Manashi Das[3], Narayan C. Talukdar[4], Binod Chandra Tripathy[5] and Soumyadeep Nandi[6,]*

[1] Mathematical Sciences Division, Institute of Advanced Study in Science and Technology, Guwahati-35, India.

[2,3,4,6] Life Science Division, Institute of Advanced Study in Science and Technology, Guwahati-35, India.

[5] Mathematics Department, Tripura University, Tripura, India.

* To whom correspondence should be addressed. Email: soumyadeep.nandi@gmail.com; snandi@iasst.gov.in

Supplementary material (Tables)

Table S1: Information of the 11 Arbuscular mycorrhizal fungus(AMF) isolates.

Accession	Abbreviation	Division	Family	Genus	Name	length
MF185110	4 Rhi in	Glomeromycota	Glomeraceae	Rhizophagus	Rhizophagus intraradices	282
MF185111	10 Gl spp	Glomeromycota	Glomeraceae	Glomus	Glomus spp	235
MF185112	11 Rhi cl	Glomeromycota	Glomeraceae	Rhizophagus	Rhizophagus clarus	288
MF185113	12 Gi ma	Glomeromycota	Gigasporaceae	Gigaspora	Gigaspora margarita	307
MF185114	13 Gi gi	Glomeromycota	Gigasporaceae	Gigaspora	Gigaspora gigantea	332
MF185115	14 Glo sp	Glomeromycota	Glomeraceae	Glomus	Glomus Sp	336
MF185116	15 Rhi in	Glomeromycota	Glomeraceae	Rhizophagus	Rhizophagus intraradices	300
MF185117	16 Glo sp	Glomeromycota	Glomeraceae	Glomus	Glomus sp	216
MF185119	18 Glo sp	Glomeromycota	Glomeraceae	Glomus	Glomus sp	297
MF185109	1 Uncl glu	Glomeromycota	Unclassified	Unclassified	Unclutured Glomeromycota sp	282
MF185118	17 Glomer	Glomeromycota	Unclassified	Unclassified	Uncultured Glomeromycota sp	290

Table S2: Information of the 16S rDNA sequences of 40 bacterial isolates.

Sample IDs (Accession)	closest strain	phylum	class	order	family	length
KJ 2 (KY486204)	Methylobacterium indicum strain SE2.11	Proteobacteria	Alphaproteobacteria	Rhizobiales	Methylobacteriaceae	1104
KJ 3 (KY486205)	Xanthomonas sacchari LMG 471	Proteobacteria	Gammaproteobacteria	Xanthomonadales	Xanthomonadaceae	761
KJ 5 (KY486206)	Xanthomonas sacchari LMG 471	Proteobacteria	Gammaproteobacteria	Xanthomonadales	Xanthomonadaceae	1452
KJ 6 (KY486207)	Knoellia flava TL1	actinobacteria	Actinobacteridae	Actinomycetales	Intrasporangiaceae	1253
KJ 34 (KY486218)	Microbacterium proteolyticum	actinobacteria	Actinobacteridae	Actinomycetales	Microbacteriaceae	1195
KJ 35 (KY486219)	Pseudomonas oryzihabitans NBRC 102199	Proteobacteria	Gammaproteobacteria	Pseudomonadales	Pseudomonadaceae	1099
KJ 36 (KY927407)	Bacillus marisflavi TF11	Firmicutes	Bacilli	Bacillales	Bacillaceae	718
KJ 37 (KY486220)	Brevibacillus borstelensis NBRC 15714	Firmicutes	Bacilli	Bacillales	Paenibacillaceae	1335
KJ 38 (KY486221)	Pantoea agglomerans DSM 3493	Proteobacteria	Gammaproteobacteria	Enterobacteriales	Enterobacteriaceae	1339
KJ 40 (KY486222)	Xanthomonas sacchari LMG 471	Proteobacteria	Gammaproteobacteria	Xanthomonadales	Xanthomonadaceae	1337
KJ 41 (KY486223)	Curtobacterium luteum DSM 20542	actinobacteria	Actinobacteridae	Actinomycetales	Microbacteriaceae	1334
IDO 14 (KY486209)	Luteibacter yeojuensis R2A16-10	Proteobacteria	Gammaproteobacteria	Xanthomonadales	Rhodanobacteraceae	1366
IDO 15 (KY486210)	Pantoea agglomerans DSM3493	Proteobacteria	Gammaproteobacteria	Enterobacteriales	Enterobacteriaceae	1356
IDO d10 (KY486232)	Pantoea agglomerans DSM3493	Proteobacteria	Gammaproteobacteria	Enterobacteriales	Enterobacteriaceae	1350
IDO d21 (KY019246)	Pantoea agglomerans DSM3493	Proteobacteria	Gammaproteobacteria	Enterobacteriales	Enterobacteriaceae	1345
IDO d5 (KY013009)	Pantoea agglomerans DSM3493	Proteobacteria	Gammaproteobacteria	Enterobacteriales	Enterobacteriaceae	1351
IDO d6 (KY927404)	Curtobacterium plantarum CL63	actinobacteria	Actinobacteridae	Actinomycetales	Microbacteriaceae	742
RN 17 (KY486211)	Pantoea allii BD390	Proteobacteria	Gammaproteobacteria	Enterobacteriales	Enterobacteriaceae	1365
RN d26 (KY013011)	Staphylococcus warneri AW25	Firmicutes	Bacilli	Bacillales	Staphylococcaceae	1031
RN d3 (KY019245)	Pantoea agglomerans DSM3493	Proteobacteria	Gammaproteobacteria	Enterobacteriales	Enterobacteriaceae	1345
RN d17(KY013010)	Bacillus subtilis JCM1465	Firmicutes	Bacilli	Bacillales	Bacillaceae	1341
FN 10 (KY486208)	Pantoea agglomerans NBRC 102470	Proteobacteria	Gammaproteobacteria	Enterobacteriales	Enterobacteriaceae	1269
FN 23 (KY486212)	Curtobacterium plantarum	actinobacteria	Actinobacteridae	Actinomycetales	Microbacteriaceae	1364
FN 24 (KY486213)	Xanthomonas sacchari LMG471	Proteobacteria	Gammaproteobacteria	Xanthomonadales	Xanthomonadaceae	1387
FN 48 (KY486228)	Pantoea agglomerans DSM3493	Proteobacteria	Gammaproteobacteria	Enterobacteriales	Enterobacteriaceae	1356
KEK 42 (KY486224)	Pantoea agglomerans DSM3493	Proteobacteria	Gammaproteobacteria	Enterobacteriales	Enterobacteriaceae	1346
KEK 44 (KY486225)	Pantoea agglomerans DSM 3493	Proteobacteria	Gammaproteobacteria	Enterobacteriales	Enterobacteriaceae	1294
KEK 45 (KY486226)	Pantoea agglomerans DSM 3493	Proteobacteria	Gammaproteobacteria	Enterobacteriales	Enterobacteriaceae	1347

KEK 46 (KY927405)	Pantoea anthophila LMG 2558	Proteobacteria	Gammaproteobacteria	Enterobacteriales	Enterobacteriaceae	753
KEK 47 (KY486227)	Pantoea agglomerans DSM 3493	Proteobacteria	Gammaproteobacteria	Enterobacteriales	Enterobacteriaceae	1345
T KW 27 (KY927408)	Curtobacterium plantarum CL63	actinobacteria	Actinobacteridae	Actinomycetales	Microbacteriaceae	785
T KW 28 (KY486214)	Pantoea agglomerans DSM 3493	Proteobacteria	Gammaproteobacteria	Enterobacteriales	Enterobacteriaceae	1411
T KW 29 (KY927406)	Curtobacterium plantarum CL63	actinobacteria	Actinobacteridae	Actinomycetales	Microbacteriaceae	785
T KW 31 (KY486215)	Pantoea agglomerans DSM 3493	Proteobacteria	Gammaproteobacteria	Enterobacteriales	Enterobacteriaceae	1319
T KW 32 (KY486216)	Pantoea agglomerans DSM 3493	Proteobacteria	Gammaproteobacteria	Enterobacteriales	Enterobacteriaceae	1345
T KW 33 (KY486217)	Pantoea agglomerans DSM 3493	Proteobacteria	Gammaproteobacteria	Enterobacteriales	Enterobacteriaceae	1341
T KW 51 (KY486229)	Pantoea agglomerans DSM 3493	Proteobacteria	Gammaproteobacteria	Enterobacteriales	Enterobacteriaceae	1345
T KW 52 (KY486230)	Pseudomonas psychrotolerans TPs-04	Proteobacteria	Gammaproteobacteria	Pseudomonadales	Pseudomonadaceae	1346
T KW 53 (KY486231)	Pantoea agglomerans DSM 3493	Proteobacteria	Gammaproteobacteria	Enterobacteriales	Enterobacteriaceae	1322
T KW 56 (KY019244)	Pantoea agglomerans DSM 3493	Proteobacteria	Gammaproteobacteria	Enterobacteriales	Enterobacteriaceae	1325

Table S3: Information of the 41 mammalian genomes

Accession Number	Abbreviation	Description	Genome Length (bp)
V00662	Human	Human	16569
D38116	Pig_Chim	Pigmy chimpanzee	16563
D38113	Com_Chim	Common chimpanzee	16554
D38114	Gorilla	Gorilla	16472
X99256	Gibbon	Gibbon	16521
Y18001	Baboon	Baboon	16389
AY863426	Ver_Monkey	Vervet monkey	16586
D38115	Bor_Oran	Bornean orangutan	16389
NC_002083	Sum_Oran	Sumatran orangutan	16499
U20753	Cat	Cat	16364
U96639	Dog	Dog	17009
AJ002189	Pig	Pig	16727
AF010406	Sheep	Sheep	16680
AF533441	Goat	Goat	16616
V00654	Cow	Cow	16640
AY488491	Buffalo	Buffalo	16338
EU442884	Wolf	Wolf	16355
EF551003	Tiger	Tiger	16774
EF551002	Leopard	Leopard	16990
X97336	Indian_Rhino	Indian Rhinoceros	16964
Y07726	White_Rhino	White Rhinoceros	16829
DQ402478	Black_Bear	Black Bear	16832
AF303110	Brown_Bear	Brown Bear	16868
AF303111	Polar_Bear	Polar Bear	17020
EF212882	Giant_Panda	Giant Panda	17017
AJ001588	Rabbit	Rabbit	16805
X88898	Hedgehog	Hedgehog	17245
NC_002764	Macaca_Thibet	Macaca Thibet	17447
AJ238588	Squirrel	Squirrel	16602
AJ001562	Dormouse	Dormouse	16507
X72204	Blue_Whale	Blue whale	16402
NC_005268	Bowhead Whale	Bowhead Whale	16390
NC_007441	chiru	chiru	16498
NC_008830	Common warthog	Common warthog	16719

NC_001788	donkey	donkey	16670
NC_001321	Fin Whale	Fin Whale	16398
NC_005270	Gray Whale	Gray Whale	16412
NC_001640	horse	horse	16660
NC_005275	Indus River Dolphin	Indus River Dolphin	16324
NC_006931	North Pacific Right Whale	North Pacific Right Whale	16386
NC_010640	Taiwan serow	Taiwan serow	16524

Table S4: Accesion number of 59 ebolaviruses

Accession Number	Species	Genome Length (bp)
FJ217161	BDBV_2007_FJ217161	18941
KC545393	BDBV_2012_KC545393	18940
KC545395	BDBV_2012_KC545395	18939
KC545394	BDBV_2012_KC545394	18938
KC545396	BDBV_2012_KC545396	18937
FJ217162	TAFV_1994_FJ217162	18936
AF522874	RESTV_1990_AF522874	18935
AB050936	RESTV_1996_AB050936	18934
JX477166	RESTV_1996_JX477166	18933
FJ621585	RESTV_2008_FJ621585	18932
FJ621583	RESTV_2008_FJ621583	18931
JX477165	RESTV_2009_JX477165	18930
FJ968794	SUDV_1976_FJ968794	18929
KC242783	SUDV_1979_KC242783	18928
EU338380	SUDV_2004_EU338380	18927
AY729654	SUDV_2000_AY729654	18926
JN638998	SUDV_2011_JN638998	18925
KC545389	SUDV_2012_KC545389	18924
KC545390	SUDV_2012_KC545390	18923

KC545391	SUDV_2012_KC545391	18922
KC545392	SUDV_2012_KC545392	18921
KC589025	SUDV_2012_KC589025	18920
KC242801	EBOV_1976_KC242801	18919
NC_002549	EBOV_1976_NC002549	18918
KC242791	EBOV_1977_KC242791	18917
KC242792	EBOV_1994_KC242792	18916
KC242793	EBOV_1996_KC242793	18915
KC242794	EBOV_1996_KC242794	18914
AY354458	EBOV_1995_AY354458	18913
KC242796	EBOV_1995_KC242796	18912
KC242799	EBOV_1995_KC242799	18911
KC242784	EBOV_2007_KC242784	18910
KC242786	EBOV_2007_KC242786	18909
KC242787	EBOV_2007_KC242787	18908
KC242789	EBOV_2007_KC242789	18907
KC242785	EBOV_2007_KC242785	18906
KC242790	EBOV_2007_KC242790	18905
KC242788	EBOV_2007_KC242788	18904
KC242800	EBOV_2002_KC242800	18903
KM034555	EBOV_2014_G3676	18902
KM034562	EBOV_2014_G3686	18901
KM233039	EBOV_2014_EM112	18900
KM034557	EBOV_2014_G3677	18899
KM034560	EBOV_2014_G3682	18898
KM233050	EBOV_2014_G3713	18897
KM233053	EBOV_2014_G3724	18896
KM233057	EBOV_2014_G3735	18895
KM233063	EBOV_2014_G3764	18894

KM233072	EBOV_2014_G3782	18893
KM233110	EBOV_2014_G3848	18892
KM233070	EBOV_2014_G3770	18891
KM233099	EBOV_2014_G3825	18890
KM233097	EBOV_2014_G3823	18889
KM233109	EBOV_2014_G3846	18888
KM233096	EBOV_2014_G3822	18887
KM233103	EBOV_2014_G3831	18886
KJ660346	EBOV_2014_KJ660346	18885
KJ660347	EBOV_2014_KJ660347	18884
KJ660348	EBOV_2014_KJ660348	18883

Table S5: Information of the 30 coronavirus genomes and the four outgroup genomes

**Accession Number**	**Abbreviation**	**Description**	**Length (bp)**
AF304460	1_HCoV-229E	Human coronavirus 229E, Group 1	27317
AF353511	1_PEDV	Porcine epidemic diarrhea virus strain, Group 1	28033
NC_005831	1_HCoV-NL63	Human coronavirus NL63, Group 1	27553
AY391777	2_HCoV-OC43	Human coronavirus OC43, Group 2	30738
U00735	2_BCoVM	Bovine coronavirus strain Mebus, Group 2	31032
AF391542	2_BCoVL	Bovine coronavirus isolate BCoV-LUN, Group 2	31028
AF220295	2_BCoVQ	Bovine coronavirus strain Quebec, Group 2	31100
NC_003045	2_BCoV	Bovine coronavirus, Group 2	31028
AF208067	2_MHVM	Murine hepatitis virus strain ML-10, Group 2	31233
AF201929	2_MHV2	Murine hepatitis virus strain 2, Group 2	31276
AF208066	2_MHVP	Murine hepatitis virus strain Penn 97-1, Group 2	31112
NC_001846	2_MHV	Murine hepatitis virus, Group 2	31357
NC_001451	3_IBV	Avian infectious bronchitis virus, Group 3	27608
EU095850	3_TCoV	Turkey coronavirus isolate MG10, Group 3	27657
AY278488	4_BJ01	SARS coronavirus BJ01, Group 4	29725
AY278741	4_Urbani	SARS coronavirus Urbani, Group 4	29727
AY278491	4_HKU-39849	SARS coronavirus HKU-39849, Group 4	29742
AY278554	4_CUHK-W1	SARS coronavirus CUHK-W1, Group 4	29736
AY282752	4_CUHK-Su10	SARS coronavirus CUHK-Su10, Group 4	29736
AY283794	4_SIN2500	SARS coronavirus isolate SIN2500, Group 4	29711
AY283795	4_SIN2677	SARS coronavirus isolate SIN2677, Group 4	29705
AY283796	4_SIN2679	SARS coronavirus isolate SIN2679, Group 4	29711
AY283797	4_SIN2748	SARS coronavirus isolate SIN2748, Group 4	29706
AY283798	4_SIN2774	SARS coronavirus isolate SIN2774, Group 4	29711
AY291451	4_TW1	SARS coronavirus TW1, Group 4	29729
NC_004718	4_TOR2	SARS coronavirus TOR2, Group 4	29751
AY297028	4_ZJ01	SARS coronavirus ZJ01, Group 4	29715

AY572034	4_Civet007	SARS coronavirus civet007, Group 4	29540
AY572035	4_Civet010	SARS coronavirus civet010, Group 4	29518
NC_006577	5_HCoV-HKU1	Human coronavirus HKU1, Group 5	29926
NC_001564	out_CellF	Cell fusing agent virus, *Flaviviridae* outgroup	10695
NC_004102	out_HepaCF	Hepatitis C virus, *Flaviviridae* outgroup	9646
NC_001512	out_NyongT	O'nyong-nyong virus, *Togaviridae* outgroup	11835
NC_001544	out_RossT	Ross River virus, *Togaviridae* outgroup	11657

Table S6: Information of the 30 bacterial genomes

**Family**	**Species**	**Accession number**	**Genome Length(bp)**
Bacillaceae	Bacillus anthracis str. A0248	CP001598.1	4983359
Bacillaceae	Bacillus anthracis str. Ames	AE016879.1	5062406
Bacillaceae	Bacillus anthracis str. CDC 684	CP001215.1	5141453
Bacillaceae	Bacillus anthracis str. Sterne	AE017225.1	5220500
Borreliaceae	Borrelia duttonii Ly	CP000976.1	4904312
Borreliaceae	Borrelia hermsii DAH	CP000048.1	5299547
Borreliaceae	Borrelia recurrentis A1	CP000993.1	5378594
Borreliaceae	Borrelia turicatae 91E135	CP000049.1	5457641
Clostridiaceae	Clostridium perfringens ATCC 13124	CP000246.1	5536688
Clostridiaceae	Clostridium perfringens SM101	CP000312.1	5615735
Clostridiaceae	Clostridium perfringens str. 13 DNA	BA000016.3	5694782
Desulfovibrionaceae	Desulfovibrio vulgaris DP4	CP000527.1	5773829
Desulfovibrionaceae	Desulfovibrio vulgaris Hildenborough	AE017285.1	5931923
Desulfovibrionaceae	Desulfovibrio vulgaris RCH1	CP002297.1	5852876
Burkholderiaceae	Ralstonia eutropha H16	AM260480.1	6722393
Burkholderiaceae	Ralstonia eutropha JMP134	CP000091.1	6801440
Rhodobacteraceae	Rhodobacter sphaeroides ATCC 17029	CP000578.1	6959534
Rhodobacteraceae	Rhodobacter sphaeroides KD131	CP001151.1	7038581
Staphylococcaceae	Staphylococcus carnosus subsp. carnosus TM300	AM295250.1	7275722
Staphylococcaceae	Staphylococcus epidermidis ATCC 12228	AE015929.1	7354769
Staphylococcaceae	Staphylococcus haemolyticus JCSC1435 DNA	AP006716.1	7512863
Staphylococcaceae	Staphylococcus lugdunensis HKU09-01	CP001837.1	7591910
Yersiniaceae	Yersinia pestis CO92	AL590842.1	7750004
Yersiniaceae	Yersinia pestis D106004	CP001585.1	7829051
Yersiniaceae	Yersinia pestis KIM10+	AE009952.1	7908098
Yersiniaceae	Yersinia pestis Z176003	CP001593.1	7987145
Enterobacteriaceae	Escherichia coli ABU 83972	CP001671.1	6327158
Enterobacteriaceae	Escherichia coli APEC O1	CP000468.1	6406205
Enterobacteriaceae	Shigella flexneri 2002017	CP001383.1	7117628
Enterobacteriaceae	Shigella flexneri 2a str. 301	AE005674.2	7196675

Table S7: Information of the 48 Hepatitis E virus(HEV)

**Strain name**	**AC**	**Genotype**
B1(Bur-82)	M73218	I
B2(Bur-86)	D10330	I
I2(Mad-93)	X99441	I
I3	AF076239	I
NP1(TK15/92)	AF051830	I
P2(Abb-2B)	AF185822	I
Yam-67	AF459438	I

C1(CHT-88)	D11092	I
C2(KS2-87)	L25595	I
C3(CHT-87)	L08816	I
C4(Uigh179)	D11093	I
China Hebei	M94177	I
P1(Sar-55)	M80581	I
I1(FHF)	X98292	I
Morocco	AY230202	I
T3	AY204877	I
M1	M74506	II
HE-JA10	AB089824	III
JKN-Sap	AB074918	III
JMY-HAW	AB074920	III
SW-US1	AF082843	III
US1	AF060668	III
US2	AF060669	III
ARKELL	AY115488	III
JBOAR1-HYO04	AB189070	III
JDEER-HYO03L	AB189071	III
JJT-KAN	AB091394	III
JMO-HYO03L	AB189072	III
JRA1	AP003430	III
JSO-HYO03L	AB189073	III
JTH-HYO03L	AB189074	III
JYO-HYO03L	AB189075	III
SWJ570	AB073912	III
KYRGYZ	AF455784	III
HE-JA1	AB097812	IV
HE-JK4	AB099347	IV
HE-JI4	AB080575	IV
JAK-Sai	AB074915	IV
JKK-SAP	AB074917	IV
JSM-SAP95	AB161717	IV
JSN-SAP-FH	AB091395	IV
JSN-SAP-FH02C	AB200239	IV
JTS-SAP02	AB161718	IV
JYW-SAP02	AB161719	IV
SWJ13–1	AB097811	IV
SWCH25	AY594199	IV
T1	AJ272108	IV
CCC220	AB108537	IV

Table S8: Information of the 24 Eutherian mammal

No.	Sequence Name	Species	Accession No.
1	Rabbit TF	*Oryctolagus coniculus*	X58533
2	Human TF	*Homo sapien*	S95936
3	Cow TF	*Bos Taurus*	U02564
4	Rat TF	*Rattus norvegicus*	D38380
5	Mouse LF	*Mus musculus*	NM_008522
6	Human LF	*H. sapiens*	NM_002343
7	Goat LF	*Capra hircus*	X78902
8	Buffalo LF	*Bubalus arnee*	AJ005203
9	Cow LF	*Bos Taurus*	X57084
10	Camel LF	*Camelus dromedaries*	AJ131674
11	Pig LF	*Sus scrofa*	M92089
12	Possum TF	*Trichosurus vulpecula*	AF092510
13	Frog TF	*Xenopus laevis*	X54530
14	Japanese flounder TF	*Paralichthys olivaceus*	D88801
15	Chinook salmon TF	*Oncorhynchus tshawytscha*	AH008271
16	Atlantic salmon TF	*Salmo salar*	L20313
17	Brown trout TF	*Salmo trutta*	D89091
18	Japanese char TF	*Salvelinus pluvius*	D89088
19	Lake trout TF	*Salvelinus namaycush*	D89090
20	Brook trout TF	*Salvelinus fontinalis*	D89089
21	Amago salmon TF	*Oncorhynchus masou*	D89086
22	Rainbow trout TF	*Oncorhynchus mykiss*	D89083
23	Coho salmon TF	*Oncorhynchus hisutch*	D89084
24	Sockeye salmon TF	*Oncorhynchus nerka*	D89085

Table S9: Information of the *Escherichia/Shigella* 29 genomes

E.coli IAI1	CU928160
E.coli SE11	AP009240
E.coli E24377A	CP000800
S.sonnei 046	CP000038
S.boydii 4227	CP000036
S.boydii CDC3083-94	CP001063
S.flexneri 5b8401	CP000266
S.flexneri 2a2457T	AE014073
S.flexneri 2a301	AE005674
E.coli ATCC 8739	CP000946
E.coli HS	CP000802
E.coli K12 MG1655	U00096
E.coli K12 W3110	AP009048

E.coli K12 DH10B	CP000948
E.coli K12 BW2952	CP001396
S.dysenteriae 1197	CP000034
E.coli O157H7 CB9615	CP001846
E.coli O157H7 EDL933	AE005174
E.coli O157H7 Sakai	BA000007
E.coli UMN026	CU928163
E.coli IAI39	CU928164
E.coli SMS 3-5	CP000970
E.coli O127H6 E234869	FM180568
E.coli 536	CP000247
E.coli ED1a	CU928162
E.coli CFT073	AE014075
E.coli S88	CU928161
E.coli UTI89	CP000243
E.coli APEC01	CP000468

Table S10: Information of the 9 mammalian X chromosomes

Name	Assembly accession	Length (Mb)
Chimpanzee X chromosome	GCF_000001515.6	135.9
Human X chromosome	GCF_000001405.25	151.1
Monkey X chromosome	GCF_000772875.2	145.7
Gorilla X chromosome	GCF_000151905.1	143.0
Dog X chromosome	GCF_000002285.3	123.2
Horse X chromosome	GCF_000002305.2	121.6
Mouse X chromosome	GCF_000001635.25	163.5
Opossum X chromosome	GCF_000002295.2	73.0
Platypus X2 chromosome	GCF_000002275.2	5.5
