# Supplementary material for: Alignment-free method for DNA sequence clustering using Fuzzy integral similarity
Source: Sci Rep. 2019 Mar 6;9:3753. doi: 10.1038/s41598-019-40452-6 (PMC6403383; doi:10.1038/s41598-019-40452-6)
Supplement: Supplementary file 2 — Dataset 2 [file 41598_2019_40452_MOESM2_ESM.pdf]

Alignment-free method for DNA sequence clustering using Fuzzy integral similarity

Ajay Kumar Saw[1], Garima Raj[2], Manashi Das[3], Narayan C. Talukdar[4], Binod Chandra Tripathy[5] and Soumyadeep Nandi[6,*]

[1] Mathematical Sciences Division, Institute of Advanced Study in Science and Technology, Guwahati-35, Assam, India.

[2,3,4,6] Life Science Division, Institute of Advanced Study in Science and Technology, Guwahati-35, Assam, India.

[5] Mathematics Department, Tripura University, Tripura, India.

*To whom correspondence should be addressed. Email: soumyadeep.nandi@gmail.com; snandi@iasst.gov.in

Supplementary material (Figures)

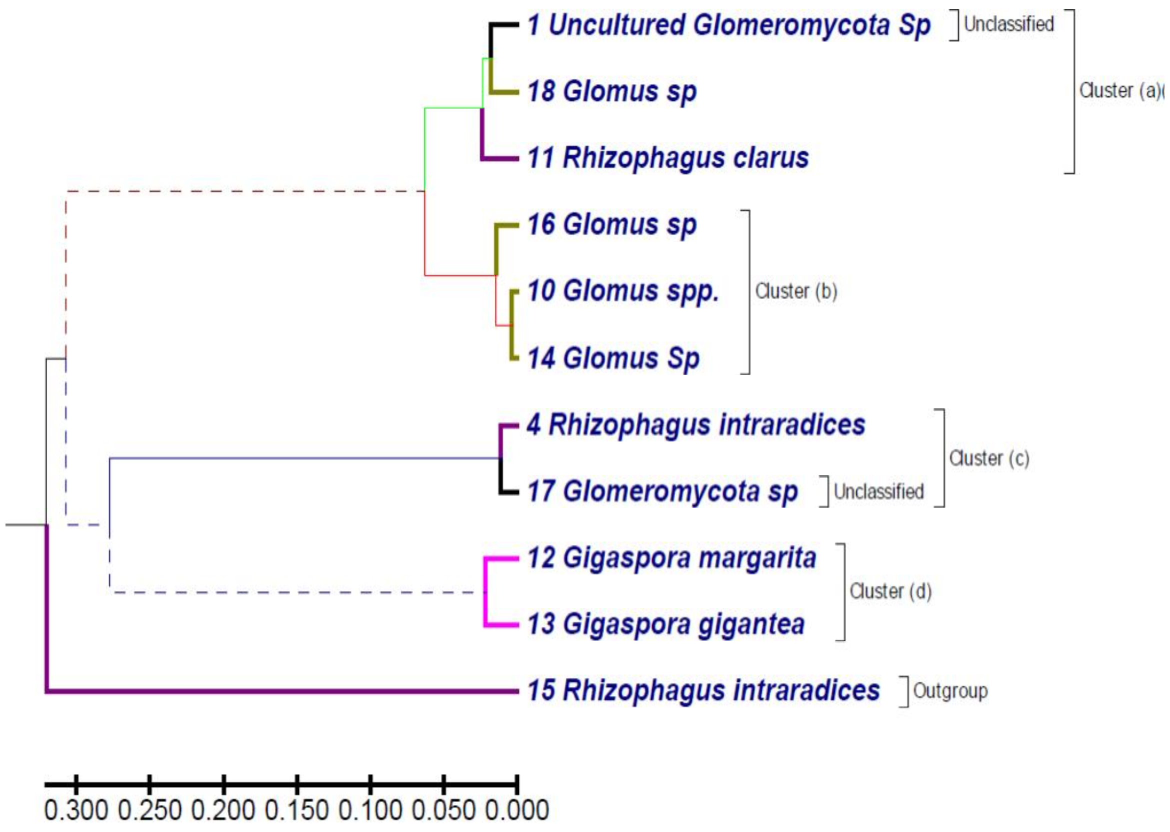

Figure S1: The phylogenetic tree of the 11 AMF sequences constructed by ClustalW method using MEGA package[a].

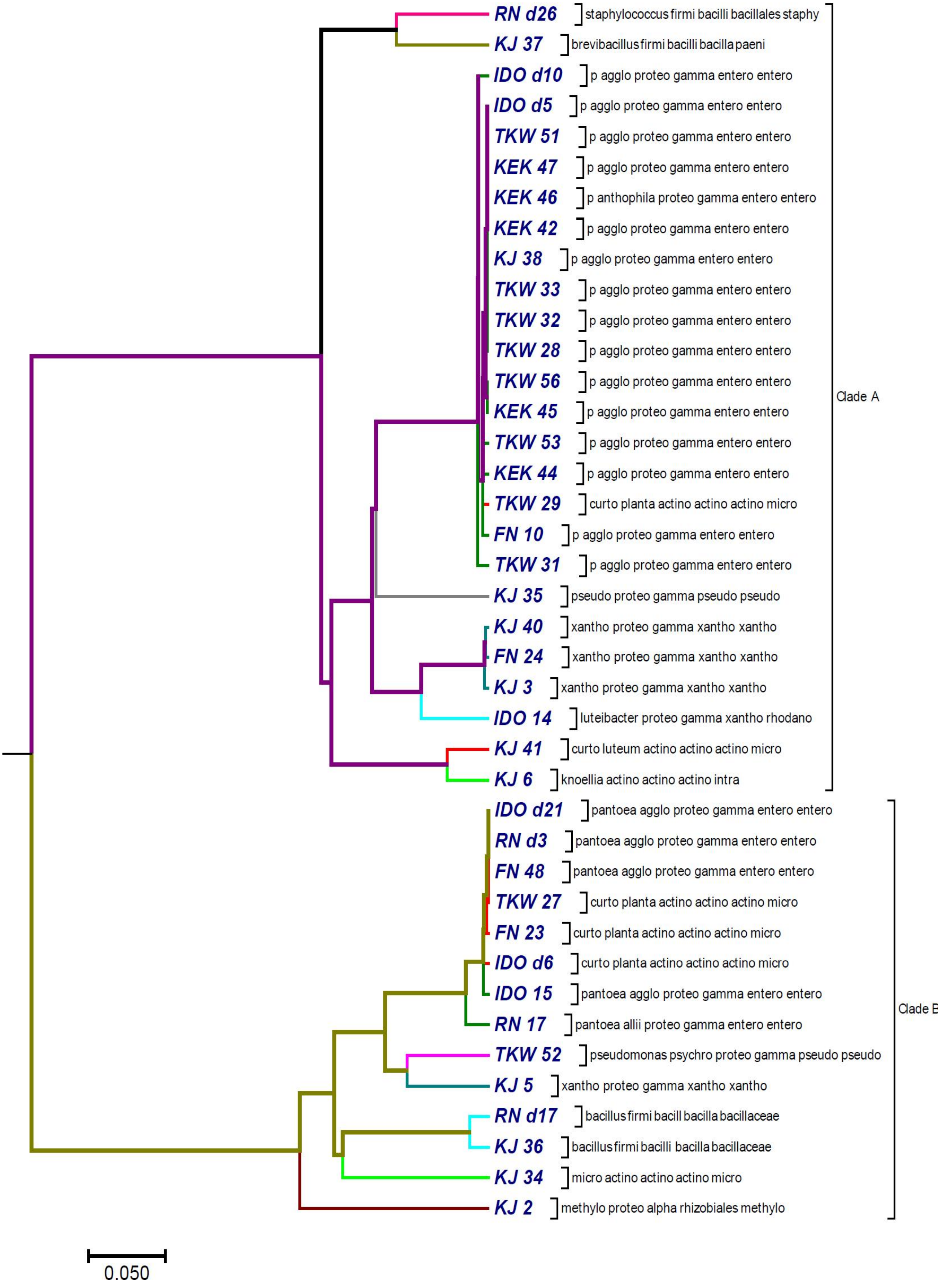

Figure S2: The phylogenetic tree of the 16S rDNA sequences from 40 bacterial isolates constructed by ClustalW method using MEGA package[a].

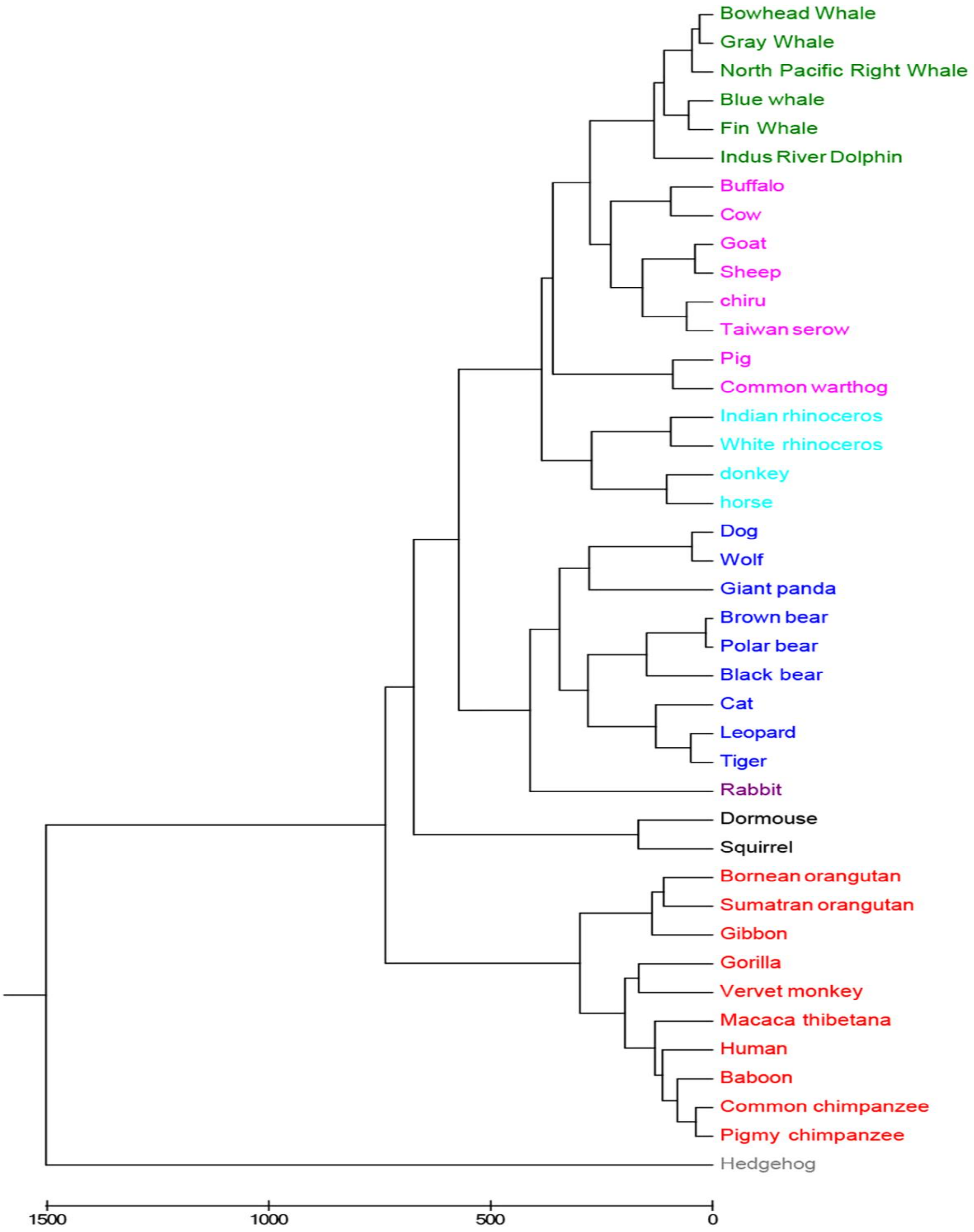

Figure S3: Phylogenetic tree of 41 mitochondrial genome sequences based on multiple encoding vector method[b]. The 8 clusters are *Primates* (red), *Cetacea* (green), *Artiodactyla* (pink), *Perissodactyla* (light green), *Rodentia* (black), *Lagomorpha* (dark red), *Carnivore* (blue), and *Erinaceomorpha* (grey).

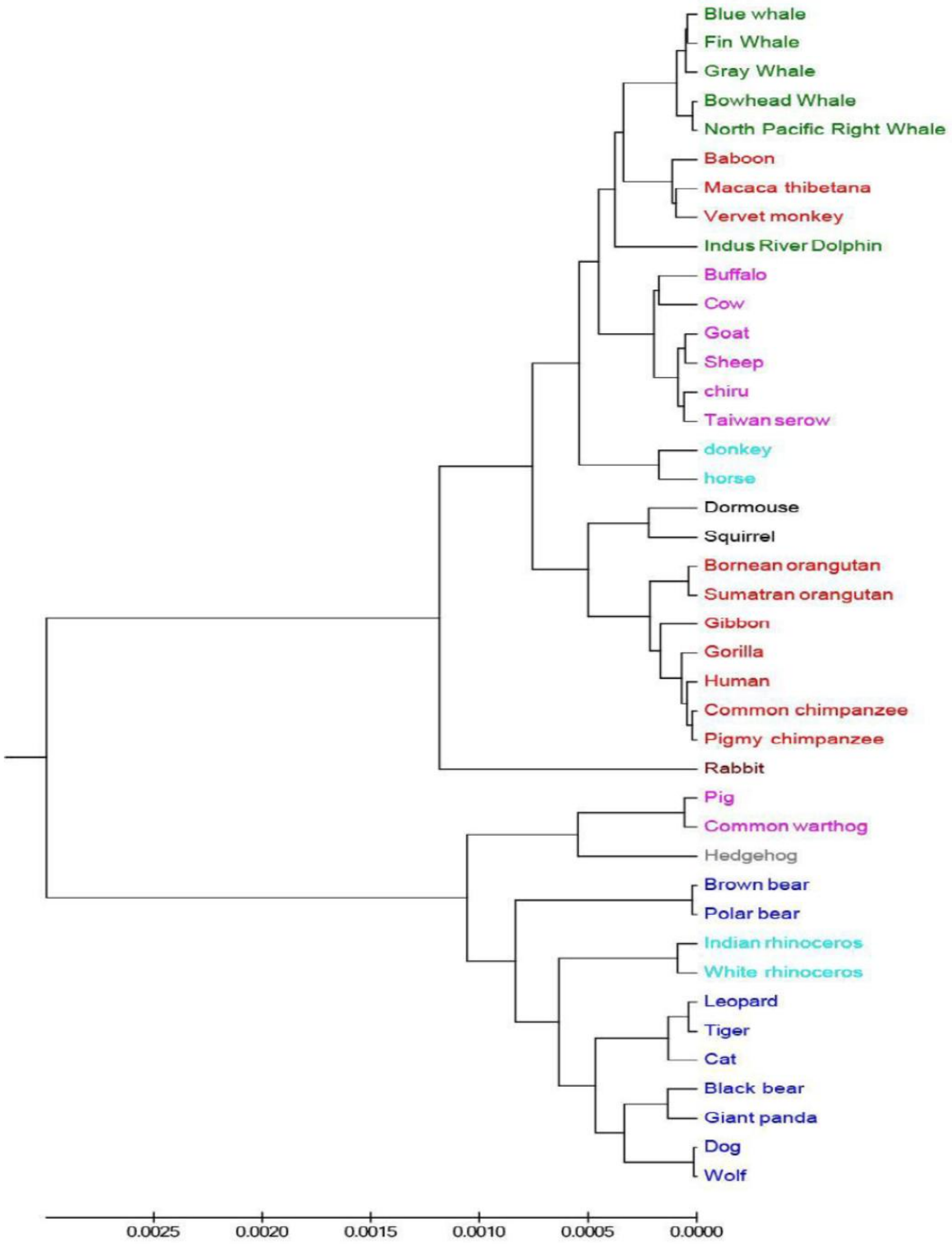

Figure S4: Phylogenetic tree of 41 mitochondrial genome sequences based on feature frequency profiles method using 7 mer[b]. The 8 clusters are *Primates* (red), *Cetacea* (green), *Artiodactyla* (pink), *Perissodactyla* (light green), *Rodentia* (black), *Lagomorpha* (dark red), *Carnivore* (blue), and *Erinaceomorpha* (grey).

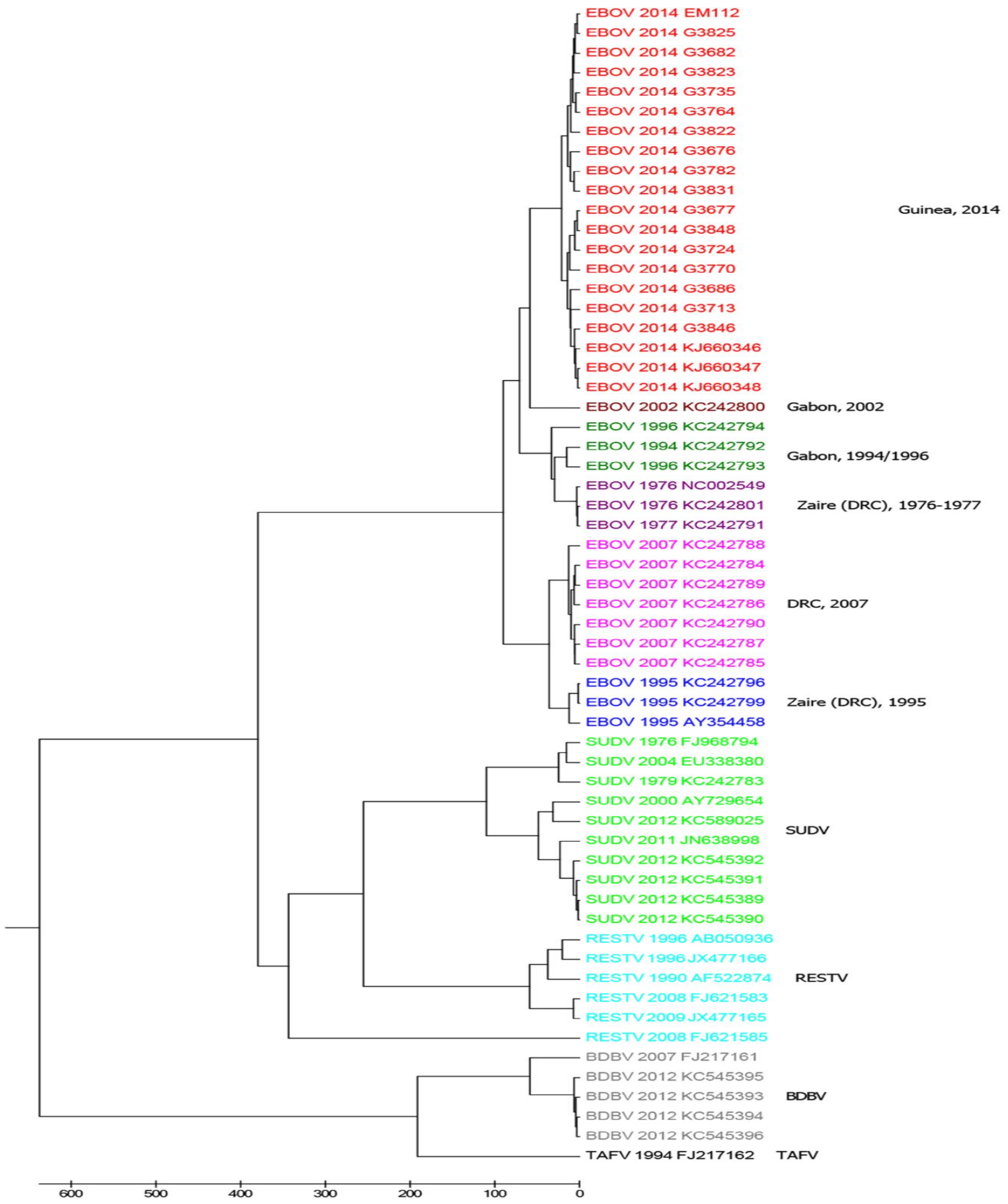

Figure S5: Phylogenetic tree of 59 ebolavirus complete genomes based on multiple encoding vector method[b]. DRC = Democratic Republic of Congo, EBOV = Ebola virus, SUDV = Sudan virus, BDBV = Bundibugyo virus, TAFV = Tai Forest virus, RESTV = Reston virus.

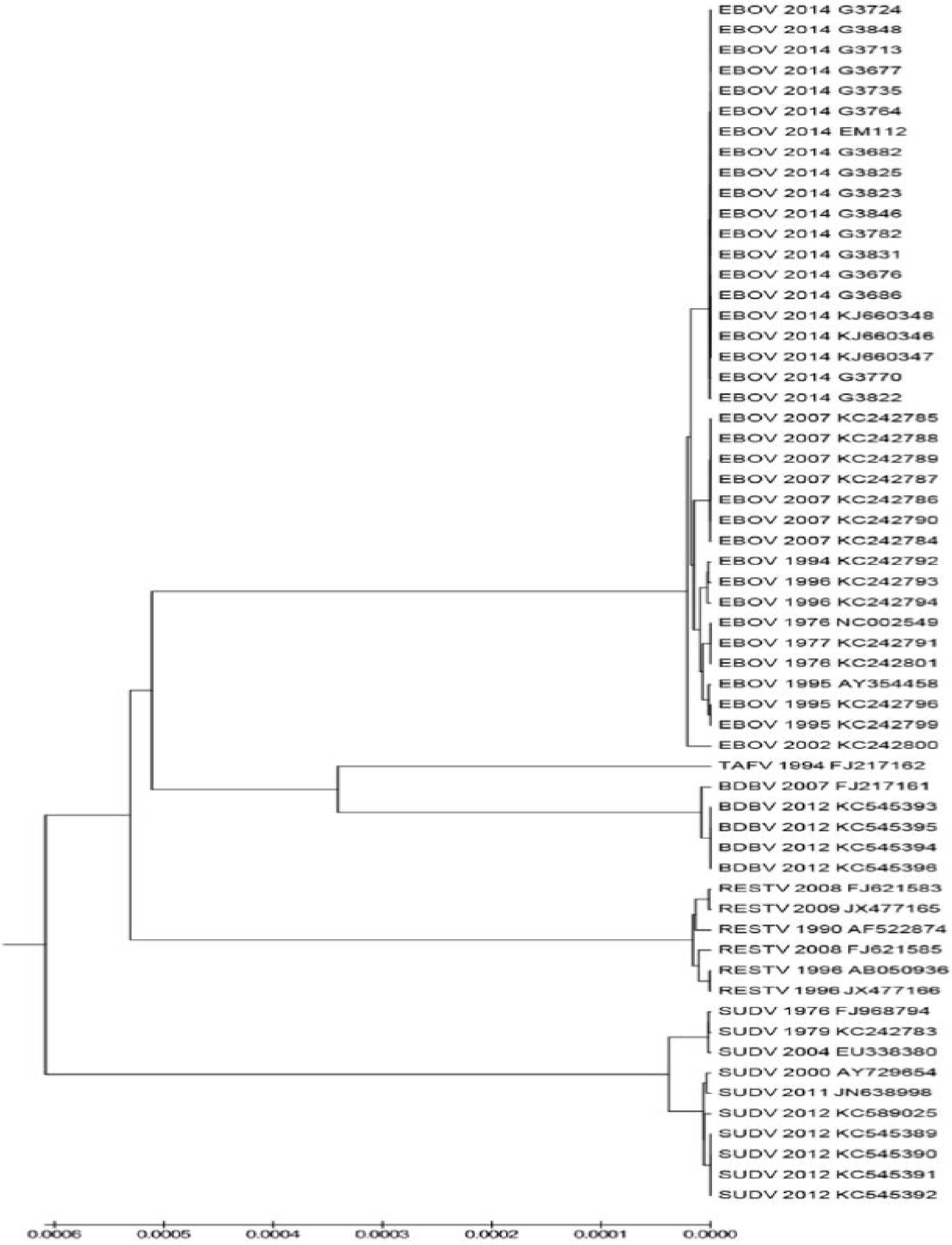

Figure S6: Phylogenetic tree of 59 ebolavirus complete genomes based on Feature frequency profiles method using 7 mer[b]. DRC = Democratic Republic of Congo, EBOV = Ebola virus, SUDV = Sudan virus, BDBV = Bundibugyo virus, TAFV = Tai Forest virus, RESTV = Reston virus.

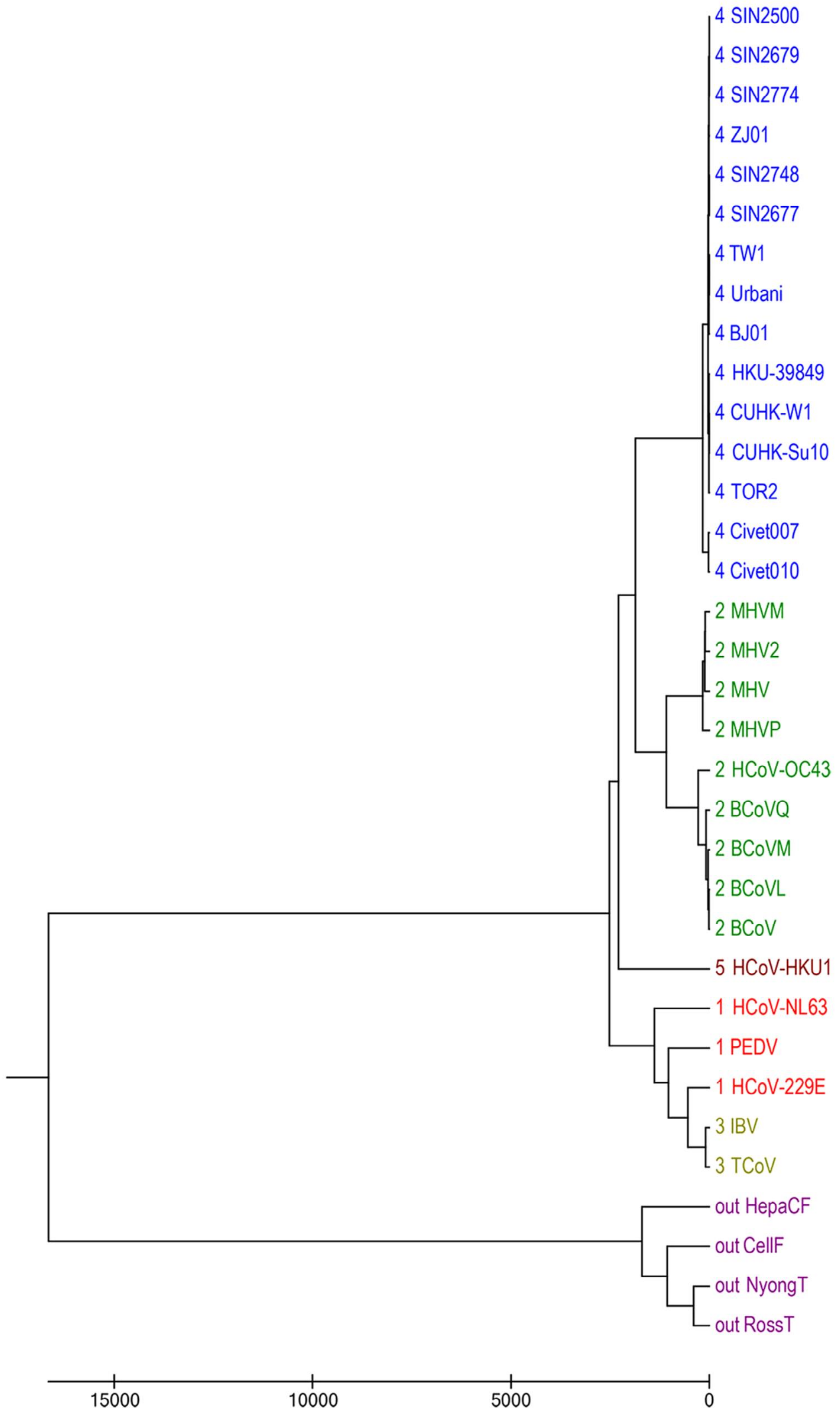

Figure S7: Phylogenetic tree of 30 coronavirus and 4 non-coronavirus whole genomes using the multiple encoding vector method[b].

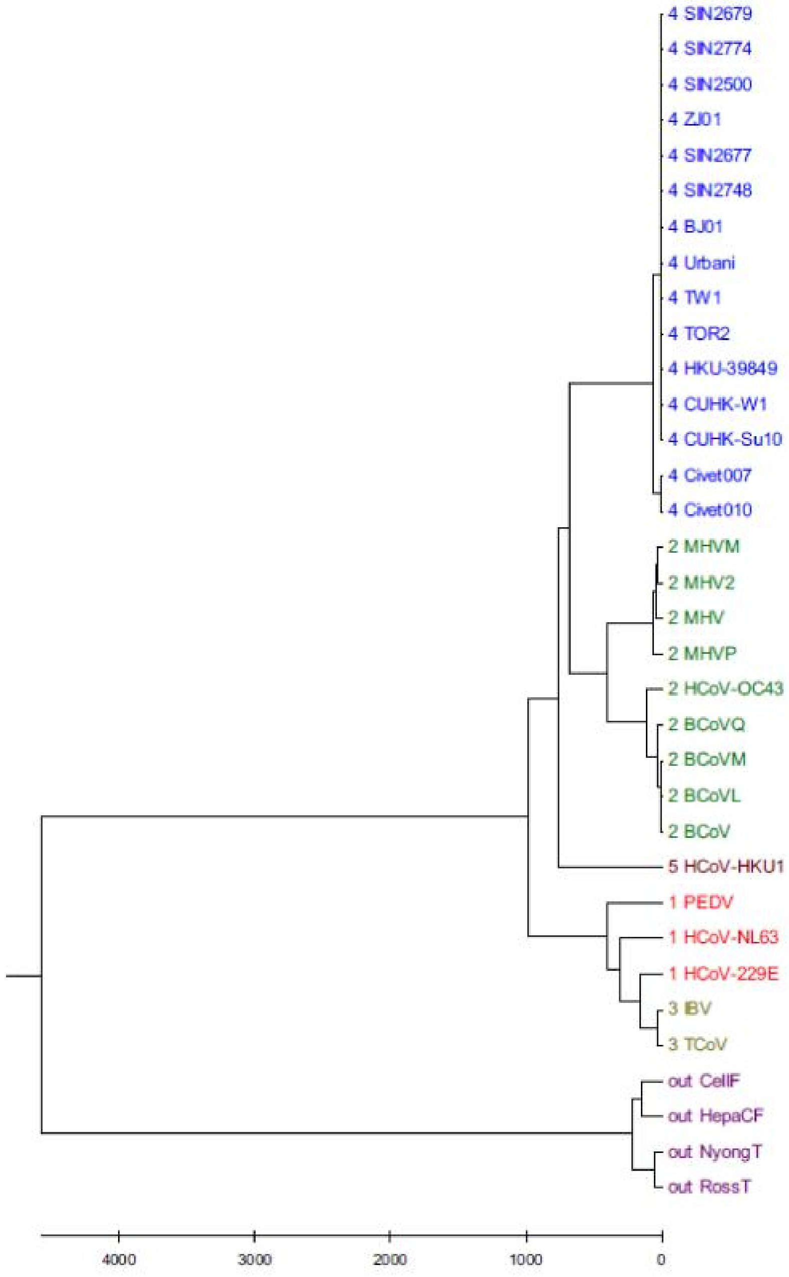

Figure S8: Phylogenetic tree of 30 coronavirus and 4 non-coronavirus whole genomes using the PS method[c].

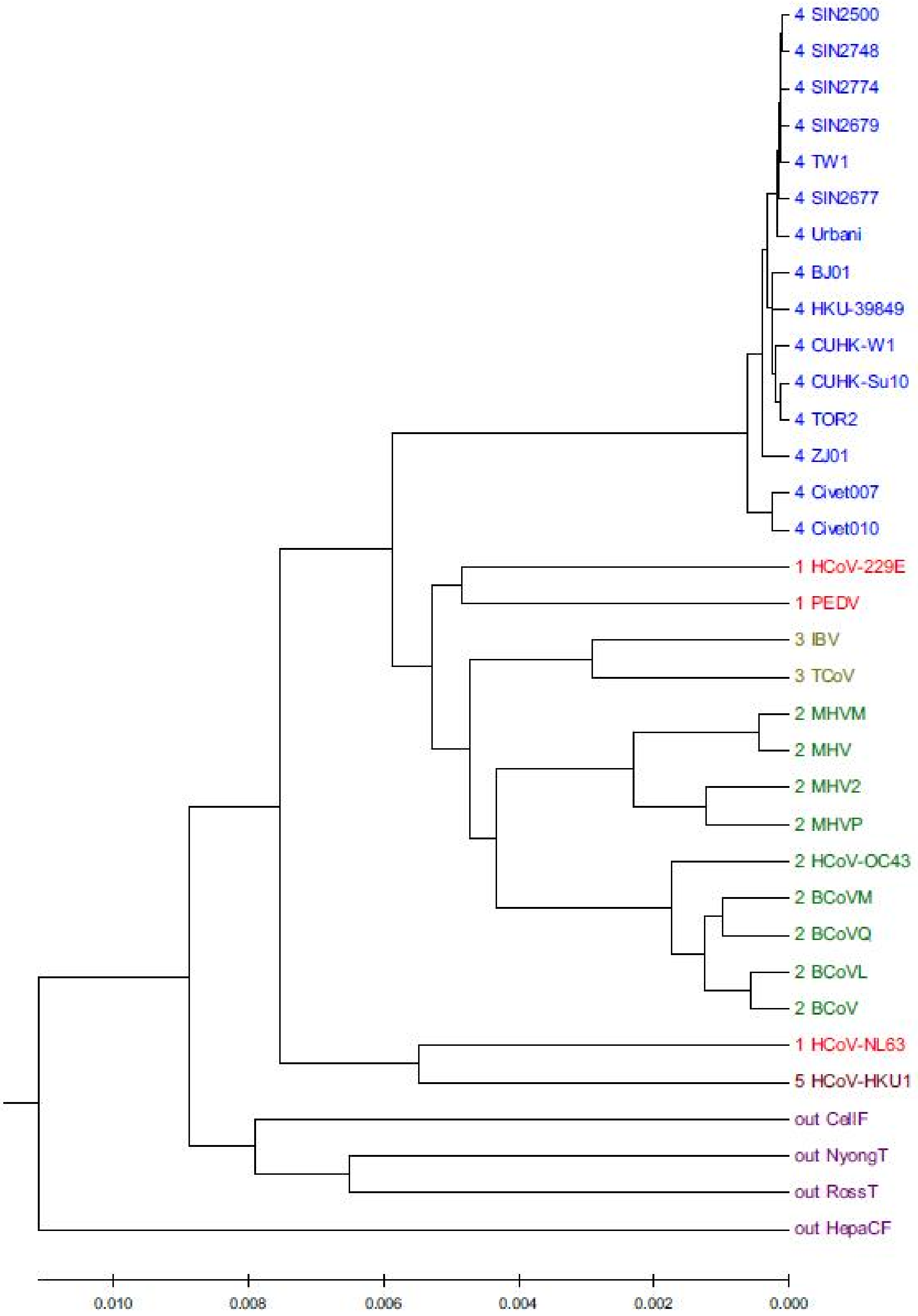

Figure S9: Phylogenetic tree of 30 coronavirus and 4 non-coronavirus whole genomes using the k-mer method, k=6[c].

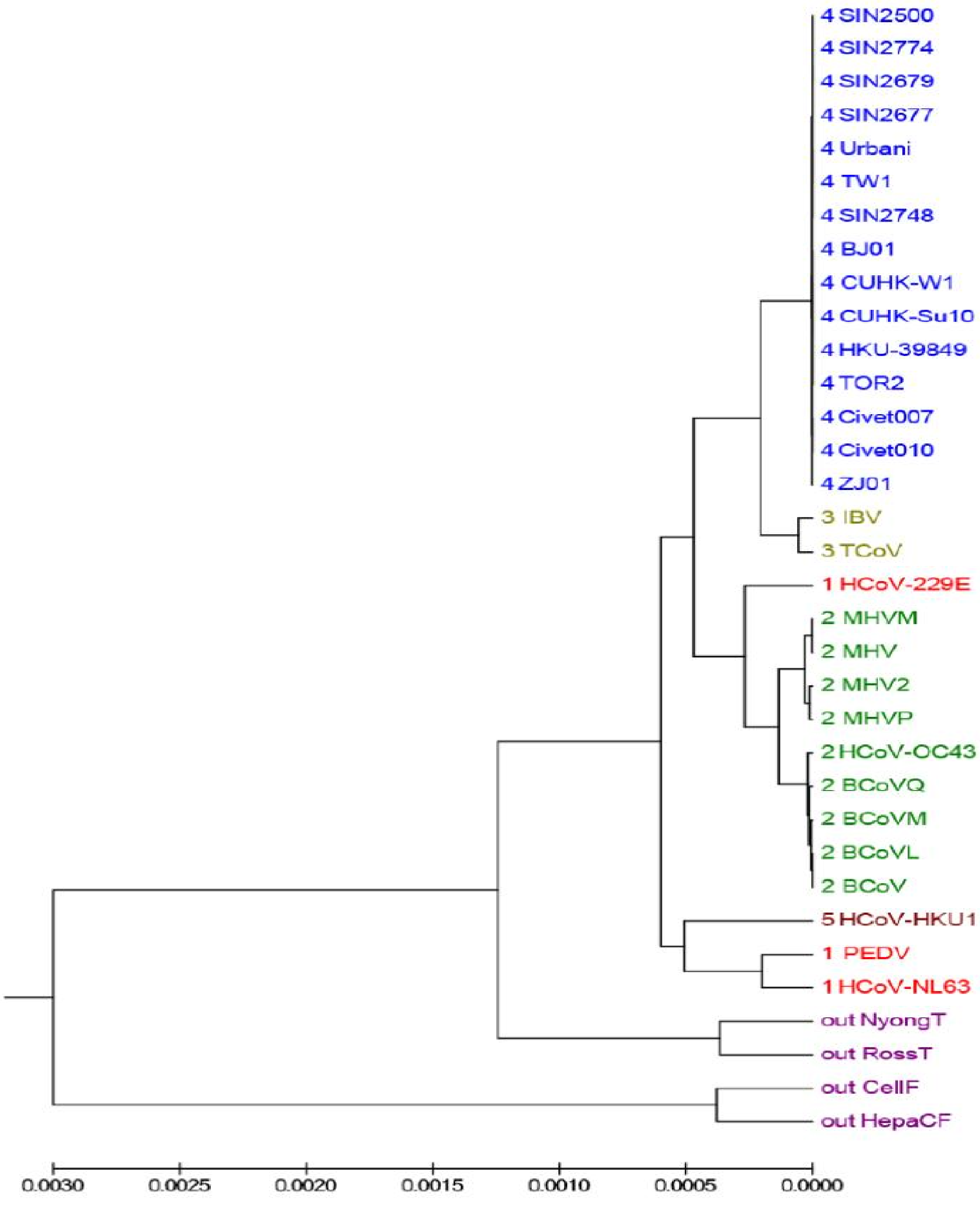

Figure S10: The UPGMA Phylogenetic tree of 30 coronavirus and 4 non-coronavirus whole genomes based on feature frequency profiles method using 6 mer[b].

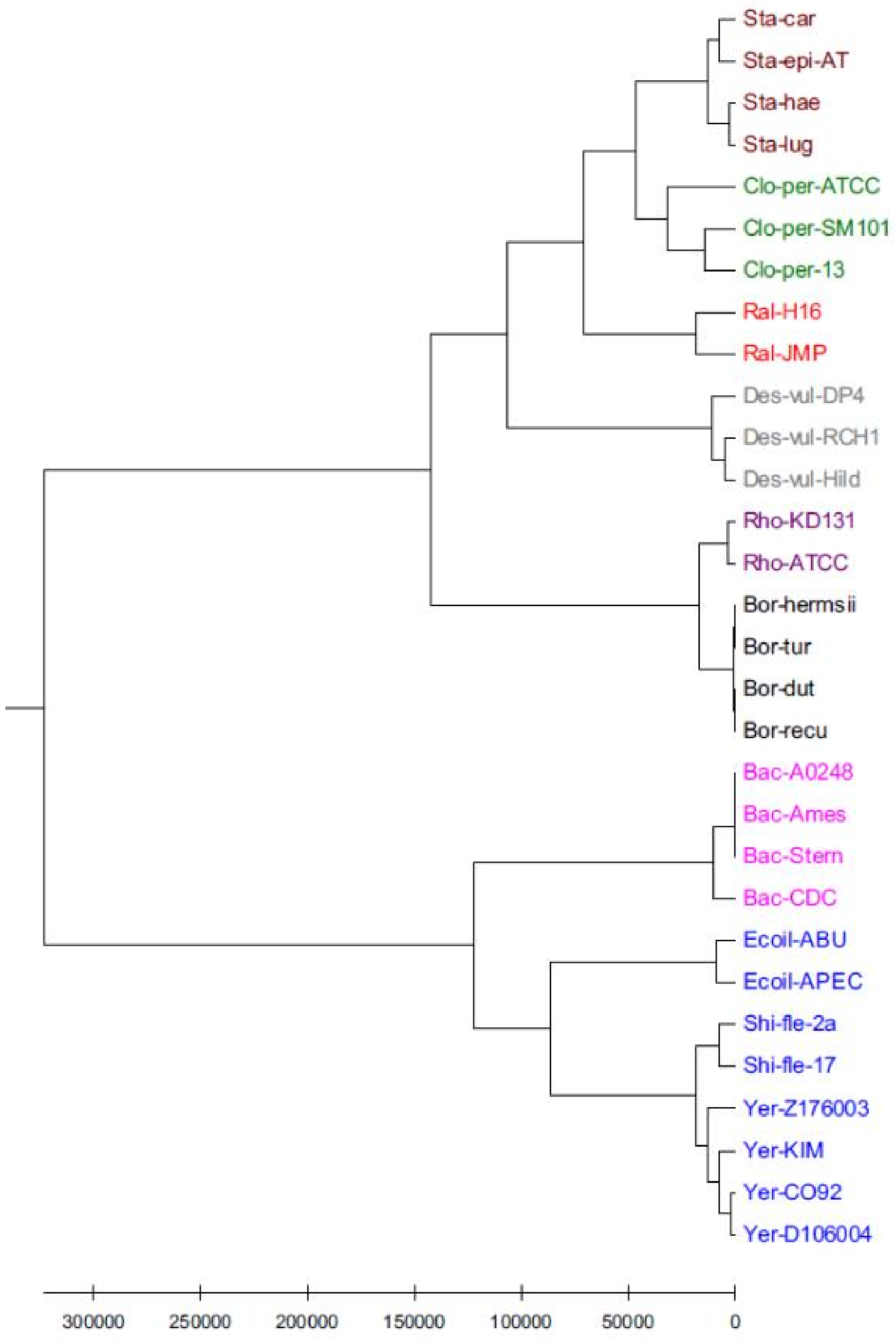

Figure S11: Phylogenetic tree of 30 bacteria whole genomes using the PS method[c].

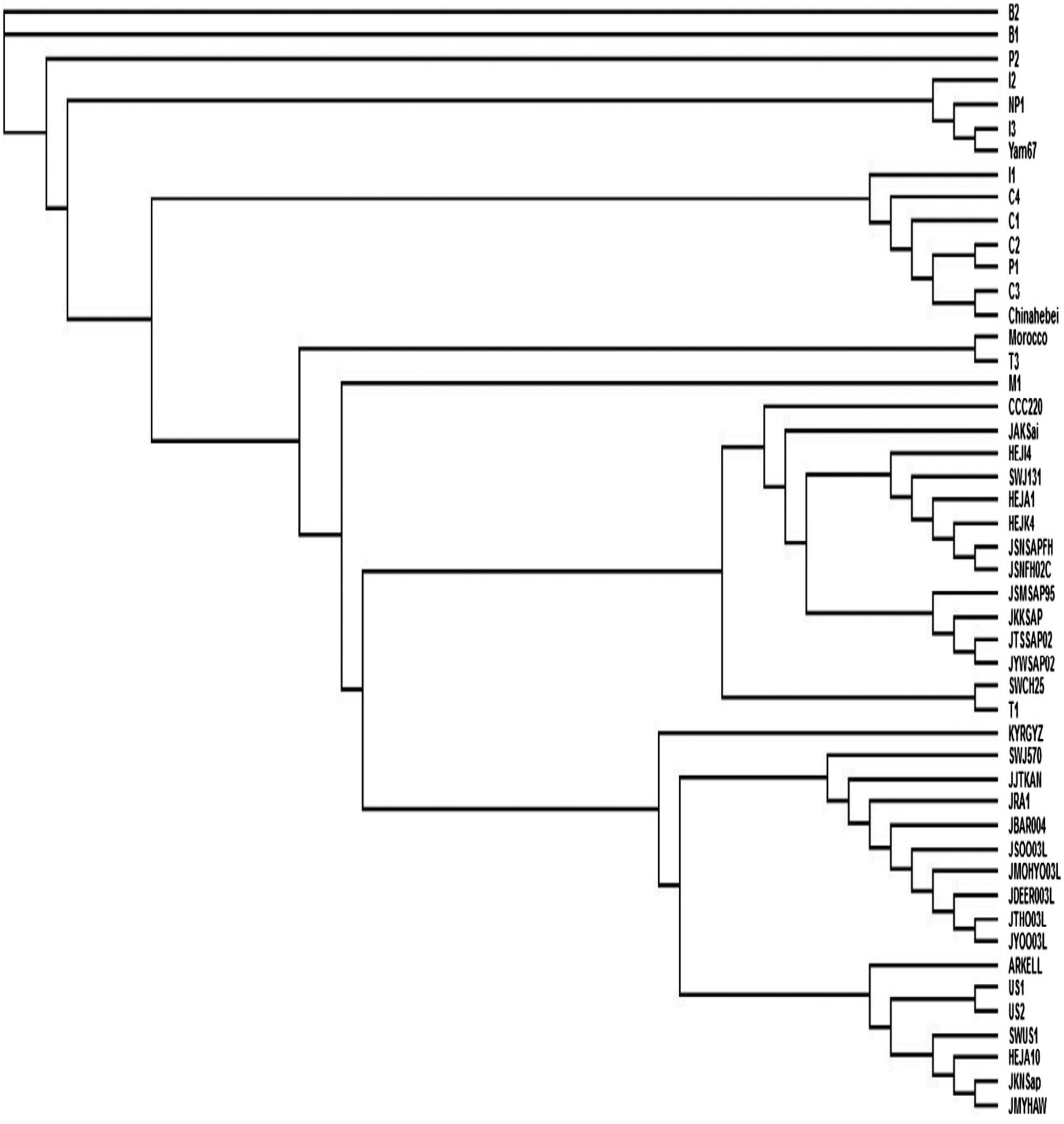

Figure S12: Phylogenetic tree obtained by the weighted measure using 48 HEV sequences[d].

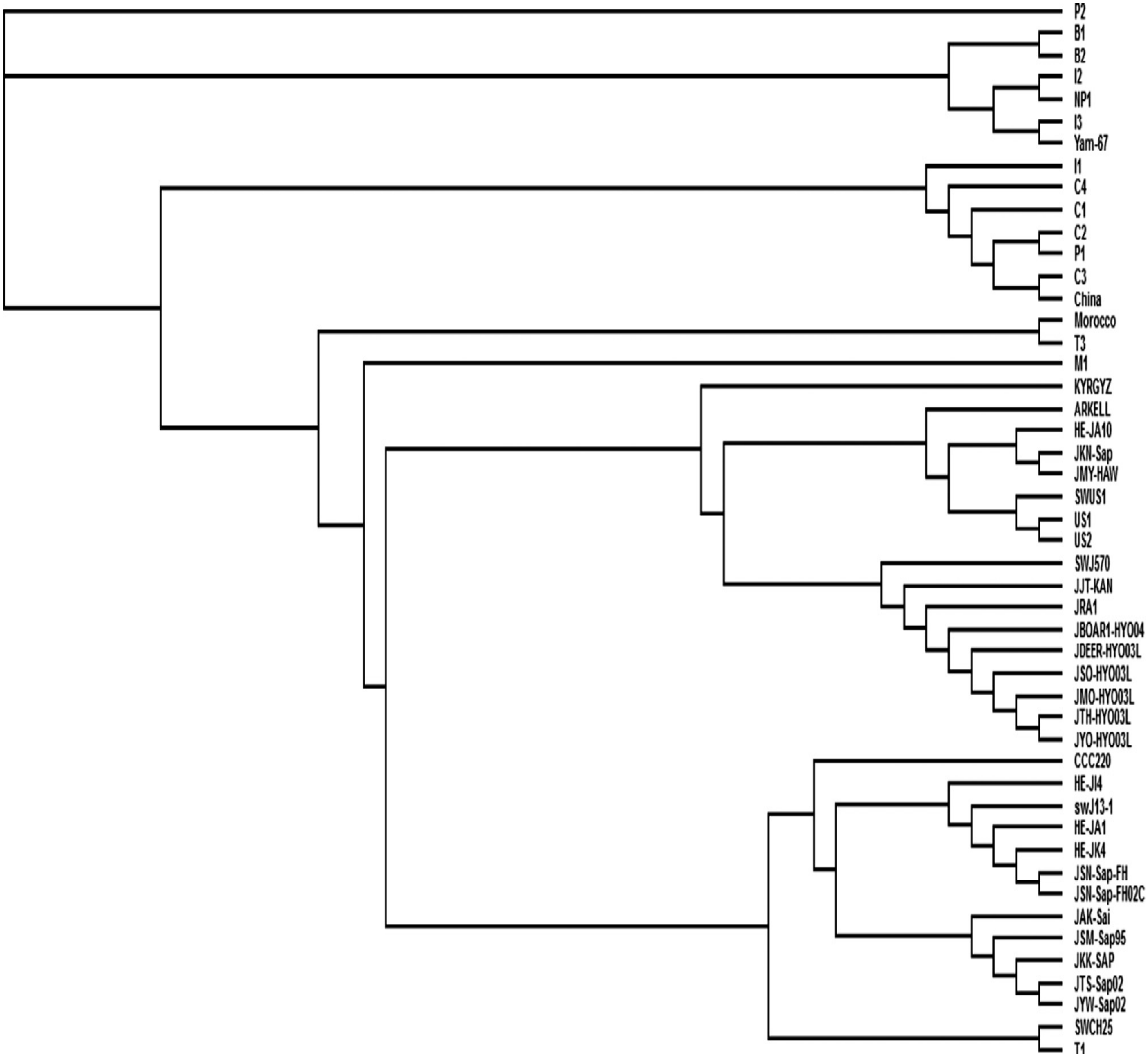

Figure S13: Phylogenetic tree obtained by the ClustalW using 48 HEV sequences[d].

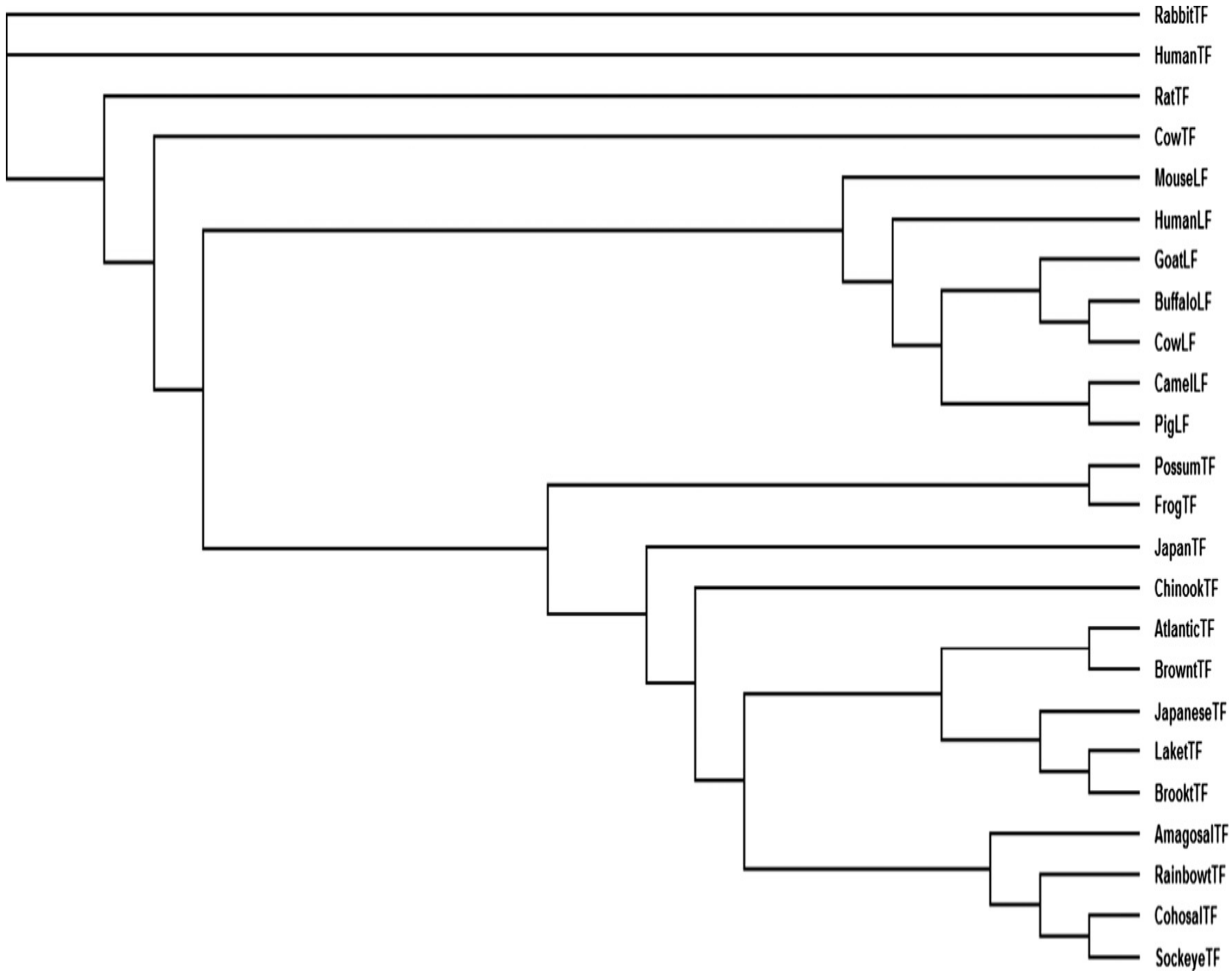

Figure S14: Phylogenetic tree obtained by the weighted measure using 24 Eutherian mammal sequences[d].

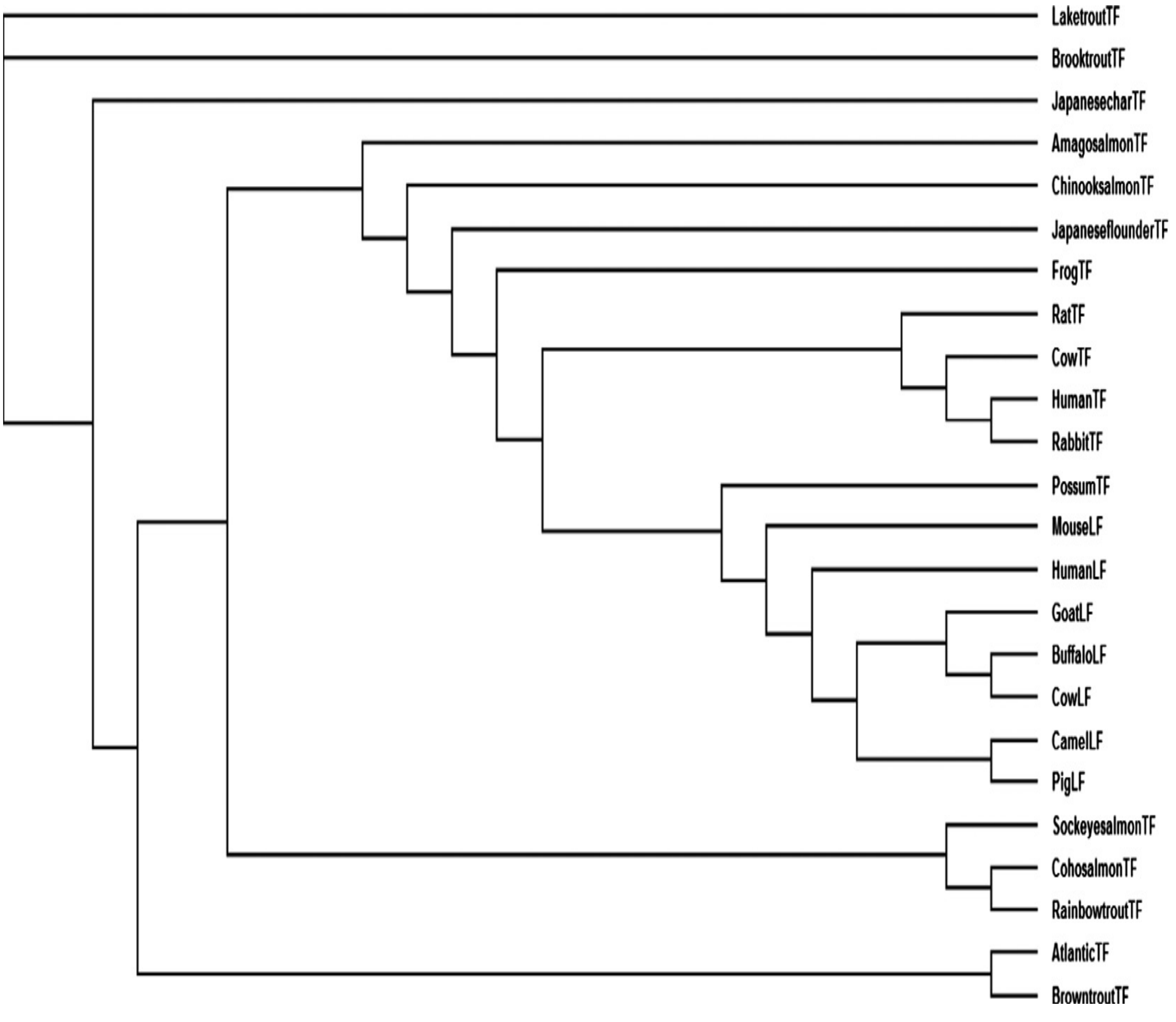

Figure S15: Phylogenetic tree obtained by ClustalW using 24 Eutherian mammal sequences[d].

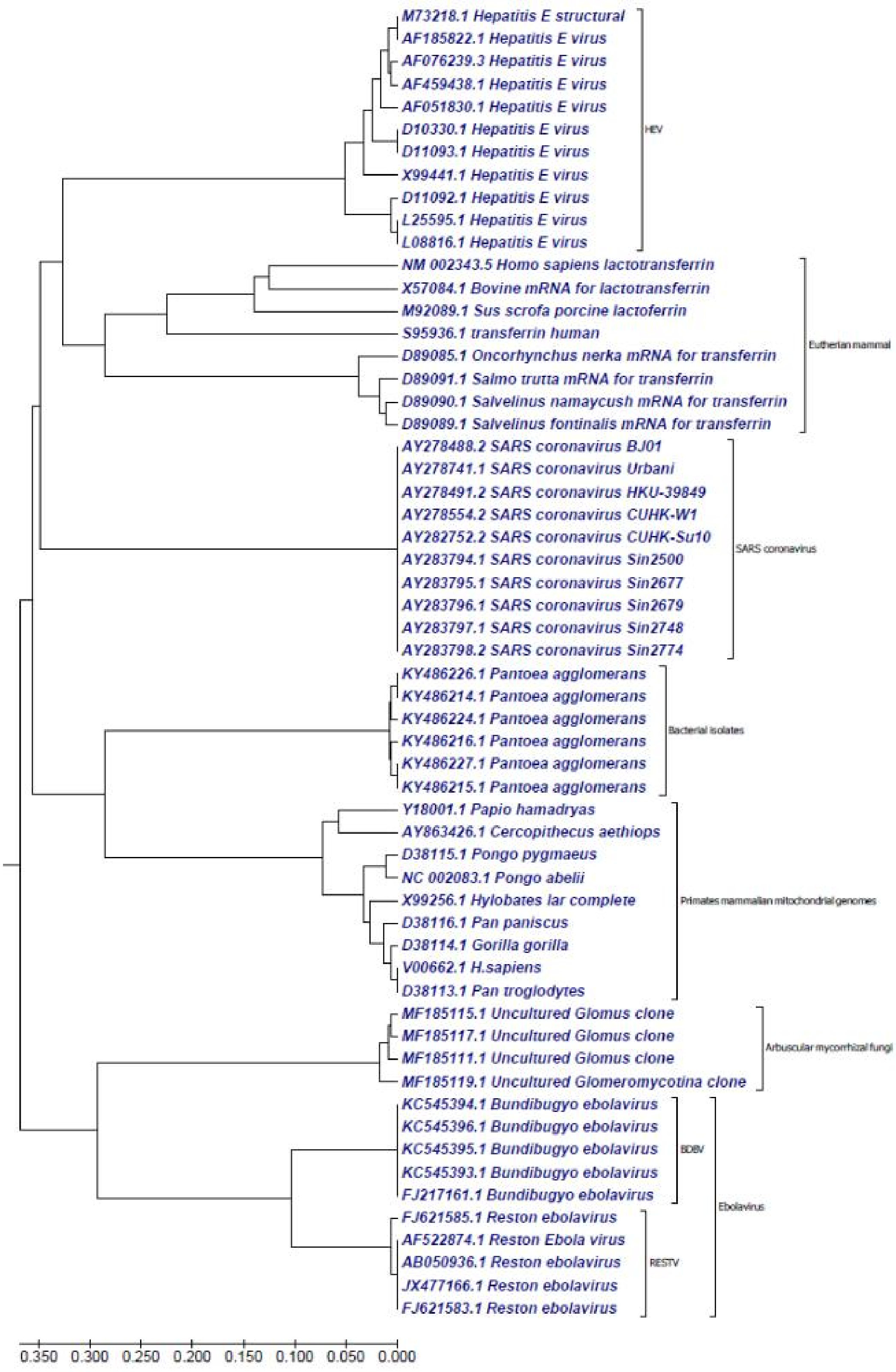

Figure S16: Phylogenetic tree obtained by ClustalW method using 58 genome datasets from different species.

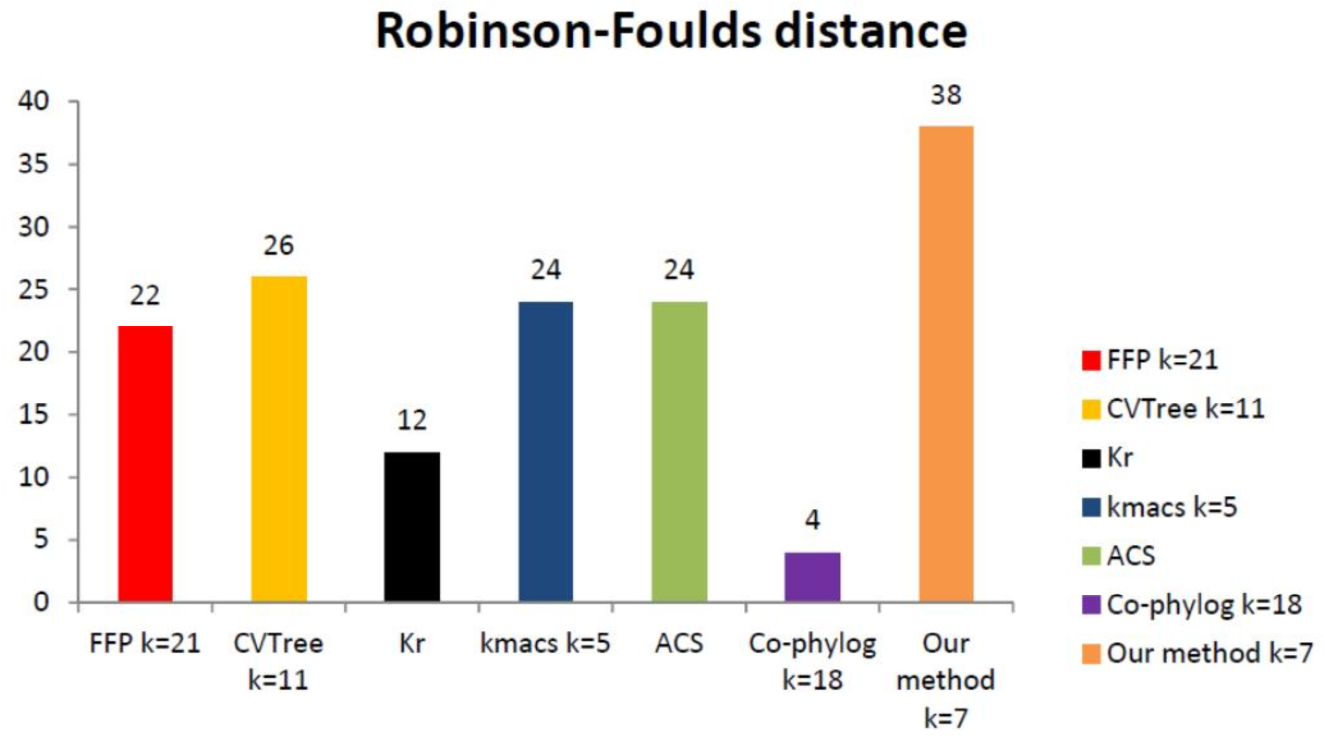

Figure S17: Robinson-Foulds distance of the benchmark tree against the trees constructed by our method, FFP k=21, CVTree k=11, Kr, kmacs k=5, ACS, Co-phylog k=18 using the Escherichia/Shigella 29 complete genomes[g].

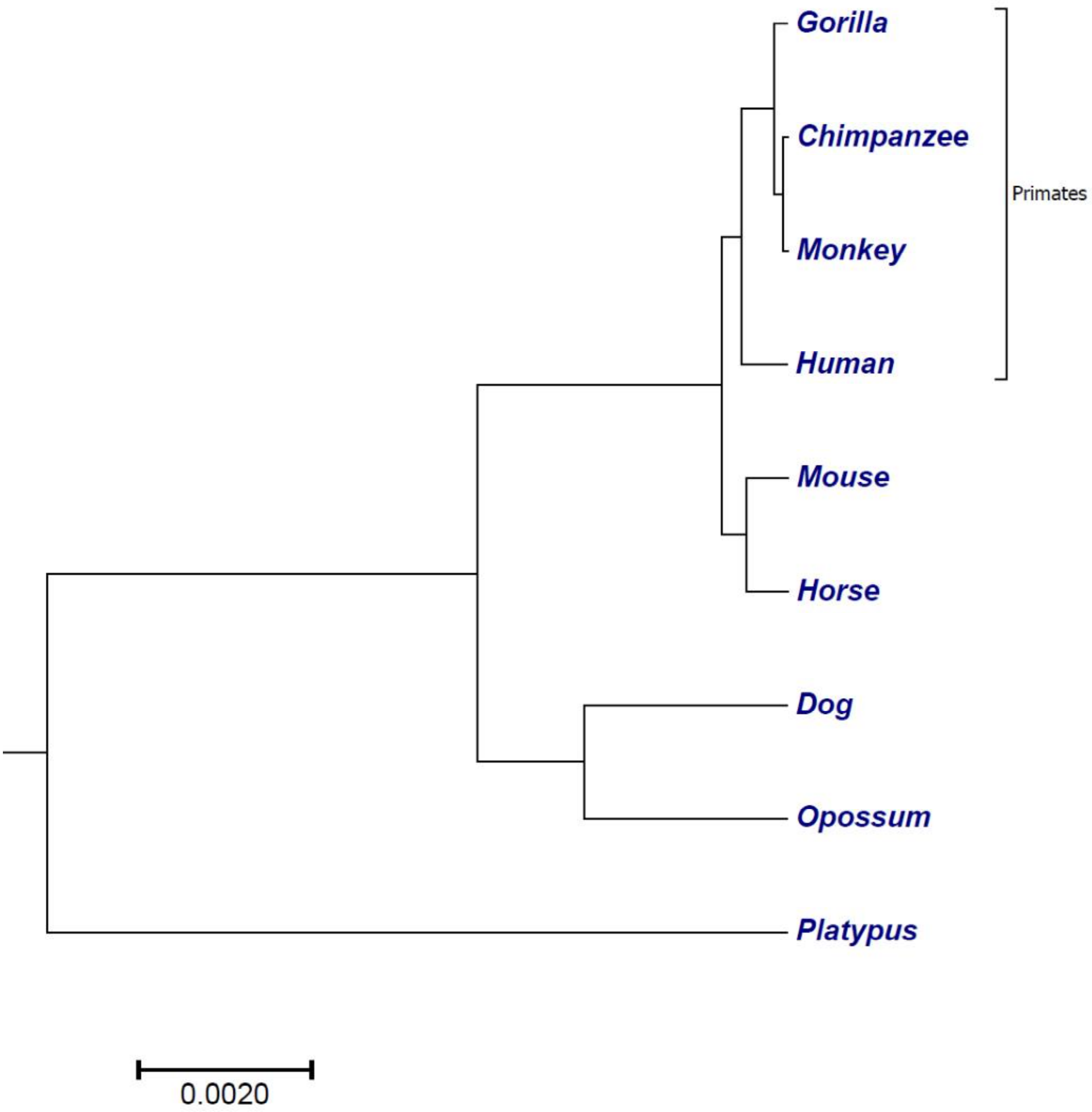

Figure S18: The phylogenetic tree of 9 mammals constructed by our method using PHYLIP package.

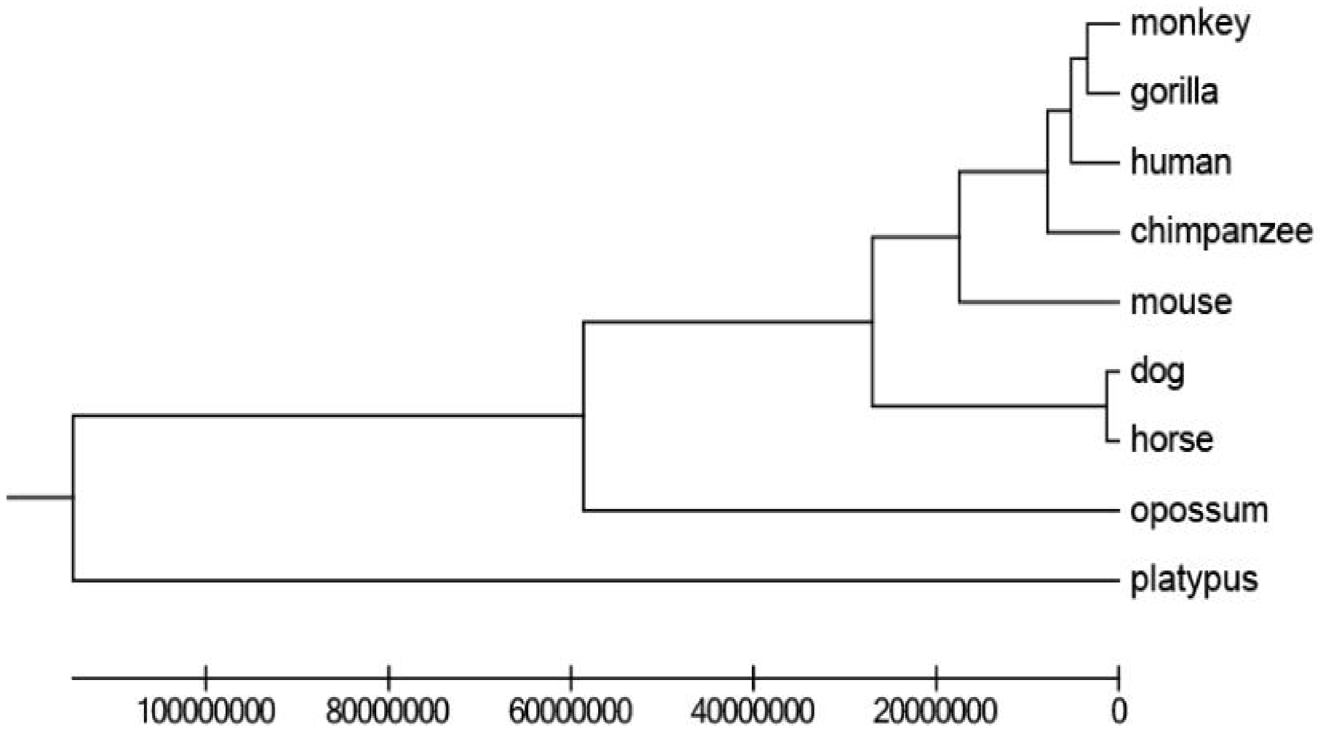

Figure S19: The phylogenetic tree of 9 mammals constructed using multiple encoding vector method[b].

REFERENCE:-

[a]:- Kumar, S., Stecher, G. & Tamura, K. Mega7: Molecular evolutionary genetics analysis version 7.0 for bigger datasets. Mol.Biol. Evol. 33, 1870–1874 (2016).

[b]:- Li, Y., He, L., He, R. L. & Yau, S. S.-T. A novel fast vector method for genetic sequence comparison. Sci. Reports 7 (2017).

[c]:- Hoang, T. et al. A new method to cluster dna sequences using fourier power spectrum. J. Theor. Biol. 372, 135 – 145 (2015).

[d]:- Liu, L., Li, C., Bai, F., Zhao, Q. & Wang, Y. An optimization approach and its application to compare dna sequences. J.Mol. Struct. 1082, 49 – 55 (2015).

[e]:- Yi, H. & Jin, L. Co-phylog: an assembly-free phylogenomic approach for closely related organisms. Nucleic Acids Res. 41, e75 (2013).

[f]:- Leimeister, C.-A., Sohrabi-Jahromi, S. & Morgenstern, B. Fast and accurate phylogeny reconstruction using filtered spaced-word matches. Bioinforma. 33, 971–979 (2017).

[g]:- Morgenstern, B., Zhu, B., Horwege, S. & Leimeister, C. A. Estimating evolutionary distances between genomic sequences from spaced-word matches. Algorithms for Mol. Biol. 10, 5 (2015).
