# Supplementary material for: Alignment-free method for DNA sequence clustering using Fuzzy integral similarity
Source: Sci Rep. 2019 Mar 6;9:3753. doi: 10.1038/s41598-019-40452-6 (PMC6403383; doi:10.1038/s41598-019-40452-6)
Supplement: Supplementary file 3 — Dataset 3 [file 41598_2019_40452_MOESM3_ESM.pdf]

Alignment-free method for DNA sequence clustering using Fuzzy integral similarity

Ajay Kumar Saw[1], Garima Raj[2], Manashi Das[3], Narayan C. Talukdar[4], Binod Chandra Tripathy[5] and Soumyadeep Nandi[6,*]

[1] Mathematical Sciences Division, Institute of Advanced Study in Science and Technology, Guwahati-35, India.

[2,3,4,6] Life Science Division, Institute of Advanced Study in Science and Technology, Guwahati-35, India.

[5] Mathematics Department, Tripura University, Tripura, India.

* To whom correspondence should be addressed. Email: soumyadeep.nandi@gmail.com; snandi@iasst.gov.in

ROC_supplementary material

Explanation:

The objective of the study is to develop an Alignment-free tool for sequence clustering. To illustrate the effectiveness of our method we compared the distance matrices and the phylogenetic trees generated by our method with the other freely available recent alignment-free tools [1]. The clustering efficiency of our tool can be noticed across all the benchmark datasets. The phylogenetic trees generated by our method and the other freely available recent methods shows superiority of our method over the other methods in terms of sequence clustering. The consistency can also be seen from the statistical analysis such as AUC (area under the ROC) values calculated from ROC (Receiver operating characteristic) curves. AUC values are often used to explain the accuracy of models and not to compare them. Accuracy classification of AUC is summarised in Table 1, which is given below.

**AUC Range**	**Classification**
AUC $\geq$ 0.9	high accuracy
0.7 $\leq$ AUC< 0.9	moderate accuracy
0.5 $\leq$ AUC< 0.7	Low accuracy

Table 1: Accuracy classification of AUC [2, 3, 4, 5, 6].

A low AUC value does not necessarily imply a bad or poor model; rather it simply suggests that, besides the accounted predictors, other factors also exercise influence on the response

variable. It is evident from the ROC plots below that our method's performance is in good agreement with other methods.

ROC calculation:-

The phylogenetic tree is generated by a distance matrix using PHYLIP package [7] which is equal to (1-similarity matrix) from equation (12) in our manuscript. We used similarity matrix for ROC analysis. We follow sonego et al.'s method [8] for calculating the ROC. In the similarity matrix, we put positive sign, if the two sequences belong to same class (i.e., genus level classification, phylum level classification, family level classification, etc.,) otherwise we put negative sign. We plotted the ROC curve by changing the decision threshold in either strictly increasing or strictly decreasing pattern lies between the minimum and maximum values of the similarity matrix. We plotted the FPR (false positive rate) on X-axis and TPR (true positive rate) on Y-axis. Every point in the ROC curve corresponds to a discrete classifier that can be calculated using a decision threshold (Figure 3 in [8]). After plotting ROC curve, we calculated the area under the ROC curve (AUC).

Phylogenetic tree on 11 AMF sequences.

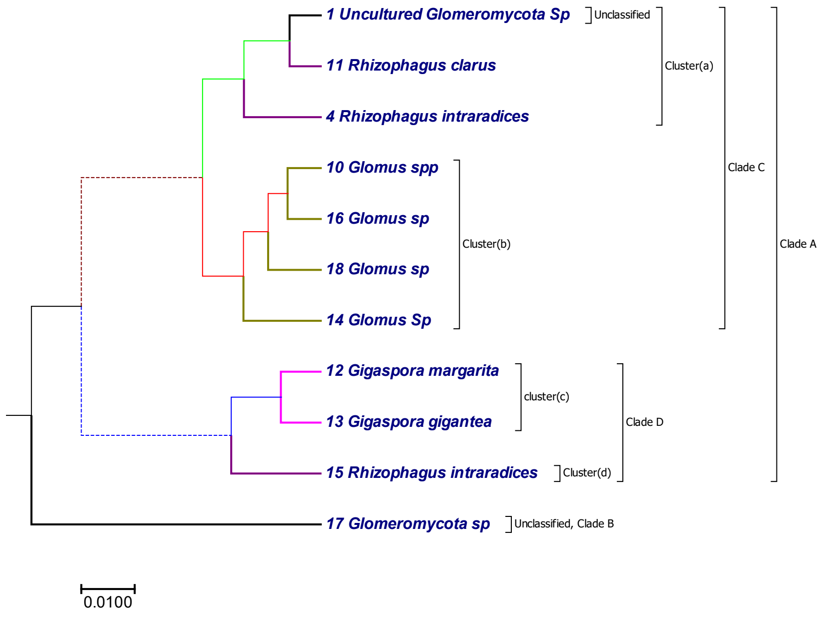

Figure 1: Our method.

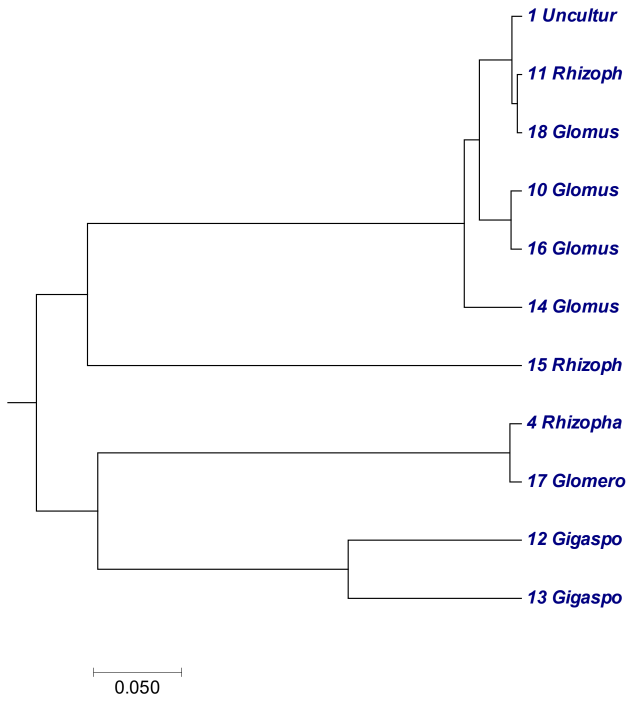

Figure 2 : CV method using string length 3.

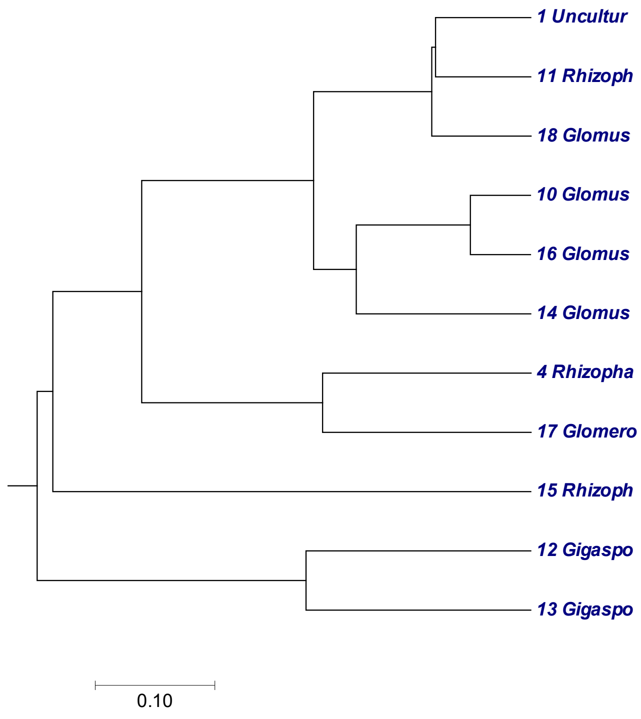

Figure 3: FFP method using string length 7.

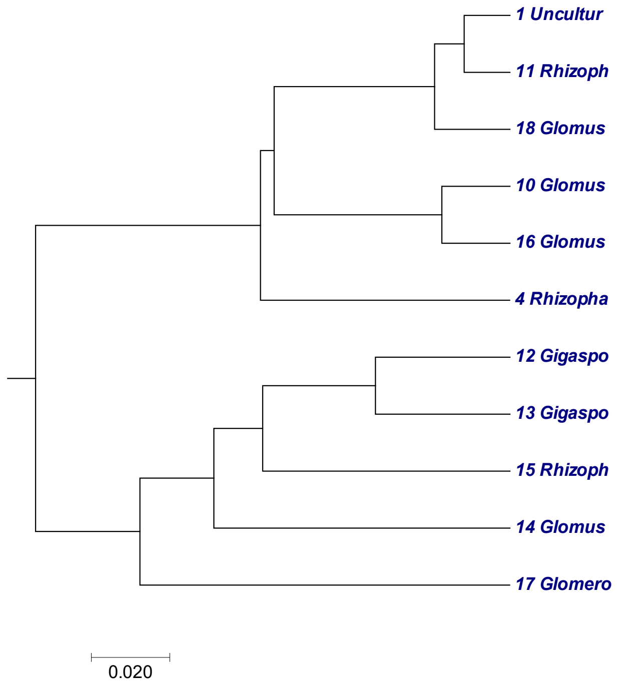

Figure 4: RTD method using string length 1.

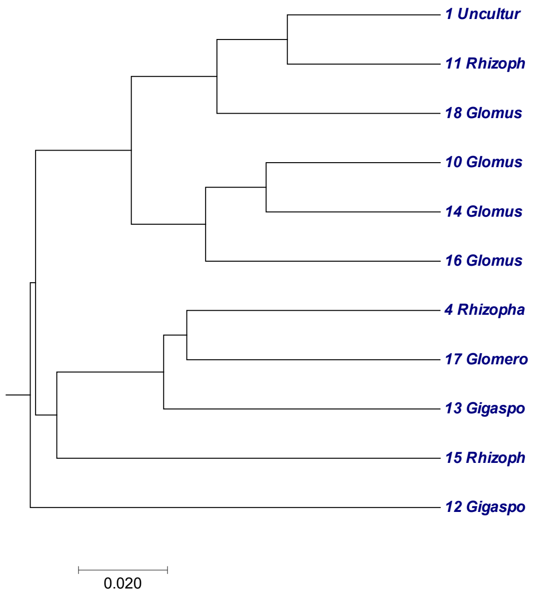

Figure 5: BBC method.

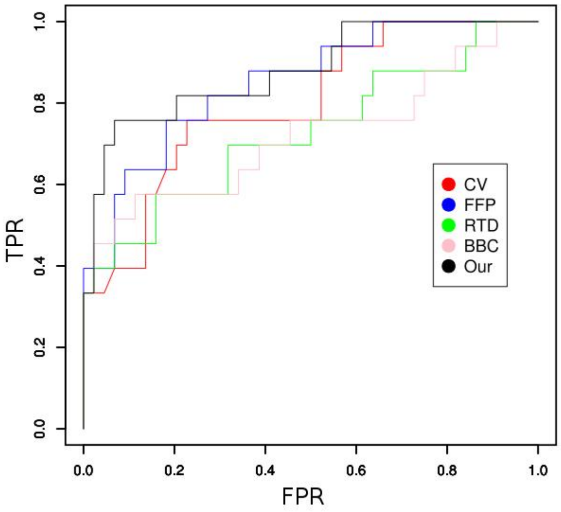

Dataset→ 11 AMF		
**Methods↓**	**AUC↓**	**Running time↓**
CV method	0.796143	<1s
FFP method	0.85124	<1s
RTD method	0.727273	<1s
BBC method	0.720386	<1s
Our method	0.880165	<1s

Figure 6: Receiver operating characteristic curve (ROC) and Area under the ROC Curve on 11 AMF sequences.

The phylogenetic tree (Figure 1) generated by our method successfully clustered all glomus genera (cluster (b)) belonging to family glomeraceae, all gigaspora genera (cluster (c)) belonging to family gigasporaceae and all rhizophagus genera (cluster (a)) belonging to family glomeraceae except 15 Rhizophagus intraradice (cluster (d)) in separate clades. While comparing the tree prepared by our method (Figure 1) with the tree prepared by other freely available tools [1] (Figures 2, 3, 4 and 5). We found that, all glomus genera were not properly clustered together in Figures 2, 3, 4 and 5. Similarly, all gigaspora genera were not clustered together in Figure 5. This shows the advantage of our method over others in terms of sequence clustering. Moreover, the AUC of our method is 0.88 (Figure 6), which indicates that our method has moderate accuracy (Table 1).

Phylogenetic tree on 41 mammalian sequences.

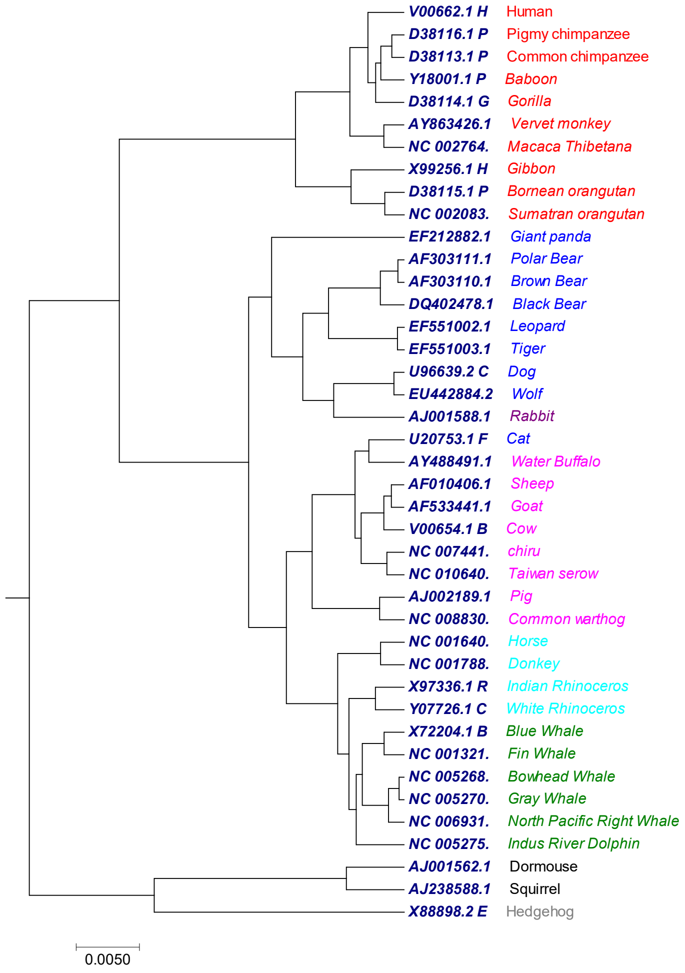

Figure 7: Our method.

(Primates (red), Cetacea (green), Artiodactyla (pink), Perissodactyla (light green), Rodentia (black), Lagomorpha (dark red), Carnivore (blue), and Erinaceomorpha (grey)).

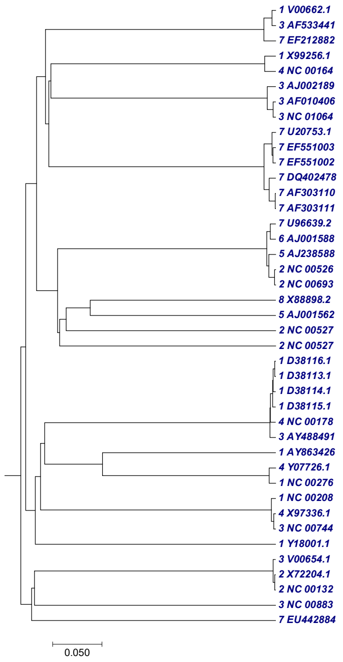

Figure 8: CV method using string length 3.

(1 → Primates, 2 → Cetacea, 3 → Artiodactyla, 4 → Perissodactyla, 5 → Rodentia, 6 → Lagomorpha, 7 → Carnivore, and 8 → Erinaceomorpha).

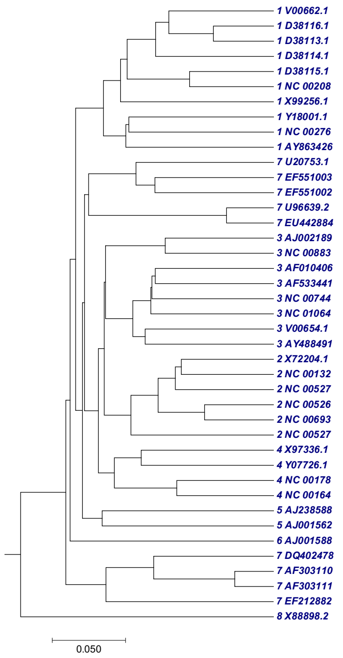

Figure 9: FFP method using string length 7.

(1 → Primates, 2 → Cetacea, 3 → Artiodactyla, 4 → Perissodactyla, 5 → Rodentia, 6 → Lagomorpha, 7 → Carnivore, and 8 → Erinaceomorpha).

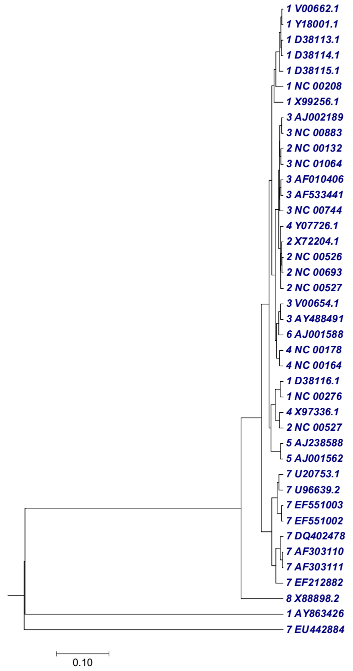

Figure 10: RTD method using string length 1.

(1 → Primates, 2 → Cetacea, 3 → Artiodactyla, 4 → Perissodactyla, 5 → Rodentia, 6 → Lagomorpha, 7 → Carnivore, and 8 → Erinaceomorpha).

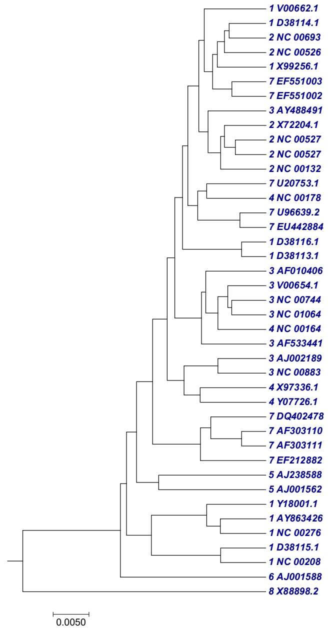

Figure 11: BBC method.

1→ Primates, 2 → Cetacea, 3 → Artiodactyla, 4 → Perissodactyla, 5 → Rodentia, 6 → Lagomorpha, 7 → Carnivore, and 8 → Erinaceomorpha).

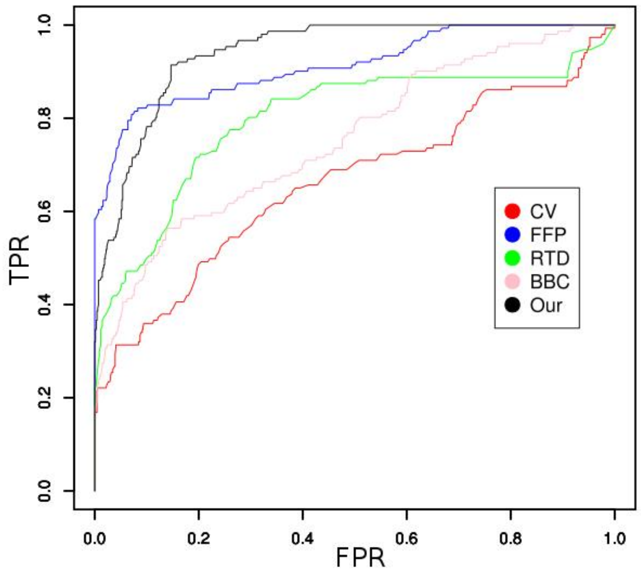

Dataset→ 41 mammalian		
**Methods↓**	**AUC↓**	**Running time ↓**
CV method	0.65962	<1s
FFP method	0.912888	1s
RTD method	0.795208	<1s
BBC method	0.753803	5s
Our method	0.940862	<1s

Figure 12: Receiver operating characteristic curve (ROC) and Area under the ROC Curve on 41 mammalian sequences.

41 species in the phylogenetic tree (Figure 7) generated by our approach, were correctly divided into eight groups: Primates (red), Cetacea (green), Artiodactyla (pink), Perissodactyla (light green), Rodentia (black), Lagomorpha (dark red), Carnivore (blue), and Erinaceomorpha (grey). The cat species in our approach was clustered with the Artiodactyla group. We compared the phylogenetic tree (Figure 7) generated by our approach with the phylogenetic tree (Figures 8, 9, 10 and 11) prepared by other freely available tools [1]. In Figure 8, eight groups were not properly clustered. In Figures 10 and 11, Primates (1), Cetacea (2), Artiodactyla (3), Perissodactyla (4) were all divided into more than one group. Figure 9 has similarity with our result (Figure 7). This shows the advantage of our method over other methods (Figures 8, 10 and 11) in terms of sequence clustering. Moreover, the AUC of our method is 0.94 (Figure 12), which indicates that our method has high accuracy (Table 1).

Phylogenetic tree on 59 ebolavirus sequences.

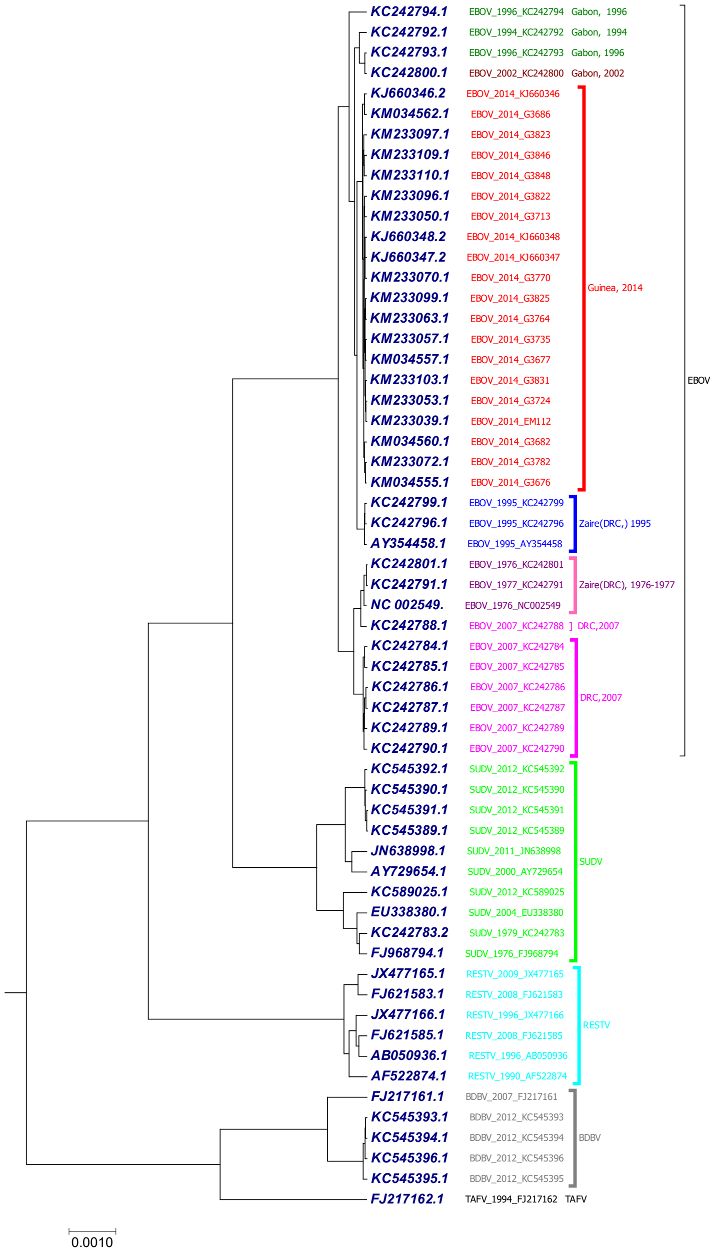

Figure 13: Our method.

(Bundibugyo virus (BDBV), Reston virus (RESTV), Ebola virus (EBOV), Sudan virus (SUDV), and Tai Forest virus (TAFV)).

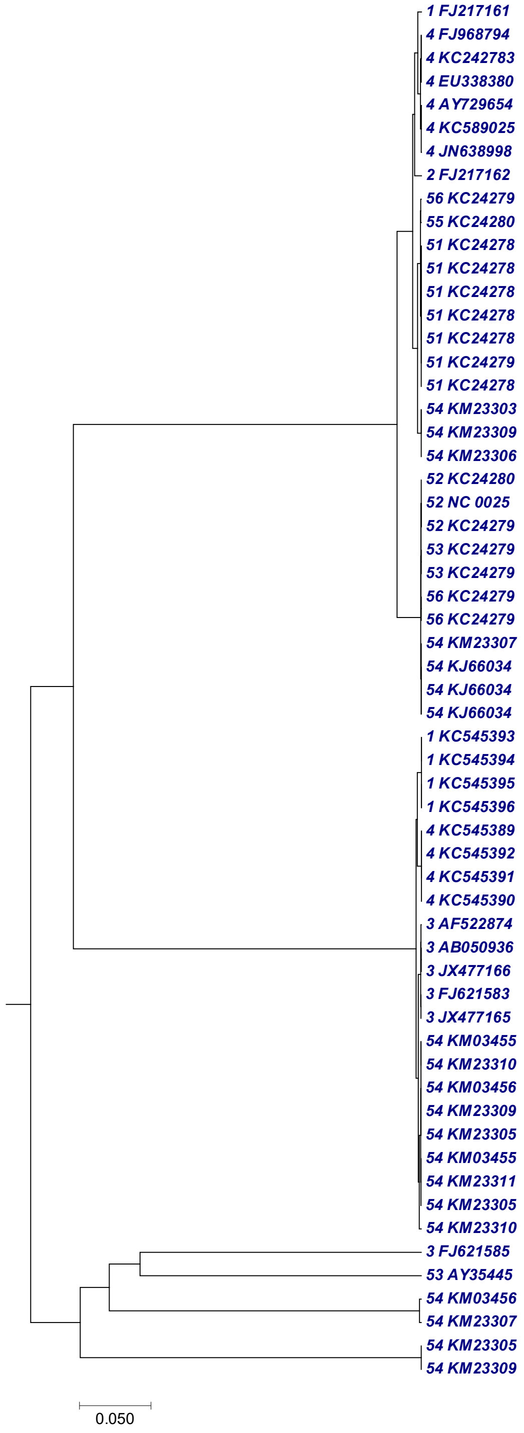

Figure 14: CV method using string length 3.

(1 →Bundibugyo virus (BDBV), 2 →Tai Forest virus (TAFV)), 3 →Reston virus (RESTV), 4 →Sudan virus (SUDV), 51,51,53,54,55 and 56 →Ebola virus (EBOV)).

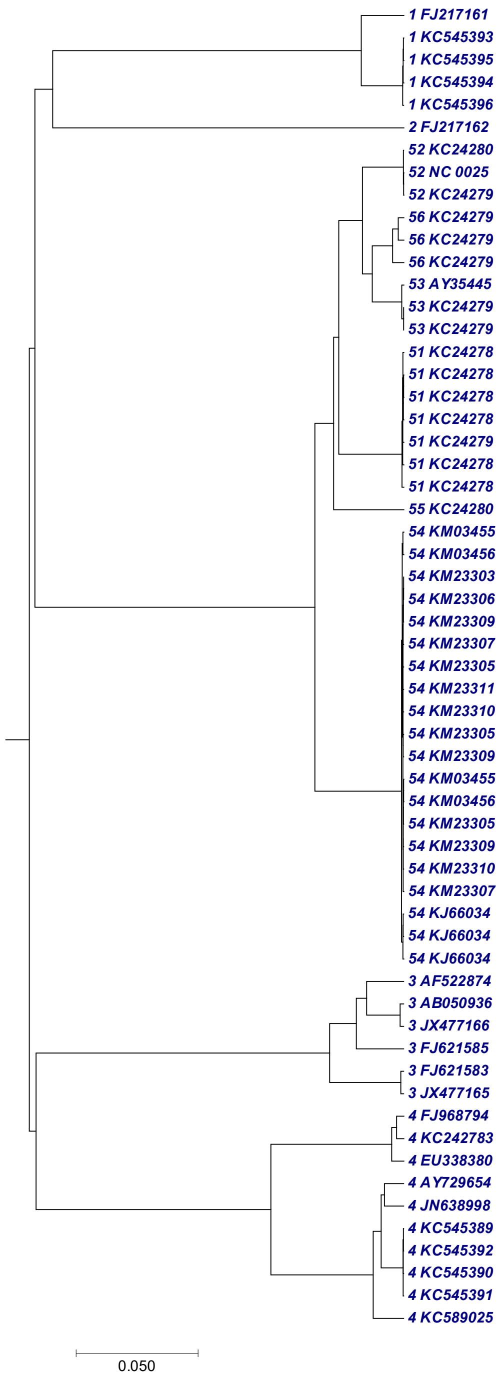

Figure 15: FFP method using string length 7.

(1 →Bundibugyo virus (BDBV), 2 →Tai Forest virus (TAFV)), 3 →Reston virus (RESTV), 4 →Sudan virus (SUDV), 51,51,53,54,55 and 56 →Ebola virus (EBOV)).

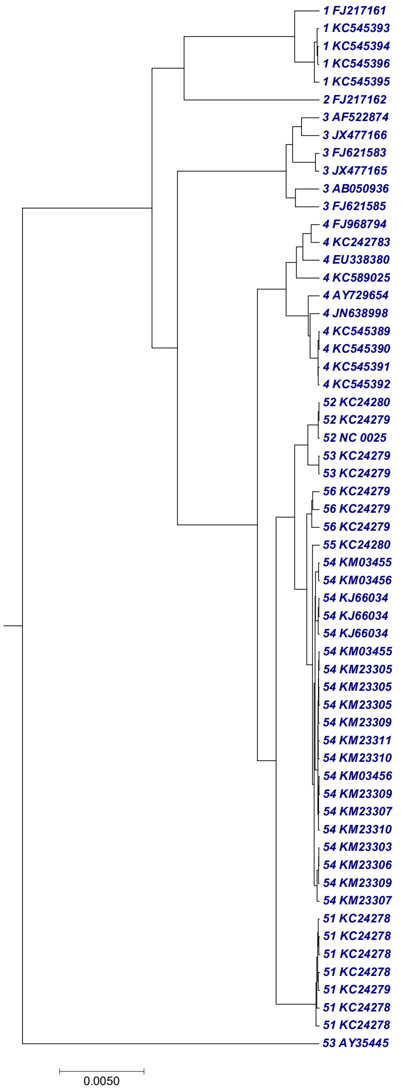

Figure 16: RTD method using string length 1.

(1 →Bundibugyo virus (BDBV), 2 →Tai Forest virus (TAFV)), 3 →Reston virus (RESTV), 4 →Sudan virus (SUDV), 51,51,53,54,55 and 56 →Ebola virus (EBOV)).

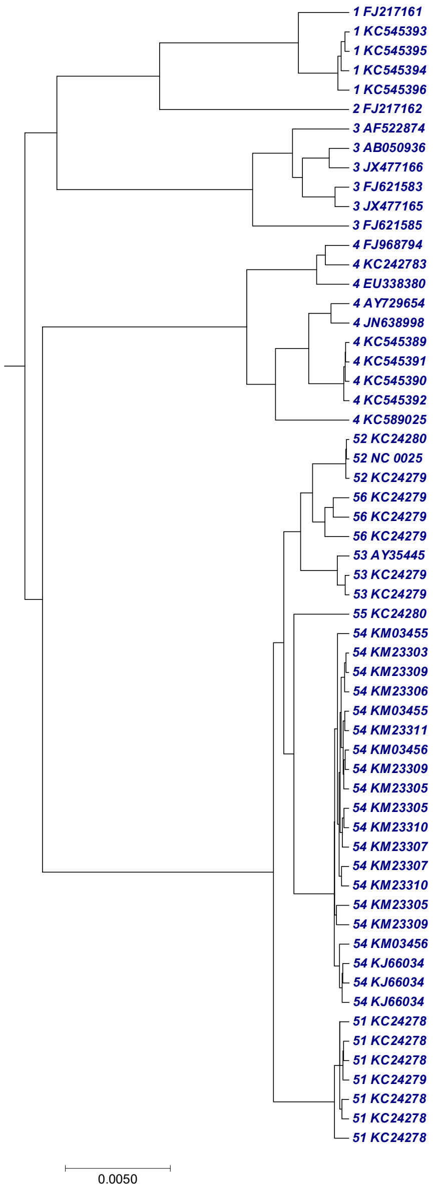

Figure 17: BBC method.

(1 →Bundibugyo virus (BDBV), 2 →Tai Forest virus (TAFV)), 3 →Reston virus (RESTV), 4 →Sudan virus (SUDV), 51,51,53,54,55 and 56 →Ebola virus (EBOV)).

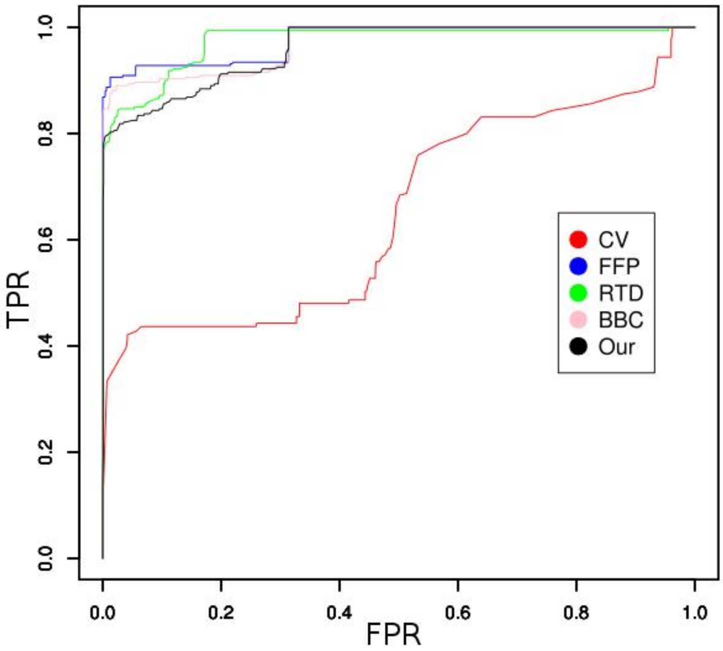

Dataset→ 59 ebolavirus		
**Methods↓**	**AUC↓**	**Running time↓**
CV method	0.644968	<1s
FFP method	0.976518	1s
RTD method	0.973445	<1s
BBC method	0.969714	7s
Our method	0.959937	<1s

Figure 18: Receiver operating characteristic curve (ROC) and Area under the ROC Curve on 59 ebolavirus sequences.

The Ebolavirus genus includes five species: Bundibugyo virus (BDBV), Reston virus (RESTV), Ebola virus (EBOV), Sudan virus (SUDV), and Tai Forest virus (TAFV). As shown in Figure 13 generated by our method, the five species were correctly separated. We compared the phylogenetic tree (Figure 13) generated by our approach with the phylogenetic trees (Figures 14, 15, 16 and 17) prepared by other freely available tools [1]. In Figure 14, five species were not properly clustered. Similarly, our result (Figure 13) was in consensus with Figures 15, 16 and 17. Moreover, the AUC of our method is 0.96 (Figure 18), which indicates that our method has high accuracy (Table 1).

Phylogenetic tree on 30 coronavirus sequences.

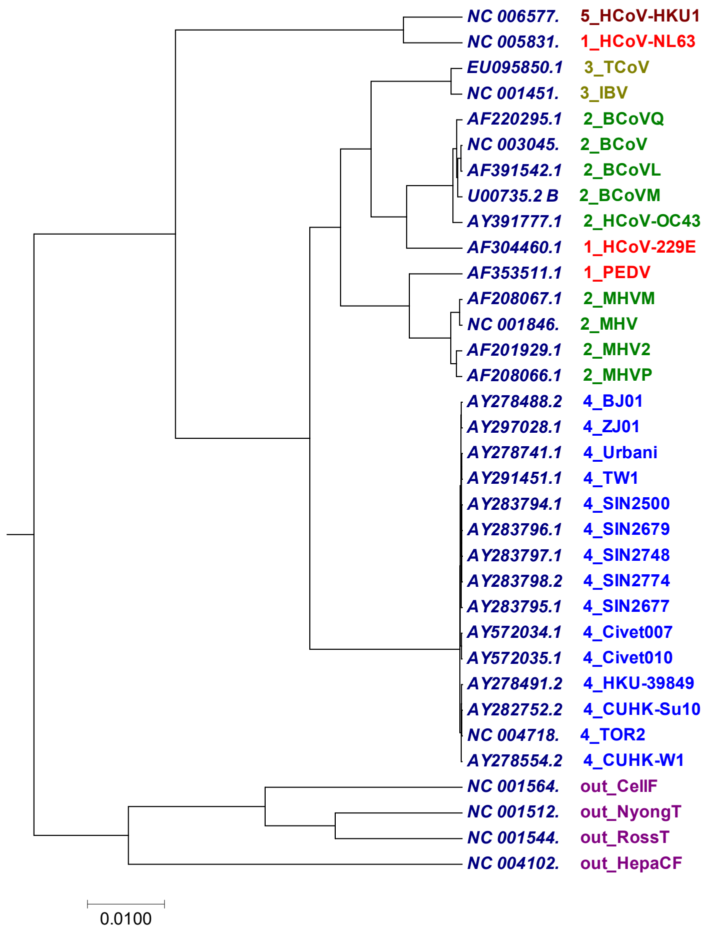

Figure 19: Our method.

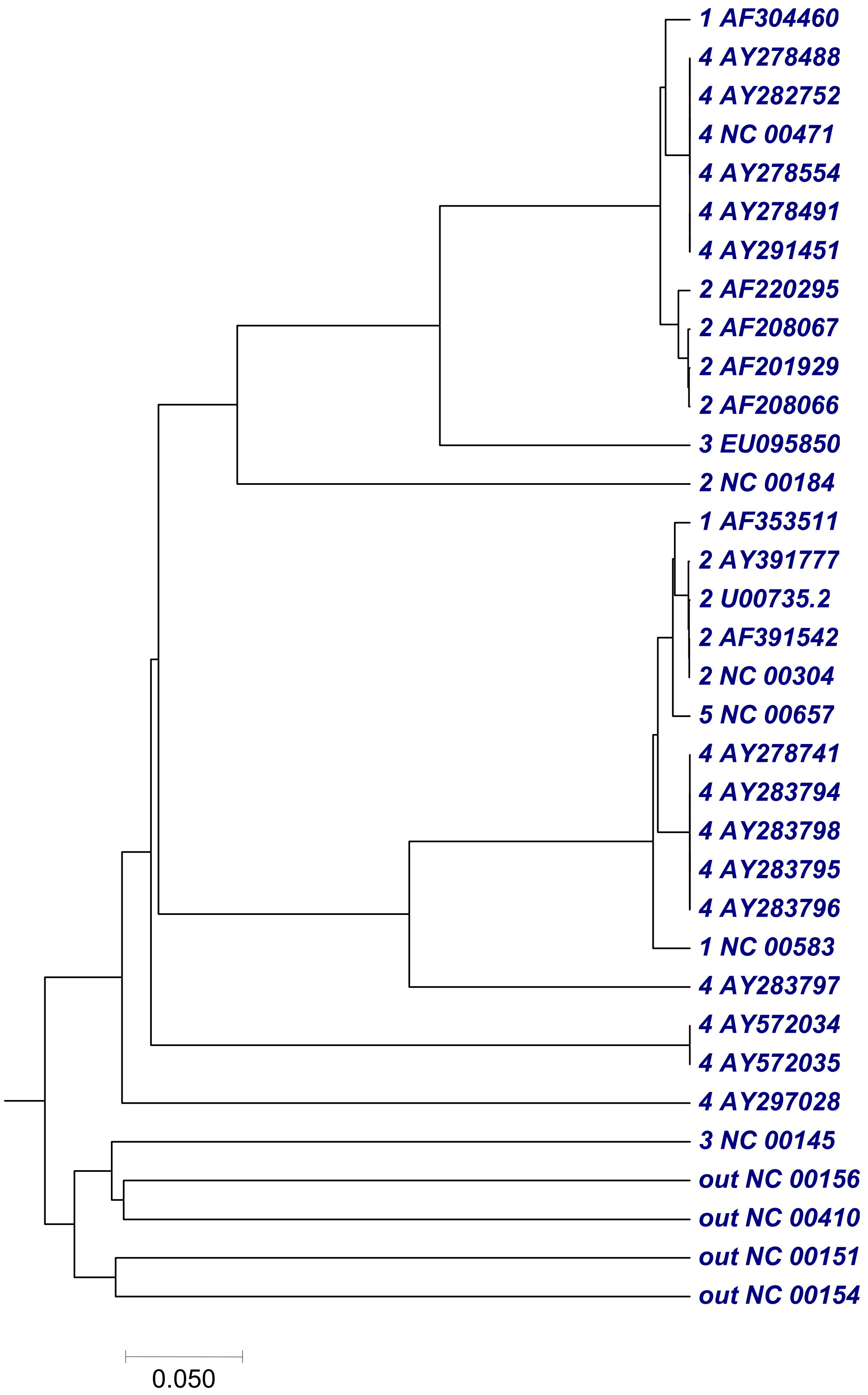

Figure 20: CV method using string length 3.

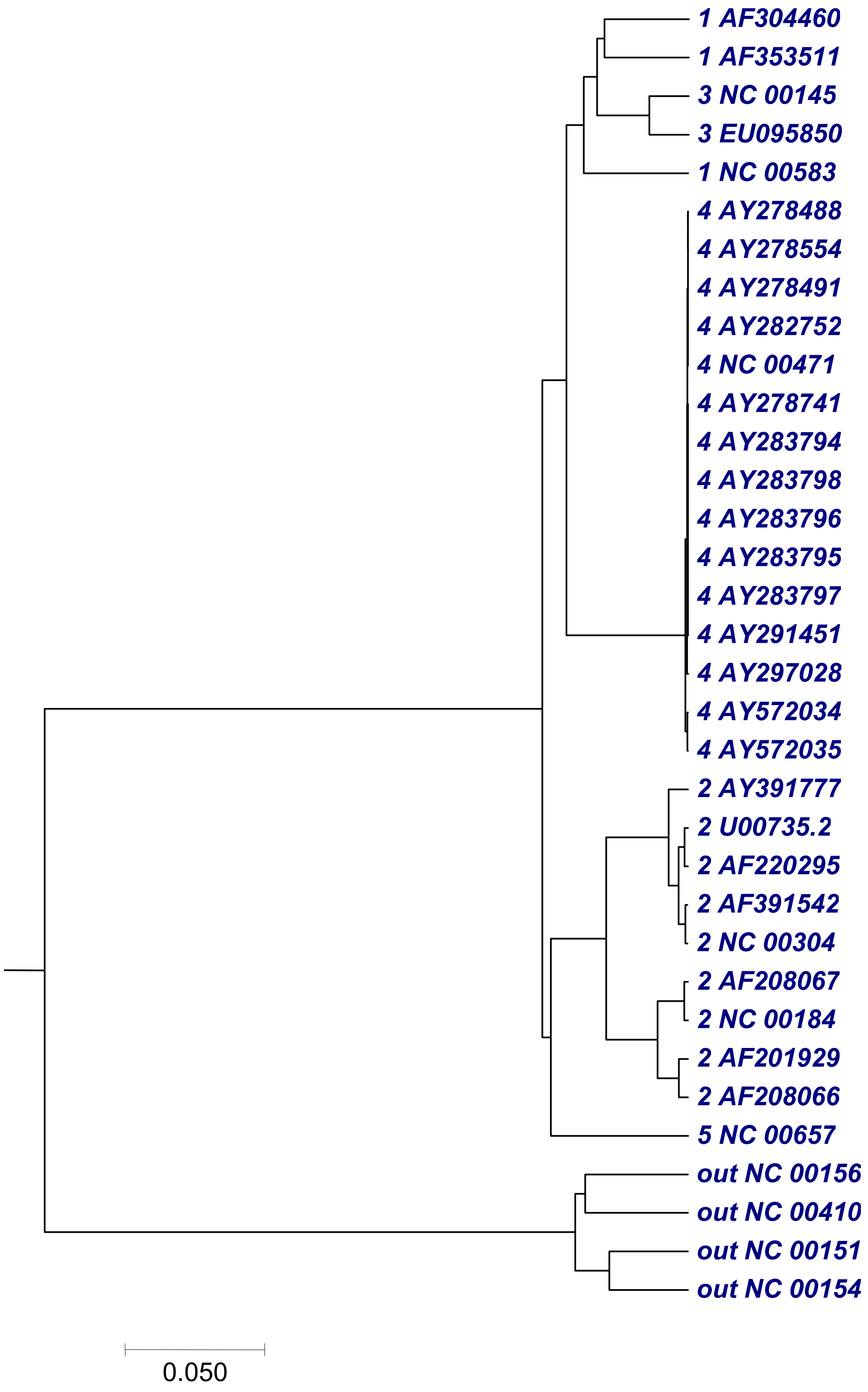

Figure 21: FFP method using string length 6.

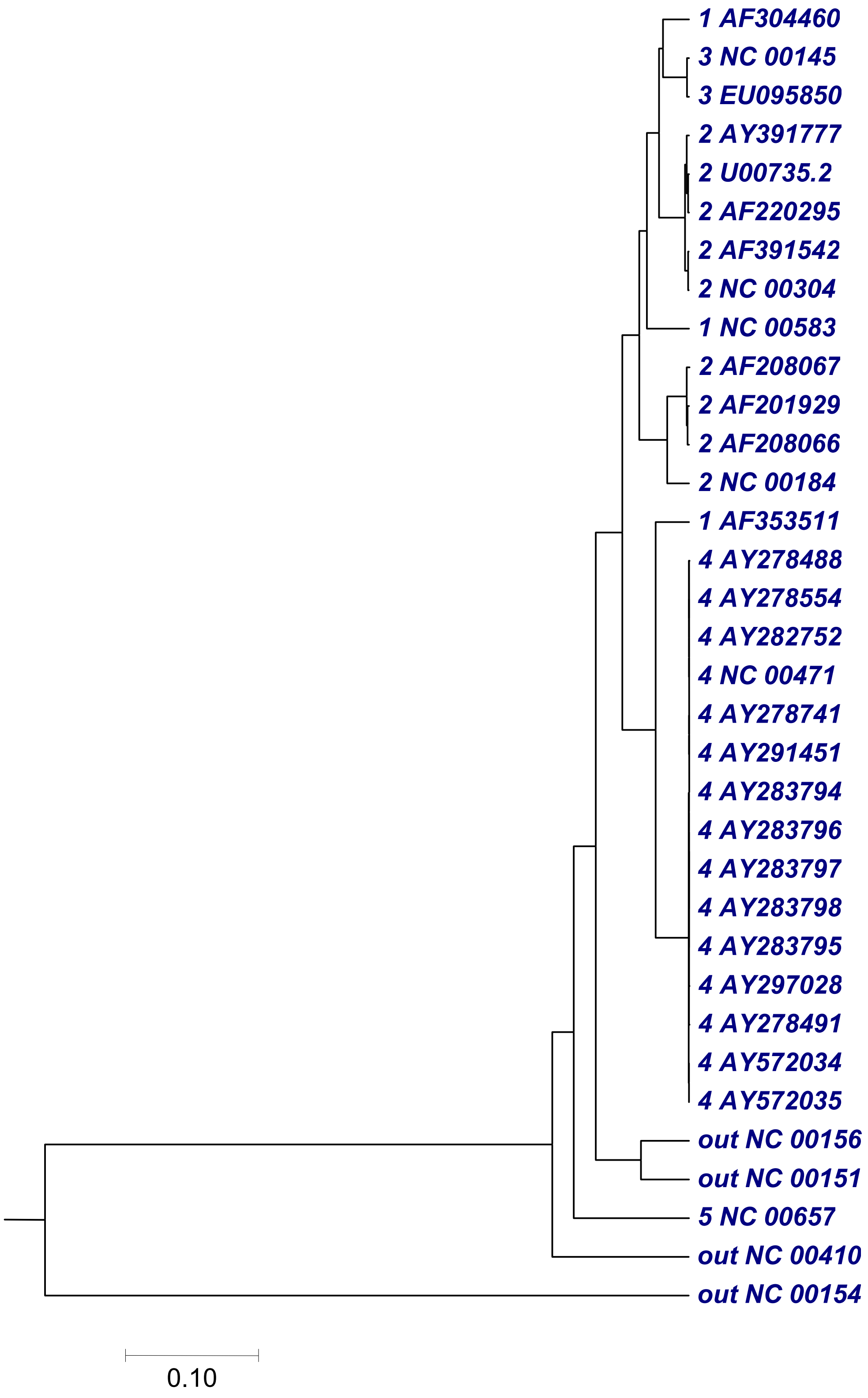

Figure 22: RTD method using string length 1.

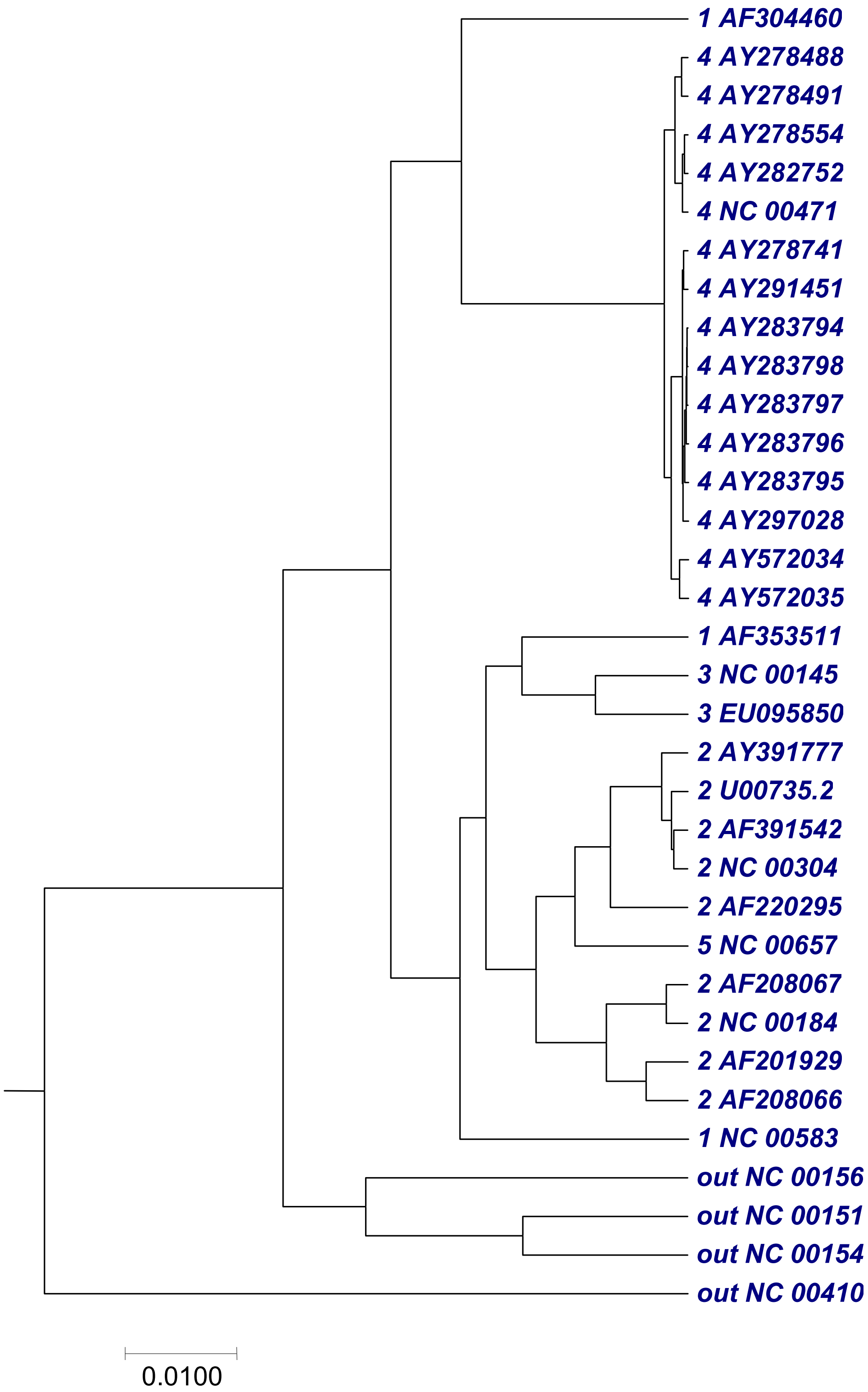

Figure 23: BBC method.

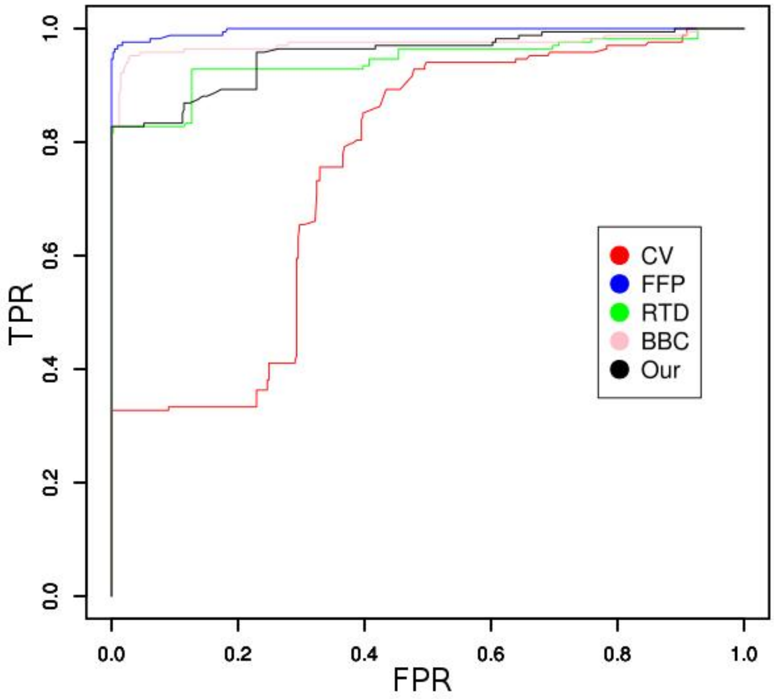

Dataset→ 30 coronavirus		
Methods↓	AUC↓	Running time↓
CV method	0.751089	1s
FFP method	0.996733	<1s
RTD method	0.942451	<1s
BBC method	0.973824	6s
Our method	0.952526	<1s

Figure 24: Receiver operating characteristic curve (ROC) and Area under the ROC Curve on 30 coronavirus sequences.

We employed our method to analyse the 30 coronavirus whole genome sequences along with 4 non-coronaviruses as outgroups. The 30 coronavirus were classified into five groups according to their host type. As shown in Figure 19 generated by our approach, we can observe that the 30 coronavirus along with 4 non-coronaviruses were correctly grouped according to their host type except group 1. We compared Figure 19 generated by our approach with Figures 20, 21, 22 and 23 prepared by other freely available tools [1]. Figure 20 did not cluster any group according to their host type. In Figures 22 and 23, the four non-coronaviruses were not clustered together. None of the methods (Figures 20, 21, 22 and 23) including our method (Figure 19) put group 1 sequences in separate clade. This shows the efficiency of our method in terms of sequence clustering. Moreover, the AUC of our method is 0.95 (Figure 24), which indicates that our method has high accuracy (Table 1).

Phylogenetic tree on 30 bacterial sequences.

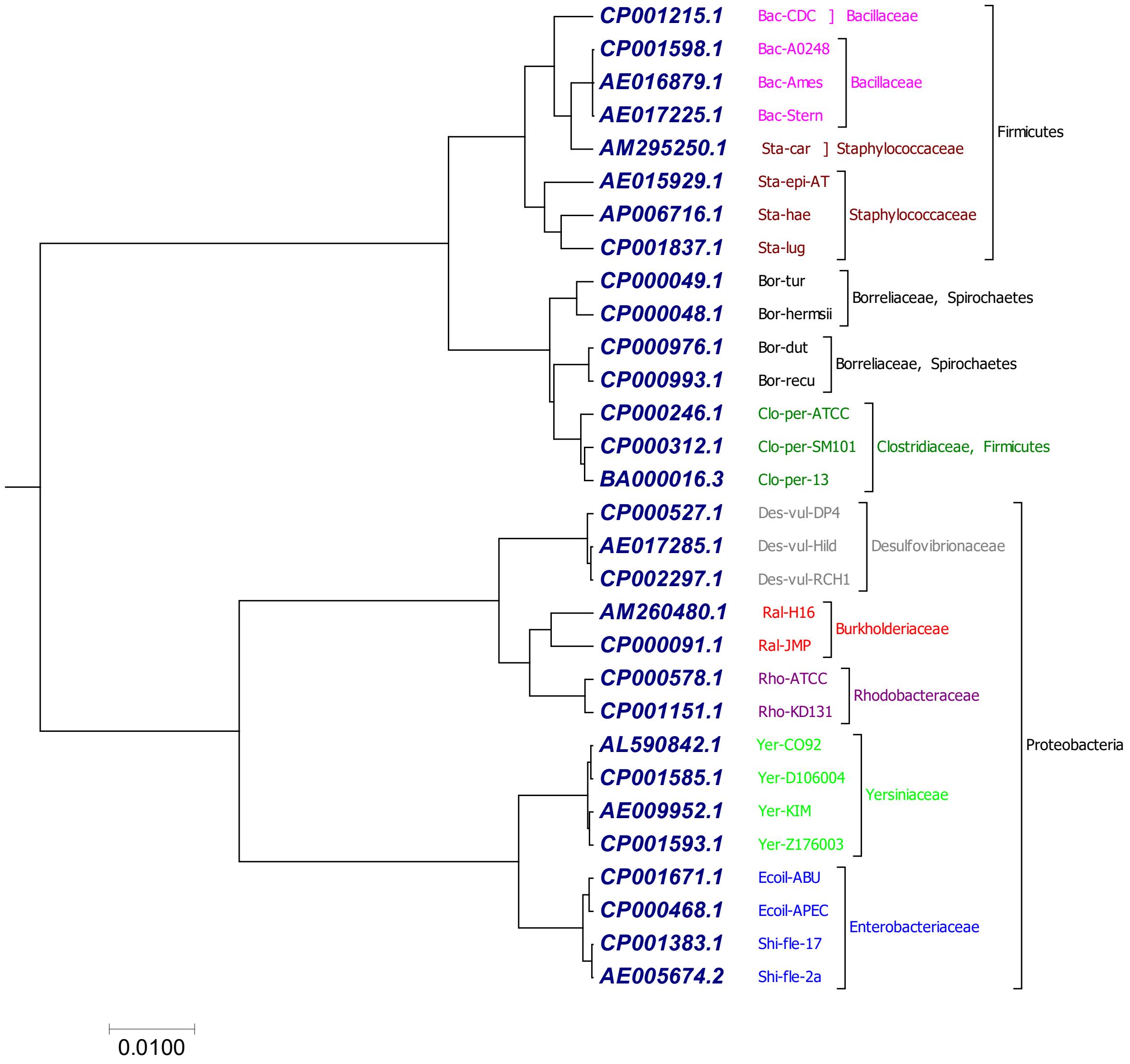

Figure 25: Our method.

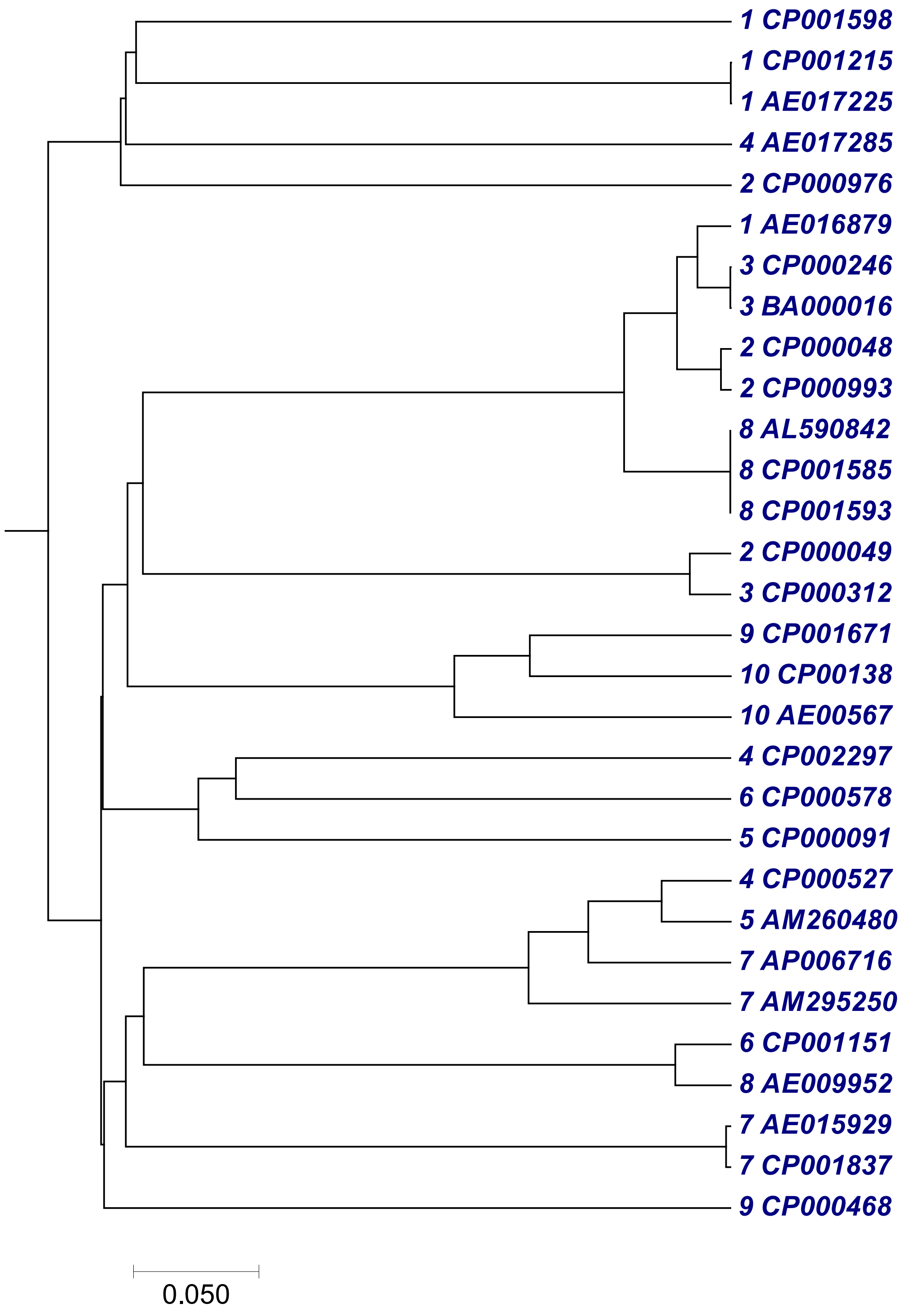

Figure 26: CV method using string length 3.

(1→Bacilleceae, 2→Borreliaceae, 3→Clostridiaceae, 4→Desulfovibrionaceae, 5→Burkholderiaceae, 6→Rhodobacteriaceae, 7→Staphylococcaceae , 8→Yersiniaceae, and 9→Enterobacteriaceae**).**

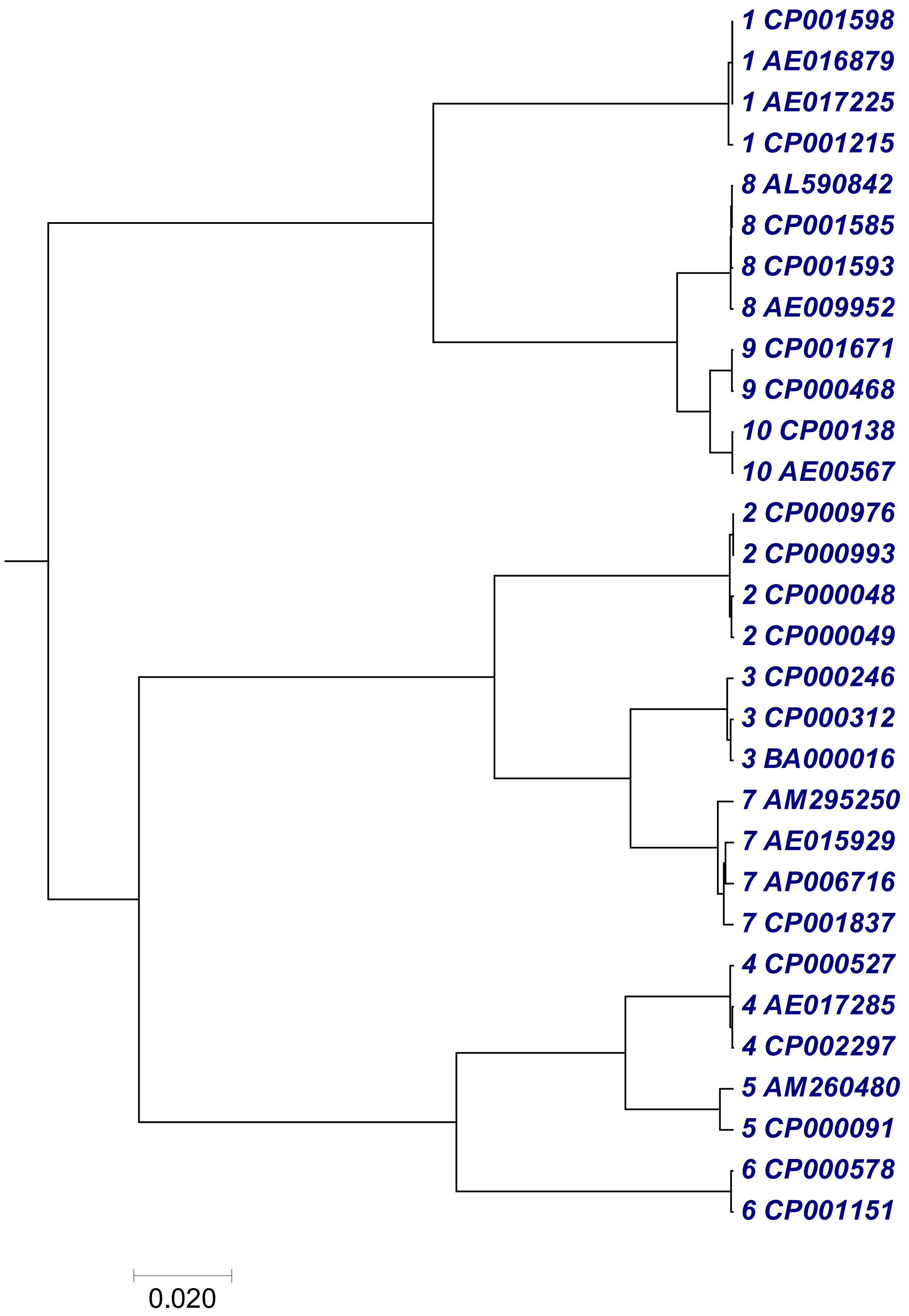

Figure 27: FFP method using string length 7.

(1→Bacilleceae, 2→Borreliaceae, 3→Clostridiaceae, 4→Desulfovibrionaceae, 5→Burkholderiaceae, 6→Rhodobacteriaceae, 7→Staphylococcaceae , 8→Yersiniaceae, and 9→Enterobacteriaceae**).**

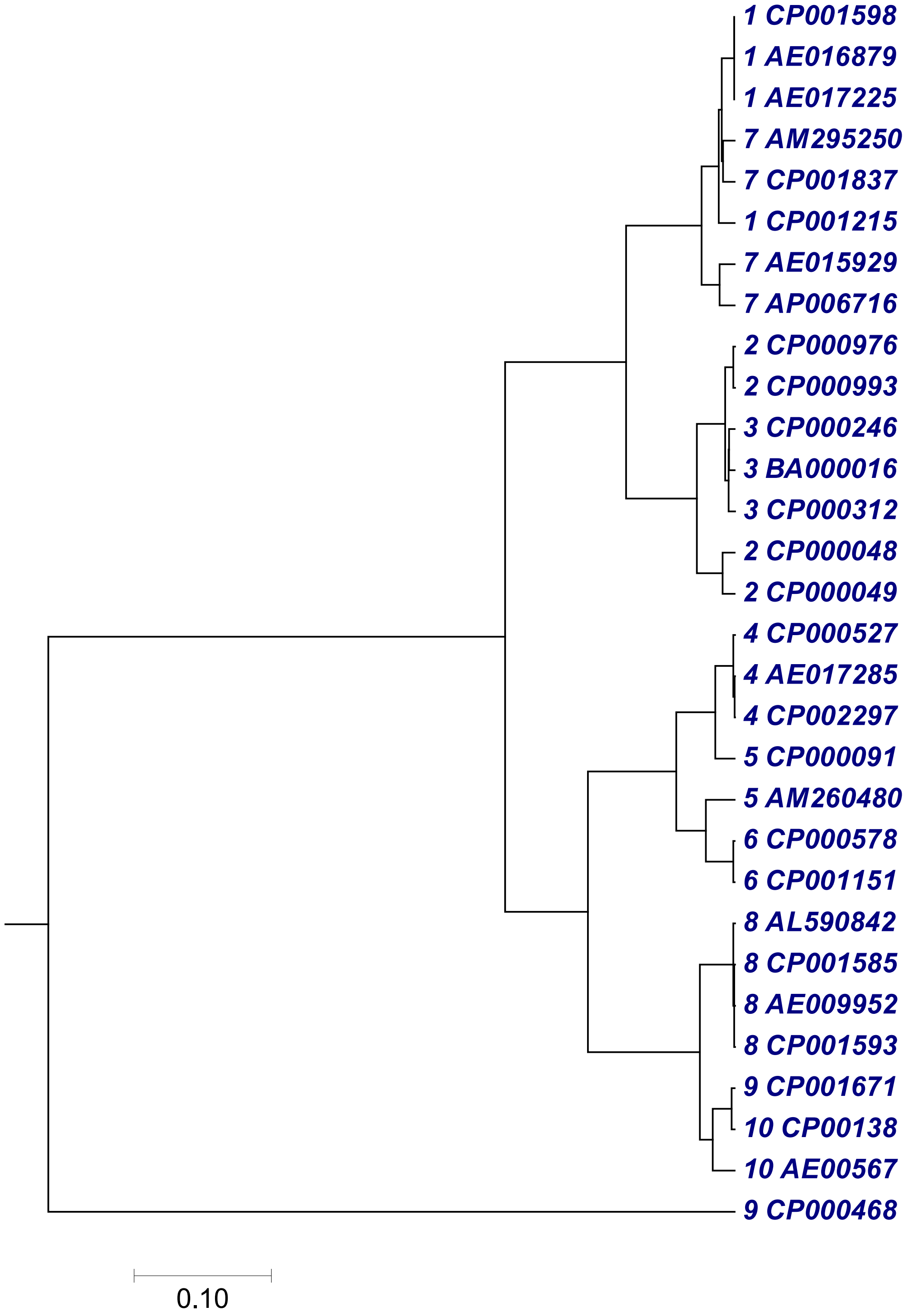

Figure 28: RTD method using string length 1.

(1→Bacilleceae, 2→Borreliaceae, 3→Clostridiaceae, 4→Desulfovibrionaceae, 5→Burkholderiaceae, 6→Rhodobacteriaceae, 7→Staphylococcaceae , 8→Yersiniaceae, and 9→Enterobacteriaceae**).**

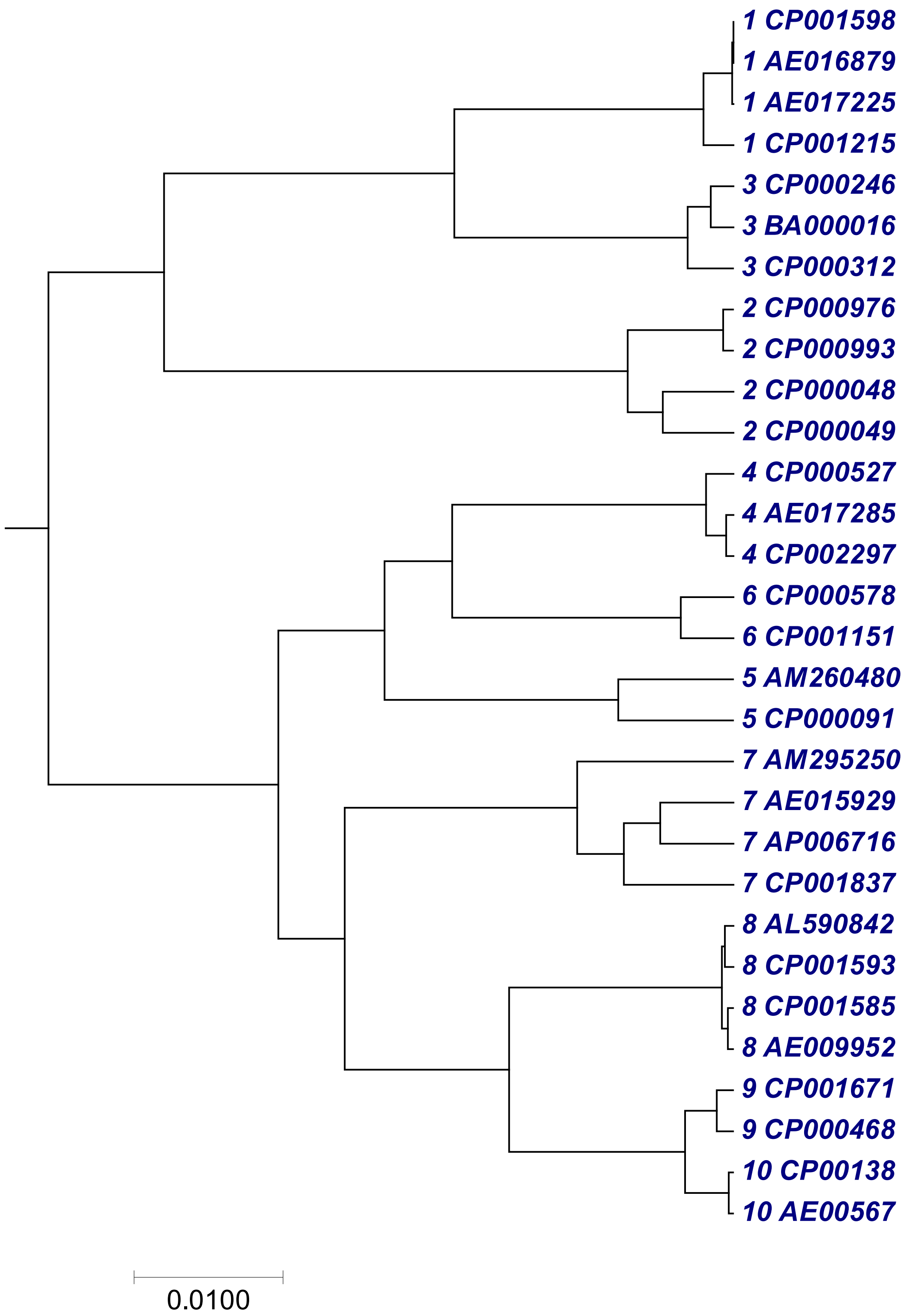

Figure 29: BBC method.

(1→Bacilleceae, 2→Borreliaceae, 3→Clostridiaceae, 4→Desulfovibrionaceae, 5→Burkholderiaceae, 6→Rhodobacteriaceae, 7→Staphylococcaceae , 8→Yersiniaceae, and 9→Enterobacteriaceae**).**

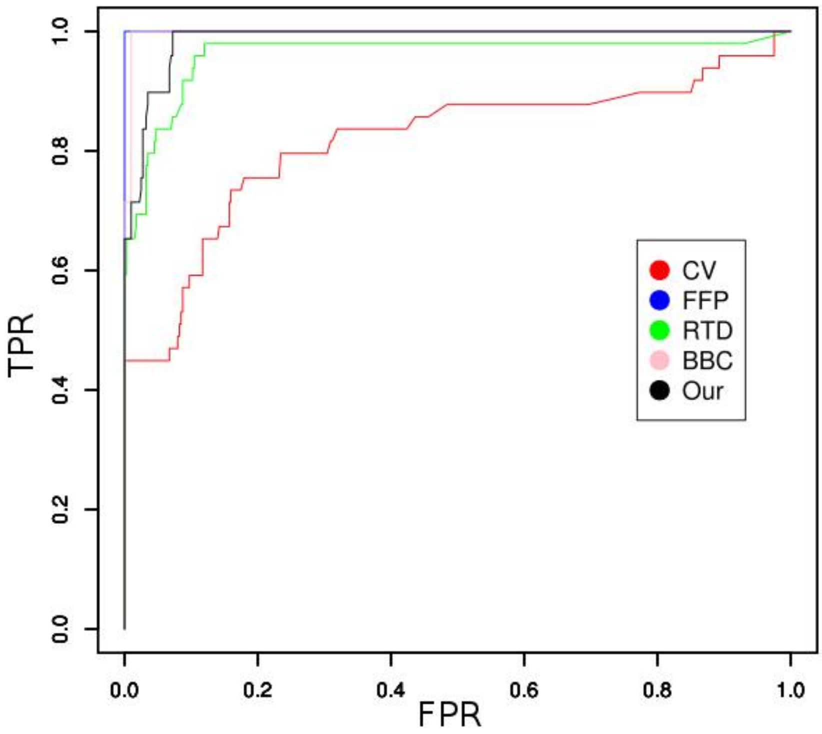

Dataset→ 30 bacterial		
**Methods↓**	**AUC↓**	**Running time↓**
CV method	0.814494	2m 6s
FFP method	1	1m
RTD method	0.960048	1m 14s
BBC method	0.99715	11m 33s
Our method	0.98692	3s

Figure 30: Receiver operating characteristic curve (ROC) and Area under the ROC Curve on 30 bacterial sequences.

The phylogenetic tree (Figure 25) generated by our method successfully clustered sequences based on taxnomic families such as, Burkholderiaceae, Rhodobacteriaceae, Enterobacteriaceae, Borreliaceae, Bacilleceae, Clostridiaceae, Desulfovibrionaceae, Yersiniaceae and Staphylococcaceae, which is lacking in Figures 26 and 28. However, our phylogenetic tree (Figure 25) has advantages at the phylum level over Figures 26, 27, 28 and 29. Our method (Figure 25) successfully clustered phylum Proteobacteria in a separate clade which were lacking in Figures 26, 27, 28 and 29. Moreover, the AUC of our method is 0.99 (Figure 30), which indicates that our method has high accuracy (Table 1).

Phylogenetic tree on 48 HEV sequences.

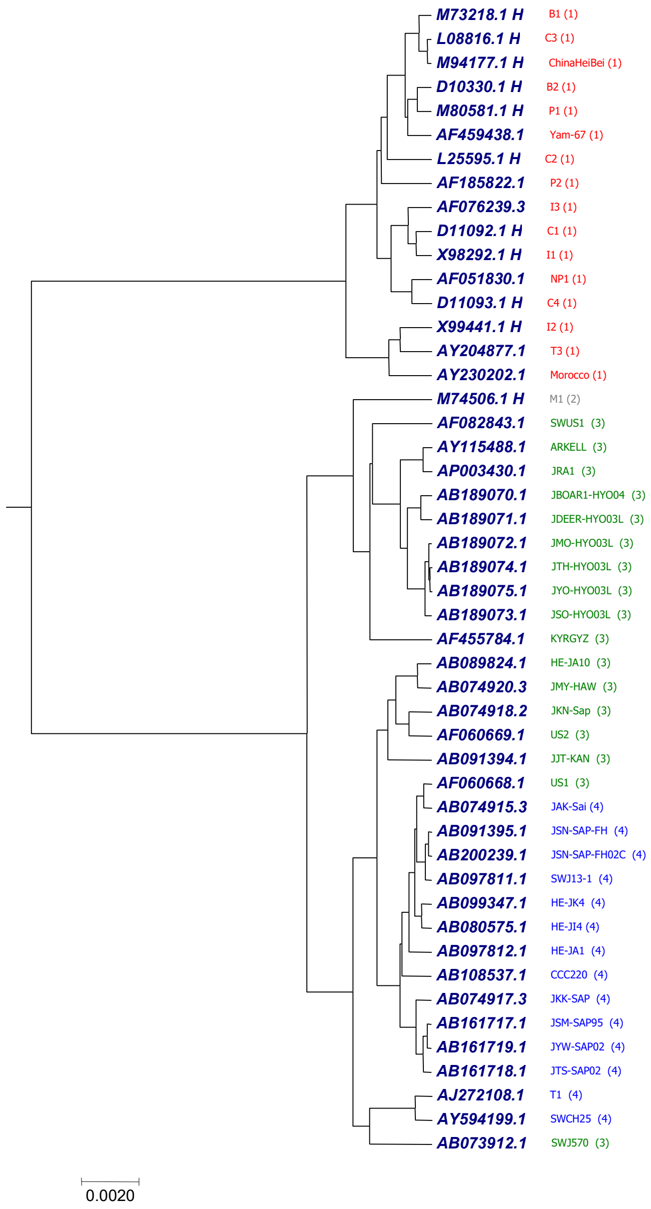

Figure 31: Our method.

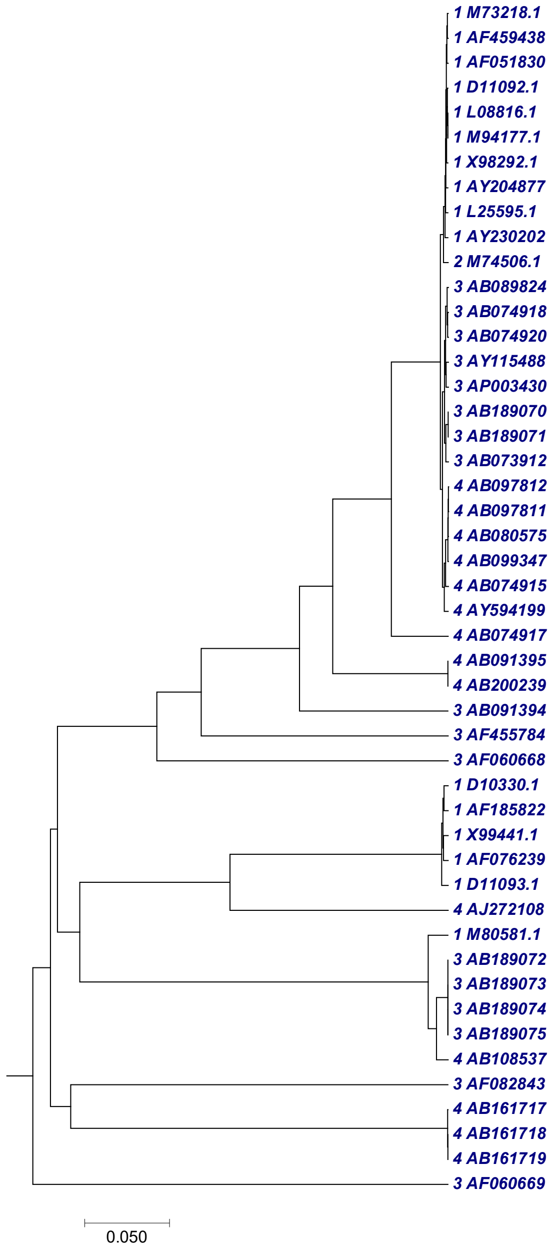

Figure 32: CV method using string length 3.

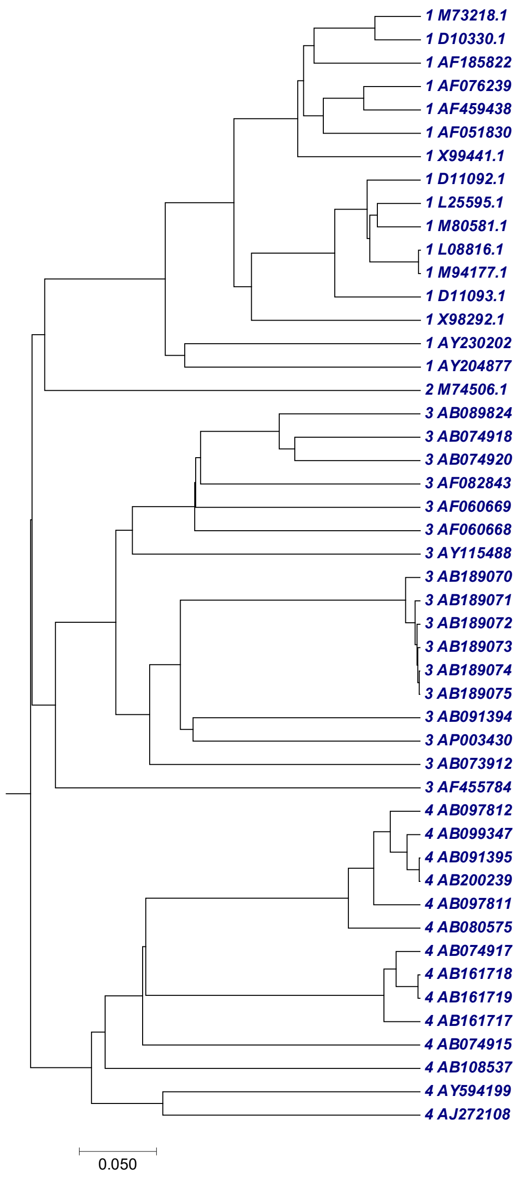

Figure 33: FFP method using string length 7.

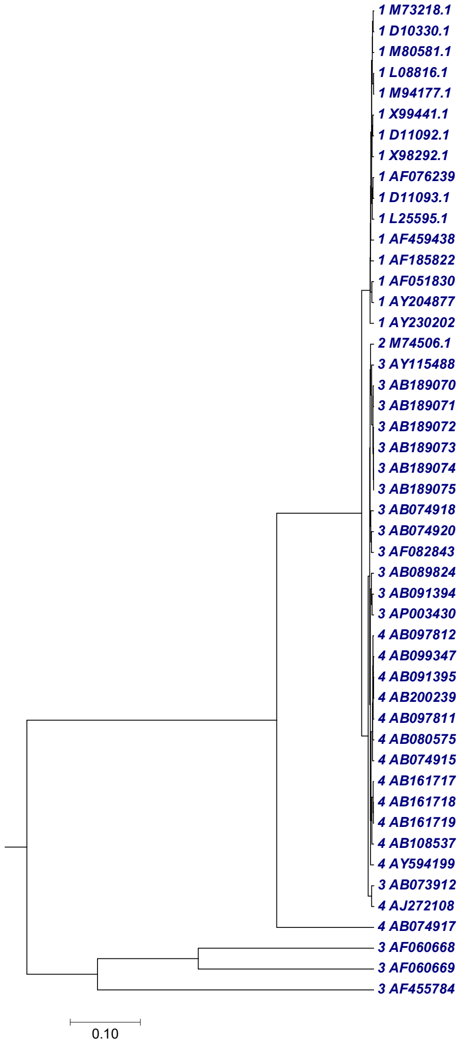

Figure 34: RTD method using string length 1.

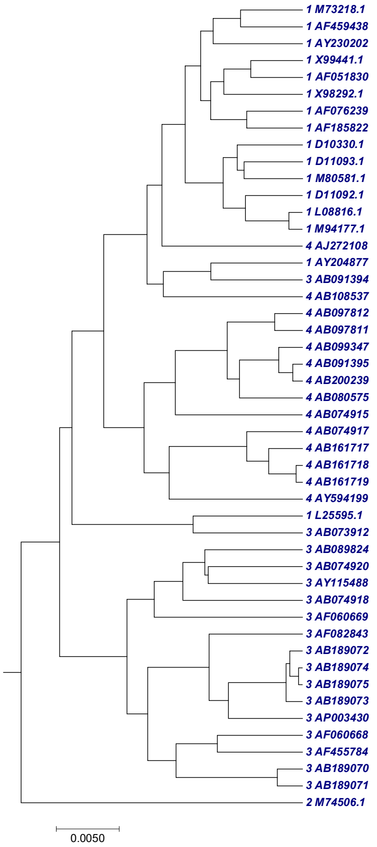

Figure 35: BBC method.

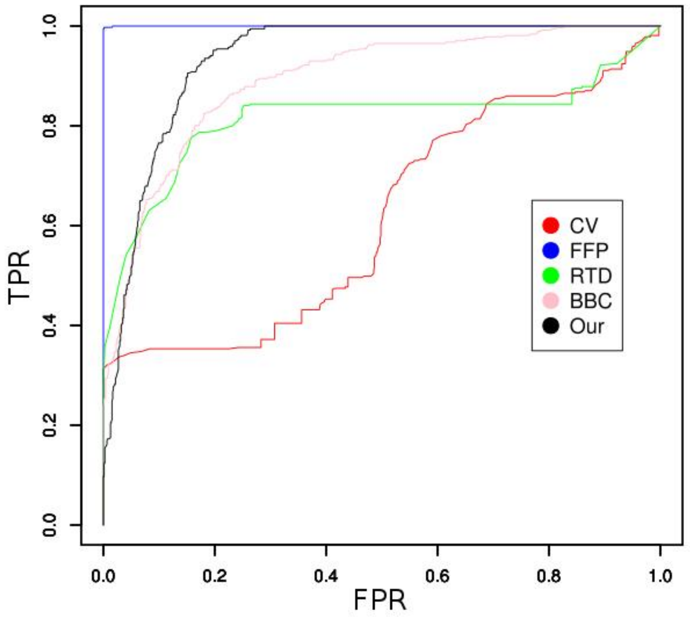

**Dataset→ 48 HEV**		
**Methods↓**	**AUC↓**	**Running time↓**
CV method	0.614583	<1s
FFP method	0.999952	1s
RTD method	0.808687	<1s
BBC method	0.891211	2s
Our method	0.933716	<1s

Figure 36: Receiver operating characteristic curve (ROC) and Area under the ROC Curve on 48 HEV sequences.

As shown in Figure 31 generated by our approach, the HEV genomes were divided into separate clades based on four genotypic categories (1(red),2(grey), 3(green) and 4(blue)) except few sequences. We compared the phylogenetic tree (Figure 31) generated by our approach with the phylogenetic trees (Figures 32, 33, 34 and 35) prepared by other freely available tools [1]. In Figure 32, four genotypic classes were not properly clustered. In Figures 34 and 35, four genotypic categories were separately clustered except few sequences. In Figure 33, all HEV genomes were correctly divided into separate clades based on four genotypic categories. Above performance shows the advantage of our method over other methods (Figures 32, 34 and 35) in terms of sequence clustering. Moreover, the AUC of our method is 0.93 (Figure 36), which indicates that our method has high accuracy (Table 1).

Phylogenetic tree on 58 mixed sequences.

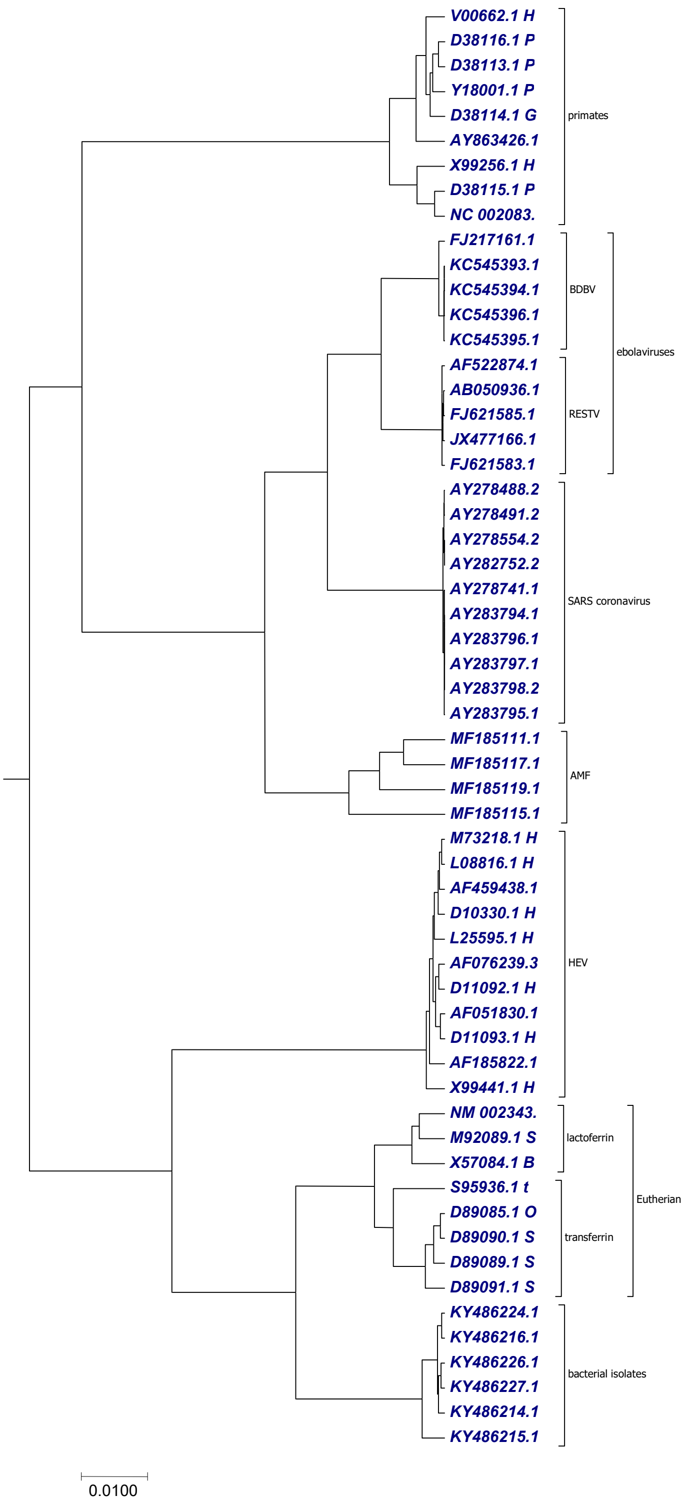

Figure 37: Our method.

(1→ primates, 2 → ebolavirus, 3→ SARS coronavirus, 4→ HEV, 5→ eutherian, 6 →AMF and 7→ bacterial isolates.)

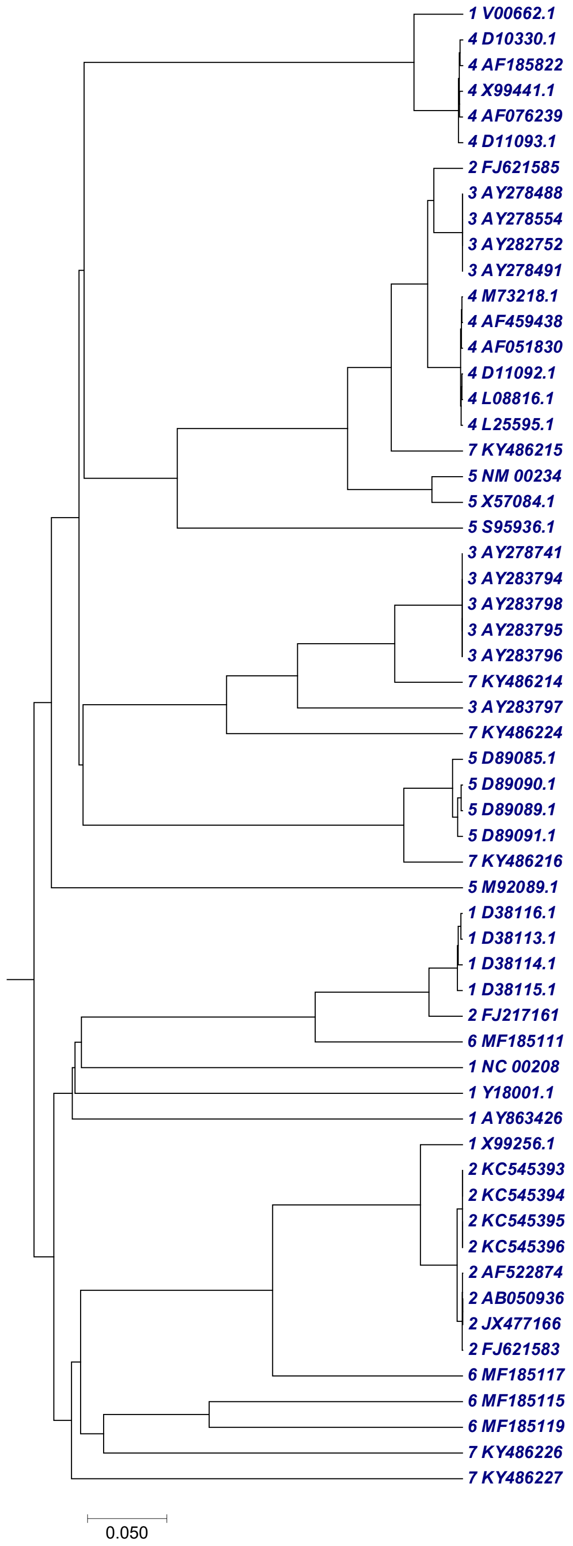

Figure 38: CV method using string length 3.

(1→ primates, 2 → ebolavirus, 3→ SARS coronavirus, 4→ HEV, 5→ eutherian, 6 →AMF and 7→ bacterial isolates.)

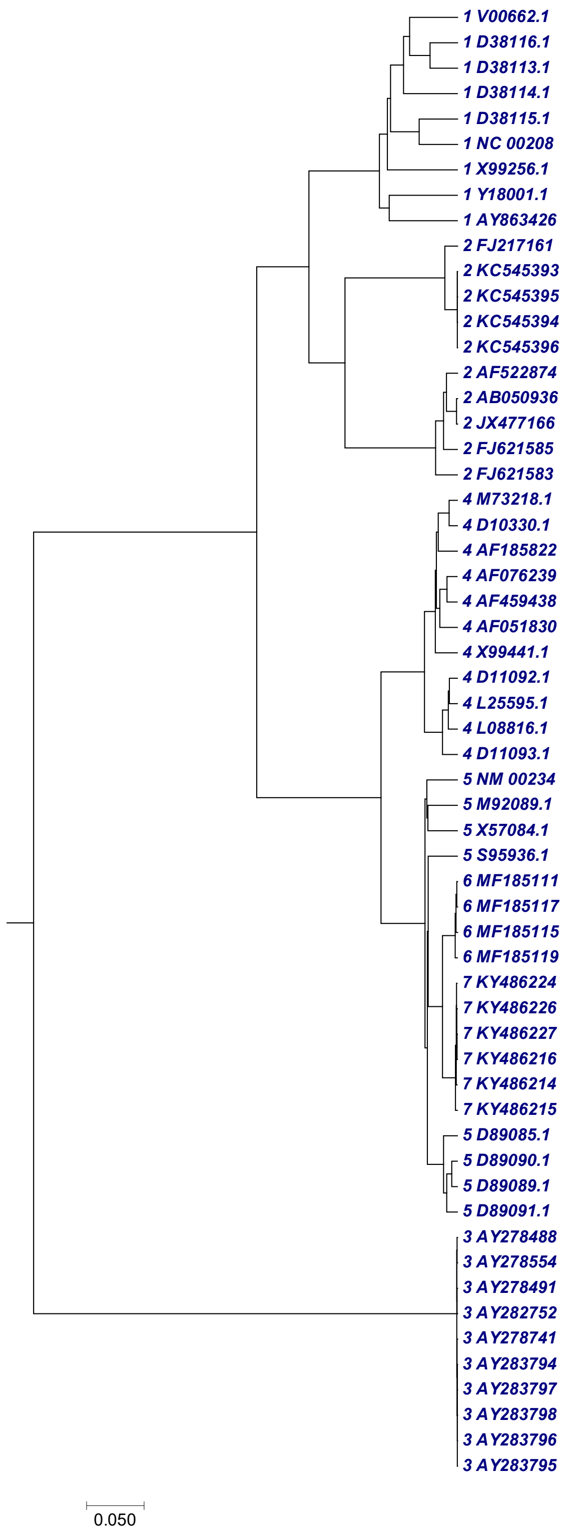

Figure 39: FFP method using string length 7.

(1→ primates, 2 → ebolavirus, 3→ SARS coronavirus, 4→ HEV, 5→ eutherian, 6 →AMF and 7→ bacterial isolates.)

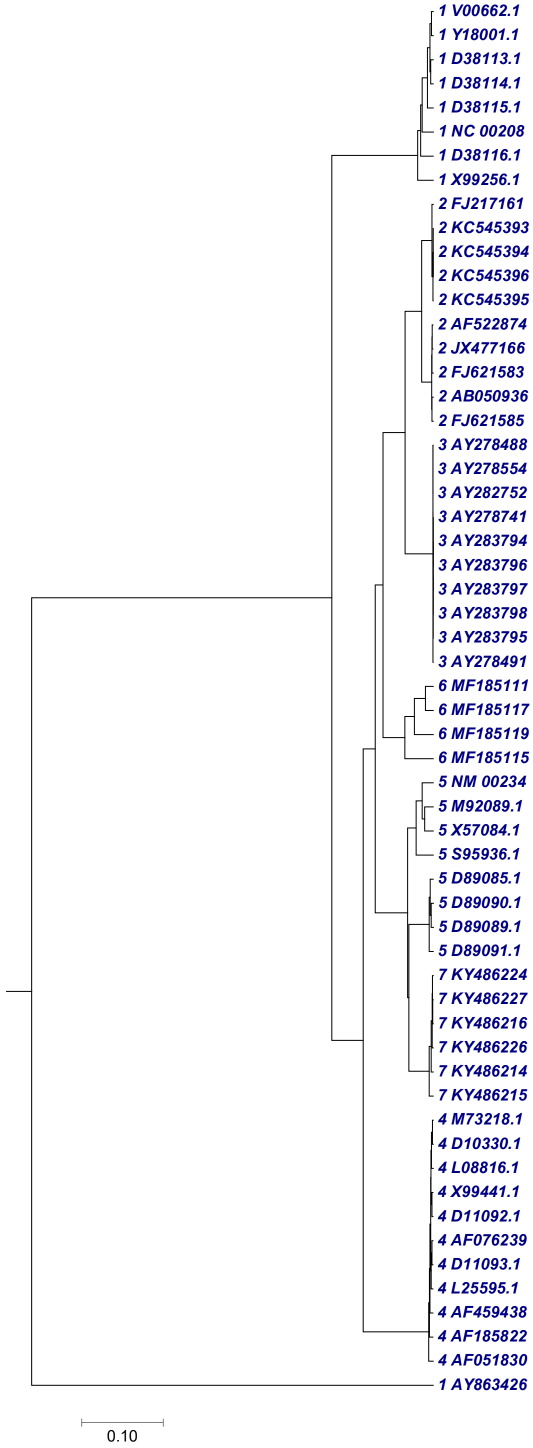

Figure 40: RTD method using string length 1.

(1→ primates, 2 → ebolavirus, 3→ SARS coronavirus, 4→ HEV, 5→ eutherian, 6 →AMF and 7→ bacterial isolates.)

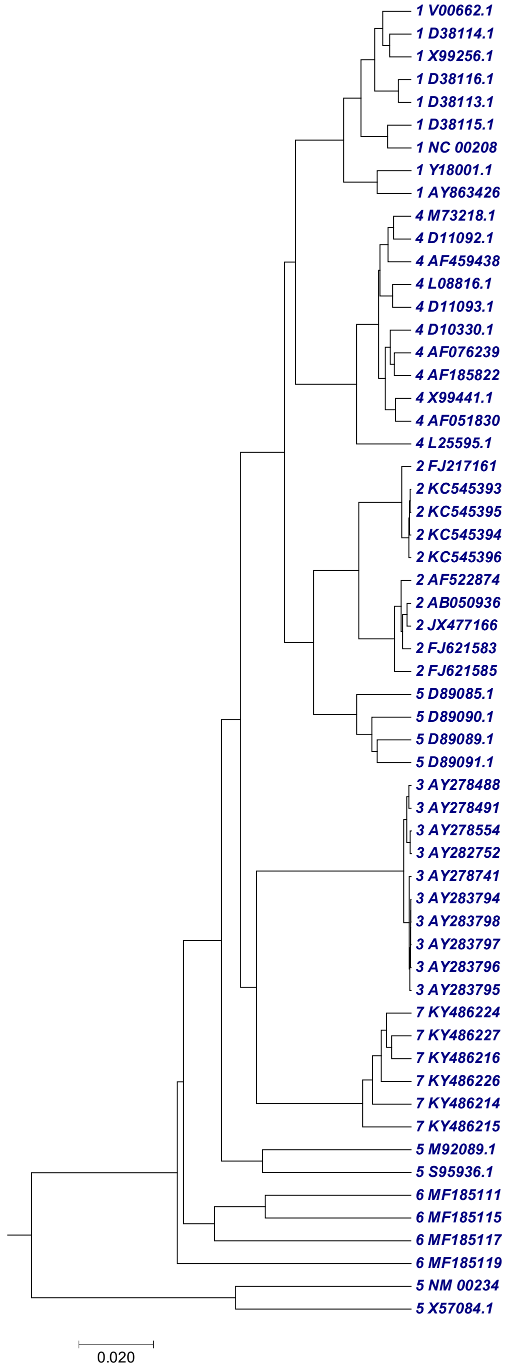

Figure 41: BBC method.

(1→ primates, 2 → ebolavirus, 3→ SARS coronavirus, 4→ HEV, 5→ eutherian, 6 →AMF and 7→ bacterial isolates.)

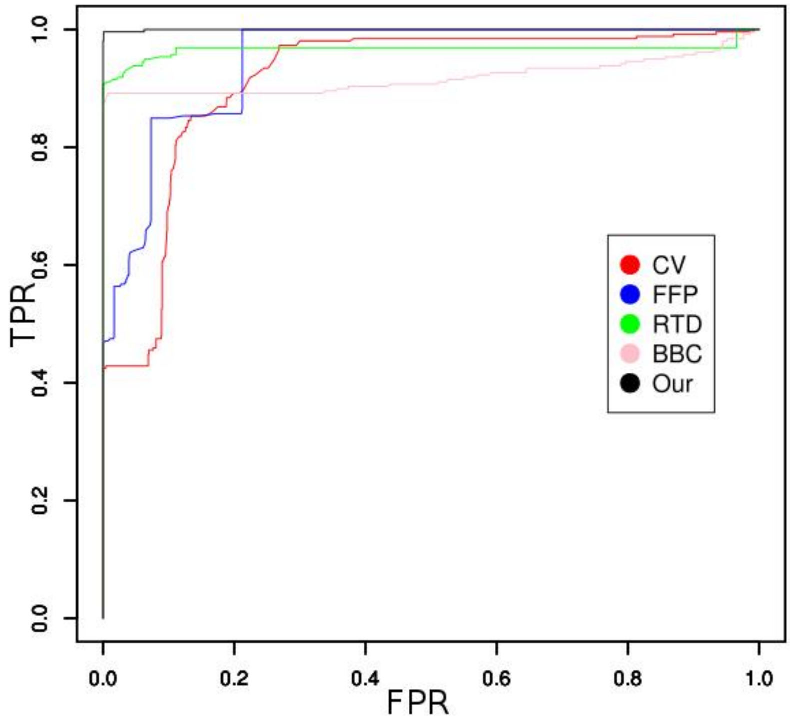

Dataset→ 58 mixed		
**Methods↓**	**AUC↓**	**Running time↓**
CV method	0.91396	1s
FFP method	0.948556	2s
RTD method	0.966549	<1s
BBC method	0.918997	5s
Our method	0.999748	<1s

Figure 42: Receiver operating characteristic curve (ROC) and Area under the ROC Curve on 58 mixed sequences.

We collected 58 genome dataset from different species, which includes nine primates mammalian mitochondrial genomes, ten ebolavirus (five reston virus (RESTV), five bundibugyo virus (BDBV)) complete genomes, ten SARS coronavirus, eleven hepatitis E virus, eight eutherian mammal, four arbuscular mycorrhizal fungi and six bacterial isolates. As shown in Figure 37 generated by our method, we observed that all the different species genome datasets were clustered separately. We compared the phylogenetic tree (Figure 37) generated by our approach with the phylogenetic trees (Figures 38, 39, 40 and 41) prepared by other freely available tools [1]. In Figure 38, 58 genome datasets from different species were not properly clustered. In Figures 39, 40 and 41, eight eutherian mammals were divided into more than one clades. . In addition to the superior phylogenetic tree, the AUC of our method is 0.99 (Figure 42), which indicates that our method has high accuracy (Table 1).

Phylogenetic tree on 29 escherichia/shigella sequences.

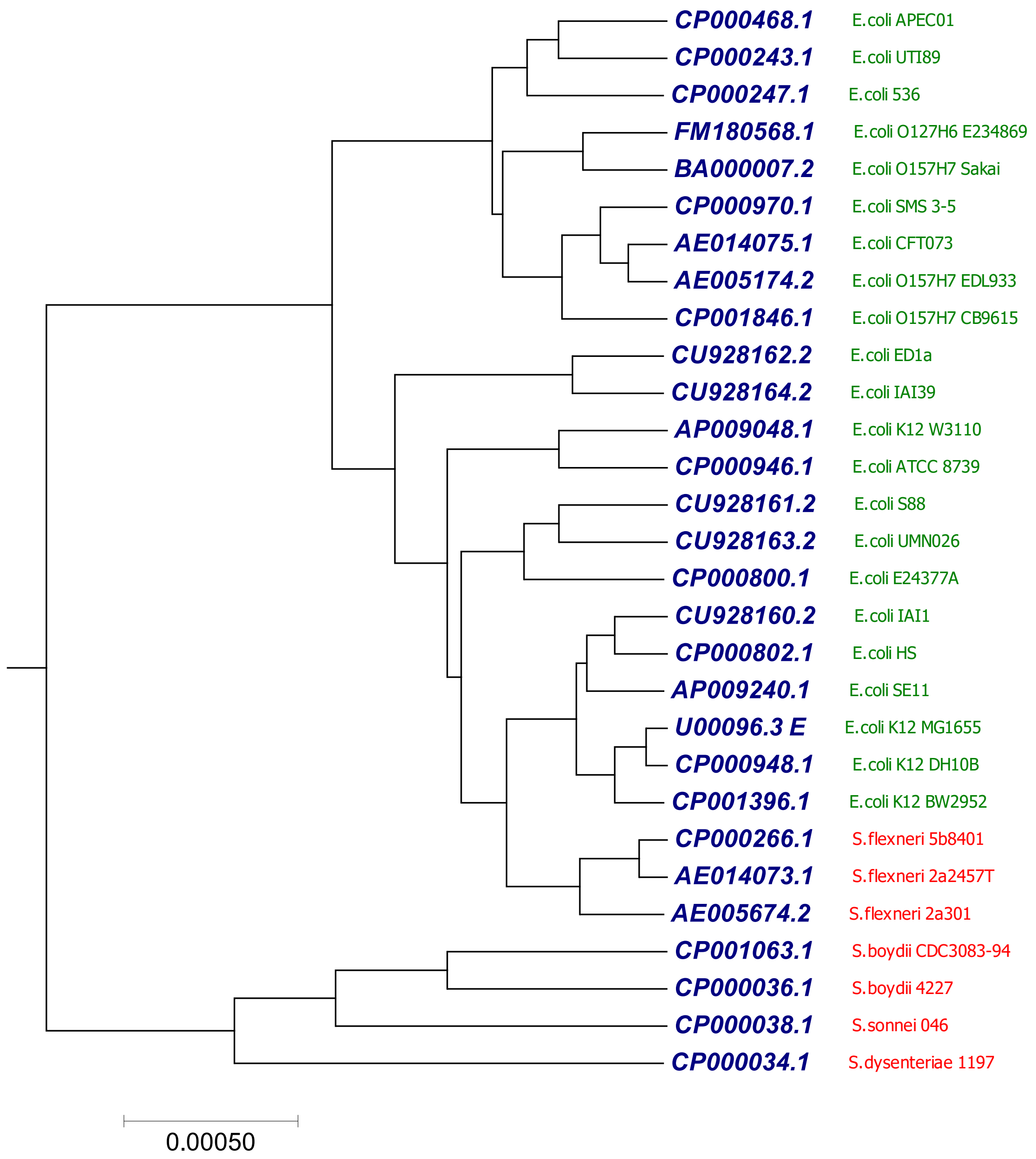

Figure 43: Our method.

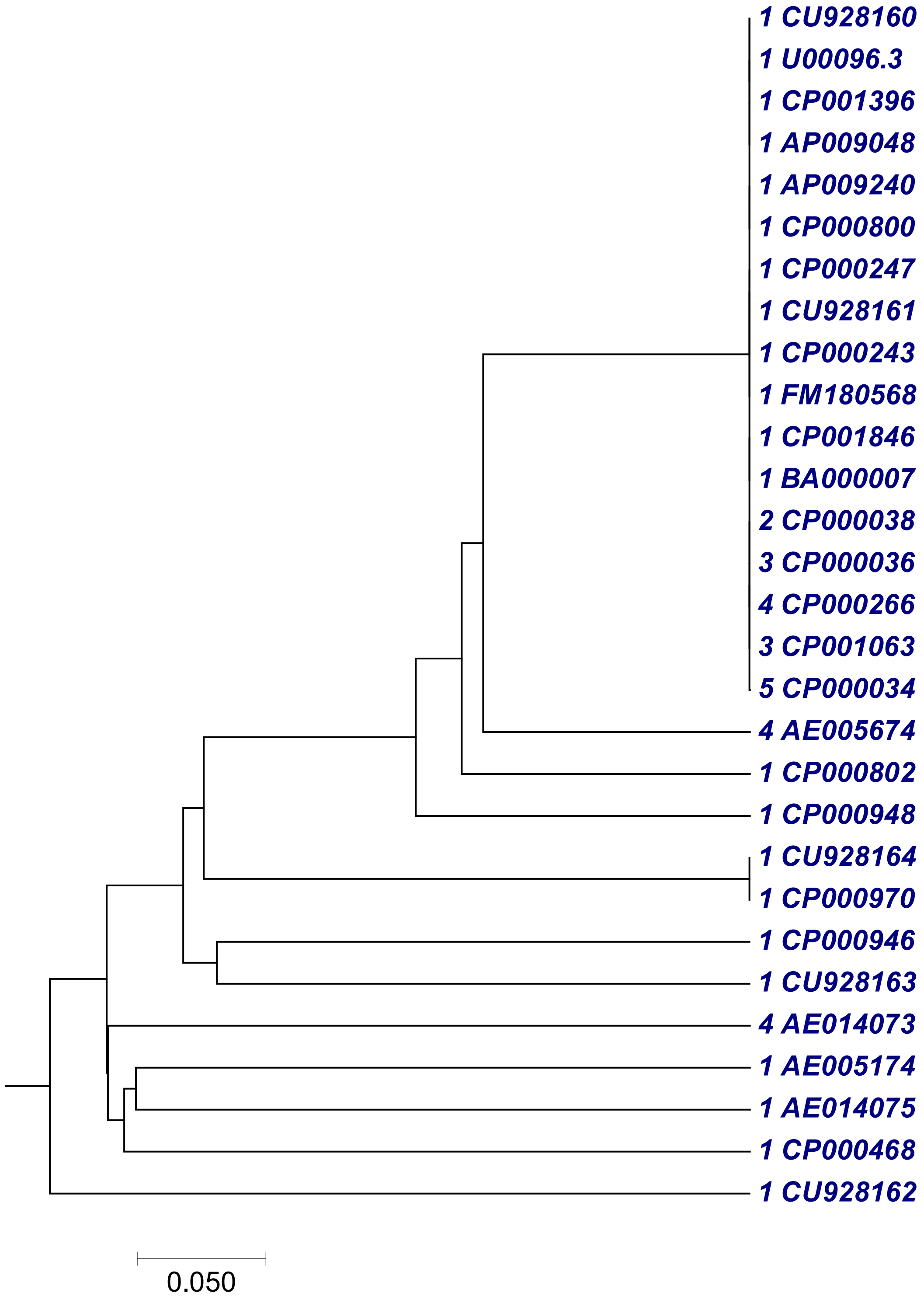

Figure 44: CV method using string length 3.

(1→ E.coli, 4→S.flexneri, 3→S.boydii, 2→S.sonnei and 5→S.dysenteriae).

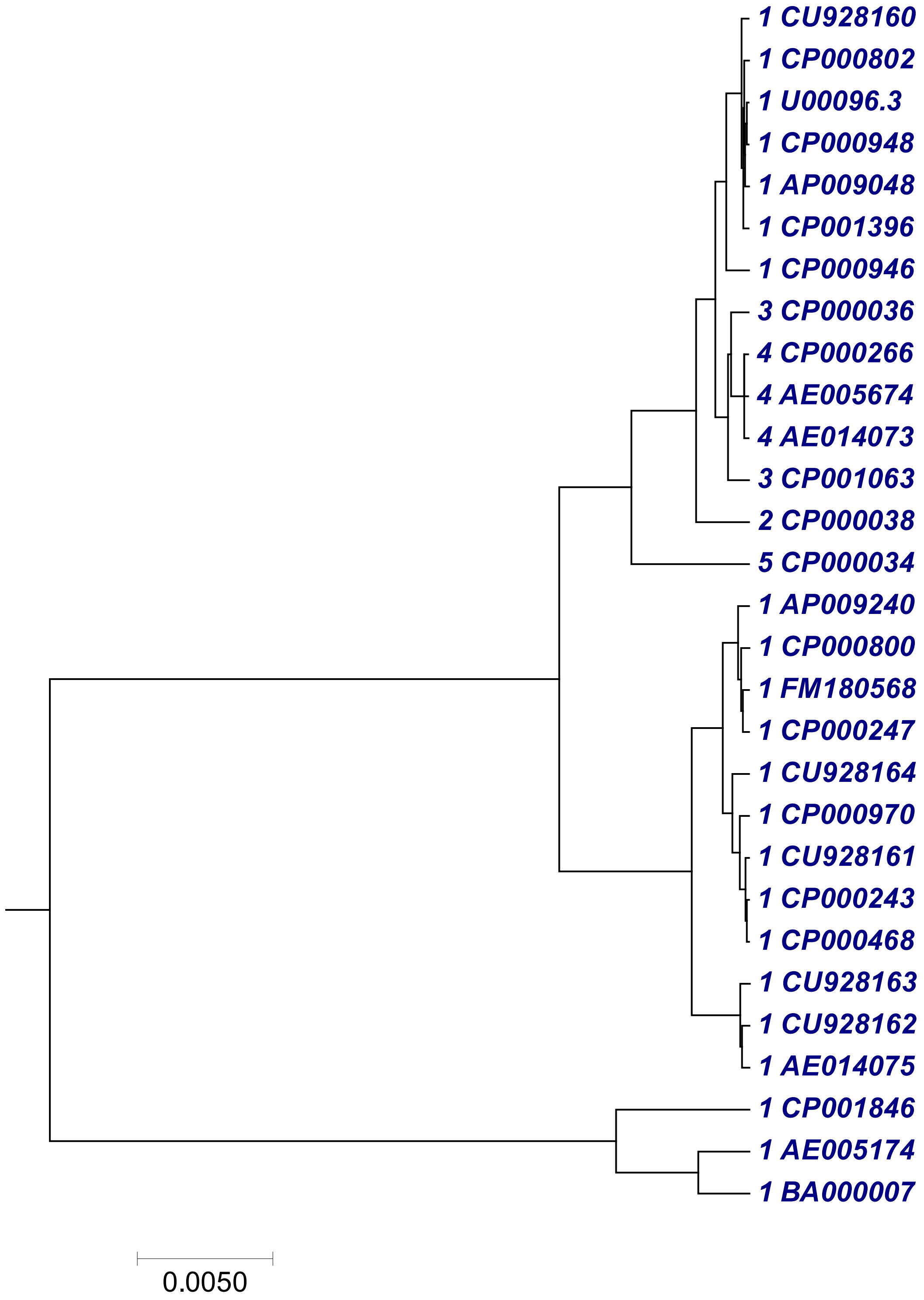

Figure 45: FFP method using string length 7.

(1→ E.coli, 4→S.flexneri, 3→S.boydii, 2→S.sonnei and 5→S.dysenteriae).

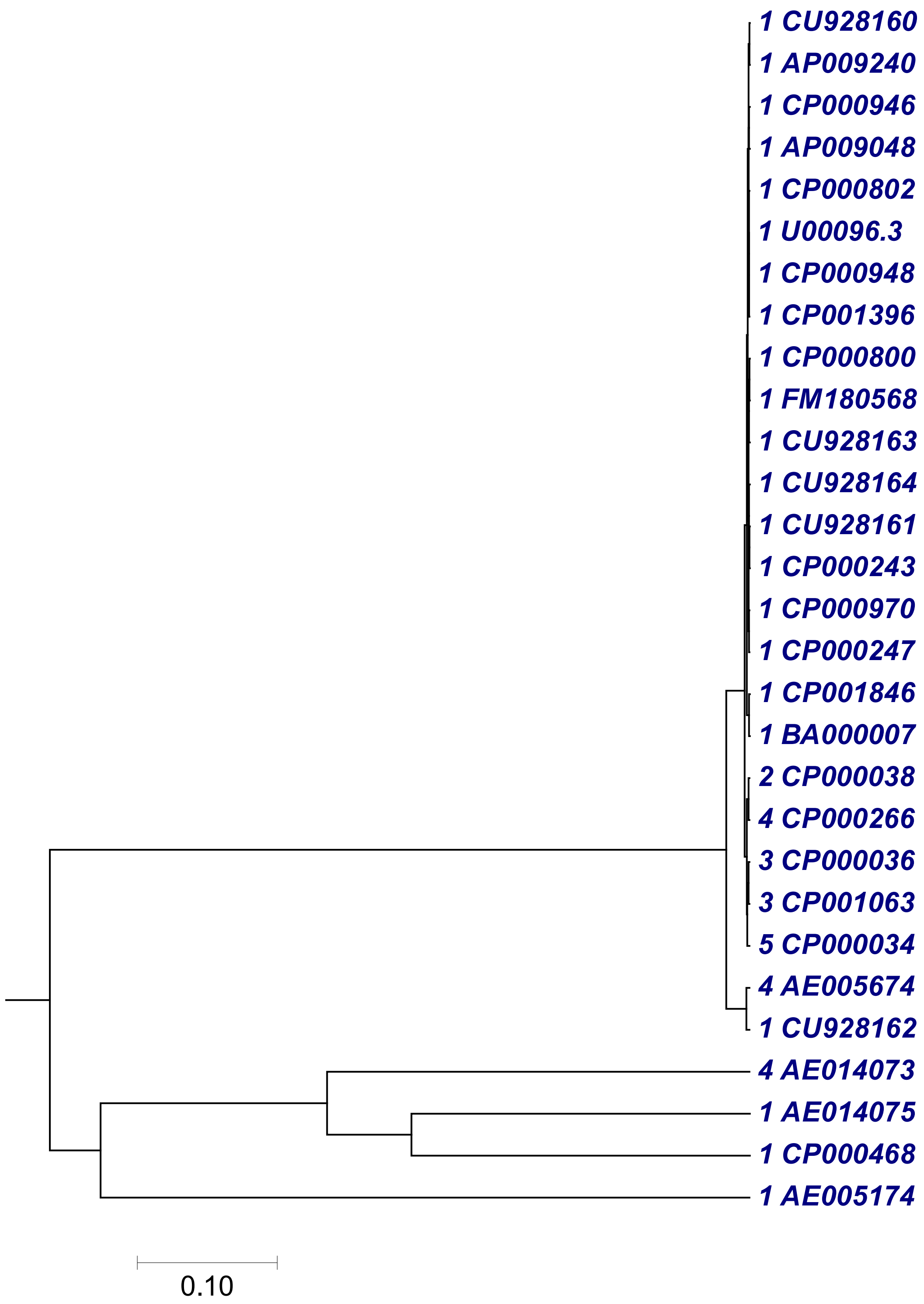

Figure 46: RTD method using string length 1.

(1→ E.coli, 4→S.flexneri, 3→S.boydii, 2→S.sonnei and 5→S.dysenteriae).

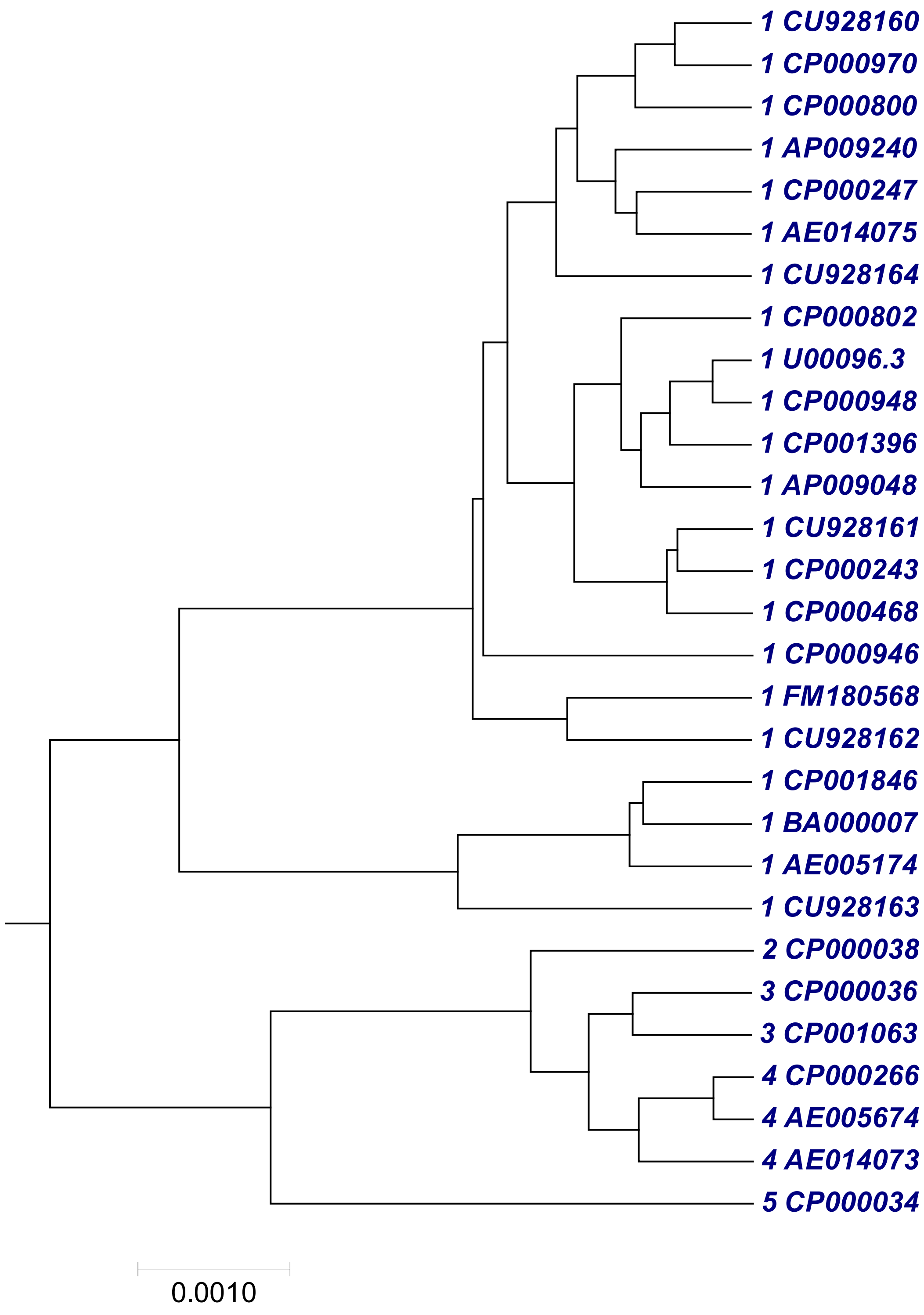

Figure 47: BBC method.

(1→ E.coli, 4→S.flexneri, 3→S.boydii, 2→S.sonnei and 5→S.dysenteriae).

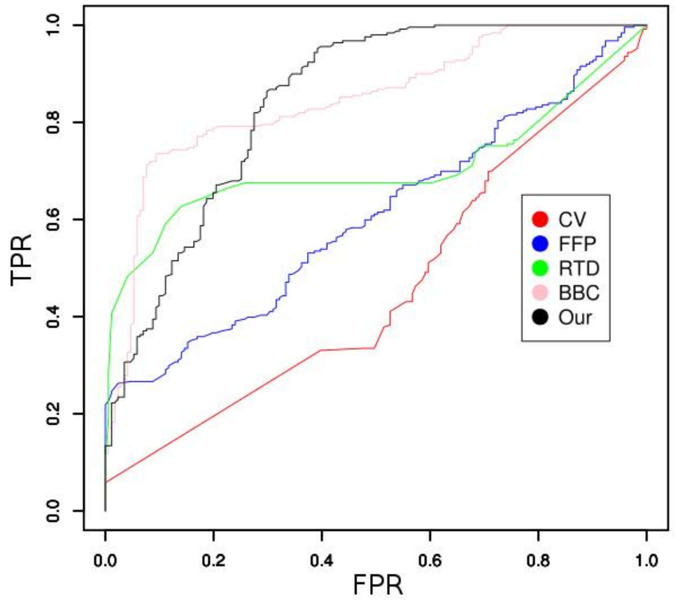

Dataset→	29 Escherichia/ Shigella	
Methods↓	AUC↓	Running time↓
CV method	0.377468	1m 45s
FFP method	0.60405	1m 2s
RTD method	0.671132	1m 6s
BBC method	0.84575	16m 52s
Our method	0.842633	3s

Figure 48: Receiver operating characteristic curve (ROC) and Area under the ROC Curve on 29 Escherichia/Shigella sequences.

The phylogenetic tree (Figure 43) generated by our method successfully clustered all organisms belongs to Escherichia coli, Shigella flexneri and Shigella boydii in separate clades. While comparing the tree prepared by our method (Figure 43) with the tree prepared by other freely available tools [1] (Figures 44, 45, 46 and 47). We found that, all organisms belong to Escherichia coli were not properly clustered in Figures 44, 45 and 46. However, Figure 47 has similarity with our result. However, the AUC of our method is 0.84 (Figure 48), which indicates that our method has moderate accuracy (Table 1).

Phylogenetic tree on 24 eutherian sequences.

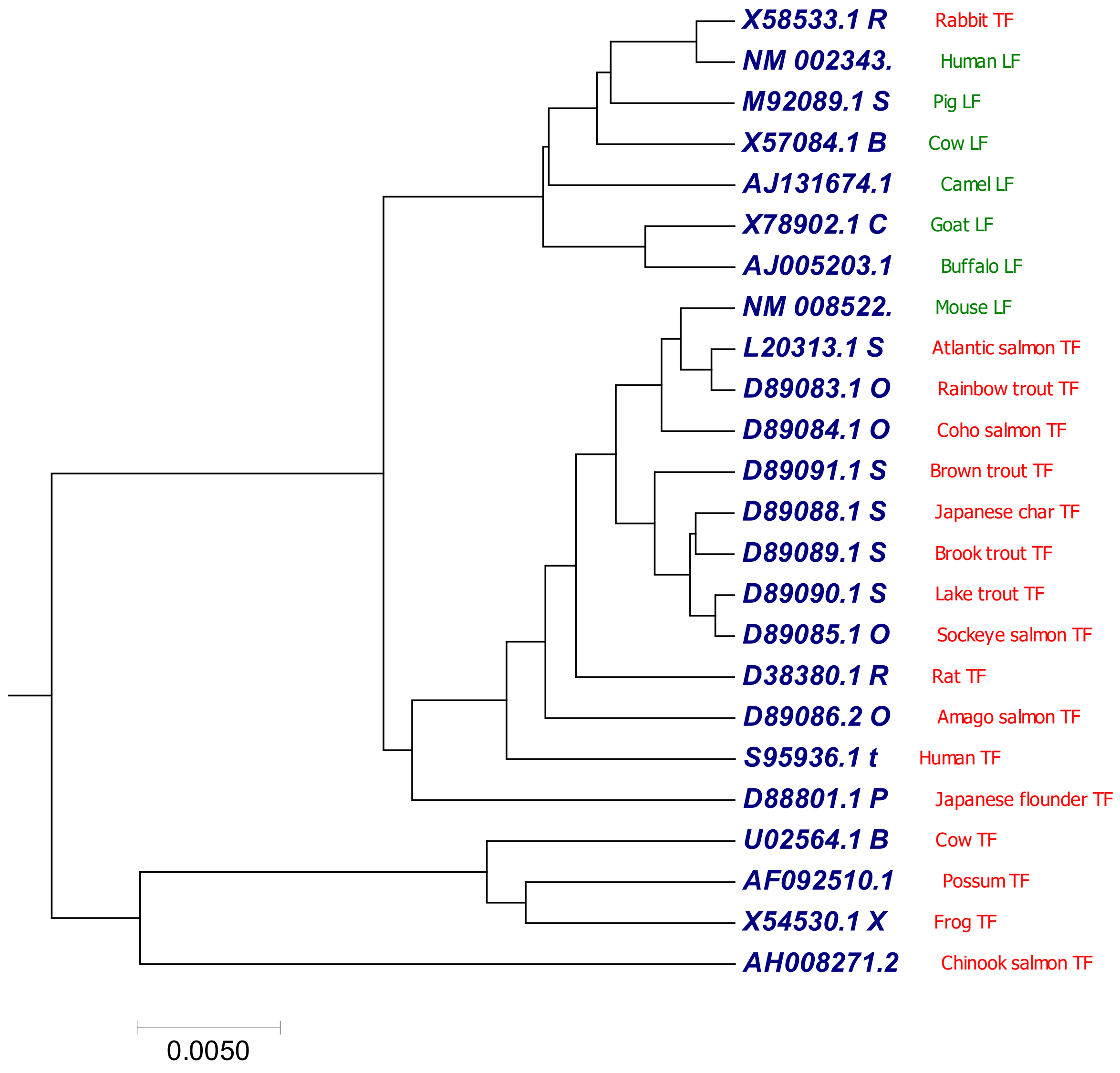

Figure 49: Our method.

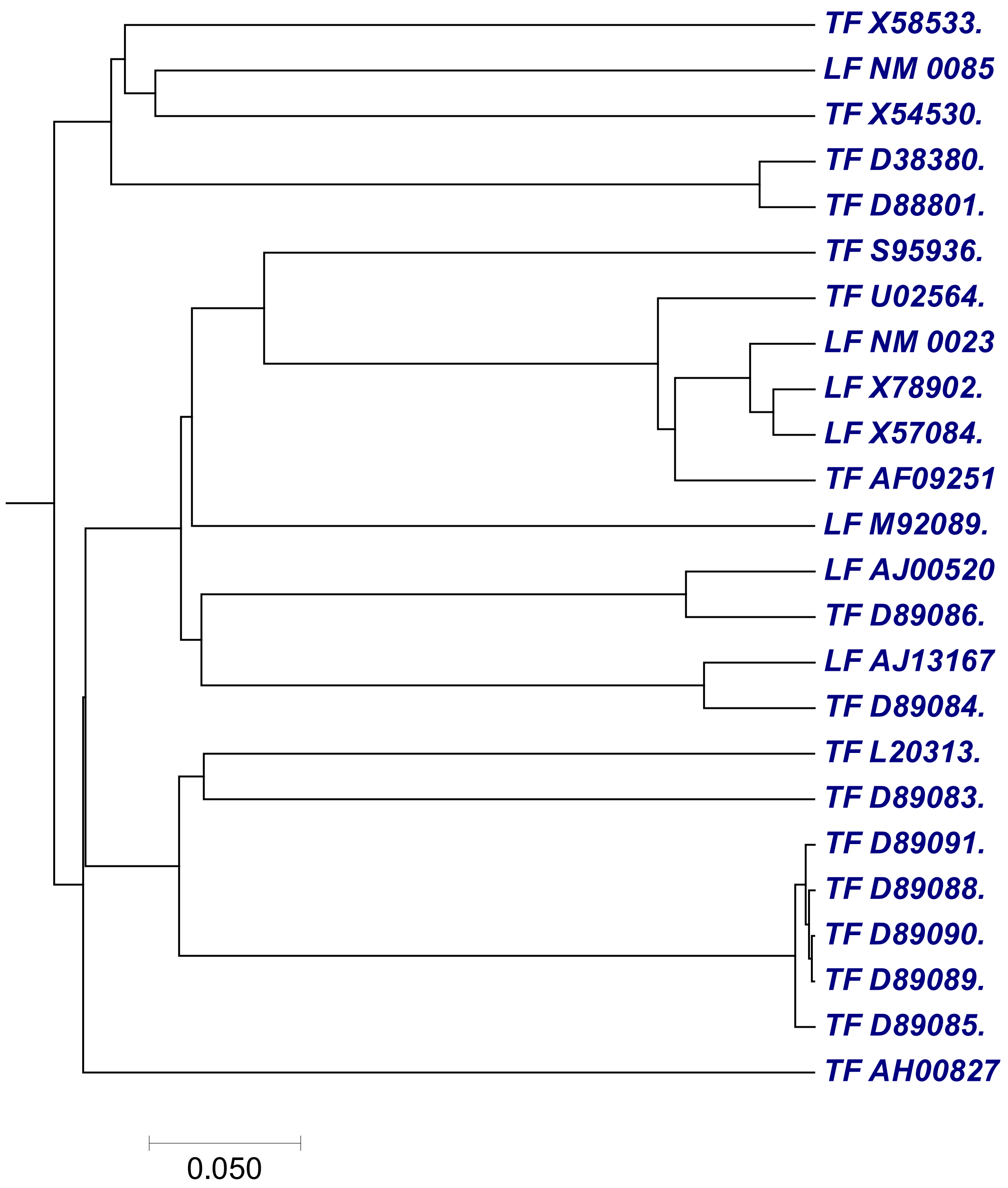

Figure 50: CV method using string length 3.

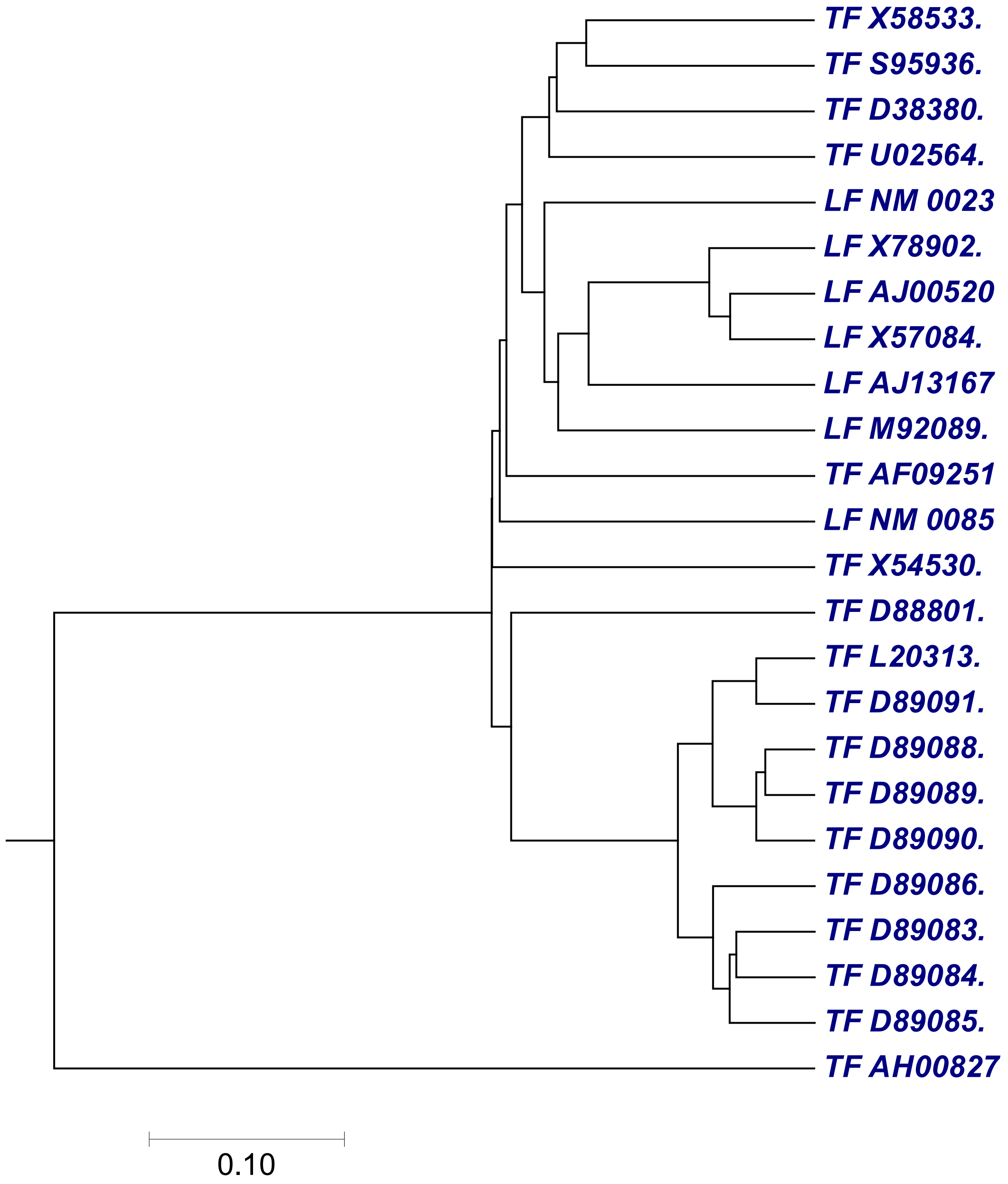

Figure 51: FFP method using string length 7.

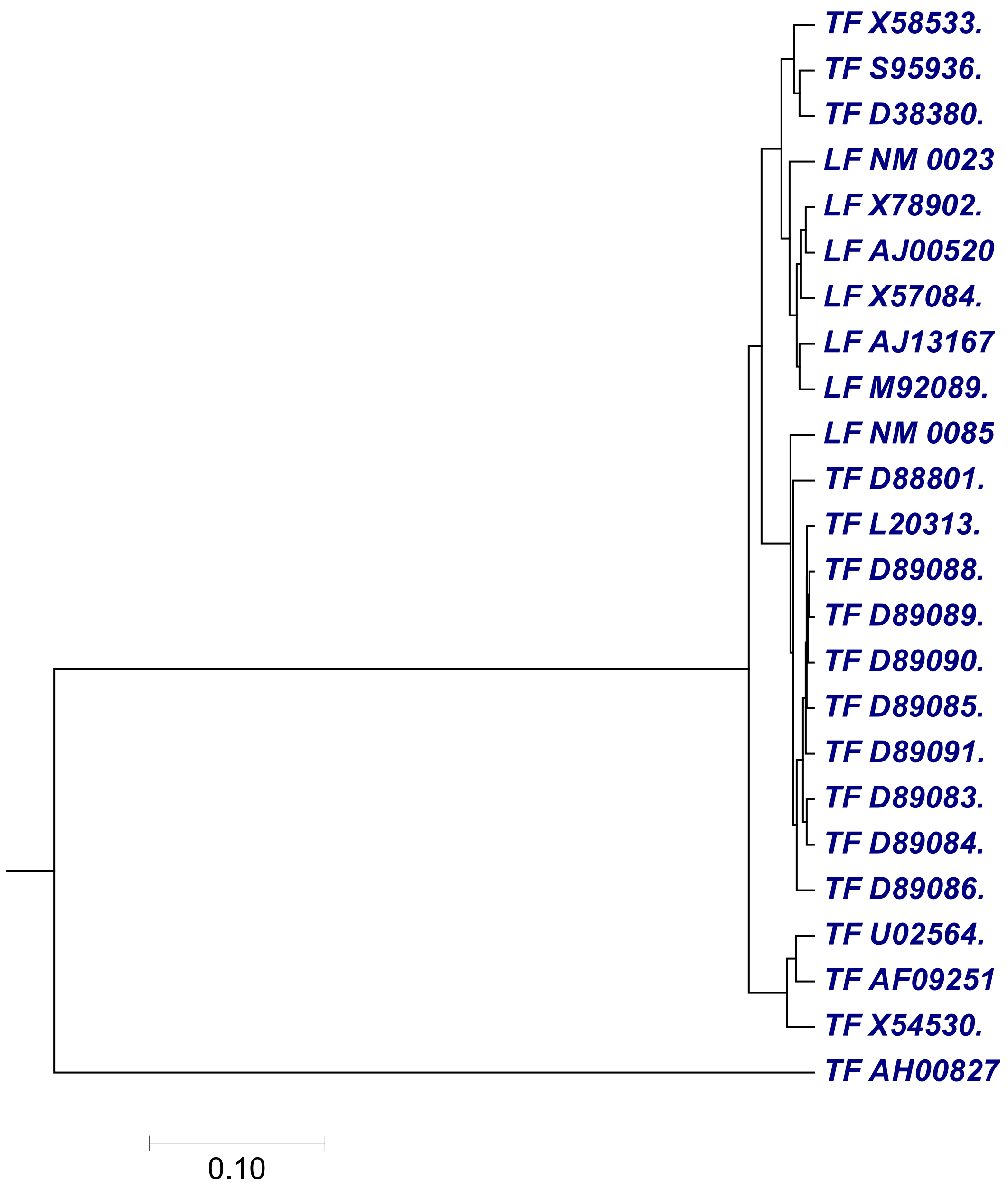

Figure 52: RTD method using string length 1.

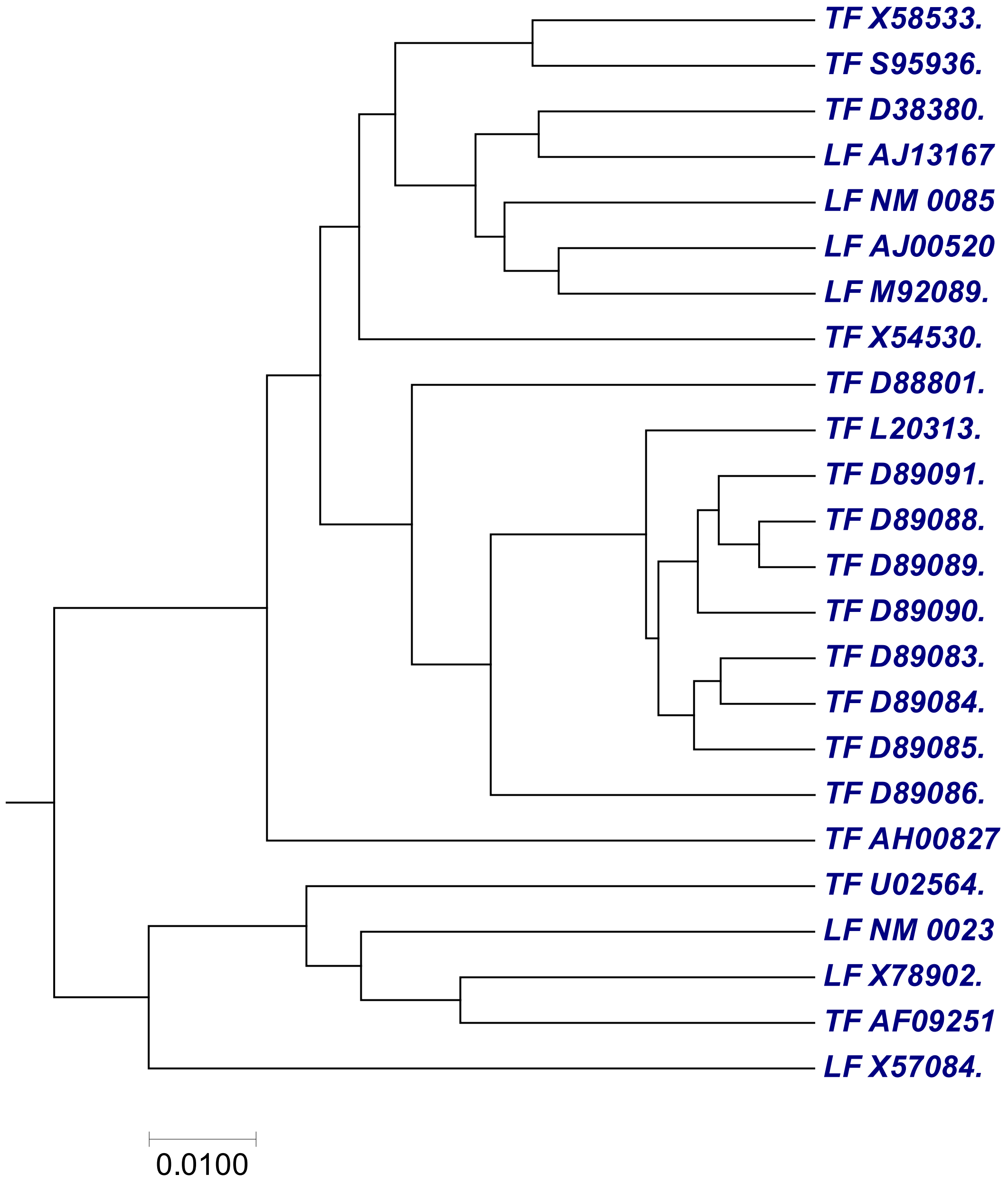

Figure 53: BBC method.

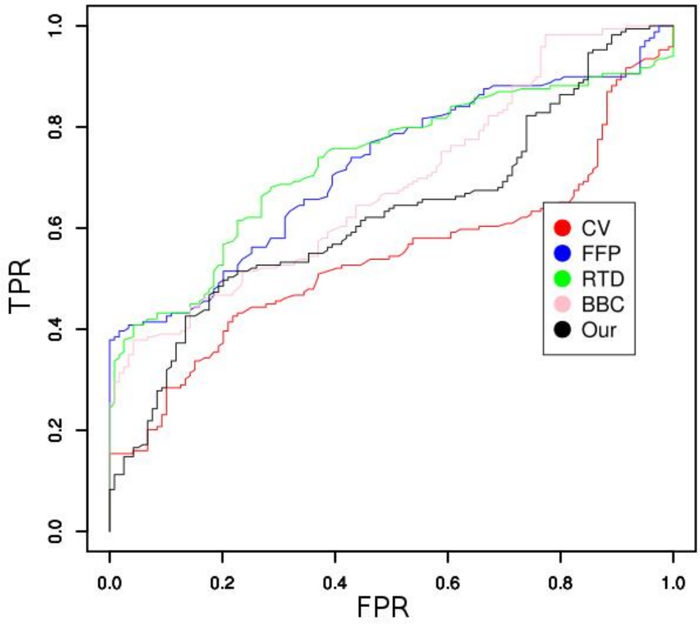

Dataset→ 24 Eutherian mammal		
**Methods↓**	**AUC↓**	**Running time↓**
CV method	0.539058	<1s
FFP method	0.717816	<1s
RTD method	0.727413	<1s
BBC method	0.685147	<1s
Our method	0.627617	<1s

Figure 54: Receiver operating characteristic curve (ROC) and Area under the ROC Curve on 24 Eutherian mammal sequences.

As shown in Figure 49 generated by our method, we can observe that all transferrin sequences (red) were clustered into two distinct clades, except rabbit transferrin sequence which was grouped with lactoferrin class. Similarly, all lactoferrin sequences (green) were clustered together, except mouse lactoferrin sequence which was grouped with transferrin class. However, the trees (Figures 50, 51, 52 and 53) generated by other methods could not cluster the sequences properly as our method. Moreover, the AUC of our method is 0.63 (Figure 54).

Phylogenetic tree on 40 bacterial isolates sequences.

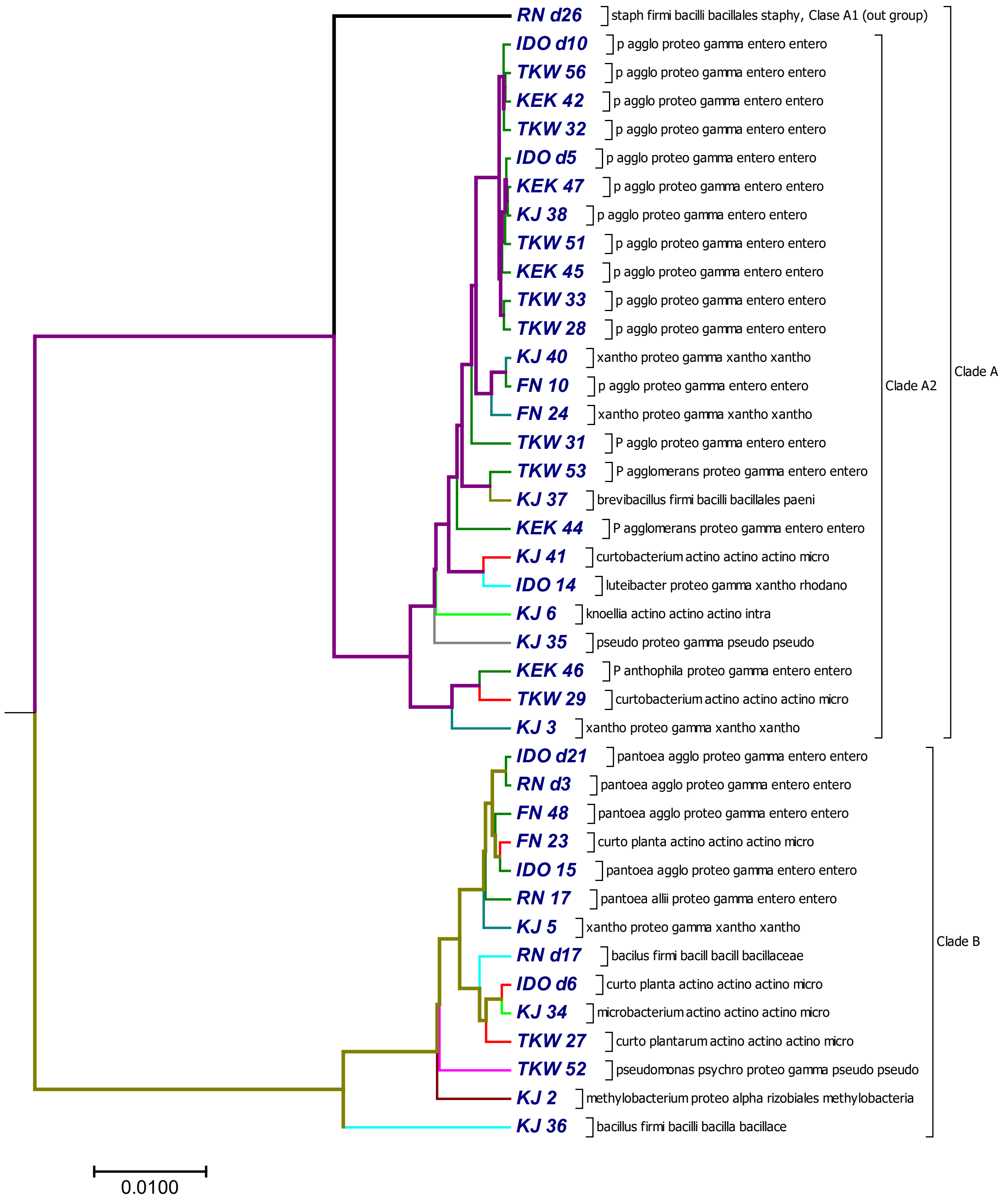

Figure 55: Our method.

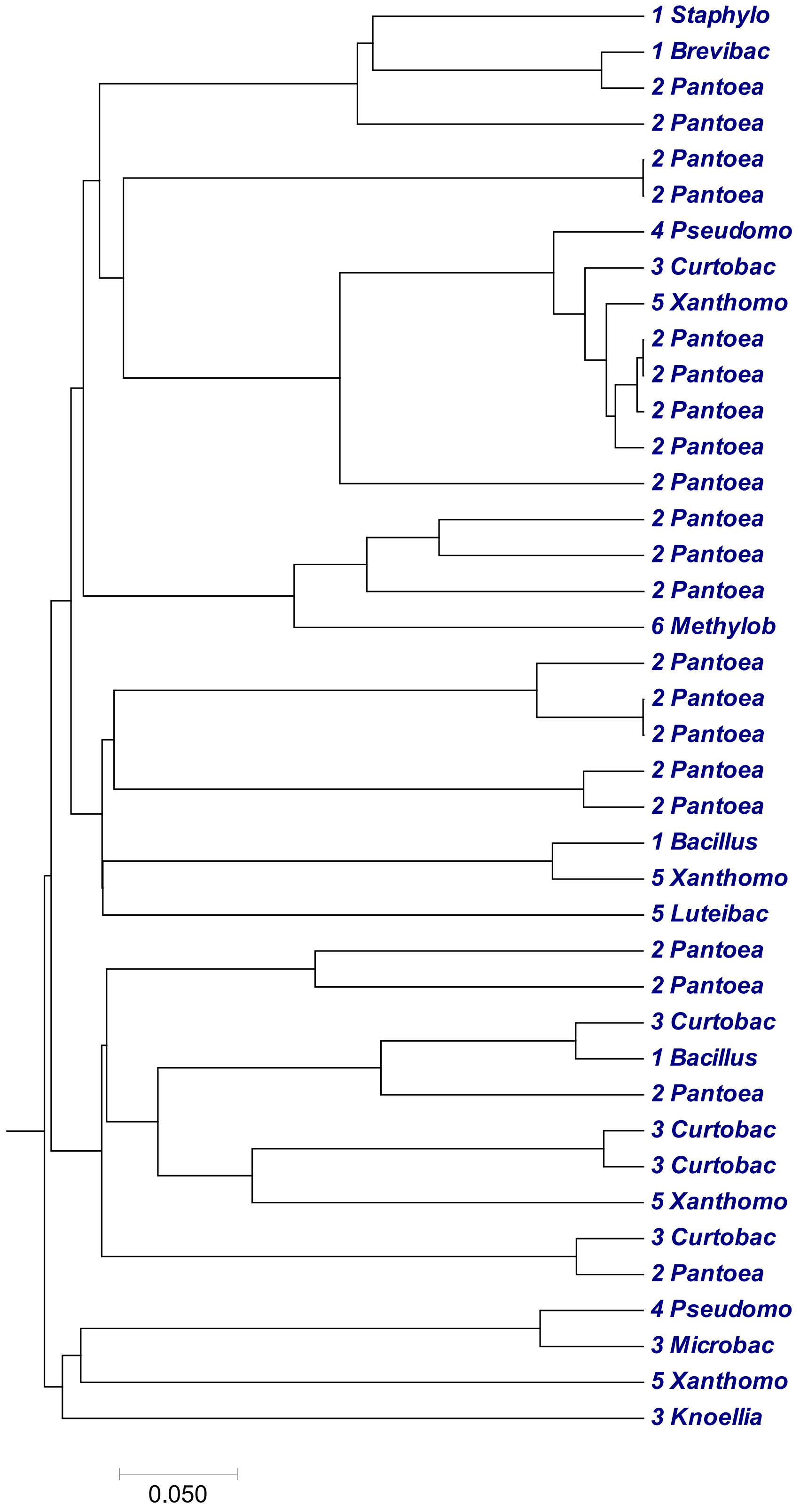

Figure 56: CV method using string length 3.

1 →Bacillales, 2→Enterobacteriales, 3→Actinomycetales, 4→Pseudomonadales, 5→Xanthomonadales, 6→Rhizobiales.

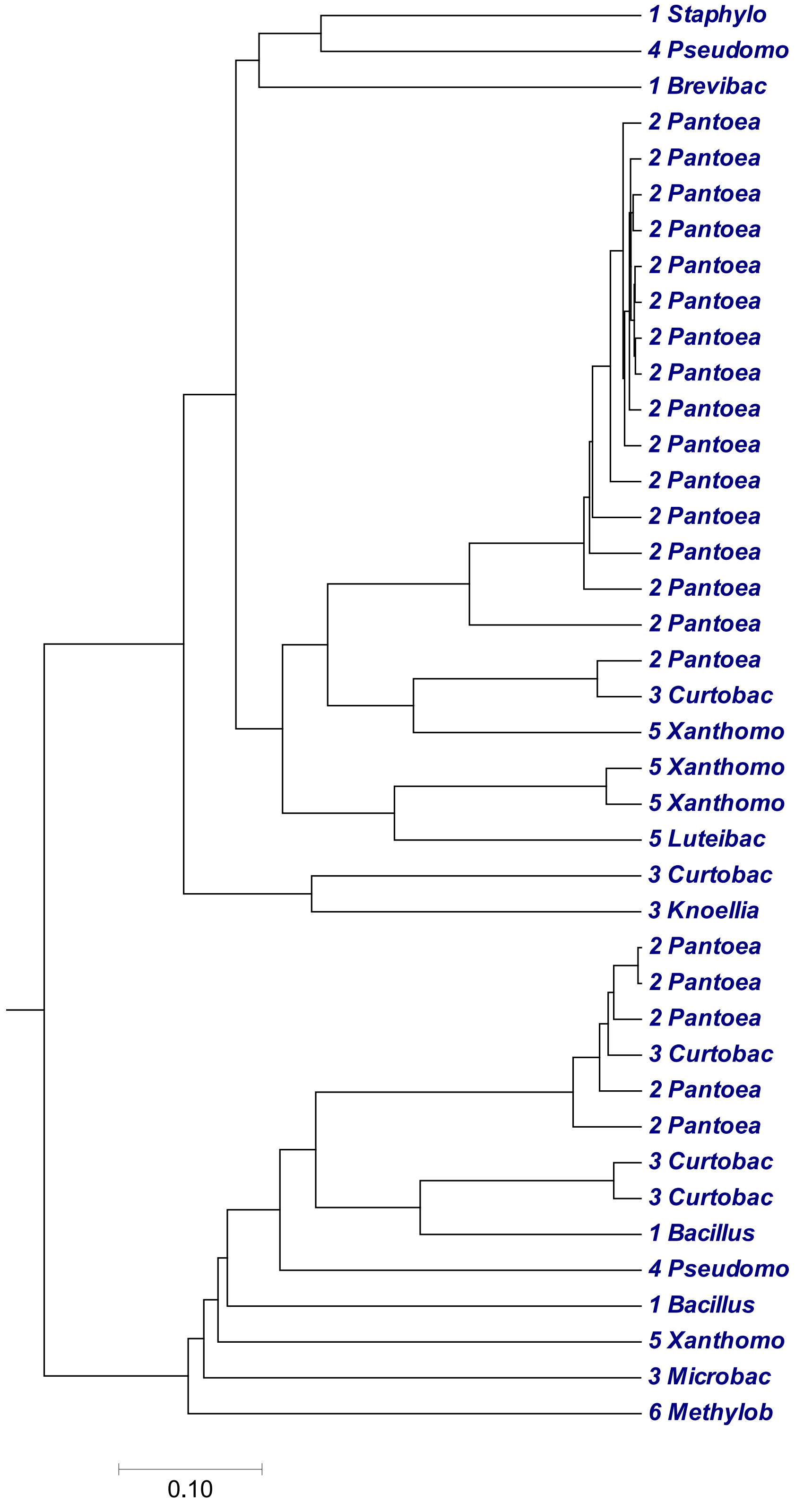

Figure 57: FFP method using string length 7.

1 →Bacillales, 2→Enterobacteriales, 3→Actinomycetales, 4→Pseudomonadales, 5→Xanthomonadales, 6→Rhizobiales.

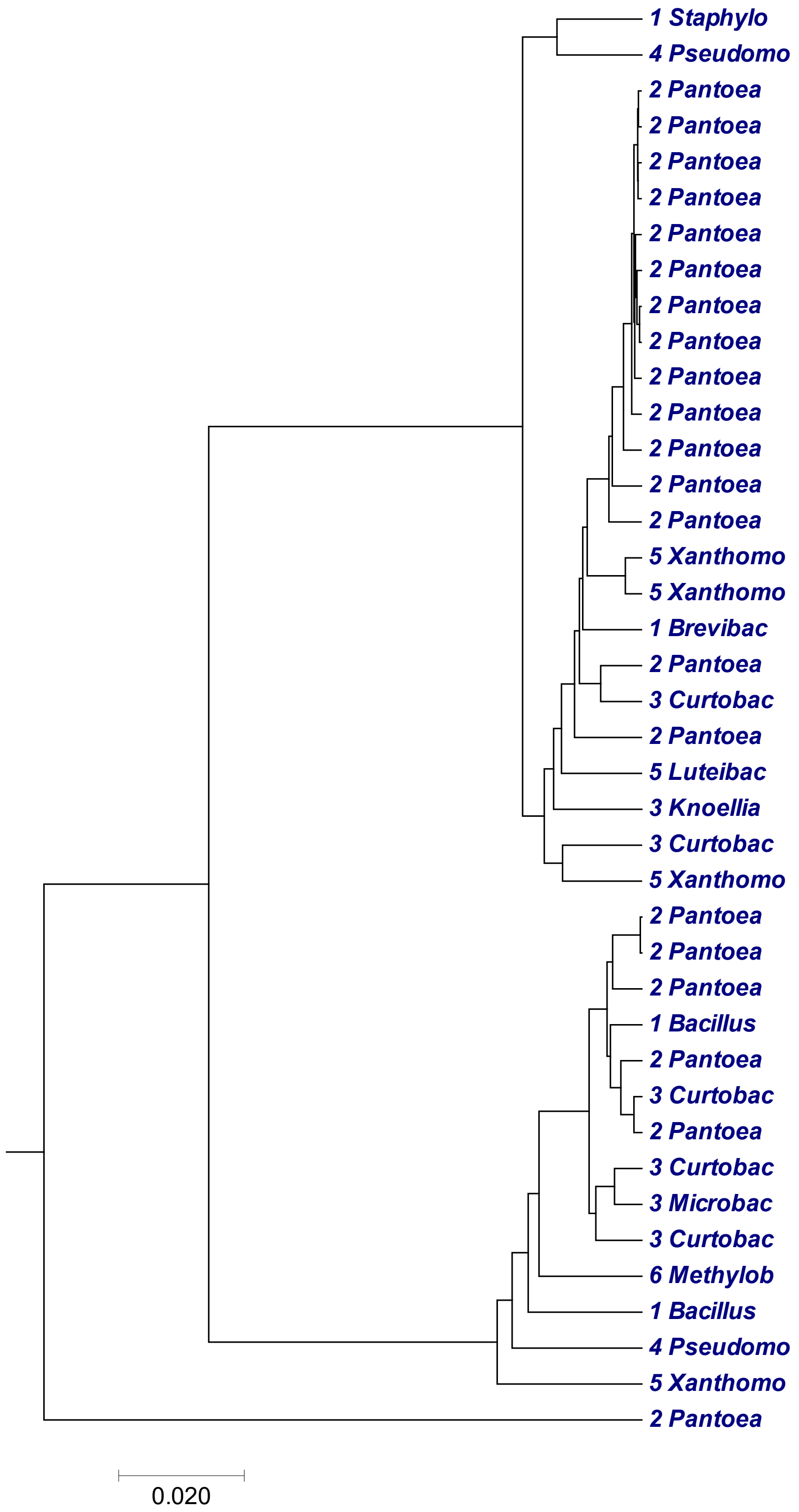

Figure 58: RTD method using string length 1.

1 →Bacillales, 2→Enterobacteriales, 3→Actinomycetales, 4→Pseudomonadales, 5→Xanthomonadales, 6→Rhizobiales.

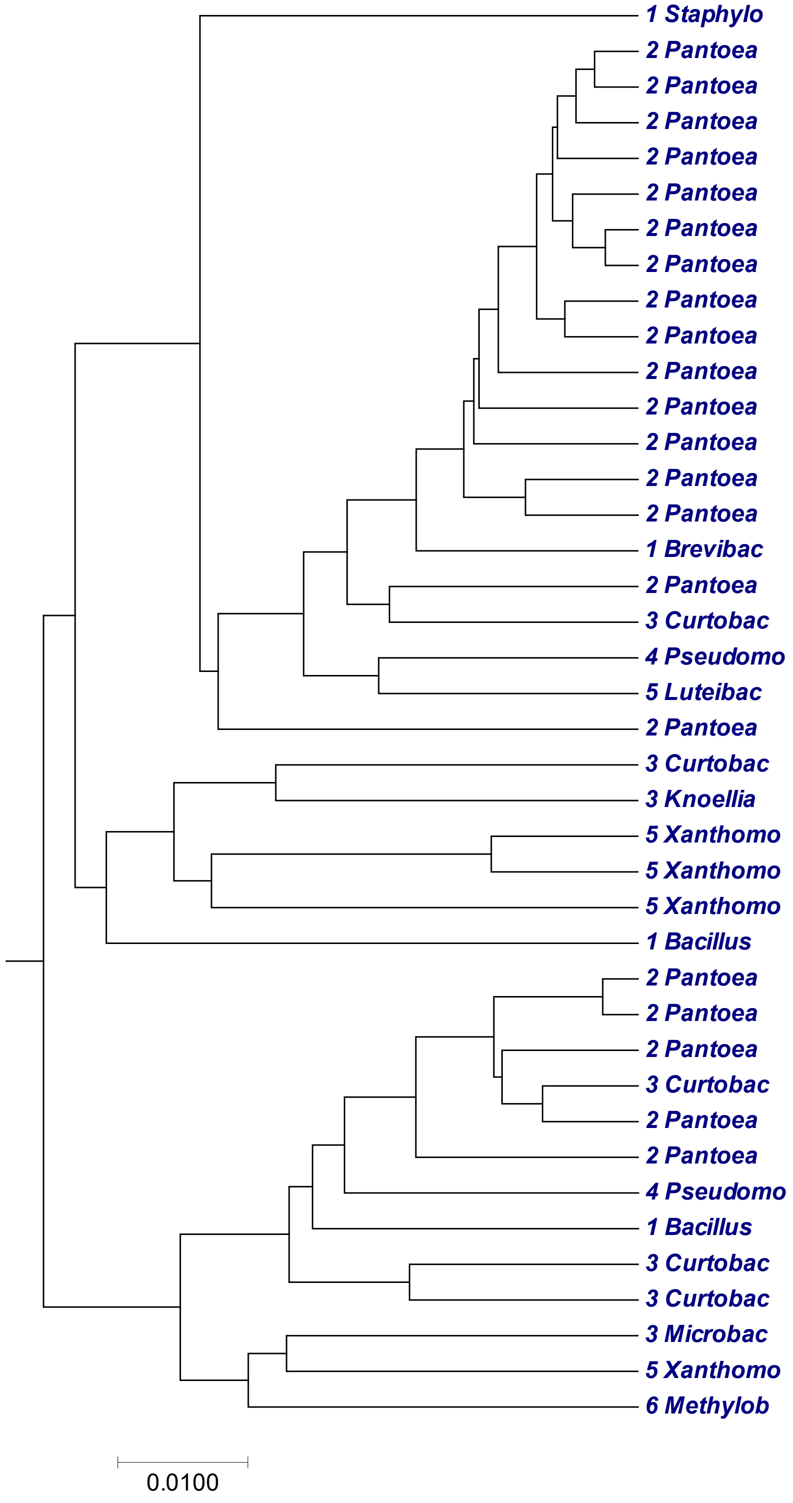

Figure 59: BBC method.

1 →Bacillales, 2→Enterobacteriales, 3→Actinomycetales, 4→Pseudomonadales, 5→Xanthomonadales, 6→Rhizobiales.

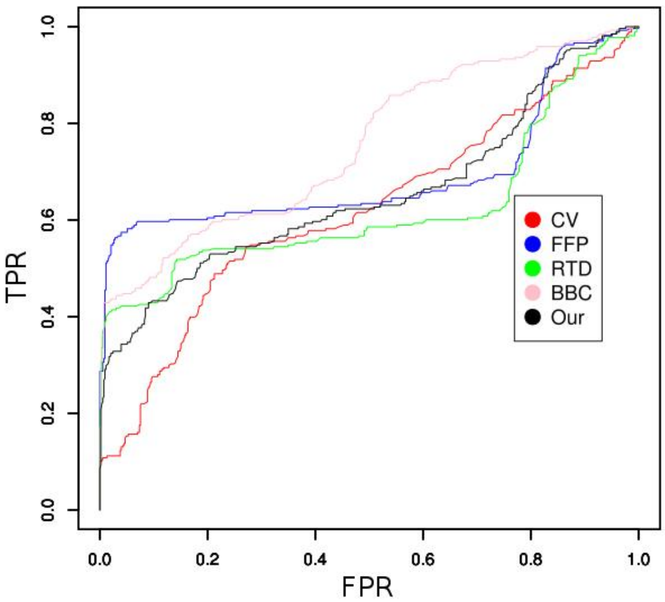

**Dataset→**	**40 bacterial isolates**	
**Methods↓**	**AUC↓**	**Running time↓**
CV method	0.618372	<1s
FFP method	0.692213	<1s
RTD method	0.624117	<1s
BBC method	0.756011	<1s
Our method	0.653343	<1s

Figure 60: Receiver operating characteristic curve (ROC) and Area under the ROC Curve on 40 bacterial isolates sequences.

Our 40 bacterial sequences belong to 11 genera and 16 species. We compared our method with four different techniques and observed little variation in their clustering pattern (Fig. 55-60) except CV method. The 40 bacterial species for AUC were numbered at order level and all the methods except CV, clustered order Enterobacteriales alike. AUC values from the five approaches range from 0.618 to 0.756 with the lowest for method CV and highest for BBC. Although BBC method has the highest AUC value but its clustering pattern to similar to our method (AUC=0.653).

Reference:

1. Zielezinski, A., Vinga, S., Almeida, J. & Karlowski, W. M. Alignment-free sequence comparison: benefits, applications, and tools. Genome Biol. 18, 186 (2017).
2. Swets, J. Measuring the accuracy of diagnostic systems. Sci. 240, 1285–1293 (1988).
3. NEMES, S. & HARTEL, T. Summary measures for binary classification systems in animal ecology. North-Western J. Zool. 6, 323–330 (2010).
4. Siriussawakul, A. et al. Predictive performance of a multivariable difficult intubation model for obese patients. PLOS ONE 1–15 (2018).
5. Antognoli, M. C. et al. Analysis of the diagnostic accuracy of the gamma interferon assay for detection of bovine tuberculosis in u.s. herds. Prev. Vet. Medicine 101, 35 – 41 (2011).
6. Zhu, W., Zeng, N. & Wang, N. Sensitivity, specificity, accuracy, associated confidence interval and roc analysis with practical SAS implementations. in Proceedings of the NESUG Health Care and Life Sciences, Baltimore, Md, USA (2010).
7. Felsenstein, J. Phylip–phylogeny inference package(version 3.2). Cladistics 5 164–166 (1989).
8. Sonego, P., Kocsor, A. & Pongor, S. Roc analysis: applications to the classification of biological sequences and 3d structures. Briefings Bioinforma. 9, 198–209 (2008).
