# Supplementary material for: Alignment-free method for DNA sequence clustering using Fuzzy integral similarity
Source: Sci Rep. 2019 Mar 6;9:3753. doi: 10.1038/s41598-019-40452-6 (PMC6403383; doi:10.1038/s41598-019-40452-6)
Supplement: Supplementary file 4 — Dataset 4 [file 41598_2019_40452_MOESM4_ESM.pdf]

Alignment-free method for DNA sequence clustering using Fuzzy integral similarity

Ajay Kumar Saw1, Garima Raj2, Manashi Das3, Narayan C. Talukdar4, Binod Chandra Tripathy5 and Soumyadeep Nandi6,*
1 Mathematical Sciences Division, Institute of Advanced Study in Science and Technology, Guwahati-35, Assam, India.
2,3,4,6 Life Science Division, Institute of Advanced Study in Science and Technology, Guwahati-35, Assam, India.
5 Mathematics Department, Tripura University, Tripura, India.
* To whom correspondence should be addressed. Email: soumyadeep.nandi@gmail.com; snandi@iasst.gov.in

Distance matrices of datasets

11 arbuscular mycorrhizal fungi (AMF) isolates

```
    11
Human_____     0.000000  0.023016  0.020521  0.061930  0.046802  0.053064  0.039552  0.037216  0.057556  0.032068  0.131337
Chimpanzee     0.023016  0.000000  0.008740  0.069335  0.054207  0.060469  0.046851  0.044262  0.064961  0.044645  0.130692
Gorilla___     0.020521  0.008740  0.000000  0.065996  0.050868  0.057130  0.043512  0.040923  0.061622  0.042150  0.121952
Lemur_____     0.061930  0.069335  0.065996  0.000000  0.017649  0.010406  0.042528  0.041782  0.010308  0.032156  0.128092
Rat_______     0.046802  0.054207  0.050868  0.017649  0.000000  0.007243  0.024879  0.024133  0.010754  0.027603  0.110443
Mouse_____     0.053064  0.060469  0.057130  0.010406  0.007243  0.000000  0.032122  0.031376  0.004492  0.029276  0.117686
Goat______     0.039552  0.046851  0.043512  0.042528  0.024879  0.032122  0.000000  0.002588  0.032220  0.043172  0.091785
Bovine____     0.037216  0.044262  0.040923  0.041782  0.024133  0.031376  0.002588  0.000000  0.031474  0.040836  0.094121
Rabbit____     0.057556  0.064961  0.061622  0.010308  0.010754  0.004492  0.032220  0.031474  0.000000  0.032787  0.117784
Opossum___     0.032068  0.044645  0.042150  0.032156  0.027603  0.029276  0.043172  0.040836  0.032787  0.000000  0.134957
Gallus____     0.131337  0.130692  0.121952  0.128092  0.110443  0.117686  0.091785  0.094121  0.117784  0.134957  0.000000
```

16S rDNA sequences from 40 bacterial isolates

```
    40
RN_d26      0.000000  0.104671  0.095211  0.030200  0.097044  0.030632  0.103956  0.029261  0.031093  0.093341  0.031258  0.100615  0.030517  0.044380  0.031528  0.031078  0.029591  0.035560  0.024883  0.030405  0.027638  0.069923  0.018017  0.095567  0.030898  0.029243  0.031251  0.043894  0.031969  0.092720  0.027435  0.103160  0.107309  0.102568  0.036746  0.025165  0.028148  0.103100  0.044800  0.097353
IDO_d21     0.104671  0.000000  0.009460  0.084581  0.007627  0.085105  0.000717  0.084648  0.081583  0.013225  0.085540  0.004056  0.085567  0.099211  0.085900  0.078991  0.084622  0.090391  0.086343  0.085961  0.078198  0.034747  0.092974  0.009103  0.086336  0.085560  0.086201  0.098725  0.087328  0.011951  0.088322  0.001801  0.002845  0.003567  0.091577  0.085629  0.084992  0.003593  0.103556  0.014673
RN_d17      0.095211  0.009460  0.000000  0.081913  0.005344  0.082684  0.008745  0.081930  0.079421  0.010522  0.083378  0.005404  0.082637  0.097049  0.082880  0.072512  0.082135  0.088229  0.077027  0.082548  0.076036  0.025287  0.083514  0.004287  0.083567  0.081912  0.077533  0.096563  0.084638  0.006747  0.080104  0.007949  0.012098  0.007357  0.089415  0.076559  0.077351  0.007889  0.097469  0.013247
IDO_d10     0.030200  0.084581  0.081913  0.000000  0.081043  0.000771  0.084087  0.001397  0.006838  0.092435  0.001465  0.080525  0.000986  0.015136  0.001681  0.009401  0.000790  0.011578  0.005317  0.001380  0.007548  0.070319  0.013144  0.080439  0.001755  0.001158  0.007051  0.014650  0.002747  0.080229  0.006076  0.083070  0.087219  0.082478  0.007502  0.005354  0.013988  0.085649  0.018975  0.081135
```

```
IDO_d6      0.097044  0.007627  0.005344  0.081043  0.000000  0.081814  0.006912  0.081060  0.078551  0.011392  0.
082508  0.005617  0.081767  0.096179  0.082010  0.071642  0.081265  0.087359  0.078716  0.081678  0.075166  0.
027120  0.085347  0.001476  0.082697  0.081042  0.078574  0.095693  0.083768  0.004324  0.080695  0.008260  0.
010265  0.008239  0.088545  0.078002  0.077365  0.006056  0.096599  0.007903
IDO_d5      0.030632  0.085105  0.082684  0.000771  0.081814  0.000000  0.084858  0.001829  0.007362  0.093206  0.
000694  0.081049  0.000462  0.014365  0.001494  0.010172  0.001041  0.011765  0.005749  0.000856  0.008072  0.
071090  0.013576  0.081210  0.001231  0.001590  0.006864  0.013879  0.002223  0.081000  0.006508  0.083594  0.
087743  0.083002  0.007139  0.006125  0.014420  0.086420  0.018451  0.081906
RN_d3       0.103956  0.000717  0.008745  0.084087  0.006912  0.084858  0.000000  0.084104  0.081595  0.012508  0.
085552  0.003969  0.084852  0.099223  0.085185  0.078276  0.084309  0.090403  0.085628  0.085246  0.078210  0.
034032  0.092259  0.008388  0.085741  0.084845  0.085486  0.098737  0.086812  0.011236  0.087607  0.001990  0.
003562  0.003579  0.091589  0.084914  0.084277  0.002876  0.102841  0.013956
TKW_56      0.029261  0.084648  0.081930  0.001397  0.081060  0.001829  0.084104  0.000000  0.006905  0.092452  0.
002455  0.080592  0.001927  0.015119  0.003027  0.009418  0.000788  0.010232  0.004902  0.002298  0.007615  0.
070336  0.011747  0.080456  0.001949  0.001169  0.008397  0.014633  0.002708  0.080246  0.004679  0.083137  0.
087286  0.082545  0.007485  0.005371  0.012591  0.085666  0.018908  0.081152
TKW_53      0.031093  0.081583  0.079421  0.006838  0.078551  0.007362  0.081595  0.006905  0.000000  0.089943  0.
007078  0.077626  0.007824  0.018184  0.008157  0.008142  0.006879  0.008808  0.008600  0.008218  0.003538  0.
067827  0.015231  0.077947  0.008593  0.007817  0.010504  0.017142  0.009585  0.077737  0.010579  0.079782  0.
080381  0.078016  0.009994  0.007886  0.014881  0.083157  0.025813  0.078643
TKW_52      0.093341  0.013225  0.010522  0.092435  0.011392  0.093206  0.012508  0.092452  0.089943  0.000000  0.
093900  0.012317  0.093159  0.107571  0.093402  0.083034  0.092657  0.098751  0.087549  0.093070  0.086558  0.
023417  0.081644  0.011996  0.094089  0.092434  0.088055  0.107085  0.095160  0.012206  0.090626  0.014498  0.
016070  0.014477  0.099937  0.087081  0.087873  0.009759  0.107991  0.016761
TKW_51      0.031258  0.085540  0.083378  0.001465  0.082508  0.000694  0.085552  0.002455  0.007078  0.093900  0.
000000  0.081583  0.000746  0.013671  0.001846  0.010866  0.001667  0.011413  0.006375  0.001140  0.007788  0.
071784  0.014202  0.081904  0.001515  0.002216  0.007216  0.013185  0.002507  0.081694  0.007134  0.083739  0.
087459  0.082718  0.006787  0.006819  0.015046  0.087114  0.018735  0.082600
FN_48       0.100615  0.004056  0.005404  0.080525  0.005617  0.081049  0.003969  0.080592  0.077626  0.012317  0.
081583  0.000000  0.081511  0.095254  0.081844  0.074935  0.080566  0.086434  0.082287  0.081905  0.074241  0.
030691  0.088918  0.005433  0.082280  0.081504  0.082145  0.094768  0.083272  0.009723  0.084266  0.002643  0.
006694  0.002622  0.087620  0.081573  0.080936  0.005531  0.099500  0.013303
KEK_47      0.030517  0.085567  0.082637  0.000986  0.081767  0.000462  0.084852  0.001927  0.007824  0.093159  0.
000746  0.081511  0.000000  0.014412  0.001100  0.010125  0.001371  0.012159  0.005634  0.000394  0.008534  0.
071043  0.013461  0.081163  0.000930  0.001475  0.006470  0.013926  0.002001  0.080953  0.006393  0.084056  0.
088205  0.083464  0.007533  0.006078  0.014305  0.086373  0.017989  0.081859
KEK_46      0.044380  0.099211  0.097049  0.015136  0.096179  0.014365  0.099223  0.015119  0.018184  0.107571  0.
013671  0.095254  0.014412  0.000000  0.014169  0.024537  0.014914  0.013465  0.020021  0.014501  0.021013  0.
085455  0.026363  0.095575  0.013482  0.015137  0.019516  0.005433  0.012411  0.095365  0.016945  0.097410  0.
098565  0.095644  0.008966  0.020490  0.019698  0.100785  0.007629  0.096271
KEK_45      0.031528  0.085900  0.082880  0.001681  0.082010  0.001494  0.085185  0.003027  0.008157  0.093402  0.
001846  0.081844  0.001100  0.014169  0.000000  0.010368  0.002471  0.013259  0.006645  0.001123  0.008867  0.
071286  0.014472  0.081406  0.001078  0.002486  0.005370  0.013683  0.001758  0.081196  0.007404  0.084389  0.
088538  0.083797  0.008633  0.006363  0.015316  0.086616  0.017656  0.082102
KEK_44      0.031078  0.078991  0.072512  0.009401  0.071642  0.010172  0.078276  0.009418  0.008142  0.083034  0.
010866  0.074935  0.010125  0.024537  0.010368  0.000000  0.009623  0.016267  0.007352  0.010036  0.009005  0.
060918  0.014022  0.071038  0.011055  0.009400  0.007210  0.024051  0.012126  0.070828  0.009331  0.077480  0.
081629  0.076888  0.016903  0.006638  0.014866  0.077420  0.024957  0.071734
KEK_42      0.029591  0.084622  0.082135  0.000790  0.081265  0.001041  0.084309  0.000788  0.006879  0.092657  0.
001667  0.080566  0.001371  0.014914  0.002471  0.009623  0.000000  0.010788  0.005107  0.001742  0.007589  0.
070541  0.012535  0.080661  0.001714  0.000938  0.007841  0.014428  0.002706  0.080451  0.005467  0.083111  0.
087260  0.082519  0.007280  0.005576  0.013379  0.085871  0.018934  0.081357
KJ_41       0.035560  0.090391  0.088229  0.011578  0.087359  0.011765  0.090403  0.010232  0.008808  0.098751  0.
011413  0.086434  0.012159  0.013465  0.013259  0.016267  0.010788  0.000000  0.012909  0.012530  0.012193  0.
076635  0.018055  0.086755  0.012181  0.011401  0.018629  0.017373  0.011780  0.086545  0.011053  0.088590  0.
088474  0.086824  0.004687  0.012946  0.010878  0.091965  0.017720  0.087451
KJ_40       0.024883  0.086343  0.077027  0.005317  0.078716  0.005749  0.085628  0.004902  0.008600  0.087549  0.
006375  0.082287  0.005634  0.020021  0.006645  0.007352  0.005107  0.012909  0.000000  0.005522  0.009310  0.
065433  0.007827  0.077239  0.006539  0.004884  0.006368  0.019535  0.007610  0.075343  0.003076  0.084832  0.
088981  0.084240  0.012387  0.000714  0.010345  0.084772  0.020441  0.079025
KJ_38       0.030405  0.085961  0.082548  0.001380  0.081678  0.000856  0.085246  0.002298  0.008218  0.093070  0.
001140  0.081905  0.000394  0.014501  0.001123  0.010036  0.001742  0.012530  0.005522  0.000000  0.008928  0.
```

```
070954  0.013349  0.081074  0.001019  0.001363  0.006099  0.014015  0.002090  0.080864  0.006281  0.084450  0.
088599  0.083858  0.007904  0.005989  0.014193  0.086284  0.017595  0.081770
KJ_37       0.027638  0.078198  0.076036  0.007548  0.075166  0.008072  0.078210  0.007615  0.003538  0.086558  0.
007788  0.074241  0.008534  0.021013  0.008867  0.009005  0.007589  0.012193  0.009310  0.008928  0.000000  0.
064442  0.015941  0.074562  0.009303  0.008527  0.011367  0.020527  0.010295  0.074352  0.011289  0.076397  0.
079671  0.074930  0.013379  0.008596  0.011343  0.079772  0.026523  0.075258
KJ_36       0.069923  0.034747  0.025287  0.070319  0.027120  0.071090  0.034032  0.070336  0.067827  0.023417  0.
071784  0.030691  0.071043  0.085455  0.071286  0.060918  0.070541  0.076635  0.065433  0.070954  0.064442  0.
000000  0.059092  0.025644  0.071973  0.070318  0.065939  0.084969  0.073044  0.022796  0.068510  0.033236  0.
037385  0.032644  0.077821  0.064965  0.065757  0.033176  0.085875  0.027429
KJ_35       0.018017  0.092974  0.083514  0.013144  0.085347  0.013576  0.092259  0.011747  0.015231  0.081644  0.
014202  0.088918  0.013461  0.026363  0.014472  0.014022  0.012535  0.018055  0.007827  0.013349  0.015941  0.
059092  0.000000  0.083870  0.013645  0.011986  0.014195  0.025877  0.013952  0.081023  0.009418  0.091463  0.
095612  0.090871  0.018729  0.008109  0.015491  0.091403  0.026783  0.085656
KJ_34       0.095567  0.009103  0.004287  0.080439  0.001476  0.081210  0.008388  0.080456  0.077947  0.011996  0.
081904  0.005433  0.081163  0.095575  0.081406  0.071038  0.080661  0.086755  0.077239  0.081074  0.074562  0.
025644  0.083870  0.000000  0.082093  0.080438  0.077097  0.095089  0.083164  0.004290  0.079218  0.008076  0.
011741  0.008055  0.087941  0.076525  0.075888  0.007532  0.095995  0.008959
TKW_33      0.030898  0.086336  0.083567  0.001755  0.082697  0.001231  0.085741  0.001949  0.008593  0.094089  0.
001515  0.082280  0.000930  0.013482  0.001078  0.011055  0.001714  0.012181  0.006539  0.001019  0.009303  0.
071973  0.013645  0.082093  0.000000  0.001659  0.006448  0.012996  0.001071  0.081883  0.006577  0.084825  0.
088974  0.084233  0.007555  0.007008  0.014489  0.087303  0.017220  0.082789
TKW_32      0.029243  0.085560  0.081912  0.001158  0.081042  0.001590  0.084845  0.001169  0.007817  0.092434  0.
002216  0.081504  0.001475  0.015137  0.002486  0.009400  0.000938  0.011401  0.004884  0.001363  0.008527  0.
070318  0.011986  0.080438  0.001659  0.000000  0.007227  0.014651  0.002726  0.080228  0.004918  0.084049  0.
088198  0.083457  0.007503  0.005353  0.012830  0.085648  0.017996  0.081134
TKW_31      0.031251  0.086201  0.077533  0.007051  0.078574  0.006864  0.085486  0.008397  0.010504  0.088055  0.
007216  0.082145  0.006470  0.019516  0.005370  0.007210  0.007841  0.018629  0.006368  0.006099  0.011367  0.
065939  0.014195  0.077097  0.006448  0.007227  0.000000  0.019030  0.007105  0.075849  0.007576  0.084690  0.
088839  0.084098  0.014003  0.006086  0.016065  0.084630  0.019936  0.078883
TKW_29      0.043894  0.098725  0.096563  0.014650  0.095693  0.013879  0.098737  0.014633  0.017142  0.107085  0.
013185  0.094768  0.013926  0.005433  0.013683  0.024051  0.014428  0.017373  0.019535  0.014015  0.020527  0.
084969  0.025877  0.095089  0.012996  0.014651  0.019030  0.000000  0.011925  0.094879  0.016931  0.096924  0.
096362  0.095158  0.012686  0.020004  0.024843  0.100299  0.013062  0.095785
TKW_28      0.031969  0.087328  0.084638  0.002747  0.083768  0.002223  0.086812  0.002708  0.009585  0.095160  0.
002507  0.083272  0.002001  0.012411  0.001758  0.012126  0.002706  0.011780  0.007610  0.002090  0.010295  0.
073044  0.013952  0.083164  0.001071  0.002726  0.007105  0.011925  0.000000  0.082954  0.006256  0.085817  0.
089966  0.085225  0.007154  0.008079  0.014168  0.088374  0.016228  0.083860
TKW_27      0.092720  0.011951  0.006747  0.080229  0.004324  0.081000  0.011236  0.080246  0.077737  0.012206  0.
081694  0.009723  0.080953  0.095365  0.081196  0.070828  0.080451  0.086545  0.075343  0.080864  0.074352  0.
022796  0.081023  0.004290  0.081883  0.080228  0.075849  0.094879  0.082954  0.000000  0.078420  0.012366  0.
014589  0.012345  0.087731  0.074875  0.075667  0.010380  0.095785  0.007307
FN_24       0.027435  0.088322  0.080104  0.006076  0.080695  0.006508  0.087607  0.004679  0.010579  0.090626  0.
007134  0.084266  0.006393  0.016945  0.007404  0.009331  0.005467  0.011053  0.003076  0.006281  0.011289  0.
068510  0.009418  0.079218  0.006577  0.004918  0.007576  0.016931  0.006256  0.078420  0.000000  0.086811  0.
090960  0.086219  0.009311  0.003545  0.008489  0.086751  0.017365  0.081004
FN_23       0.103160  0.001801  0.007949  0.083070  0.008260  0.083594  0.001990  0.083137  0.079782  0.014498  0.
083739  0.002643  0.084056  0.097410  0.084389  0.077480  0.083111  0.088590  0.084832  0.084450  0.076397  0.
033236  0.091463  0.008076  0.084825  0.084049  0.084690  0.096924  0.085817  0.012366  0.086811  0.000000  0.
004149  0.001766  0.089776  0.084118  0.083481  0.004866  0.102045  0.015946
RN_17       0.107309  0.002845  0.012098  0.087219  0.010265  0.087743  0.003562  0.087286  0.080381  0.016070  0.
087459  0.006694  0.088205  0.098565  0.088538  0.081629  0.087260  0.088474  0.088981  0.088599  0.079671  0.
037385  0.095612  0.011741  0.088974  0.088198  0.088839  0.096362  0.089966  0.014589  0.090960  0.004149  0.
000000  0.004741  0.089599  0.088267  0.087630  0.006438  0.106194  0.017518
IDO_15      0.102568  0.003567  0.007357  0.082478  0.008239  0.083002  0.003579  0.082545  0.078016  0.014477  0.
082718  0.002622  0.083464  0.095644  0.083797  0.076888  0.082519  0.086824  0.084240  0.083858  0.074930  0.
032644  0.090871  0.008055  0.084233  0.083457  0.084098  0.095158  0.085225  0.012345  0.086219  0.001766  0.
004741  0.000000  0.088010  0.083526  0.082889  0.005141  0.101453  0.015925
IDO_14      0.036746  0.091577  0.089415  0.007502  0.088545  0.007139  0.091589  0.007485  0.009994  0.099937  0.
006787  0.087620  0.007533  0.008966  0.008633  0.016903  0.007280  0.004687  0.012387  0.007904  0.013379  0.
077821  0.018729  0.087941  0.007555  0.007503  0.014003  0.012686  0.007154  0.087731  0.009311  0.089776  0.
089599  0.088010  0.000000  0.012856  0.012157  0.093151  0.016595  0.088637
```

```
FN_10       0.025165  0.085629  0.076559  0.005354  0.078002  0.006125  0.084914  0.005371  0.007886  0.087081  0.
006819  0.081573  0.006078  0.020490  0.006363  0.006638  0.005576  0.012946  0.000714  0.005989  0.008596  0.
064965  0.008109  0.076525  0.007008  0.005353  0.006086  0.020004  0.008079  0.074875  0.003545  0.084118  0.
088267  0.083526  0.012856  0.000000  0.010382  0.084058  0.020910  0.078311
KJ_6        0.028148  0.084992  0.077351  0.013988  0.077365  0.014420  0.084277  0.012591  0.014881  0.087873  0.
015046  0.080936  0.014305  0.019698  0.015316  0.014866  0.013379  0.010878  0.010345  0.014193  0.011343  0.
065757  0.015491  0.075888  0.014489  0.012830  0.016065  0.024843  0.014168  0.075667  0.008489  0.083481  0.
087630  0.082889  0.012157  0.010382  0.000000  0.083421  0.020118  0.077674
KJ_5        0.103100  0.003593  0.007889  0.085649  0.006056  0.086420  0.002876  0.085666  0.083157  0.009759  0.
087114  0.005531  0.086373  0.100785  0.086616  0.077420  0.085871  0.091965  0.084772  0.086284  0.079772  0.
033176  0.091403  0.007532  0.087303  0.085648  0.084630  0.100299  0.088374  0.010380  0.086751  0.004866  0.
006438  0.005141  0.093151  0.084058  0.083421  0.000000  0.101985  0.011080
KJ_3        0.044800  0.103556  0.097469  0.018975  0.096599  0.018451  0.102841  0.018908  0.025813  0.107991  0.
018735  0.099500  0.017989  0.007629  0.017656  0.024957  0.018934  0.017720  0.020441  0.017595  0.026523  0.
085875  0.026783  0.095995  0.017220  0.017996  0.019936  0.013062  0.016228  0.095785  0.017365  0.102045  0.
106194  0.101453  0.016595  0.020910  0.020118  0.101985  0.000000  0.096691
KJ_2        0.097353  0.014673  0.013247  0.081135  0.007903  0.081906  0.013956  0.081152  0.078643  0.016761  0.
082600  0.013303  0.081859  0.096271  0.082102  0.071734  0.081357  0.087451  0.079025  0.081770  0.075258  0.
027429  0.085656  0.008959  0.082789  0.081134  0.078883  0.095785  0.083860  0.007307  0.081004  0.015946  0.
017518  0.015925  0.088637  0.078311  0.077674  0.011080  0.096691  0.000000
```

41 mammalian mitochondrial genomes

```
    41
V00662.1_H     0.000000  0.005647  0.004698  0.005499  0.007525  0.006899  0.010812  0.012034  0.015085  0.050534
 0.057299  0.049565  0.054271  0.053199  0.053056  0.049602  0.058088  0.046769  0.046656  0.037272  0.032749  0.
058365  0.056521  0.057004  0.074368  0.046580  0.111624  0.010720  0.067591  0.076702  0.036389  0.031185  0.
048240  0.052870  0.023311  0.039554  0.030523  0.027037  0.035193  0.033313  0.045874
D38116.1_P     0.005647  0.000000  0.001945  0.004966  0.013172  0.004251  0.006713  0.017478  0.020529  0.045090
 0.051855  0.044121  0.048827  0.047755  0.047612  0.044158  0.052644  0.041325  0.041212  0.031828  0.027305  0.
052921  0.051077  0.051560  0.068924  0.041136  0.106180  0.006621  0.062147  0.071258  0.030945  0.025741  0.
042796  0.047426  0.017867  0.034110  0.025079  0.021593  0.029749  0.027869  0.040430
D38113.1_P     0.004698  0.001945  0.000000  0.003145  0.012223  0.003105  0.008658  0.016404  0.019455  0.046163
 0.052928  0.045194  0.049900  0.048828  0.048685  0.045231  0.053717  0.042398  0.042285  0.032901  0.028378  0.
053994  0.052150  0.052633  0.069997  0.042209  0.107254  0.008566  0.063220  0.072331  0.032018  0.026814  0.
043869  0.048499  0.018940  0.035183  0.026152  0.022666  0.030822  0.028942  0.041503
D38114.1_G     0.005499  0.004966  0.003145  0.000000  0.012396  0.005295  0.010848  0.017533  0.020584  0.045035
 0.051800  0.044066  0.048772  0.047700  0.047557  0.044103  0.052589  0.041270  0.041157  0.031773  0.027250  0.
052866  0.051022  0.051505  0.068869  0.041081  0.106125  0.010756  0.062092  0.071203  0.030890  0.025686  0.
042741  0.047371  0.017812  0.034055  0.025024  0.021538  0.029694  0.027814  0.040375
X99256.1_H     0.007525  0.013172  0.012223  0.012396  0.000000  0.012079  0.015221  0.006854  0.009905  0.055714
 0.062479  0.054745  0.059451  0.058379  0.058236  0.054782  0.063268  0.051949  0.051836  0.042452  0.037929  0.
063545  0.061701  0.062184  0.079548  0.051760  0.116804  0.014486  0.072771  0.081882  0.041569  0.036365  0.
053420  0.058050  0.028491  0.044734  0.035703  0.032217  0.040373  0.038493  0.051054
Y18001.1_P     0.006899  0.004251  0.003105  0.005295  0.012079  0.000000  0.006212  0.018933  0.021984  0.043634
 0.050399  0.042665  0.047371  0.046299  0.046156  0.042702  0.051188  0.039869  0.039756  0.030372  0.025849  0.
051465  0.049621  0.050104  0.067468  0.039680  0.104724  0.005461  0.060691  0.069802  0.029489  0.024285  0.
041340  0.045970  0.016411  0.032654  0.023623  0.020137  0.028293  0.026413  0.038974
AY863426.1     0.010812  0.006713  0.008658  0.010848  0.015221  0.006212  0.000000  0.022075  0.025126  0.040493
 0.047258  0.039524  0.044230  0.043158  0.043015  0.039561  0.048047  0.036728  0.036615  0.027231  0.022708  0.
048324  0.046480  0.046963  0.064327  0.036539  0.101583  0.003149  0.057550  0.066661  0.026348  0.021144  0.
038199  0.042829  0.013270  0.029513  0.020482  0.016996  0.025152  0.023272  0.035833
D38115.1_P     0.012034  0.017478  0.016404  0.017533  0.006854  0.018933  0.022075  0.000000  0.003051  0.062568
 0.069333  0.061599  0.066305  0.065233  0.065090  0.061636  0.070122  0.058803  0.058690  0.049306  0.044783  0.
070399  0.068555  0.069038  0.086402  0.058614  0.123658  0.021340  0.079625  0.088736  0.048423  0.043219  0.
060274  0.064904  0.035345  0.051588  0.042557  0.039071  0.047227  0.045347  0.057908
NC_002083.     0.015085  0.020529  0.019455  0.020584  0.009905  0.021984  0.025126  0.003051  0.000000  0.065619
 0.072384  0.064650  0.069356  0.068284  0.068141  0.064687  0.073173  0.061854  0.061741  0.052357  0.047834  0.
073450  0.071606  0.072089  0.089453  0.061665  0.126709  0.024391  0.082676  0.091787  0.051474  0.046270  0.
063325  0.067955  0.038396  0.054639  0.045608  0.042122  0.050278  0.048398  0.060959
```

```
U20753.1_F     0.050534  0.045090  0.046163  0.045035  0.055714  0.043634  0.040493  0.062568  0.065619  0.000000
 0.016405  0.021235  0.010775  0.009902  0.008239  0.005572  0.016204  0.006992  0.007741  0.014825  0.017785  0.
013461  0.016957  0.017197  0.023834  0.012301  0.062905  0.041228  0.037804  0.042845  0.014145  0.019349  0.
010285  0.020183  0.027223  0.010980  0.020011  0.023497  0.016484  0.017220  0.008112
U96639.2_C     0.057299  0.051855  0.052928  0.051800  0.062479  0.050399  0.047258  0.069333  0.072384  0.016405
 0.000000  0.030869  0.020409  0.018421  0.017873  0.019698  0.001607  0.017575  0.016513  0.025455  0.029776  0.
012750  0.015781  0.016749  0.017069  0.010719  0.054325  0.047993  0.021399  0.026440  0.021323  0.026982  0.
019665  0.029817  0.033988  0.019916  0.027695  0.030262  0.022106  0.025271  0.021164
AJ002189.1     0.049565  0.044121  0.045194  0.044066  0.054745  0.042665  0.039524  0.061599  0.064650  0.021235
 0.030869  0.000000  0.016402  0.017024  0.014384  0.015663  0.031485  0.028227  0.028976  0.012293  0.016816  0.
034696  0.038192  0.038432  0.036938  0.032309  0.076149  0.040259  0.051048  0.056089  0.019481  0.020460  0.
011204  0.003882  0.026254  0.020316  0.019679  0.025515  0.017731  0.020197  0.013123
AF010406.1     0.054271  0.048827  0.049900  0.048772  0.059451  0.047371  0.044230  0.066305  0.069356  0.010775
 0.020409  0.016402  0.000000  0.001988  0.003339  0.006985  0.021025  0.017767  0.018516  0.016999  0.021522  0.
024236  0.027732  0.027972  0.020536  0.021849  0.059747  0.044965  0.034646  0.039687  0.017882  0.023086  0.
006031  0.012520  0.030960  0.014717  0.023748  0.027234  0.019078  0.020957  0.008397
AF533441.1     0.053199  0.047755  0.048828  0.047700  0.058379  0.046299  0.043158  0.065233  0.068284  0.009902
 0.018421  0.017024  0.001988  0.000000  0.003045  0.007073  0.019037  0.015779  0.016528  0.015927  0.020450  0.
022646  0.025744  0.026645  0.021169  0.019861  0.059125  0.043893  0.034024  0.039065  0.016810  0.022014  0.
005820  0.013142  0.029888  0.013645  0.022676  0.026162  0.018006  0.019885  0.008539
V00654.1_B     0.053056  0.047612  0.048685  0.047557  0.058236  0.046156  0.043015  0.065090  0.068141  0.008239
 0.017873  0.014384  0.003339  0.003045  0.000000  0.004433  0.018489  0.015231  0.015980  0.015784  0.020307  0.
021700  0.025196  0.025436  0.022554  0.019313  0.061765  0.043750  0.036664  0.041705  0.016667  0.021871  0.
004816  0.011944  0.029745  0.013502  0.022533  0.026019  0.017863  0.019742  0.007182
AY488491.1     0.049602  0.044158  0.045231  0.044103  0.054782  0.042702  0.039561  0.061636  0.064687  0.005572
 0.019698  0.015663  0.006985  0.007073  0.004433  0.000000  0.019497  0.012564  0.013313  0.012330  0.016853  0.
019033  0.022529  0.022769  0.026987  0.016646  0.066198  0.040296  0.041097  0.046138  0.013213  0.018417  0.
007074  0.014611  0.026291  0.010048  0.019079  0.022565  0.014409  0.016288  0.004802
EU442884.2     0.058088  0.052644  0.053717  0.052589  0.063268  0.051188  0.048047  0.070122  0.073173  0.016204
 0.001607  0.031485  0.021025  0.019037  0.018489  0.019497  0.000000  0.017374  0.016312  0.025254  0.029575  0.
011143  0.014174  0.015142  0.016280  0.011508  0.053536  0.048782  0.021600  0.026641  0.021699  0.026903  0.
020281  0.030433  0.034777  0.019715  0.027565  0.031051  0.022895  0.025070  0.020963
EF551003.1     0.046769  0.041325  0.042398  0.041270  0.051949  0.039869  0.036728  0.058803  0.061854  0.006992
 0.017575  0.028227  0.017767  0.015779  0.015231  0.012564  0.017374  0.000000  0.001062  0.019221  0.016990  0.
011596  0.011378  0.012346  0.027599  0.013471  0.064855  0.037463  0.038974  0.044015  0.015377  0.015584  0.
017023  0.027175  0.023458  0.012784  0.016246  0.019732  0.020880  0.013788  0.015104
EF551002.1     0.046656  0.041212  0.042285  0.041157  0.051836  0.039756  0.036615  0.058690  0.061741  0.007741
 0.016513  0.028976  0.018516  0.016528  0.015980  0.013313  0.016312  0.001062  0.000000  0.018796  0.016565  0.
011709  0.011804  0.012772  0.027712  0.012409  0.064968  0.037350  0.037912  0.042953  0.014952  0.015471  0.
017772  0.027924  0.023345  0.012359  0.016133  0.019619  0.020455  0.013363  0.015853
X97336.1_R     0.037272  0.031828  0.032901  0.031773  0.042452  0.030372  0.027231  0.049306  0.052357  0.014825
 0.025455  0.012293  0.016999  0.015927  0.015784  0.012330  0.025254  0.019221  0.018796  0.000000  0.004523  0.
027569  0.030600  0.031568  0.037096  0.021687  0.074352  0.027966  0.046854  0.051895  0.008859  0.009838  0.
010968  0.015598  0.013961  0.009694  0.009057  0.014893  0.007110  0.009575  0.008602
Y07726.1_C     0.032749  0.027305  0.028378  0.027250  0.037929  0.025849  0.022708  0.044783  0.047834  0.017785
 0.029776  0.016816  0.021522  0.020450  0.020307  0.016853  0.029575  0.016990  0.016565  0.004523  0.000000  0.
025616  0.028369  0.029337  0.041619  0.025672  0.078875  0.023443  0.051175  0.056216  0.008453  0.007405  0.
015491  0.020121  0.010494  0.009860  0.006624  0.012460  0.011011  0.007142  0.013125
DQ402478.1     0.058365  0.052921  0.053994  0.052866  0.063545  0.051465  0.048324  0.070399  0.073450  0.013461
 0.012750  0.034696  0.024236  0.022646  0.021700  0.019033  0.011143  0.011596  0.011709  0.027569  0.025616  0.
000000  0.003496  0.003998  0.016003  0.017925  0.054358  0.049059  0.029257  0.034298  0.023725  0.027180  0.
023492  0.033644  0.035054  0.021132  0.027842  0.031328  0.029228  0.025051  0.021573
AF303110.1     0.056521  0.051077  0.052150  0.051022  0.061701  0.049621  0.046480  0.068555  0.071606  0.016957
 0.015781  0.038192  0.027732  0.025744  0.025196  0.022529  0.014174  0.011378  0.011804  0.030600  0.028369  0.
003496  0.000000  0.000968  0.017847  0.020955  0.055736  0.047215  0.031768  0.035676  0.026756  0.025336  0.
026988  0.037140  0.033210  0.024163  0.026051  0.029484  0.032259  0.025167  0.025069
AF303111.1     0.057004  0.051560  0.052633  0.051505  0.062184  0.050104  0.046963  0.069038  0.072089  0.017197
 0.016749  0.038432  0.027972  0.026645  0.025436  0.022769  0.015142  0.012346  0.012772  0.031568  0.029337  0.
003998  0.000968  0.000000  0.017364  0.021923  0.055981  0.047698  0.032736  0.035921  0.027724  0.026289  0.
027228  0.037380  0.033693  0.025131  0.027019  0.029967  0.033227  0.026135  0.025309
EF212882.1     0.074368  0.068924  0.069997  0.068869  0.079548  0.067468  0.064327  0.086402  0.089453  0.023834
 0.017069  0.036938  0.020536  0.021169  0.022554  0.026987  0.016280  0.027599  0.027712  0.037096  0.041619  0.
```

```
016003  0.017847  0.017364  0.000000  0.027788  0.039211  0.065062  0.024440  0.023656  0.037979  0.043183  0.
026128  0.033056  0.051057  0.034814  0.043845  0.047331  0.039175  0.041054  0.028494
AJ001588.1     0.046580  0.041136  0.042209  0.041081  0.051760  0.039680  0.036539  0.058614  0.061665  0.012301
 0.010719  0.032309  0.021849  0.019861  0.019313  0.016646  0.011508  0.013471  0.012409  0.021687  0.025672  0.
017925  0.020955  0.021923  0.027788  0.000000  0.065044  0.037274  0.025502  0.030544  0.017219  0.022878  0.
021105  0.031257  0.027609  0.015812  0.023591  0.024272  0.014661  0.021167  0.019186
X88898.2_E     0.111624  0.106180  0.107254  0.106125  0.116804  0.104724  0.101583  0.123658  0.126709  0.062905
 0.054325  0.076149  0.059747  0.059125  0.061765  0.066198  0.053536  0.064855  0.064968  0.074352  0.078875  0.
054358  0.055736  0.055981  0.039211  0.065044  0.000000  0.102318  0.044033  0.034922  0.075235  0.080439  0.
064945  0.072267  0.088313  0.072070  0.081101  0.084587  0.076431  0.078310  0.067664
NC_002764.     0.010720  0.006621  0.008566  0.010756  0.014486  0.005461  0.003149  0.021340  0.024391  0.041228
 0.047993  0.040259  0.044965  0.043893  0.043750  0.040296  0.048782  0.037463  0.037350  0.027966  0.023443  0.
049059  0.047215  0.047698  0.065062  0.037274  0.102318  0.000000  0.059174  0.067396  0.027083  0.021879  0.
038934  0.043564  0.014005  0.030248  0.021217  0.017731  0.025887  0.024007  0.036568
AJ238588.1     0.067591  0.062147  0.063220  0.062092  0.072771  0.060691  0.057550  0.079625  0.082676  0.037804
 0.021399  0.051048  0.034646  0.034024  0.036664  0.041097  0.021600  0.038974  0.037912  0.046854  0.051175  0.
029257  0.031768  0.032736  0.024440  0.025502  0.044033  0.059174  0.000000  0.009111  0.042722  0.048381  0.
039844  0.047166  0.053112  0.041315  0.049094  0.049775  0.040164  0.046670  0.042563
AJ001562.1     0.076702  0.071258  0.072331  0.071203  0.081882  0.069802  0.066661  0.088736  0.091787  0.042845
 0.026440  0.056089  0.039687  0.039065  0.041705  0.046138  0.026641  0.044015  0.042953  0.051895  0.056216  0.
034298  0.035676  0.035921  0.023656  0.030544  0.034922  0.067396  0.009111  0.000000  0.047763  0.053422  0.
044885  0.052207  0.058153  0.046356  0.054135  0.054816  0.045205  0.051711  0.047604
X72204.1_B     0.036389  0.030945  0.032018  0.030890  0.041569  0.029489  0.026348  0.048423  0.051474  0.014145
 0.021323  0.019481  0.017882  0.016810  0.016667  0.013213  0.021699  0.015377  0.014952  0.008859  0.008453  0.
023725  0.026756  0.027724  0.037979  0.017219  0.075235  0.027083  0.042722  0.047763  0.000000  0.005659  0.
011851  0.018429  0.013078  0.003165  0.006372  0.009352  0.005503  0.003948  0.009485
NC_005268.     0.031185  0.025741  0.026814  0.025686  0.036365  0.024285  0.021144  0.043219  0.046270  0.019349
 0.026982  0.020460  0.023086  0.022014  0.021871  0.018417  0.026903  0.015584  0.015471  0.009838  0.007405  0.
027180  0.025336  0.026289  0.043183  0.022878  0.080439  0.021879  0.048381  0.053422  0.005659  0.000000  0.
017055  0.021685  0.007874  0.008369  0.000781  0.005055  0.008217  0.002128  0.014689
NC_007441.     0.048240  0.042796  0.043869  0.042741  0.053420  0.041340  0.038199  0.060274  0.063325  0.010285
 0.019665  0.011204  0.006031  0.005820  0.004816  0.007074  0.020281  0.017023  0.017772  0.010968  0.015491  0.
023492  0.026988  0.027228  0.026128  0.021105  0.064945  0.038934  0.039844  0.044885  0.011851  0.017055  0.
000000  0.010152  0.024929  0.009112  0.017717  0.021203  0.013047  0.014926  0.002719
NC_008830.     0.052870  0.047426  0.048499  0.047371  0.058050  0.045970  0.042829  0.064904  0.067955  0.020183
 0.029817  0.003882  0.012520  0.013142  0.011944  0.014611  0.030433  0.027175  0.027924  0.015598  0.020121  0.
033644  0.037140  0.037380  0.033056  0.031257  0.072267  0.043564  0.047166  0.052207  0.018429  0.021685  0.
010152  0.000000  0.029559  0.019264  0.022347  0.025833  0.017677  0.019556  0.012071
NC_001788.     0.023311  0.017867  0.018940  0.017812  0.028491  0.016411  0.013270  0.035345  0.038396  0.027223
 0.033988  0.026254  0.030960  0.029888  0.029745  0.026291  0.034777  0.023458  0.023345  0.013961  0.010494  0.
035054  0.033210  0.033693  0.051057  0.027609  0.088313  0.014005  0.053112  0.058153  0.013078  0.007874  0.
024929  0.029559  0.000000  0.016243  0.007212  0.003726  0.012948  0.010002  0.022563
NC_001321.     0.039554  0.034110  0.035183  0.034055  0.044734  0.032654  0.029513  0.051588  0.054639  0.010980
 0.019916  0.020316  0.014717  0.013645  0.013502  0.010048  0.019715  0.012784  0.012359  0.009694  0.009860  0.
021132  0.024163  0.025131  0.034814  0.015812  0.072070  0.030248  0.041315  0.046356  0.003165  0.008369  0.
009112  0.019264  0.016243  0.000000  0.009031  0.012517  0.008096  0.006240  0.007193
NC_005270.     0.030523  0.025079  0.026152  0.025024  0.035703  0.023623  0.020482  0.042557  0.045608  0.020011
 0.027695  0.019679  0.023748  0.022676  0.022533  0.019079  0.027565  0.016246  0.016133  0.009057  0.006624  0.
027842  0.026051  0.027019  0.043845  0.023591  0.081101  0.021217  0.049094  0.054135  0.006372  0.000781  0.
017717  0.022347  0.007212  0.009031  0.000000  0.005836  0.008930  0.002790  0.015351
NC_001640.     0.027037  0.021593  0.022666  0.021538  0.032217  0.020137  0.016996  0.039071  0.042122  0.023497
 0.030262  0.025515  0.027234  0.026162  0.026019  0.022565  0.031051  0.019732  0.019619  0.014893  0.012460  0.
031328  0.029484  0.029967  0.047331  0.024272  0.084587  0.017731  0.049775  0.054816  0.009352  0.005055  0.
021203  0.025833  0.003726  0.012517  0.005836  0.000000  0.009611  0.006276  0.018837
NC_005275.     0.035193  0.029749  0.030822  0.029694  0.040373  0.028293  0.025152  0.047227  0.050278  0.016484
 0.022106  0.017731  0.019078  0.018006  0.017863  0.014409  0.022895  0.020880  0.020455  0.007110  0.011011  0.
029228  0.032259  0.033227  0.039175  0.014661  0.076431  0.025887  0.040164  0.045205  0.005503  0.008217  0.
013047  0.017677  0.012948  0.008096  0.008930  0.009611  0.000000  0.007092  0.010681
NC_006931.     0.033313  0.027869  0.028942  0.027814  0.038493  0.026413  0.023272  0.045347  0.048398  0.017220
 0.025271  0.020197  0.020957  0.019885  0.019742  0.016288  0.025070  0.013788  0.013363  0.009575  0.007142  0.
025051  0.025167  0.026135  0.041054  0.021167  0.078310  0.024007  0.046670  0.051711  0.003948  0.002128  0.
014926  0.019556  0.010002  0.006240  0.002790  0.006276  0.007092  0.000000  0.012560
```

```
NC_010640.     0.045874  0.040430  0.041503  0.040375  0.051054  0.038974  0.035833  0.057908  0.060959  0.008112
 0.021164  0.013123  0.008397  0.008539  0.007182  0.004802  0.020963  0.015104  0.015853  0.008602  0.013125  0.
021573  0.025069  0.025309  0.028494  0.019186  0.067664  0.036568  0.042563  0.047604  0.009485  0.014689  0.
002719  0.012071  0.022563  0.007193  0.015351  0.018837  0.010681  0.012560  0.000000
```

59 ebolavirus complete genomes

```
    59
FJ217161.1     0.000000  0.001623  0.001781  0.001675  0.001623  0.005529  0.020316  0.020515  0.020793  0.020862
 0.020272  0.020272  0.014579  0.014897  0.014685  0.012620  0.013255  0.012128  0.012128  0.012128  0.012075  0.
013891  0.015366  0.015472  0.015366  0.014575  0.014564  0.013995  0.014650  0.014733  0.014680  0.015830  0.
015988  0.015988  0.016094  0.015935  0.015988  0.015619  0.014564  0.014631  0.014657  0.014560  0.014487  0.
014548  0.014646  0.014551  0.014518  0.014530  0.014620  0.014434  0.014575  0.014508  0.014453  0.014405  0.
014611  0.014578  0.014575  0.014575  0.014628
KC545393.1     0.001623  0.000000  0.000159  0.000053  0.000053  0.006388  0.018693  0.018892  0.019170  0.019239
 0.018649  0.018649  0.012956  0.013274  0.013062  0.010997  0.011632  0.010505  0.010505  0.010505  0.010452  0.
012268  0.013743  0.013849  0.013743  0.012952  0.012941  0.012372  0.013027  0.013110  0.013057  0.014207  0.
014365  0.014365  0.014471  0.014312  0.014365  0.013996  0.012941  0.013008  0.013034  0.012937  0.012864  0.
012925  0.013023  0.012928  0.012895  0.012907  0.012997  0.012811  0.012952  0.012885  0.012830  0.012782  0.
012988  0.012955  0.012952  0.012952  0.013005
KC545395.1     0.001781  0.000159  0.000000  0.000159  0.000159  0.006547  0.018535  0.018734  0.019012  0.019081
 0.018491  0.018491  0.012798  0.013116  0.012904  0.010839  0.011474  0.010347  0.010347  0.010347  0.010294  0.
012110  0.013585  0.013691  0.013585  0.012794  0.012783  0.012214  0.012869  0.012952  0.012899  0.014049  0.
014207  0.014207  0.014313  0.014154  0.014207  0.013838  0.012783  0.012850  0.012876  0.012779  0.012706  0.
012767  0.012865  0.012770  0.012737  0.012749  0.012839  0.012653  0.012794  0.012727  0.012672  0.012624  0.
012830  0.012797  0.012794  0.012794  0.012847
KC545394.1     0.001675  0.000053  0.000159  0.000000  0.000106  0.006388  0.018640  0.018839  0.019117  0.019186
 0.018596  0.018596  0.012904  0.013222  0.013010  0.010944  0.011579  0.010452  0.010452  0.010452  0.010399  0.
012215  0.013690  0.013796  0.013690  0.012899  0.012888  0.012319  0.012974  0.013057  0.013005  0.014154  0.
014312  0.014312  0.014418  0.014259  0.014312  0.013943  0.012888  0.012955  0.012981  0.012884  0.012811  0.
012872  0.012970  0.012875  0.012842  0.012854  0.012944  0.012758  0.012899  0.012832  0.012777  0.012729  0.
012935  0.012902  0.012899  0.012899  0.012952
KC545396.1     0.001623  0.000053  0.000159  0.000106  0.000000  0.006388  0.018693  0.018892  0.019170  0.019239
 0.018649  0.018649  0.012956  0.013274  0.013062  0.010997  0.011632  0.010505  0.010505  0.010505  0.010452  0.
012268  0.013743  0.013849  0.013743  0.012952  0.012941  0.012372  0.013027  0.013110  0.013057  0.014207  0.
014365  0.014365  0.014471  0.014312  0.014365  0.013996  0.012941  0.013008  0.013034  0.012937  0.012864  0.
012925  0.013023  0.012928  0.012895  0.012907  0.012997  0.012811  0.012952  0.012885  0.012830  0.012782  0.
012988  0.012955  0.012952  0.012952  0.013005
FJ217162.1     0.005529  0.006388  0.006547  0.006388  0.006388  0.000000  0.022859  0.023058  0.023336  0.023405
 0.022815  0.022815  0.017122  0.017440  0.017228  0.015163  0.015798  0.014671  0.014671  0.014671  0.014618  0.
016434  0.017909  0.018015  0.017909  0.017118  0.017107  0.016538  0.017193  0.017276  0.017223  0.018373  0.
018531  0.018531  0.018637  0.018478  0.018531  0.018162  0.017107  0.017174  0.017200  0.017103  0.017030  0.
017091  0.017189  0.017094  0.017061  0.017073  0.017163  0.016977  0.017118  0.017051  0.016996  0.016948  0.
017154  0.017121  0.017118  0.017118  0.017171
AF522874.1     0.020316  0.018693  0.018535  0.018640  0.018693  0.022859  0.000000  0.000798  0.000477  0.001002
 0.001154  0.001524  0.005736  0.005418  0.005630  0.007696  0.007060  0.008188  0.008188  0.008188  0.008241  0.
006424  0.009970  0.009865  0.009970  0.010234  0.010325  0.010709  0.009999  0.010076  0.010076  0.009428  0.
009323  0.009323  0.009217  0.009376  0.009270  0.009692  0.010325  0.010369  0.010416  0.010413  0.010451  0.
010398  0.010400  0.010413  0.010426  0.010441  0.010355  0.010504  0.010498  0.010465  0.010464  0.010588  0.
010410  0.010422  0.010498  0.010498  0.010498
AB050936.1     0.020515  0.018892  0.018734  0.018839  0.018892  0.023058  0.000798  0.000000  0.000405  0.000347
 0.000700  0.000859  0.005935  0.005617  0.005829  0.007895  0.007259  0.008387  0.008387  0.008387  0.008440  0.
006623  0.009882  0.009777  0.009882  0.010146  0.010237  0.010621  0.009911  0.009988  0.009988  0.009340  0.
009235  0.009235  0.009129  0.009288  0.009182  0.009604  0.010237  0.010281  0.010328  0.010325  0.010363  0.
010310  0.010312  0.010325  0.010338  0.010353  0.010267  0.010416  0.010410  0.010377  0.010376  0.010500  0.
010322  0.010334  0.010410  0.010410  0.010410
JX477166.1     0.020793  0.019170  0.019012  0.019117  0.019170  0.023336  0.000477  0.000405  0.000000  0.000525
 0.000894  0.001053  0.006213  0.005895  0.006107  0.008173  0.007537  0.008665  0.008665  0.008665  0.008718  0.
006901  0.010076  0.009971  0.010076  0.010340  0.010431  0.010815  0.010105  0.010182  0.010182  0.009534  0.
009429  0.009429  0.009323  0.009482  0.009376  0.009798  0.010431  0.010475  0.010522  0.010519  0.010557  0.
010504  0.010506  0.010519  0.010532  0.010547  0.010461  0.010610  0.010604  0.010571  0.010570  0.010694  0.
```

```
010516  0.010528  0.010604  0.010604  0.010604
FJ621585.1     0.020862  0.019239  0.019081  0.019186  0.019239  0.023405  0.001002  0.000347  0.000525  0.000000
 0.000713  0.000872  0.006282  0.005964  0.006176  0.008242  0.007606  0.008734  0.008734  0.008734  0.008787  0.
006970  0.009895  0.009790  0.009895  0.010159  0.010250  0.010634  0.009924  0.010001  0.010001  0.009353  0.
009248  0.009248  0.009142  0.009301  0.009195  0.009617  0.010250  0.010294  0.010341  0.010338  0.010376  0.
010323  0.010325  0.010338  0.010351  0.010366  0.010280  0.010429  0.010423  0.010390  0.010389  0.010513  0.
010335  0.010347  0.010423  0.010423  0.010423
FJ621583.1     0.020272  0.018649  0.018491  0.018596  0.018649  0.022815  0.001154  0.000700  0.000894  0.000713
 0.000000  0.000370  0.005692  0.005374  0.005586  0.007652  0.007016  0.008144  0.008144  0.008144  0.008197  0.
006380  0.009182  0.009077  0.009182  0.009446  0.009537  0.009921  0.009211  0.009288  0.009288  0.008640  0.
008535  0.008535  0.008429  0.008588  0.008482  0.008904  0.009537  0.009581  0.009628  0.009625  0.009663  0.
009610  0.009612  0.009625  0.009638  0.009653  0.009567  0.009716  0.009710  0.009677  0.009676  0.009800  0.
009622  0.009634  0.009710  0.009710  0.009710
JX477165.1     0.020272  0.018649  0.018491  0.018596  0.018649  0.022815  0.001524  0.000859  0.001053  0.000872
 0.000370  0.000000  0.005692  0.005374  0.005586  0.007652  0.007016  0.008144  0.008144  0.008144  0.008197  0.
006380  0.009023  0.008918  0.009023  0.009287  0.009378  0.009762  0.009052  0.009129  0.009129  0.008481  0.
008376  0.008376  0.008270  0.008429  0.008323  0.008745  0.009378  0.009422  0.009469  0.009466  0.009504  0.
009451  0.009453  0.009466  0.009479  0.009494  0.009408  0.009557  0.009551  0.009518  0.009517  0.009641  0.
009463  0.009475  0.009551  0.009551  0.009551
FJ968794.1     0.014579  0.012956  0.012798  0.012904  0.012956  0.017122  0.005736  0.005935  0.006213  0.006282
 0.005692  0.005692  0.000000  0.000318  0.000477  0.001959  0.001430  0.002451  0.002451  0.002451  0.002504  0.
001165  0.006842  0.006842  0.006842  0.006103  0.005909  0.005699  0.006439  0.006525  0.006472  0.006859  0.
006964  0.006911  0.006911  0.006806  0.006806  0.006964  0.005856  0.006363  0.006351  0.006468  0.006422  0.
006382  0.006465  0.006458  0.006454  0.006487  0.006380  0.006475  0.006525  0.006468  0.006368  0.006440  0.
006519  0.006469  0.006420  0.006472  0.006525
KC242783.2     0.014897  0.013274  0.013116  0.013222  0.013274  0.017440  0.005418  0.005617  0.005895  0.005964
 0.005374  0.005374  0.000318  0.000000  0.000371  0.002277  0.001642  0.002769  0.002769  0.002769  0.002822  0.
001006  0.006524  0.006524  0.006524  0.005785  0.005633  0.006017  0.006121  0.006207  0.006154  0.006541  0.
006646  0.006593  0.006593  0.006488  0.006488  0.006646  0.005633  0.006045  0.006033  0.006150  0.006104  0.
006064  0.006147  0.006140  0.006136  0.006169  0.006062  0.006157  0.006207  0.006150  0.006050  0.006122  0.
006201  0.006151  0.006102  0.006154  0.006207
EU338380.1     0.014685  0.013062  0.012904  0.013010  0.013062  0.017228  0.005630  0.005829  0.006107  0.006176
 0.005586  0.005586  0.000477  0.000371  0.000000  0.002065  0.001430  0.002557  0.002557  0.002557  0.002610  0.
000847  0.006365  0.006365  0.006365  0.005626  0.005432  0.005646  0.005962  0.006048  0.005995  0.006382  0.
006487  0.006434  0.006434  0.006329  0.006329  0.006487  0.005379  0.005886  0.005874  0.005991  0.005945  0.
005905  0.005988  0.005981  0.005977  0.006010  0.005903  0.005998  0.006048  0.005991  0.005891  0.005963  0.
006042  0.005992  0.005943  0.005995  0.006048
AY729654.1     0.012620  0.010997  0.010839  0.010944  0.010997  0.015163  0.007696  0.007895  0.008173  0.008242
 0.007652  0.007652  0.001959  0.002277  0.002065  0.000000  0.000635  0.000673  0.000673  0.000673  0.000726  0.
001271  0.006047  0.006047  0.006047  0.005308  0.005114  0.004886  0.005644  0.005730  0.005677  0.006064  0.
006169  0.006116  0.006116  0.006011  0.006011  0.006169  0.005061  0.005568  0.005556  0.005673  0.005627  0.
005587  0.005670  0.005663  0.005659  0.005692  0.005585  0.005680  0.005730  0.005673  0.005573  0.005645  0.
005724  0.005674  0.005625  0.005677  0.005730
JN638998.1     0.013255  0.011632  0.011474  0.011579  0.011632  0.015798  0.007060  0.007259  0.007537  0.007606
 0.007016  0.007016  0.001430  0.001642  0.001430  0.000635  0.000000  0.001127  0.001127  0.001127  0.001180  0.
000900  0.005464  0.005464  0.005464  0.004725  0.004531  0.004428  0.005061  0.005147  0.005094  0.005481  0.
005586  0.005533  0.005533  0.005428  0.005428  0.005586  0.004478  0.004985  0.004973  0.005090  0.005044  0.
005004  0.005087  0.005080  0.005076  0.005109  0.005002  0.005097  0.005147  0.005090  0.004990  0.005062  0.
005141  0.005091  0.005042  0.005095  0.005147
KC545389.1     0.012128  0.010505  0.010347  0.010452  0.010505  0.014671  0.008188  0.008387  0.008665  0.008734
 0.008144  0.008144  0.002451  0.002769  0.002557  0.000673  0.001127  0.000000  0.000053  0.000000  0.000053  0.
001785  0.005816  0.005816  0.005816  0.005077  0.004883  0.004655  0.005413  0.005499  0.005446  0.005833  0.
005938  0.005885  0.005885  0.005780  0.005780  0.005938  0.004830  0.005337  0.005325  0.005442  0.005396  0.
005356  0.005439  0.005432  0.005428  0.005461  0.005354  0.005449  0.005499  0.005442  0.005342  0.005414  0.
005493  0.005443  0.005394  0.005446  0.005499
KC545390.1     0.012128  0.010505  0.010347  0.010452  0.010505  0.014671  0.008188  0.008387  0.008665  0.008734
 0.008144  0.008144  0.002451  0.002769  0.002557  0.000673  0.001127  0.000053  0.000000  0.000053  0.000106  0.
001785  0.005763  0.005763  0.005763  0.005024  0.004830  0.004602  0.005360  0.005446  0.005393  0.005780  0.
005885  0.005832  0.005832  0.005727  0.005727  0.005885  0.004777  0.005284  0.005272  0.005389  0.005343  0.
005303  0.005386  0.005379  0.005375  0.005408  0.005301  0.005396  0.005446  0.005389  0.005289  0.005361  0.
005440  0.005390  0.005341  0.005393  0.005446
KC545391.1     0.012128  0.010505  0.010347  0.010452  0.010505  0.014671  0.008188  0.008387  0.008665  0.008734
```

```
 0.008144  0.008144  0.002451  0.002769  0.002557  0.000673  0.001127  0.000000  0.000053  0.000000  0.000053  0.
001785  0.005816  0.005816  0.005816  0.005077  0.004883  0.004655  0.005413  0.005499  0.005446  0.005833  0.
005938  0.005885  0.005885  0.005780  0.005780  0.005938  0.004830  0.005337  0.005325  0.005442  0.005396  0.
005356  0.005439  0.005432  0.005428  0.005461  0.005354  0.005449  0.005499  0.005442  0.005342  0.005414  0.
005493  0.005443  0.005394  0.005447  0.005499
KC545392.1     0.012075  0.010452  0.010294  0.010399  0.010452  0.014618  0.008241  0.008440  0.008718  0.008787
 0.008197  0.008197  0.002504  0.002822  0.002610  0.000726  0.001180  0.000053  0.000106  0.000053  0.000000  0.
001838  0.005869  0.005869  0.005869  0.005130  0.004936  0.004708  0.005466  0.005552  0.005499  0.005886  0.
005991  0.005938  0.005938  0.005833  0.005833  0.005991  0.004883  0.005390  0.005378  0.005495  0.005449  0.
005409  0.005492  0.005485  0.005481  0.005514  0.005407  0.005502  0.005552  0.005495  0.005395  0.005467  0.
005546  0.005496  0.005447  0.005499  0.005552
KC589025.1     0.013891  0.012268  0.012110  0.012215  0.012268  0.016434  0.006424  0.006623  0.006901  0.006970
 0.006380  0.006380  0.001165  0.001006  0.000847  0.001271  0.000900  0.001785  0.001785  0.001785  0.001838  0.
000000  0.005994  0.005994  0.005994  0.005255  0.005061  0.005328  0.005591  0.005677  0.005624  0.006011  0.
006116  0.006063  0.006063  0.005958  0.005958  0.006116  0.005008  0.005515  0.005503  0.005620  0.005574  0.
005534  0.005617  0.005610  0.005606  0.005639  0.005532  0.005627  0.005677  0.005620  0.005520  0.005592  0.
005671  0.005621  0.005572  0.005625  0.005677
KC242801.1     0.015366  0.013743  0.013585  0.013690  0.013743  0.017909  0.009970  0.009882  0.010076  0.009895
 0.009182  0.009023  0.006842  0.006524  0.006365  0.006047  0.005464  0.005816  0.005763  0.005816  0.005869  0.
005994  0.000000  0.000106  0.000000  0.000791  0.000933  0.001371  0.000716  0.000633  0.000685  0.000541  0.
000647  0.000647  0.000752  0.000594  0.000700  0.000278  0.000986  0.000735  0.000709  0.000806  0.000879  0.
000818  0.000720  0.000815  0.000848  0.000836  0.000746  0.000932  0.000791  0.000858  0.000913  0.000961  0.
000755  0.000788  0.000791  0.000791  0.000738
NC_002549.     0.015472  0.013849  0.013691  0.013796  0.013849  0.018015  0.009865  0.009777  0.009971  0.009790
 0.009077  0.008918  0.006842  0.006524  0.006365  0.006047  0.005464  0.005816  0.005763  0.005816  0.005869  0.
005994  0.000106  0.000000  0.000106  0.000897  0.000933  0.001477  0.000822  0.000739  0.000791  0.000436  0.
000542  0.000542  0.000647  0.000489  0.000595  0.000173  0.000986  0.000841  0.000815  0.000912  0.000985  0.
000924  0.000826  0.000921  0.000954  0.000942  0.000852  0.001038  0.000897  0.000964  0.001019  0.001067  0.
000861  0.000894  0.000897  0.000897  0.000844
KC242791.1     0.015366  0.013743  0.013585  0.013690  0.013743  0.017909  0.009970  0.009882  0.010076  0.009895
 0.009182  0.009023  0.006842  0.006524  0.006365  0.006047  0.005464  0.005816  0.005763  0.005816  0.005869  0.
005994  0.000000  0.000106  0.000000  0.000791  0.000933  0.001371  0.000716  0.000633  0.000685  0.000541  0.
000647  0.000647  0.000752  0.000594  0.000700  0.000278  0.000986  0.000735  0.000709  0.000806  0.000879  0.
000818  0.000720  0.000815  0.000848  0.000836  0.000746  0.000932  0.000791  0.000858  0.000913  0.000961  0.
000755  0.000788  0.000791  0.000791  0.000738
KC242792.1     0.014575  0.012952  0.012794  0.012899  0.012952  0.017118  0.010234  0.010146  0.010340  0.010159
 0.009446  0.009287  0.006103  0.005785  0.005626  0.005308  0.004725  0.005077  0.005024  0.005077  0.005130  0.
005255  0.000791  0.000897  0.000791  0.000000  0.000274  0.000580  0.000496  0.000422  0.000422  0.001255  0.
001413  0.001413  0.001519  0.001360  0.001413  0.001044  0.000327  0.000260  0.000248  0.000365  0.000319  0.
000279  0.000362  0.000355  0.000351  0.000384  0.000277  0.000372  0.000422  0.000365  0.000265  0.000354  0.
000416  0.000366  0.000317  0.000369  0.000422
KC242793.1     0.014564  0.012941  0.012783  0.012888  0.012941  0.017107  0.010325  0.010237  0.010431  0.010250
 0.009537  0.009378  0.005909  0.005633  0.005432  0.005114  0.004531  0.004883  0.004830  0.004883  0.004936  0.
005061  0.000933  0.000933  0.000933  0.000274  0.000000  0.000569  0.000770  0.000696  0.000696  0.001266  0.
001424  0.001424  0.001530  0.001371  0.001424  0.001055  0.000053  0.000454  0.000442  0.000559  0.000513  0.
000473  0.000556  0.000549  0.000545  0.000578  0.000471  0.000566  0.000616  0.000559  0.000459  0.000531  0.
000610  0.000560  0.000511  0.000563  0.000616
KC242794.1     0.013995  0.012372  0.012214  0.012319  0.012372  0.016538  0.010709  0.010621  0.010815  0.010634
 0.009921  0.009762  0.005699  0.006017  0.005646  0.004886  0.004428  0.004655  0.004602  0.004655  0.004708  0.
005328  0.001371  0.001477  0.001371  0.000580  0.000569  0.000000  0.000812  0.000844  0.000791  0.001835  0.
001993  0.001993  0.002099  0.001940  0.001993  0.001624  0.000569  0.000682  0.000670  0.000787  0.000741  0.
000701  0.000784  0.000777  0.000773  0.000806  0.000699  0.000794  0.000844  0.000787  0.000687  0.000759  0.
000838  0.000788  0.000739  0.000791  0.000844
AY354458.1     0.014650  0.013027  0.012869  0.012974  0.013027  0.017193  0.009999  0.009911  0.010105  0.009924
 0.009211  0.009052  0.006439  0.006121  0.005962  0.005644  0.005061  0.005413  0.005360  0.005413  0.005466  0.
005591  0.000716  0.000822  0.000716  0.000496  0.000770  0.000812  0.000000  0.000086  0.000077  0.001180  0.
001338  0.001338  0.001444  0.001285  0.001338  0.000969  0.000823  0.000427  0.000512  0.000414  0.000452  0.
000399  0.000401  0.000414  0.000427  0.000442  0.000385  0.000505  0.000499  0.000466  0.000465  0.000589  0.
000411  0.000423  0.000499  0.000499  0.000499
KC242796.1     0.014733  0.013110  0.012952  0.013057  0.013110  0.017276  0.010076  0.009988  0.010182  0.010001
 0.009288  0.009129  0.006525  0.006207  0.006048  0.005730  0.005147  0.005499  0.005446  0.005499  0.005552  0.
005677  0.000633  0.000739  0.000633  0.000422  0.000696  0.000844  0.000086  0.000000  0.000053  0.001097  0.
```

```
001255  0.001255  0.001361  0.001202  0.001255  0.000886  0.000749  0.000353  0.000438  0.000337  0.000375  0.
000322  0.000324  0.000337  0.000350  0.000365  0.000311  0.000428  0.000422  0.000389  0.000388  0.000512  0.
000334  0.000346  0.000422  0.000422  0.000422
KC242799.1     0.014680  0.013057  0.012899  0.013005  0.013057  0.017223  0.010076  0.009988  0.010182  0.010001
 0.009288  0.009129  0.006472  0.006154  0.005995  0.005677  0.005094  0.005446  0.005393  0.005446  0.005499  0.
005624  0.000685  0.000791  0.000685  0.000422  0.000696  0.000791  0.000077  0.000053  0.000000  0.001149  0.
001307  0.001307  0.001413  0.001254  0.001307  0.000938  0.000749  0.000353  0.000438  0.000337  0.000375  0.
000322  0.000324  0.000337  0.000350  0.000365  0.000311  0.000428  0.000422  0.000389  0.000388  0.000512  0.
000334  0.000346  0.000422  0.000422  0.000422
KC242784.1     0.015830  0.014207  0.014049  0.014154  0.014207  0.018373  0.009428  0.009340  0.009534  0.009353
 0.008640  0.008481  0.006859  0.006541  0.006382  0.006064  0.005481  0.005833  0.005780  0.005833  0.005886  0.
006011  0.000541  0.000436  0.000541  0.001255  0.001266  0.001835  0.001180  0.001097  0.001149  0.000000  0.
000158  0.000158  0.000264  0.000105  0.000158  0.000263  0.001266  0.001199  0.001173  0.001270  0.001343  0.
001282  0.001184  0.001279  0.001312  0.001300  0.001210  0.001396  0.001255  0.001322  0.001377  0.001425  0.
001219  0.001252  0.001255  0.001255  0.001202
KC242786.1     0.015988  0.014365  0.014207  0.014312  0.014365  0.018531  0.009323  0.009235  0.009429  0.009248
 0.008535  0.008376  0.006964  0.006646  0.006487  0.006169  0.005586  0.005938  0.005885  0.005938  0.005991  0.
006116  0.000647  0.000542  0.000647  0.001413  0.001424  0.001993  0.001338  0.001255  0.001307  0.000158  0.
000000  0.000053  0.000106  0.000158  0.000158  0.000369  0.001424  0.001357  0.001331  0.001428  0.001501  0.
001440  0.001342  0.001437  0.001470  0.001458  0.001368  0.001554  0.001413  0.001480  0.001535  0.001583  0.
001377  0.001410  0.001413  0.001413  0.001360
KC242787.1     0.015988  0.014365  0.014207  0.014312  0.014365  0.018531  0.009323  0.009235  0.009429  0.009248
 0.008535  0.008376  0.006911  0.006593  0.006434  0.006116  0.005533  0.005885  0.005832  0.005885  0.005938  0.
006063  0.000647  0.000542  0.000647  0.001413  0.001424  0.001993  0.001338  0.001255  0.001307  0.000158  0.
000053  0.000000  0.000106  0.000105  0.000105  0.000369  0.001424  0.001357  0.001331  0.001428  0.001501  0.
001440  0.001342  0.001437  0.001470  0.001458  0.001368  0.001554  0.001413  0.001480  0.001535  0.001583  0.
001377  0.001410  0.001413  0.001413  0.001360
KC242789.1     0.016094  0.014471  0.014313  0.014418  0.014471  0.018637  0.009217  0.009129  0.009323  0.009142
 0.008429  0.008270  0.006911  0.006593  0.006434  0.006116  0.005533  0.005885  0.005832  0.005885  0.005938  0.
006063  0.000752  0.000647  0.000752  0.001519  0.001530  0.002099  0.001444  0.001361  0.001413  0.000264  0.
000106  0.000106  0.000000  0.000159  0.000106  0.000475  0.001530  0.001463  0.001437  0.001534  0.001607  0.
001546  0.001448  0.001543  0.001576  0.001564  0.001474  0.001660  0.001519  0.001586  0.001641  0.001689  0.
001483  0.001516  0.001519  0.001519  0.001466
KC242785.1     0.015935  0.014312  0.014154  0.014259  0.014312  0.018478  0.009376  0.009288  0.009482  0.009301
 0.008588  0.008429  0.006806  0.006488  0.006329  0.006011  0.005428  0.005780  0.005727  0.005780  0.005833  0.
005958  0.000594  0.000489  0.000594  0.001360  0.001371  0.001940  0.001285  0.001202  0.001254  0.000105  0.
000158  0.000105  0.000159  0.000000  0.000106  0.000316  0.001371  0.001304  0.001278  0.001375  0.001448  0.
001387  0.001289  0.001384  0.001417  0.001405  0.001315  0.001501  0.001360  0.001427  0.001482  0.001530  0.
001324  0.001357  0.001360  0.001360  0.001307
KC242790.1     0.015988  0.014365  0.014207  0.014312  0.014365  0.018531  0.009270  0.009182  0.009376  0.009195
 0.008482  0.008323  0.006806  0.006488  0.006329  0.006011  0.005428  0.005780  0.005727  0.005780  0.005833  0.
005958  0.000700  0.000595  0.000700  0.001413  0.001424  0.001993  0.001338  0.001255  0.001307  0.000158  0.
000158  0.000105  0.000106  0.000106  0.000000  0.000422  0.001424  0.001357  0.001331  0.001428  0.001501  0.
001440  0.001342  0.001437  0.001470  0.001458  0.001368  0.001554  0.001413  0.001480  0.001535  0.001583  0.
001377  0.001410  0.001413  0.001413  0.001360
KC242788.1     0.015619  0.013996  0.013838  0.013943  0.013996  0.018162  0.009692  0.009604  0.009798  0.009617
 0.008904  0.008745  0.006964  0.006646  0.006487  0.006169  0.005586  0.005938  0.005885  0.005938  0.005991  0.
006116  0.000278  0.000173  0.000278  0.001044  0.001055  0.001624  0.000969  0.000886  0.000938  0.000263  0.
000369  0.000369  0.000475  0.000316  0.000422  0.000000  0.001108  0.000988  0.000962  0.001059  0.001132  0.
001071  0.000973  0.001068  0.001101  0.001089  0.000999  0.001185  0.001044  0.001111  0.001166  0.001214  0.
001008  0.001041  0.001044  0.001044  0.000991
KC242800.1     0.014564  0.012941  0.012783  0.012888  0.012941  0.017107  0.010325  0.010237  0.010431  0.010250
 0.009537  0.009378  0.005856  0.005633  0.005379  0.005061  0.004478  0.004830  0.004777  0.004830  0.004883  0.
005008  0.000986  0.000986  0.000986  0.000327  0.000053  0.000569  0.000823  0.000749  0.000749  0.001266  0.
001424  0.001424  0.001530  0.001371  0.001424  0.001108  0.000000  0.000507  0.000495  0.000612  0.000566  0.
000526  0.000609  0.000602  0.000598  0.000631  0.000524  0.000619  0.000669  0.000612  0.000512  0.000584  0.
000663  0.000613  0.000564  0.000616  0.000669
KM034555.1     0.014631  0.013008  0.012850  0.012955  0.013008  0.017174  0.010369  0.010281  0.010475  0.010294
 0.009581  0.009422  0.006363  0.006045  0.005886  0.005568  0.004985  0.005337  0.005284  0.005337  0.005390  0.
005515  0.000735  0.000841  0.000735  0.000260  0.000454  0.000682  0.000427  0.000353  0.000353  0.001199  0.
001357  0.001357  0.001463  0.001304  0.001357  0.000988  0.000507  0.000000  0.000085  0.000132  0.000144  0.
000083  0.000102  0.000132  0.000147  0.000153  0.000042  0.000197  0.000162  0.000132  0.000178  0.000226  0.
```

```
000156  0.000106  0.000129  0.000129  0.000162
KM034562.1     0.014657  0.013034  0.012876  0.012981  0.013034  0.017200  0.010416  0.010328  0.010522  0.010341
 0.009628  0.009469  0.006351  0.006033  0.005874  0.005556  0.004973  0.005325  0.005272  0.005325  0.005378  0.
005503  0.000709  0.000815  0.000709  0.000248  0.000442  0.000670  0.000512  0.000438  0.000438  0.001173  0.
001331  0.001331  0.001437  0.001278  0.001331  0.000962  0.000495  0.000085  0.000000  0.000217  0.000206  0.
000159  0.000141  0.000217  0.000232  0.000238  0.000127  0.000259  0.000174  0.000217  0.000204  0.000252  0.
000220  0.000191  0.000082  0.000122  0.000174
KM233039.1     0.014560  0.012937  0.012779  0.012884  0.012937  0.017103  0.010413  0.010325  0.010519  0.010338
 0.009625  0.009466  0.006468  0.006150  0.005991  0.005673  0.005090  0.005442  0.005389  0.005442  0.005495  0.
005620  0.000806  0.000912  0.000806  0.000365  0.000559  0.000787  0.000414  0.000337  0.000337  0.001270  0.
001428  0.001428  0.001534  0.001375  0.001428  0.001059  0.000612  0.000132  0.000217  0.000000  0.000073  0.
000086  0.000086  0.000010  0.000042  0.000030  0.000090  0.000126  0.000085  0.000052  0.000107  0.000175  0.
000051  0.000026  0.000148  0.000095  0.000095
KM034557.1     0.014487  0.012864  0.012706  0.012811  0.012864  0.017030  0.010451  0.010363  0.010557  0.010376
 0.009663  0.009504  0.006422  0.006104  0.005945  0.005627  0.005044  0.005396  0.005343  0.005396  0.005449  0.
005574  0.000879  0.000985  0.000879  0.000319  0.000513  0.000741  0.000452  0.000375  0.000375  0.001343  0.
001501  0.001501  0.001607  0.001448  0.001501  0.001132  0.000566  0.000144  0.000206  0.000073  0.000000  0.
000061  0.000159  0.000064  0.000032  0.000065  0.000133  0.000053  0.000103  0.000046  0.000054  0.000137  0.
000124  0.000091  0.000137  0.000088  0.000141
KM034560.1     0.014548  0.012925  0.012767  0.012872  0.012925  0.017091  0.010398  0.010310  0.010504  0.010323
 0.009610  0.009451  0.006382  0.006064  0.005905  0.005587  0.005004  0.005356  0.005303  0.005356  0.005409  0.
005534  0.000818  0.000924  0.000818  0.000279  0.000473  0.000701  0.000399  0.000322  0.000322  0.001282  0.
001440  0.001440  0.001546  0.001387  0.001440  0.001071  0.000526  0.000083  0.000159  0.000086  0.000061  0.
000000  0.000098  0.000076  0.000073  0.000105  0.000072  0.000114  0.000143  0.000086  0.000095  0.000190  0.
000137  0.000087  0.000100  0.000100  0.000143
KM233050.1     0.014646  0.013023  0.012865  0.012970  0.013023  0.017189  0.010400  0.010312  0.010506  0.010325
 0.009612  0.009453  0.006465  0.006147  0.005988  0.005670  0.005087  0.005439  0.005386  0.005439  0.005492  0.
005617  0.000720  0.000826  0.000720  0.000362  0.000556  0.000784  0.000401  0.000324  0.000324  0.001184  0.
001342  0.001342  0.001448  0.001289  0.001342  0.000973  0.000609  0.000102  0.000141  0.000086  0.000159  0.
000098  0.000000  0.000095  0.000128  0.000116  0.000085  0.000212  0.000098  0.000138  0.000193  0.000241  0.
000079  0.000068  0.000098  0.000098  0.000098
KM233053.1     0.014551  0.012928  0.012770  0.012875  0.012928  0.017094  0.010413  0.010325  0.010519  0.010338
 0.009625  0.009466  0.006458  0.006140  0.005981  0.005663  0.005080  0.005432  0.005379  0.005432  0.005485  0.
005610  0.000815  0.000921  0.000815  0.000355  0.000549  0.000777  0.000414  0.000337  0.000337  0.001279  0.
001437  0.001437  0.001543  0.001384  0.001437  0.001068  0.000602  0.000132  0.000217  0.000010  0.000064  0.
000076  0.000095  0.000000  0.000033  0.000029  0.000090  0.000117  0.000085  0.000052  0.000098  0.000175  0.
000061  0.000027  0.000148  0.000095  0.000095
KM233057.1     0.014518  0.012895  0.012737  0.012842  0.012895  0.017061  0.010426  0.010338  0.010532  0.010351
 0.009638  0.009479  0.006454  0.006136  0.005977  0.005659  0.005076  0.005428  0.005375  0.005428  0.005481  0.
005606  0.000848  0.000954  0.000848  0.000351  0.000545  0.000773  0.000427  0.000350  0.000350  0.001312  0.
001470  0.001470  0.001576  0.001417  0.001470  0.001101  0.000598  0.000147  0.000232  0.000042  0.000032  0.
000073  0.000128  0.000033  0.000000  0.000033  0.000105  0.000084  0.000072  0.000039  0.000086  0.000162  0.
000093  0.000060  0.000163  0.000110  0.000110
KM233063.1     0.014530  0.012907  0.012749  0.012854  0.012907  0.017073  0.010441  0.010353  0.010547  0.010366
 0.009653  0.009494  0.006487  0.006169  0.006010  0.005692  0.005109  0.005461  0.005408  0.005461  0.005514  0.
005639  0.000836  0.000942  0.000836  0.000384  0.000578  0.000806  0.000442  0.000365  0.000365  0.001300  0.
001458  0.001458  0.001564  0.001405  0.001458  0.001089  0.000631  0.000153  0.000238  0.000030  0.000065  0.
000105  0.000116  0.000029  0.000033  0.000000  0.000111  0.000096  0.000064  0.000024  0.000119  0.000147  0.
000081  0.000048  0.000169  0.000116  0.000116
KM233072.1     0.014620  0.012997  0.012839  0.012944  0.012997  0.017163  0.010355  0.010267  0.010461  0.010280
 0.009567  0.009408  0.006380  0.006062  0.005903  0.005585  0.005002  0.005354  0.005301  0.005354  0.005407  0.
005532  0.000746  0.000852  0.000746  0.000277  0.000471  0.000699  0.000385  0.000311  0.000311  0.001210  0.
001368  0.001368  0.001474  0.001315  0.001368  0.000999  0.000524  0.000042  0.000127  0.000090  0.000133  0.
000072  0.000085  0.000090  0.000105  0.000111  0.000000  0.000186  0.000145  0.000112  0.000167  0.000233  0.
000139  0.000089  0.000143  0.000143  0.000145
KM233110.1     0.014434  0.012811  0.012653  0.012758  0.012811  0.016977  0.010504  0.010416  0.010610  0.010429
 0.009716  0.009557  0.006475  0.006157  0.005998  0.005680  0.005097  0.005449  0.005396  0.005449  0.005502  0.
005627  0.000932  0.001038  0.000932  0.000372  0.000566  0.000794  0.000505  0.000428  0.000428  0.001396  0.
001554  0.001554  0.001660  0.001501  0.001554  0.001185  0.000619  0.000197  0.000259  0.000126  0.000053  0.
000114  0.000212  0.000117  0.000084  0.000096  0.000186  0.000000  0.000141  0.000074  0.000107  0.000090  0.
000177  0.000144  0.000190  0.000141  0.000194
KM233070.1     0.014575  0.012952  0.012794  0.012899  0.012952  0.017118  0.010498  0.010410  0.010604  0.010423
```

```
 0.009710  0.009551  0.006525  0.006207  0.006048  0.005730  0.005147  0.005499  0.005446  0.005499  0.005552  0.
005677  0.000791  0.000897  0.000791  0.000422  0.000616  0.000844  0.000499  0.000422  0.000422  0.001255  0.
001413  0.001413  0.001519  0.001360  0.001413  0.001044  0.000669  0.000162  0.000174  0.000085  0.000103  0.
000143  0.000098  0.000085  0.000072  0.000064  0.000145  0.000141  0.000000  0.000067  0.000157  0.000170  0.
000088  0.000076  0.000105  0.000052  0.000053
KM233099.1     0.014508  0.012885  0.012727  0.012832  0.012885  0.017051  0.010465  0.010377  0.010571  0.010390
 0.009677  0.009518  0.006468  0.006150  0.005991  0.005673  0.005090  0.005442  0.005389  0.005442  0.005495  0.
005620  0.000858  0.000964  0.000858  0.000365  0.000559  0.000787  0.000466  0.000389  0.000389  0.001322  0.
001480  0.001480  0.001586  0.001427  0.001480  0.001111  0.000612  0.000132  0.000217  0.000052  0.000046  0.
000086  0.000138  0.000052  0.000039  0.000024  0.000112  0.000074  0.000067  0.000000  0.000100  0.000123  0.
000103  0.000070  0.000148  0.000095  0.000120
KM233097.1     0.014453  0.012830  0.012672  0.012777  0.012830  0.016996  0.010464  0.010376  0.010570  0.010389
 0.009676  0.009517  0.006368  0.006050  0.005891  0.005573  0.004990  0.005342  0.005289  0.005342  0.005395  0.
005520  0.000913  0.001019  0.000913  0.000265  0.000459  0.000687  0.000465  0.000388  0.000388  0.001377  0.
001535  0.001535  0.001641  0.001482  0.001535  0.001166  0.000512  0.000178  0.000204  0.000107  0.000054  0.
000095  0.000193  0.000098  0.000086  0.000119  0.000167  0.000107  0.000157  0.000100  0.000000  0.000124  0.
000158  0.000125  0.000122  0.000122  0.000175
KM233109.1     0.014405  0.012782  0.012624  0.012729  0.012782  0.016948  0.010588  0.010500  0.010694  0.010513
 0.009800  0.009641  0.006440  0.006122  0.005963  0.005645  0.005062  0.005414  0.005361  0.005414  0.005467  0.
005592  0.000961  0.001067  0.000961  0.000354  0.000531  0.000759  0.000589  0.000512  0.000512  0.001425  0.
001583  0.001583  0.001689  0.001530  0.001583  0.001214  0.000584  0.000226  0.000252  0.000175  0.000137  0.
000190  0.000241  0.000175  0.000162  0.000147  0.000233  0.000090  0.000170  0.000123  0.000124  0.000000  0.
000206  0.000173  0.000170  0.000170  0.000223
KM233096.1     0.014611  0.012988  0.012830  0.012935  0.012988  0.017154  0.010410  0.010322  0.010516  0.010335
 0.009622  0.009463  0.006519  0.006201  0.006042  0.005724  0.005141  0.005493  0.005440  0.005493  0.005546  0.
005671  0.000755  0.000861  0.000755  0.000416  0.000610  0.000838  0.000411  0.000334  0.000334  0.001219  0.
001377  0.001377  0.001483  0.001324  0.001377  0.001008  0.000663  0.000156  0.000220  0.000051  0.000124  0.
000137  0.000079  0.000061  0.000093  0.000081  0.000139  0.000177  0.000088  0.000103  0.000158  0.000206  0.
000000  0.000050  0.000151  0.000098  0.000098
KM233103.1     0.014578  0.012955  0.012797  0.012902  0.012955  0.017121  0.010422  0.010334  0.010528  0.010347
 0.009634  0.009475  0.006469  0.006151  0.005992  0.005674  0.005091  0.005443  0.005390  0.005443  0.005496  0.
005621  0.000788  0.000894  0.000788  0.000366  0.000560  0.000788  0.000423  0.000346  0.000346  0.001252  0.
001410  0.001410  0.001516  0.001357  0.001410  0.001041  0.000613  0.000106  0.000191  0.000026  0.000091  0.
000087  0.000068  0.000027  0.000060  0.000048  0.000089  0.000144  0.000076  0.000070  0.000125  0.000173  0.
000050  0.000000  0.000122  0.000076  0.000076
KJ660346.2     0.014575  0.012952  0.012794  0.012899  0.012952  0.017118  0.010498  0.010410  0.010604  0.010423
 0.009710  0.009551  0.006420  0.006102  0.005943  0.005625  0.005042  0.005394  0.005341  0.005394  0.005447  0.
005572  0.000791  0.000897  0.000791  0.000317  0.000511  0.000739  0.000499  0.000422  0.000422  0.001255  0.
001413  0.001413  0.001519  0.001360  0.001413  0.001044  0.000564  0.000129  0.000082  0.000148  0.000137  0.
000100  0.000098  0.000148  0.000163  0.000169  0.000143  0.000190  0.000105  0.000148  0.000122  0.000170  0.
000151  0.000122  0.000000  0.000053  0.000105
KJ660347.2     0.014575  0.012952  0.012794  0.012899  0.012952  0.017118  0.010498  0.010410  0.010604  0.010423
 0.009710  0.009551  0.006472  0.006154  0.005995  0.005677  0.005095  0.005446  0.005393  0.005447  0.005499  0.
005625  0.000791  0.000897  0.000791  0.000369  0.000563  0.000791  0.000499  0.000422  0.000422  0.001255  0.
001413  0.001413  0.001519  0.001360  0.001413  0.001044  0.000616  0.000129  0.000122  0.000095  0.000088  0.
000100  0.000098  0.000095  0.000110  0.000116  0.000143  0.000141  0.000052  0.000095  0.000122  0.000170  0.
000098  0.000076  0.000053  0.000000  0.000053
KJ660348.2     0.014628  0.013005  0.012847  0.012952  0.013005  0.017171  0.010498  0.010410  0.010604  0.010423
 0.009710  0.009551  0.006525  0.006207  0.006048  0.005730  0.005147  0.005499  0.005446  0.005499  0.005552  0.
005677  0.000738  0.000844  0.000738  0.000422  0.000616  0.000844  0.000499  0.000422  0.000422  0.001202  0.
001360  0.001360  0.001466  0.001307  0.001360  0.000991  0.000669  0.000162  0.000174  0.000095  0.000141  0.
000143  0.000098  0.000095  0.000110  0.000116  0.000145  0.000194  0.000053  0.000120  0.000175  0.000223  0.
000098  0.000076  0.000105  0.000053  0.000000
```

30 coronavirus with 4 non-coronavirus whole genomes

```
    34
AF304460.1     0.000000  0.025460  0.046399  0.014989  0.014652  0.013666  0.015049  0.013376  0.022949  0.021213
 0.022271  0.023119  0.016951  0.016535  0.038478  0.038155  0.038381  0.038625  0.038524  0.038260  0.038265  0.
038260  0.038254  0.038227  0.038179  0.038440  0.038317  0.038613  0.038691  0.055269  0.101034  0.133415  0.
139580  0.137598
AF353511.1     0.025460  0.000000  0.059794  0.040450  0.040113  0.039127  0.040510  0.038837  0.012978  0.014599
```

```
 0.014114  0.012951  0.041116  0.040700  0.037350  0.036966  0.037493  0.037615  0.037615  0.037088  0.036943  0.
037088  0.037053  0.037088  0.037012  0.037608  0.037191  0.036967  0.036978  0.068664  0.087639  0.110493  0.
126185  0.124203
NC_005831.     0.046399  0.059794  0.000000  0.036244  0.035910  0.036766  0.036690  0.036928  0.069133  0.066380
 0.066494  0.069518  0.060041  0.062850  0.084877  0.084554  0.084780  0.085024  0.084923  0.084659  0.084664  0.
084659  0.084653  0.084626  0.084578  0.084839  0.084716  0.085012  0.085090  0.015150  0.147433  0.170287  0.
185979  0.183997
AY391777.1     0.014989  0.040450  0.036244  0.000000  0.002454  0.002548  0.001824  0.002516  0.032889  0.030136
 0.030250  0.033274  0.023797  0.026606  0.048633  0.048310  0.048536  0.048780  0.048679  0.048415  0.048420  0.
048415  0.048409  0.048382  0.048334  0.048595  0.048472  0.048768  0.048846  0.045114  0.111189  0.148405  0.
149735  0.147753
U00735.2_B     0.014652  0.040113  0.035910  0.002454  0.000000  0.000986  0.001136  0.001276  0.033223  0.030470
 0.030584  0.033608  0.024131  0.026940  0.048967  0.048644  0.048870  0.049114  0.049013  0.048749  0.048754  0.
048749  0.048743  0.048716  0.048668  0.048929  0.048806  0.049102  0.049180  0.044780  0.111523  0.148068  0.
150069  0.148087
AF391542.1     0.013666  0.039127  0.036766  0.002548  0.000986  0.000000  0.001383  0.000290  0.032367  0.029614
 0.029728  0.032752  0.023275  0.026084  0.048111  0.047788  0.048014  0.048258  0.048157  0.047893  0.047898  0.
047893  0.047887  0.047860  0.047812  0.048073  0.047950  0.048246  0.048324  0.045636  0.110667  0.147082  0.
149213  0.147231
AF220295.1     0.015049  0.040510  0.036690  0.001824  0.001136  0.001383  0.000000  0.001673  0.032443  0.029690
 0.029804  0.032828  0.023351  0.026160  0.048358  0.048340  0.048309  0.048334  0.048233  0.048348  0.048355  0.
048315  0.048328  0.048281  0.048329  0.048181  0.048199  0.048623  0.048639  0.045560  0.110743  0.148465  0.
149289  0.147307
NC_003045.     0.013376  0.038837  0.036928  0.002516  0.001276  0.000290  0.001673  0.000000  0.032205  0.029452
 0.029566  0.032590  0.023113  0.025922  0.047949  0.047626  0.047852  0.048096  0.047995  0.047731  0.047736  0.
047731  0.047725  0.047698  0.047650  0.047911  0.047788  0.048084  0.048162  0.045798  0.110505  0.146792  0.
149051  0.147069
AF208067.1     0.022949  0.012978  0.069133  0.032889  0.033223  0.032367  0.032443  0.032205  0.000000  0.002753
 0.002639  0.000636  0.029649  0.029233  0.030966  0.030885  0.031155  0.031043  0.031043  0.030911  0.030768  0.
030877  0.030862  0.030877  0.030896  0.031084  0.030808  0.030712  0.030662  0.078003  0.078300  0.120933  0.
116846  0.114864
AF201929.1     0.021213  0.014599  0.066380  0.030136  0.030470  0.029614  0.029690  0.029452  0.002753  0.000000
 0.001428  0.003138  0.029046  0.028630  0.029230  0.029149  0.029419  0.029307  0.029307  0.029175  0.029032  0.
029141  0.029126  0.029141  0.029160  0.029348  0.029072  0.028976  0.028926  0.075250  0.081053  0.122554  0.
119599  0.117617
AF208066.1     0.022271  0.014114  0.066494  0.030250  0.030584  0.029728  0.029804  0.029566  0.002639  0.001428
 0.000000  0.003024  0.030474  0.030058  0.030288  0.030207  0.030477  0.030365  0.030365  0.030233  0.030090  0.
030199  0.030184  0.030199  0.030218  0.030406  0.030130  0.030034  0.029984  0.075364  0.080939  0.122069  0.
119485  0.117503
NC_001846.     0.023119  0.012951  0.069518  0.033274  0.033608  0.032752  0.032828  0.032590  0.000636  0.003138
 0.003024  0.000000  0.029013  0.028597  0.030688  0.030607  0.030877  0.030765  0.030765  0.030633  0.030490  0.
030599  0.030584  0.030599  0.030618  0.030806  0.030530  0.030434  0.030384  0.078388  0.077915  0.120906  0.
116461  0.114479
NC_001451.     0.016951  0.041116  0.060041  0.023797  0.024131  0.023275  0.023351  0.023113  0.029649  0.029046
 0.030474  0.029013  0.000000  0.002809  0.037600  0.037582  0.037551  0.037560  0.037459  0.037590  0.037597  0.
037557  0.037570  0.037523  0.037571  0.037423  0.037441  0.037865  0.037881  0.068911  0.087392  0.137707  0.
125938  0.123956
EU095850.1     0.016535  0.040700  0.062850  0.026606  0.026940  0.026084  0.026160  0.025922  0.029233  0.028630
 0.030058  0.028597  0.002809  0.000000  0.036644  0.036626  0.036595  0.036604  0.036503  0.036634  0.036641  0.
036601  0.036614  0.036567  0.036615  0.036467  0.036485  0.036909  0.036925  0.071720  0.084583  0.136751  0.
123129  0.121147
AY278488.2     0.038478  0.037350  0.084877  0.048633  0.048967  0.048111  0.048358  0.047949  0.030966  0.029230
 0.030288  0.030688  0.037600  0.036644  0.000000  0.000384  0.000189  0.000265  0.000265  0.000262  0.000407  0.
000262  0.000297  0.000262  0.000338  0.000258  0.000161  0.000383  0.000372  0.093747  0.065202  0.100107  0.
101102  0.099120
AY278741.1     0.038155  0.036966  0.084554  0.048310  0.048644  0.047788  0.048340  0.047626  0.030885  0.029149
 0.030207  0.030607  0.037582  0.036626  0.000384  0.000000  0.000527  0.000649  0.000649  0.000122  0.000117  0.
000122  0.000099  0.000122  0.000046  0.000642  0.000225  0.000458  0.000536  0.093424  0.065121  0.100125  0.
101425  0.099443
AY278491.2     0.038381  0.037493  0.084780  0.048536  0.048870  0.048014  0.048309  0.047852  0.031155  0.029419
 0.030477  0.030877  0.037551  0.036595  0.000189  0.000527  0.000000  0.000244  0.000143  0.000405  0.000550  0.
000405  0.000440  0.000405  0.000481  0.000128  0.000347  0.000526  0.000515  0.093650  0.065391  0.100156  0.
```

101199 0.099217
AY278554.2   0.038625 0.037615 0.085024 0.048780 0.049114 0.048258 0.048334 0.048096 0.031043 0.029307
 0.030365 0.030765 0.037560 0.036604 0.000265 0.000649 0.000244 0.000000 0.000101 0.000527 0.000672 0.
000527 0.000562 0.000527 0.000603 0.000185 0.000424 0.000648 0.000637 0.093894 0.065279 0.100147 0.
100955 0.098973
AY282752.2   0.038524 0.037615 0.084923 0.048679 0.049013 0.048157 0.048233 0.047995 0.031043 0.029307
 0.030365 0.030765 0.037459 0.036503 0.000265 0.000649 0.000143 0.000101 0.000000 0.000527 0.000672 0.
000527 0.000562 0.000527 0.000603 0.000084 0.000424 0.000648 0.000637 0.093793 0.065279 0.100248 0.
101056 0.099074
AY283794.1   0.038260 0.037088 0.084659 0.048415 0.048749 0.047893 0.048348 0.047731 0.030911 0.029175
 0.030233 0.030633 0.037590 0.036634 0.000262 0.000122 0.000405 0.000527 0.000527 0.000000 0.000145 0.
000034 0.000049 0.000067 0.000081 0.000520 0.000149 0.000353 0.000431 0.093529 0.065147 0.100117 0.
101320 0.099338
AY283795.1   0.038265 0.036943 0.084664 0.048420 0.048754 0.047898 0.048355 0.047736 0.030768 0.029032
 0.030090 0.030490 0.037597 0.036641 0.000407 0.000117 0.000550 0.000672 0.000672 0.000145 0.000000 0.
000145 0.000110 0.000145 0.000128 0.000665 0.000248 0.000347 0.000425 0.093534 0.065004 0.100110 0.
101314 0.099332
AY283796.1   0.038260 0.037088 0.084659 0.048415 0.048749 0.047893 0.048315 0.047731 0.030877 0.029141
 0.030199 0.030599 0.037557 0.036601 0.000262 0.000122 0.000405 0.000527 0.000527 0.000034 0.000145 0.
000000 0.000035 0.000034 0.000081 0.000520 0.000116 0.000353 0.000431 0.093529 0.065113 0.100150 0.
101320 0.099338
AY283797.1   0.038254 0.037053 0.084653 0.048409 0.048743 0.047887 0.048328 0.047725 0.030862 0.029126
 0.030184 0.030584 0.037570 0.036614 0.000297 0.000099 0.000440 0.000562 0.000562 0.000049 0.000110 0.
000035 0.000000 0.000047 0.000075 0.000555 0.000138 0.000359 0.000437 0.093523 0.065098 0.100137 0.
101326 0.099344
AY283798.2   0.038227 0.037088 0.084626 0.048382 0.048716 0.047860 0.048281 0.047698 0.030877 0.029141
 0.030199 0.030599 0.037523 0.036567 0.000262 0.000122 0.000405 0.000527 0.000527 0.000067 0.000145 0.
000034 0.000047 0.000000 0.000076 0.000520 0.000103 0.000386 0.000464 0.093496 0.065113 0.100184 0.
101353 0.099371
AY291451.1   0.038179 0.037012 0.084578 0.048334 0.048668 0.047812 0.048329 0.047650 0.030896 0.029160
 0.030218 0.030618 0.037571 0.036615 0.000338 0.000046 0.000481 0.000603 0.000603 0.000081 0.000128 0.
000081 0.000075 0.000076 0.000000 0.000596 0.000179 0.000434 0.000512 0.093448 0.065132 0.100136 0.
101401 0.099419
NC_004718.   0.038440 0.037608 0.084839 0.048595 0.048929 0.048073 0.048181 0.047911 0.031084 0.029348
 0.030406 0.030806 0.037423 0.036467 0.000258 0.000642 0.000128 0.000185 0.000084 0.000520 0.000665 0.
000520 0.000555 0.000520 0.000596 0.000000 0.000417 0.000641 0.000630 0.093709 0.065320 0.100284 0.
101140 0.099158
AY297028.1   0.038317 0.037191 0.084716 0.048472 0.048806 0.047950 0.048199 0.047788 0.030808 0.029072
 0.030130 0.030530 0.037441 0.036485 0.000161 0.000225 0.000347 0.000424 0.000424 0.000149 0.000248 0.
000116 0.000138 0.000103 0.000179 0.000417 0.000000 0.000424 0.000440 0.093586 0.065044 0.100266 0.
101263 0.099281
AY572034.1   0.038613 0.036967 0.085012 0.048768 0.049102 0.048246 0.048623 0.048084 0.030712 0.028976
 0.030034 0.030434 0.037865 0.036909 0.000383 0.000458 0.000526 0.000648 0.000648 0.000353 0.000347 0.
000353 0.000359 0.000386 0.000434 0.000641 0.000424 0.000000 0.000078 0.093882 0.064948 0.099842 0.
100967 0.098985
AY572035.1   0.038691 0.036978 0.085090 0.048846 0.049180 0.048324 0.048639 0.048162 0.030662 0.028926
 0.029984 0.030384 0.037881 0.036925 0.000372 0.000536 0.000515 0.000637 0.000637 0.000431 0.000425 0.
000431 0.000437 0.000464 0.000512 0.000630 0.000440 0.000078 0.000000 0.093960 0.064898 0.099826 0.
100889 0.098907
NC_006577.   0.055269 0.068664 0.015150 0.045114 0.044780 0.045636 0.045560 0.045798 0.078003 0.075250
 0.075364 0.078388 0.068911 0.071720 0.093747 0.093424 0.093650 0.093894 0.093793 0.093529 0.093534 0.
093529 0.093523 0.093496 0.093448 0.093709 0.093586 0.093882 0.093960 0.000000 0.156303 0.179157 0.
194849 0.192867
NC_001564.   0.101034 0.087639 0.147433 0.111189 0.111523 0.110667 0.110743 0.110505 0.078300 0.081053
 0.080939 0.077915 0.087392 0.084583 0.065202 0.065121 0.065391 0.065279 0.065279 0.065147 0.065004 0.
065113 0.065098 0.065113 0.065132 0.065320 0.065044 0.064948 0.064898 0.156303 0.000000 0.062985 0.
065537 0.036564
NC_004102.   0.133415 0.110493 0.170287 0.148405 0.148068 0.147082 0.148465 0.146792 0.120933 0.122554
 0.122069 0.120906 0.137707 0.136751 0.100107 0.100125 0.100156 0.100147 0.100248 0.100117 0.100110 0.
100150 0.100137 0.100184 0.100136 0.100284 0.100266 0.099842 0.099826 0.179157 0.062985 0.000000 0.
114706 0.081825
NC_001512.   0.139580 0.126185 0.185979 0.149735 0.150069 0.149213 0.149289 0.149051 0.116846 0.119599

```
 0.119485  0.116461  0.125938  0.123129  0.101102  0.101425  0.101199  0.100955  0.101056  0.101320  0.101314  0.
101320  0.101326  0.101353  0.101401  0.101140  0.101263  0.100967  0.100889  0.194849  0.065537  0.114706  0.
000000  0.032881
NC_001544.     0.137598  0.124203  0.183997  0.147753  0.148087  0.147231  0.147307  0.147069  0.114864  0.117617
 0.117503  0.114479  0.123956  0.121147  0.099120  0.099443  0.099217  0.098973  0.099074  0.099338  0.099332  0.
099338  0.099344  0.099371  0.099419  0.099158  0.099281  0.098985  0.098907  0.192867  0.036564  0.081825  0.
032881  0.000000
```

30 bacterial whole genomes

```
    30
CP001598.1     0.000000  0.000002  0.008350  0.000036  0.040600  0.029651  0.040328  0.033443  0.039866  0.042664
 0.040806  0.138857  0.139792  0.140149  0.158535  0.149838  0.168940  0.168433  0.005037  0.019943  0.015584  0.
010154  0.063563  0.063897  0.063537  0.064315  0.077344  0.076710  0.078638  0.078797
AE016879.1     0.000002  0.000000  0.008352  0.000037  0.040598  0.029649  0.040326  0.033441  0.039864  0.042662
 0.040804  0.138857  0.139794  0.140149  0.158537  0.149840  0.168940  0.168435  0.005035  0.019941  0.015583  0.
010152  0.063563  0.063897  0.063537  0.064315  0.077346  0.076710  0.078640  0.078799
CP001215.1     0.008350  0.008352  0.000000  0.008325  0.047564  0.037122  0.047326  0.040818  0.041658  0.040121
 0.038899  0.147170  0.148099  0.148462  0.166295  0.158042  0.177253  0.176192  0.010981  0.024043  0.023357  0.
018504  0.071876  0.072210  0.071850  0.072628  0.085284  0.085023  0.085713  0.085967
AE017225.1     0.000036  0.000037  0.008325  0.000000  0.040587  0.029638  0.040315  0.033430  0.039853  0.042651
 0.040793  0.138858  0.139805  0.140150  0.158548  0.149851  0.168941  0.168446  0.005024  0.019930  0.015620  0.
010179  0.063564  0.063898  0.063538  0.064316  0.077357  0.076711  0.078651  0.078810
CP000976.1     0.040600  0.040598  0.047564  0.040587  0.000000  0.011975  0.000951  0.008583  0.007197  0.008693
 0.010006  0.179183  0.180392  0.180417  0.196678  0.188425  0.207636  0.206575  0.036583  0.024434  0.030338  0.
033761  0.102825  0.102740  0.102233  0.103011  0.117944  0.117075  0.119238  0.119397
CP000048.1     0.029651  0.029649  0.037122  0.029638  0.011975  0.000000  0.012892  0.003792  0.010215  0.013013
 0.011155  0.168234  0.169443  0.169468  0.184862  0.179489  0.195661  0.194600  0.026141  0.013079  0.019389  0.
022682  0.091876  0.091791  0.090767  0.091136  0.106995  0.106126  0.108289  0.108448
CP000993.1     0.040328  0.040326  0.047326  0.040315  0.000951  0.012892  0.000000  0.009500  0.008148  0.009644
 0.010957  0.178911  0.180120  0.180145  0.197595  0.189342  0.208553  0.207492  0.036345  0.025385  0.030066  0.
034678  0.103176  0.103510  0.103150  0.103928  0.117672  0.116803  0.118966  0.119125
CP000049.1     0.033443  0.033441  0.040818  0.033430  0.008583  0.003792  0.009500  0.000000  0.006559  0.009397
 0.007448  0.172026  0.173235  0.173260  0.188558  0.183281  0.199053  0.197992  0.029837  0.016775  0.023181  0.
026474  0.095668  0.095583  0.094559  0.094928  0.110787  0.109918  0.112081  0.112240
CP000246.1     0.039866  0.039864  0.041658  0.039853  0.007197  0.010215  0.008148  0.006559  0.000000  0.002838
 0.002809  0.178449  0.179658  0.179683  0.194654  0.186401  0.205612  0.204551  0.034829  0.019923  0.029604  0.
032897  0.102091  0.102006  0.100982  0.101351  0.117210  0.116341  0.118504  0.118663
CP000312.1     0.042664  0.042662  0.040121  0.042651  0.008693  0.013013  0.009644  0.009397  0.002838  0.000000
 0.001949  0.181247  0.182456  0.182481  0.197492  0.189239  0.208450  0.207389  0.037627  0.022721  0.032402  0.
035695  0.104889  0.104804  0.103780  0.104149  0.120008  0.119139  0.121302  0.121461
BA000016.3     0.040806  0.040804  0.038899  0.040793  0.010006  0.011155  0.010957  0.007448  0.002809  0.001949
 0.000000  0.179389  0.180598  0.180623  0.195543  0.187290  0.206501  0.205440  0.035769  0.020863  0.030544  0.
033837  0.103031  0.102946  0.101922  0.102291  0.118150  0.117281  0.119444  0.119603
CP000527.1     0.138857  0.138857  0.147170  0.138858  0.179183  0.168234  0.178911  0.172026  0.178449  0.181247
 0.179389  0.000000  0.001209  0.001292  0.019952  0.011255  0.030083  0.029850  0.143670  0.158526  0.153350  0.
147645  0.078464  0.078899  0.078387  0.078788  0.063042  0.062527  0.061675  0.061439
AE017285.1     0.139792  0.139794  0.148099  0.139805  0.180392  0.169443  0.180120  0.173235  0.179658  0.182456
 0.180598  0.001209  0.000000  0.000443  0.018743  0.010046  0.029154  0.028641  0.144829  0.159735  0.153447  0.
148023  0.078842  0.079277  0.078765  0.079166  0.063139  0.063317  0.062386  0.062132
CP002297.1     0.140149  0.140149  0.148462  0.140150  0.180417  0.169468  0.180145  0.173260  0.179683  0.182481
 0.180623  0.001292  0.000443  0.000000  0.018718  0.010021  0.028791  0.028616  0.144962  0.159760  0.153342  0.
147580  0.078399  0.078834  0.078701  0.078723  0.063178  0.063439  0.062749  0.062495
AM260480.1     0.158535  0.158537  0.166295  0.158548  0.196678  0.184862  0.197595  0.188558  0.194654  0.197492
 0.195543  0.019952  0.018743  0.018718  0.000000  0.009756  0.010957  0.009897  0.163572  0.178478  0.171121  0.
166244  0.097063  0.097498  0.097419  0.097387  0.081191  0.082060  0.080582  0.080328
CP000091.1     0.149838  0.149840  0.158042  0.149851  0.188425  0.179489  0.189342  0.183281  0.186401  0.189239
 0.187290  0.011255  0.010046  0.010021  0.009756  0.000000  0.019209  0.019079  0.154875  0.169781  0.161809  0.
156807  0.087613  0.087740  0.088722  0.088353  0.072758  0.073363  0.072329  0.072075
CP000578.1     0.168940  0.168940  0.177253  0.168941  0.207636  0.195661  0.208553  0.199053  0.205612  0.208450
 0.206501  0.030083  0.029154  0.028791  0.010957  0.019209  0.000000  0.001841  0.173753  0.186066  0.179709  0.
```

```
173875  0.106161  0.106246  0.107270  0.106901  0.091969  0.092230  0.091540  0.091286
CP001151.1     0.168433  0.168435  0.176192  0.168446  0.206575  0.194600  0.207492  0.197992  0.204551  0.207389
 0.205440  0.029850  0.028641  0.028616  0.009897  0.019079  0.001841  0.000000  0.172692  0.185005  0.180420  0.
175567  0.106386  0.106821  0.107317  0.106948  0.091089  0.091958  0.090479  0.090225
AM295250.1     0.005037  0.005035  0.010981  0.005024  0.036583  0.026141  0.036345  0.029837  0.034829  0.037627
 0.035769  0.143670  0.144829  0.144962  0.163572  0.154875  0.173753  0.172692  0.000000  0.014906  0.013177  0.
007523  0.068376  0.068710  0.068350  0.069128  0.082381  0.081523  0.083675  0.083834
AE015929.1     0.019943  0.019941  0.024043  0.019930  0.024434  0.013079  0.025385  0.016775  0.019923  0.022721
 0.020863  0.158526  0.159735  0.159760  0.178478  0.169781  0.186066  0.185005  0.014906  0.000000  0.009681  0.
012974  0.082168  0.082083  0.081059  0.081441  0.097287  0.096418  0.098581  0.098740
AP006716.1     0.015584  0.015583  0.023357  0.015620  0.030338  0.019389  0.030066  0.023181  0.029604  0.032402
 0.030544  0.153350  0.153447  0.153342  0.171121  0.161809  0.179709  0.180420  0.013177  0.009681  0.000000  0.
007464  0.075949  0.075933  0.077108  0.077116  0.090308  0.090823  0.092337  0.092478
CP001837.1     0.010154  0.010152  0.018504  0.010179  0.033761  0.022682  0.034678  0.026474  0.032897  0.035695
 0.033837  0.147645  0.148023  0.147580  0.166244  0.156807  0.173875  0.175567  0.007523  0.012974  0.007464  0.
000000  0.069194  0.069109  0.069644  0.069652  0.085252  0.085157  0.087484  0.087625
AL590842.1     0.063563  0.063563  0.071876  0.063564  0.102825  0.091876  0.103176  0.095668  0.102091  0.104889
 0.103031  0.078464  0.078842  0.078399  0.097063  0.087613  0.106161  0.106386  0.068376  0.082168  0.075949  0.
069194  0.000000  0.000435  0.001159  0.001167  0.016071  0.015976  0.018303  0.018444
CP001585.1     0.063897  0.063897  0.072210  0.063898  0.102740  0.091791  0.103510  0.095583  0.102006  0.104804
 0.102946  0.078899  0.079277  0.078834  0.097498  0.087740  0.106246  0.106821  0.068710  0.082083  0.075933  0.
069109  0.000435  0.000000  0.001175  0.001183  0.016506  0.016411  0.018738  0.018879
AE009952.1     0.063537  0.063537  0.071850  0.063538  0.102233  0.090767  0.103150  0.094559  0.100982  0.103780
 0.101922  0.078387  0.078765  0.078701  0.097419  0.088722  0.107270  0.107317  0.068350  0.081059  0.077108  0.
069644  0.001159  0.001175  0.000000  0.000778  0.016228  0.015899  0.018226  0.018367
CP001593.1     0.064315  0.064315  0.072628  0.064316  0.103011  0.091136  0.103928  0.094928  0.101351  0.104149
 0.102291  0.078788  0.079166  0.078723  0.097387  0.088353  0.106901  0.106948  0.069128  0.081441  0.077116  0.
069652  0.001167  0.001183  0.000778  0.000000  0.016395  0.016300  0.018627  0.018768
CP001671.1     0.077344  0.077346  0.085284  0.077357  0.117944  0.106995  0.117672  0.110787  0.117210  0.120008
 0.118150  0.063042  0.063139  0.063178  0.081191  0.072758  0.091969  0.091089  0.082381  0.097287  0.090308  0.
085252  0.016071  0.016506  0.016228  0.016395  0.000000  0.000869  0.002232  0.002373
CP000468.1     0.076710  0.076710  0.085023  0.076711  0.117075  0.106126  0.116803  0.109918  0.116341  0.119139
 0.117281  0.062527  0.063317  0.063439  0.082060  0.073363  0.092230  0.091958  0.081523  0.096418  0.090823  0.
085157  0.015976  0.016411  0.015899  0.016300  0.000869  0.000000  0.002327  0.002468
CP001383.1     0.078638  0.078640  0.085713  0.078651  0.119238  0.108289  0.118966  0.112081  0.118504  0.121302
 0.119444  0.061675  0.062386  0.062749  0.080582  0.072329  0.091540  0.090479  0.083675  0.098581  0.092337  0.
087484  0.018303  0.018738  0.018226  0.018627  0.002232  0.002327  0.000000  0.000254
AE005674.2     0.078797  0.078799  0.085967  0.078810  0.119397  0.108448  0.119125  0.112240  0.118663  0.121461
 0.119603  0.061439  0.062132  0.062495  0.080328  0.072075  0.091286  0.090225  0.083834  0.098740  0.092478  0.
087625  0.018444  0.018879  0.018367  0.018768  0.002373  0.002468  0.000254  0.000000


48 Hepatitis E virus (HEV) whole genomes

    48
M73218.1_H     0.000000  0.001774  0.006783  0.003638  0.003889  0.002809  0.001205  0.004025  0.002853  0.000970
 0.002667  0.000720  0.001114  0.003673  0.008408  0.006174  0.021784  0.030945  0.028636  0.030202  0.025812  0.
032817  0.029633  0.027356  0.026203  0.026064  0.031316  0.025408  0.027585  0.025966  0.025548  0.025548  0.
035569  0.027695  0.030782  0.031565  0.031215  0.032253  0.033867  0.033382  0.031538  0.031665  0.033938  0.
033521  0.031608  0.037586  0.036433  0.033033
D10330.1_H     0.001774  0.000000  0.006814  0.002504  0.003426  0.002564  0.002075  0.004056  0.004627  0.002232
 0.002227  0.002357  0.000964  0.003704  0.008439  0.005773  0.021815  0.030976  0.028667  0.030233  0.025843  0.
032848  0.029664  0.027387  0.026234  0.026095  0.031347  0.025439  0.027616  0.025997  0.025579  0.025579  0.
035600  0.027726  0.030813  0.031596  0.031246  0.032284  0.033898  0.033413  0.031569  0.031696  0.033969  0.
033552  0.031639  0.037617  0.036464  0.033064
X99441.1_H     0.006783  0.006814  0.000000  0.004310  0.003388  0.009378  0.005578  0.002758  0.004909  0.007049
 0.004587  0.006817  0.006370  0.003206  0.003266  0.002195  0.015000  0.024161  0.021852  0.023418  0.019028  0.
026033  0.022849  0.020572  0.019419  0.019280  0.024532  0.018624  0.020801  0.019182  0.018764  0.018764  0.
028785  0.020911  0.023998  0.024781  0.024431  0.025469  0.027083  0.026598  0.024754  0.024881  0.027154  0.
026737  0.024824  0.030802  0.029649  0.026249
AF076239.3     0.003638  0.002504  0.004310  0.000000  0.002876  0.005068  0.003742  0.001771  0.003837  0.003391
 0.001530  0.003535  0.003466  0.001472  0.005935  0.003269  0.019311  0.028472  0.026163  0.027729  0.023339  0.
```

```
030344  0.027160  0.024883  0.023730  0.023591  0.028843  0.022935  0.025112  0.023493  0.023075  0.023075  0.
033096  0.025222  0.028309  0.029092  0.028742  0.029780  0.031394  0.030909  0.029065  0.029192  0.031465  0.
031048  0.029135  0.035113  0.033960  0.030560
AF051830.1      0.003889  0.003426  0.003388  0.002876  0.000000  0.005990  0.003993  0.003977  0.005581  0.003661
 0.001371  0.003786  0.003717  0.003678  0.005013  0.002347  0.018389  0.027550  0.025241  0.026807  0.022417  0.
029422  0.026238  0.023961  0.022808  0.022669  0.027921  0.022013  0.024190  0.022571  0.022153  0.022153  0.
032174  0.024300  0.027387  0.028170  0.027820  0.028858  0.030472  0.029987  0.028143  0.028270  0.030543  0.
030126  0.028213  0.034191  0.033038  0.029638
AF185822.1      0.002809  0.002564  0.009378  0.005068  0.005990  0.000000  0.003800  0.006620  0.005662  0.003267
 0.004791  0.003392  0.003008  0.006268  0.011003  0.008337  0.024379  0.033540  0.031231  0.032797  0.028407  0.
035412  0.032228  0.029951  0.028798  0.028659  0.033911  0.028003  0.030180  0.028561  0.028143  0.028143  0.
038164  0.030290  0.033377  0.034160  0.033810  0.034848  0.036462  0.035977  0.034133  0.034260  0.036533  0.
036116  0.034203  0.040181  0.039028  0.035628
AF459438.1      0.001205  0.002075  0.005578  0.003742  0.003993  0.003800  0.000000  0.002820  0.002552  0.001978
 0.002771  0.001728  0.001174  0.003757  0.007426  0.006278  0.020579  0.029740  0.027431  0.028997  0.024607  0.
031612  0.028428  0.026151  0.024998  0.024859  0.030111  0.024203  0.026380  0.024761  0.024343  0.024343  0.
034364  0.026490  0.029577  0.030360  0.030010  0.031048  0.032662  0.032177  0.030333  0.030460  0.032733  0.
032316  0.030403  0.036381  0.035228  0.031828
D11092.1_H      0.004025  0.004056  0.002758  0.001771  0.003977  0.006620  0.002820  0.000000  0.002836  0.004291
 0.002606  0.004059  0.003612  0.001069  0.004685  0.003537  0.017759  0.026920  0.024611  0.026177  0.021787  0.
028792  0.025608  0.023331  0.022178  0.022039  0.027291  0.021383  0.023560  0.021941  0.021523  0.021523  0.
031544  0.023670  0.026757  0.027540  0.027190  0.028228  0.029842  0.029357  0.027513  0.027640  0.029913  0.
029496  0.027583  0.033561  0.032408  0.029008
L25595.1_H      0.002853  0.004627  0.004909  0.003837  0.005581  0.005662  0.002552  0.002836  0.000000  0.002395
 0.004210  0.002270  0.003726  0.003852  0.007521  0.006373  0.019910  0.029071  0.026762  0.028328  0.023938  0.
030943  0.027759  0.025482  0.024329  0.024190  0.029442  0.023534  0.025711  0.024092  0.023674  0.023674  0.
033695  0.025821  0.028908  0.029691  0.029341  0.030379  0.031993  0.031508  0.029664  0.029791  0.032064  0.
031647  0.029734  0.035712  0.034559  0.031159
L08816.1_H      0.000970  0.002232  0.007049  0.003391  0.003661  0.003267  0.001978  0.004291  0.002395  0.000000
 0.002462  0.000250  0.002084  0.003939  0.008674  0.006008  0.022050  0.031211  0.028902  0.030468  0.026078  0.
033083  0.029899  0.027622  0.026469  0.026330  0.031582  0.025674  0.027851  0.026232  0.025814  0.025814  0.
035835  0.027961  0.031048  0.031831  0.031481  0.032519  0.034133  0.033648  0.031804  0.031931  0.034204  0.
033787  0.031874  0.037852  0.036699  0.033299
D11093.1_H      0.002667  0.002227  0.004587  0.001530  0.001371  0.004791  0.002771  0.002606  0.004210  0.002462
 0.000000  0.002564  0.002495  0.002307  0.006212  0.003546  0.019588  0.028749  0.026440  0.028006  0.023616  0.
030621  0.027437  0.025160  0.024007  0.023868  0.029120  0.023212  0.025389  0.023770  0.023352  0.023352  0.
033373  0.025499  0.028586  0.029369  0.029019  0.030057  0.031671  0.031186  0.029342  0.029469  0.031742  0.
031325  0.029412  0.035390  0.034237  0.030837
M94177.1_H      0.000720  0.002357  0.006817  0.003535  0.003786  0.003392  0.001728  0.004059  0.002270  0.000250
 0.002564  0.000000  0.001834  0.003707  0.008442  0.006071  0.021818  0.030979  0.028670  0.030236  0.025846  0.
032851  0.029667  0.027390  0.026237  0.026098  0.031350  0.025442  0.027619  0.026000  0.025582  0.025582  0.
035603  0.027729  0.030816  0.031599  0.031249  0.032287  0.033901  0.033416  0.031572  0.031699  0.033972  0.
033555  0.031642  0.037620  0.036467  0.033067
M80581.1_H      0.001114  0.000964  0.006370  0.003466  0.003717  0.003008  0.001174  0.003612  0.003726  0.002084
 0.002495  0.001834  0.000000  0.003481  0.007995  0.006002  0.021371  0.030532  0.028223  0.029789  0.025399  0.
032404  0.029220  0.026943  0.025790  0.025651  0.030903  0.024995  0.027172  0.025553  0.025135  0.025135  0.
035156  0.027282  0.030369  0.031152  0.030802  0.031840  0.033454  0.032969  0.031125  0.031252  0.033525  0.
033108  0.031195  0.037173  0.036020  0.032620
X98292.1_H      0.003673  0.003704  0.003206  0.001472  0.003678  0.006268  0.003757  0.001069  0.003852  0.003939
 0.002307  0.003707  0.003481  0.000000  0.004734  0.002520  0.018110  0.027271  0.024962  0.026528  0.022138  0.
029143  0.025959  0.023682  0.022529  0.022390  0.027642  0.021734  0.023911  0.022292  0.021874  0.021874  0.
031895  0.024021  0.027108  0.027891  0.027541  0.028579  0.030193  0.029708  0.027864  0.027991  0.030264  0.
029847  0.027934  0.033912  0.032759  0.029359
AY230202.1      0.008408  0.008439  0.003266  0.005935  0.005013  0.011003  0.007426  0.004685  0.007521  0.008674
 0.006212  0.008442  0.007995  0.004734  0.000000  0.002666  0.013375  0.022536  0.020227  0.021793  0.017403  0.
024408  0.021224  0.018947  0.017794  0.017655  0.022907  0.016999  0.019176  0.017557  0.017139  0.017139  0.
027160  0.019286  0.022373  0.023156  0.022806  0.023844  0.025458  0.024973  0.023129  0.023256  0.025529  0.
025112  0.023199  0.029177  0.028024  0.024624
AY204877.1      0.006174  0.005773  0.002195  0.003269  0.002347  0.008337  0.006278  0.003537  0.006373  0.006008
 0.003546  0.006071  0.006002  0.002520  0.002666  0.000000  0.016042  0.025203  0.022894  0.024460  0.020070  0.
027075  0.023891  0.021614  0.020461  0.020322  0.025574  0.019666  0.021843  0.020224  0.019806  0.019806  0.
029827  0.021953  0.025040  0.025823  0.025473  0.026511  0.028125  0.027640  0.025796  0.025923  0.028196  0.
```

```
027779  0.025866  0.031844  0.030691  0.027291
M74506.1_H     0.021784  0.021815  0.015000  0.019311  0.018389  0.024379  0.020579  0.017759  0.019910  0.022050
 0.019588  0.021818  0.021371  0.018110  0.013375  0.016042  0.000000  0.010343  0.006978  0.009769  0.007225  0.
012931  0.008551  0.006132  0.005785  0.006362  0.009810  0.004597  0.005801  0.004182  0.004457  0.004318  0.
013785  0.005911  0.010760  0.012295  0.011615  0.012953  0.013271  0.013797  0.012406  0.012193  0.014491  0.
014074  0.011862  0.015802  0.015527  0.013024
AB089824.1     0.030945  0.030976  0.024161  0.028472  0.027550  0.033540  0.029740  0.026920  0.029071  0.031211
 0.028749  0.030979  0.030532  0.027271  0.022536  0.025203  0.010343  0.000000  0.003365  0.000966  0.005133  0.
002588  0.001792  0.005261  0.007458  0.007404  0.003101  0.007008  0.004638  0.006450  0.006869  0.006868  0.
006741  0.008398  0.001231  0.001952  0.001272  0.002610  0.002928  0.003454  0.002362  0.002144  0.004148  0.
003731  0.002196  0.006641  0.005488  0.002681
AB074918.2     0.028636  0.028667  0.021852  0.026163  0.025241  0.031231  0.027431  0.024611  0.026762  0.028902
 0.026440  0.028670  0.028223  0.024962  0.020227  0.022894  0.006978  0.003365  0.000000  0.002791  0.002824  0.
005953  0.001573  0.003198  0.004093  0.004039  0.002832  0.003643  0.002734  0.003085  0.003504  0.003503  0.
006933  0.005033  0.003782  0.005317  0.004637  0.005975  0.006293  0.006819  0.005428  0.005215  0.007513  0.
007096  0.004884  0.008950  0.008549  0.006046
AB074920.3     0.030202  0.030233  0.023418  0.027729  0.026807  0.032797  0.028997  0.026177  0.028328  0.030468
 0.028006  0.030236  0.029789  0.026528  0.021793  0.024460  0.009769  0.000966  0.002791  0.000000  0.004390  0.
003162  0.001947  0.004865  0.006884  0.006830  0.004067  0.006434  0.004401  0.005876  0.006295  0.006294  0.
007707  0.007824  0.002028  0.002526  0.001846  0.003310  0.003665  0.004028  0.003159  0.002941  0.004722  0.
004305  0.002993  0.007384  0.006231  0.003255
AF082843.1     0.025812  0.025843  0.019028  0.023339  0.022417  0.028407  0.024607  0.021787  0.023938  0.026078
 0.023616  0.025846  0.025399  0.022138  0.017403  0.020070  0.007225  0.005133  0.002824  0.004390  0.000000  0.
007005  0.003821  0.004726  0.004379  0.004956  0.005504  0.003890  0.004262  0.003332  0.003751  0.003750  0.
009757  0.005280  0.004970  0.005753  0.005403  0.006441  0.008055  0.007570  0.005726  0.005853  0.008126  0.
007709  0.005796  0.011774  0.010621  0.007221
AF060668.1     0.032817  0.032848  0.026033  0.030344  0.029422  0.035412  0.031612  0.028792  0.030943  0.033083
 0.030621  0.032851  0.032404  0.029143  0.024408  0.027075  0.012931  0.002588  0.005953  0.003162  0.007005  0.
000000  0.004380  0.007849  0.010046  0.009992  0.003121  0.009596  0.007226  0.009038  0.009457  0.009456  0.
005320  0.010986  0.002171  0.001252  0.001602  0.000564  0.001151  0.000866  0.001279  0.001152  0.001560  0.
001143  0.001209  0.004769  0.003616  0.002019
AF060669.1     0.029633  0.029664  0.022849  0.027160  0.026238  0.032228  0.028428  0.025608  0.027759  0.029899
 0.027437  0.029667  0.029220  0.025959  0.021224  0.023891  0.008551  0.001792  0.001573  0.001947  0.003821  0.
004380  0.000000  0.003469  0.005666  0.005612  0.002120  0.005216  0.002846  0.004658  0.005077  0.005076  0.
005936  0.006606  0.002209  0.003744  0.003064  0.004402  0.004720  0.005246  0.003855  0.003642  0.005940  0.
005523  0.003311  0.007953  0.006976  0.004473
AY115488.1     0.027356  0.027387  0.020572  0.024883  0.023961  0.029951  0.026151  0.023331  0.025482  0.027622
 0.025160  0.027390  0.026943  0.023682  0.018947  0.021614  0.006132  0.005261  0.003198  0.004865  0.004726  0.
007849  0.003469  0.000000  0.002197  0.002143  0.004728  0.001948  0.000623  0.001953  0.001808  0.001814  0.
008213  0.005219  0.005678  0.007213  0.006533  0.007871  0.008189  0.008715  0.007324  0.007111  0.009409  0.
008992  0.006780  0.010230  0.010445  0.007942
AB189070.1     0.026203  0.026234  0.019419  0.023730  0.022808  0.028798  0.024998  0.022178  0.024329  0.026469
 0.024007  0.026237  0.025790  0.022529  0.017794  0.020461  0.005785  0.007458  0.004093  0.006884  0.004379  0.
010046  0.005666  0.002197  0.000000  0.000769  0.006925  0.001188  0.002820  0.001606  0.001328  0.001467  0.
009365  0.003828  0.007875  0.009410  0.008730  0.010068  0.010386  0.010912  0.009521  0.009308  0.011606  0.
011189  0.008977  0.012383  0.012642  0.010139
AB189071.1     0.026064  0.026095  0.019280  0.023591  0.022669  0.028659  0.024859  0.022039  0.024190  0.026330
 0.023868  0.026098  0.025651  0.022390  0.017655  0.020322  0.006362  0.007404  0.004039  0.006830  0.004956  0.
009992  0.005612  0.002143  0.000769  0.000000  0.006871  0.001765  0.002766  0.002183  0.001905  0.002044  0.
009505  0.004597  0.007821  0.009356  0.008676  0.010014  0.010332  0.010858  0.009467  0.009254  0.011552  0.
011135  0.008923  0.012330  0.012588  0.010085
AB091394.1     0.031316  0.031347  0.024532  0.028843  0.027921  0.033911  0.030111  0.027291  0.029442  0.031582
 0.029120  0.031350  0.030903  0.027642  0.022907  0.025574  0.009810  0.003101  0.002832  0.004067  0.005504  0.
003121  0.002120  0.004728  0.006925  0.006871  0.000000  0.006475  0.004105  0.005917  0.006336  0.006335  0.
004253  0.007865  0.002449  0.002799  0.003120  0.003143  0.003461  0.003987  0.002596  0.002383  0.004681  0.
004264  0.002052  0.006270  0.005717  0.003576
AB189072.1     0.025408  0.025439  0.018624  0.022935  0.022013  0.028003  0.024203  0.021383  0.023534  0.025674
 0.023212  0.025442  0.024995  0.021734  0.016999  0.019666  0.004597  0.007008  0.003643  0.006434  0.003890  0.
009596  0.005216  0.001948  0.001188  0.001765  0.006475  0.000000  0.002370  0.000558  0.000140  0.000279  0.
010161  0.003884  0.007425  0.008960  0.008280  0.009618  0.009936  0.010462  0.009071  0.008858  0.011156  0.
010739  0.008527  0.012178  0.012192  0.009689
AP003430.1     0.027585  0.027616  0.020801  0.025112  0.024190  0.030180  0.026380  0.023560  0.025711  0.027851
```

```
 0.025389  0.027619  0.027172  0.023911  0.019176  0.021843  0.005801  0.004638  0.002734  0.004401  0.004262  0.
007226  0.002846  0.000623  0.002820  0.002766  0.004105  0.002370  0.000000  0.001812  0.002231  0.002230  0.
007984  0.005149  0.005055  0.006590  0.005910  0.007248  0.007566  0.008092  0.006701  0.006488  0.008786  0.
008369  0.006157  0.010001  0.009822  0.007319
AB189073.1     0.025966  0.025997  0.019182  0.023493  0.022571  0.028561  0.024761  0.021941  0.024092  0.026232
 0.023770  0.026000  0.025553  0.022292  0.017557  0.020224  0.004182  0.006450  0.003085  0.005876  0.003332  0.
009038  0.004658  0.001953  0.001606  0.002183  0.005917  0.000558  0.001812  0.000000  0.000418  0.000418  0.
009603  0.003466  0.006867  0.008402  0.007722  0.009060  0.009378  0.009904  0.008513  0.008300  0.010598  0.
010181  0.007969  0.011620  0.011634  0.009131
AB189074.1     0.025548  0.025579  0.018764  0.023075  0.022153  0.028143  0.024343  0.021523  0.023674  0.025814
 0.023352  0.025582  0.025135  0.021874  0.017139  0.019806  0.004457  0.006869  0.003504  0.006295  0.003751  0.
009457  0.005077  0.001808  0.001328  0.001905  0.006336  0.000140  0.002231  0.000418  0.000000  0.000139  0.
010021  0.003745  0.007286  0.008821  0.008141  0.009479  0.009797  0.010323  0.008932  0.008719  0.011017  0.
010600  0.008388  0.012038  0.012053  0.009550
AB189075.1     0.025548  0.025579  0.018764  0.023075  0.022153  0.028143  0.024343  0.021523  0.023674  0.025814
 0.023352  0.025582  0.025135  0.021874  0.017139  0.019806  0.004318  0.006868  0.003503  0.006294  0.003750  0.
009456  0.005076  0.001814  0.001467  0.002044  0.006335  0.000279  0.002230  0.000418  0.000139  0.000000  0.
010021  0.003606  0.007285  0.008820  0.008140  0.009478  0.009796  0.010322  0.008931  0.008718  0.011016  0.
010599  0.008387  0.012038  0.012052  0.009549
AB073912.1     0.035569  0.035600  0.028785  0.033096  0.032174  0.038164  0.034364  0.031544  0.033695  0.035835
 0.033373  0.035603  0.035156  0.031895  0.027160  0.029827  0.013785  0.006741  0.006933  0.007707  0.009757  0.
005320  0.005936  0.008213  0.009365  0.009505  0.004253  0.010161  0.007984  0.009603  0.010021  0.010021  0.
000000  0.009129  0.006089  0.006439  0.006760  0.005531  0.004169  0.005217  0.005849  0.005727  0.005217  0.
005078  0.005401  0.004194  0.004453  0.007216
AF455784.1     0.027695  0.027726  0.020911  0.025222  0.024300  0.030290  0.026490  0.023670  0.025821  0.027961
 0.025499  0.027729  0.027282  0.024021  0.019286  0.021953  0.005911  0.008398  0.005033  0.007824  0.005280  0.
010986  0.006606  0.005219  0.003828  0.004597  0.007865  0.003884  0.005149  0.003466  0.003745  0.003606  0.
009129  0.000000  0.008815  0.010350  0.009670  0.011008  0.011326  0.011852  0.010461  0.010248  0.012546  0.
012129  0.009917  0.013324  0.013582  0.011079
AB097812.1     0.030782  0.030813  0.023998  0.028309  0.027387  0.033377  0.029577  0.026757  0.028908  0.031048
 0.028586  0.030816  0.030369  0.027108  0.022373  0.025040  0.010760  0.001231  0.003782  0.002028  0.004970  0.
002171  0.002209  0.005678  0.007875  0.007821  0.002449  0.007425  0.005055  0.006867  0.007286  0.007285  0.
006089  0.008815  0.000000  0.001535  0.000855  0.002193  0.003085  0.003037  0.001646  0.001433  0.003731  0.
003314  0.001102  0.006804  0.005651  0.002264
AB099347.1     0.031565  0.031596  0.024781  0.029092  0.028170  0.034160  0.030360  0.027540  0.029691  0.031831
 0.029369  0.031599  0.031152  0.027891  0.023156  0.025823  0.012295  0.001952  0.005317  0.002526  0.005753  0.
001252  0.003744  0.007213  0.009410  0.009356  0.002799  0.008960  0.006590  0.008402  0.008821  0.008820  0.
006439  0.010350  0.001535  0.000000  0.000680  0.000908  0.002302  0.001817  0.000729  0.000712  0.002373  0.
001956  0.001038  0.006021  0.004868  0.001516
AB080575.1     0.031215  0.031246  0.024431  0.028742  0.027820  0.033810  0.030010  0.027190  0.029341  0.031481
 0.029019  0.031249  0.030802  0.027541  0.022806  0.025473  0.011615  0.001272  0.004637  0.001846  0.005403  0.
001602  0.003064  0.006533  0.008730  0.008676  0.003120  0.008280  0.005910  0.007722  0.008141  0.008140  0.
006760  0.009670  0.000855  0.000680  0.000000  0.001530  0.002652  0.002182  0.001379  0.001161  0.002876  0.
002459  0.001359  0.006371  0.005218  0.001818
AB074915.3     0.032253  0.032284  0.025469  0.029780  0.028858  0.034848  0.031048  0.028228  0.030379  0.032519
 0.030057  0.032287  0.031840  0.028579  0.023844  0.026511  0.012953  0.002610  0.005975  0.003310  0.006441  0.
000564  0.004402  0.007871  0.010068  0.010014  0.003143  0.009618  0.007248  0.009060  0.009479  0.009478  0.
005531  0.011008  0.002193  0.000908  0.001530  0.000000  0.001614  0.001129  0.000715  0.000760  0.001685  0.
001268  0.001091  0.005333  0.004180  0.002396
AB074917.3     0.033867  0.033898  0.027083  0.031394  0.030472  0.036462  0.032662  0.029842  0.031993  0.034133
 0.031671  0.033901  0.033454  0.030193  0.025458  0.028125  0.013271  0.002928  0.006293  0.003665  0.008055  0.
001151  0.004720  0.008189  0.010386  0.010332  0.003461  0.009936  0.007566  0.009378  0.009797  0.009796  0.
004169  0.011326  0.003085  0.002302  0.002652  0.001614  0.000000  0.001048  0.002329  0.002202  0.001220  0.
000909  0.002259  0.003719  0.002566  0.003047
AB161717.1     0.033382  0.033413  0.026598  0.030909  0.029987  0.035977  0.032177  0.029357  0.031508  0.033648
 0.031186  0.033416  0.032969  0.029708  0.024973  0.027640  0.013797  0.003454  0.006819  0.004028  0.007570  0.
000866  0.005246  0.008715  0.010912  0.010858  0.003987  0.010462  0.008092  0.009904  0.010323  0.010322  0.
005217  0.011852  0.003037  0.001817  0.002182  0.001129  0.001048  0.000000  0.001844  0.001717  0.000694  0.
000278  0.001935  0.004204  0.003051  0.002423
AB091395.1     0.031538  0.031569  0.024754  0.029065  0.028143  0.034133  0.030333  0.027513  0.029664  0.031804
 0.029342  0.031572  0.031125  0.027864  0.023129  0.025796  0.012406  0.002362  0.005428  0.003159  0.005726  0.
001279  0.003855  0.007324  0.009521  0.009467  0.002596  0.009071  0.006701  0.008513  0.008932  0.008931  0.
```

```
005849  0.010461  0.001646  0.000729  0.001379  0.000715  0.002329  0.001844  0.000000  0.000218  0.002400  0.
001983  0.000544  0.006048  0.004895  0.002245
AB200239.1     0.031665  0.031696  0.024881  0.029192  0.028270  0.034260  0.030460  0.027640  0.029791  0.031931
 0.029469  0.031699  0.031252  0.027991  0.023256  0.025923  0.012193  0.002144  0.005215  0.002941  0.005853  0.
001152  0.003642  0.007111  0.009308  0.009254  0.002383  0.008858  0.006488  0.008300  0.008719  0.008718  0.
005727  0.010248  0.001433  0.000712  0.001161  0.000760  0.002202  0.001717  0.000218  0.000000  0.002298  0.
001881  0.000331  0.005921  0.004768  0.002027
AB161718.1     0.033938  0.033969  0.027154  0.031465  0.030543  0.036533  0.032733  0.029913  0.032064  0.034204
 0.031742  0.033972  0.033525  0.030264  0.025529  0.028196  0.014491  0.004148  0.007513  0.004722  0.008126  0.
001560  0.005940  0.009409  0.011606  0.011552  0.004681  0.011156  0.008786  0.010598  0.011017  0.011016  0.
005217  0.012546  0.003731  0.002373  0.002876  0.001685  0.001220  0.000694  0.002400  0.002298  0.000000  0.
000417  0.002629  0.003648  0.002495  0.002562
AB161719.1     0.033521  0.033552  0.026737  0.031048  0.030126  0.036116  0.032316  0.029496  0.031647  0.033787
 0.031325  0.033555  0.033108  0.029847  0.025112  0.027779  0.014074  0.003731  0.007096  0.004305  0.007709  0.
001143  0.005523  0.008992  0.011189  0.011135  0.004264  0.010739  0.008369  0.010181  0.010600  0.010599  0.
005078  0.012129  0.003314  0.001956  0.002459  0.001268  0.000909  0.000278  0.001983  0.001881  0.000417  0.
000000  0.002212  0.004065  0.002912  0.002701
AB097811.1     0.031608  0.031639  0.024824  0.029135  0.028213  0.034203  0.030403  0.027583  0.029734  0.031874
 0.029412  0.031642  0.031195  0.027934  0.023199  0.025866  0.011862  0.002196  0.004884  0.002993  0.005796  0.
001209  0.003311  0.006780  0.008977  0.008923  0.002052  0.008527  0.006157  0.007969  0.008388  0.008387  0.
005401  0.009917  0.001102  0.001038  0.001359  0.001091  0.002259  0.001935  0.000544  0.000331  0.002629  0.
002212  0.000000  0.005978  0.004825  0.002079
AY594199.1     0.037586  0.037617  0.030802  0.035113  0.034191  0.040181  0.036381  0.033561  0.035712  0.037852
 0.035390  0.037620  0.037173  0.033912  0.029177  0.031844  0.015802  0.006641  0.008950  0.007384  0.011774  0.
004769  0.007953  0.010230  0.012383  0.012330  0.006270  0.012178  0.010001  0.011620  0.012038  0.012038  0.
004194  0.013324  0.006804  0.006021  0.006371  0.005333  0.003719  0.004204  0.006048  0.005921  0.003648  0.
004065  0.005978  0.000000  0.001153  0.004553
AJ272108.1     0.036433  0.036464  0.029649  0.033960  0.033038  0.039028  0.035228  0.032408  0.034559  0.036699
 0.034237  0.036467  0.036020  0.032759  0.028024  0.030691  0.015527  0.005488  0.008549  0.006231  0.010621  0.
003616  0.006976  0.010445  0.012642  0.012588  0.005717  0.012192  0.009822  0.011634  0.012053  0.012052  0.
004453  0.013582  0.005651  0.004868  0.005218  0.004180  0.002566  0.003051  0.004895  0.004768  0.002495  0.
002912  0.004825  0.001153  0.000000  0.003637
AB108537.1     0.033033  0.033064  0.026249  0.030560  0.029638  0.035628  0.031828  0.029008  0.031159  0.033299
 0.030837  0.033067  0.032620  0.029359  0.024624  0.027291  0.013024  0.002681  0.006046  0.003255  0.007221  0.
002019  0.004473  0.007942  0.010139  0.010085  0.003576  0.009689  0.007319  0.009131  0.009550  0.009549  0.
007216  0.011079  0.002264  0.001516  0.001818  0.002396  0.003047  0.002423  0.002245  0.002027  0.002562  0.
002701  0.002079  0.004553  0.003637  0.000000


24 Eutherian mammal sequences

    24
X58533.1_R     0.000000  0.014391  0.039530  0.014631  0.026769  0.002626  0.014092  0.014741  0.008869  0.016469
 0.009505  0.044183  0.058718  0.036920  0.071142  0.025380  0.023122  0.023122  0.020545  0.023291  0.013332  0.
026981  0.024616  0.021426
S95936.1_t     0.014391  0.000000  0.028169  0.014493  0.014893  0.015052  0.027967  0.028158  0.022744  0.030344
 0.021328  0.032822  0.047357  0.025044  0.059266  0.015217  0.017347  0.017757  0.014892  0.015092  0.022477  0.
015456  0.012740  0.014888
U02564.1_B     0.039530  0.028169  0.000000  0.038987  0.030085  0.041339  0.052226  0.054271  0.039919  0.049621
 0.049035  0.015419  0.019188  0.017977  0.045601  0.032069  0.034199  0.034609  0.031744  0.031944  0.039329  0.
032308  0.032925  0.031740
D38380.1_R     0.014631  0.014493  0.038987  0.000000  0.012138  0.017257  0.022401  0.017781  0.019049  0.015851
 0.011447  0.043640  0.058175  0.030689  0.056511  0.010749  0.014649  0.010133  0.008937  0.012001  0.013370  0.
012350  0.009985  0.008507
NM_008522.     0.026769  0.014893  0.030085  0.012138  0.000000  0.029395  0.034305  0.029919  0.023869  0.022508
 0.023585  0.031766  0.046301  0.018815  0.046649  0.003614  0.010536  0.006431  0.006224  0.005465  0.014894  0.
003846  0.005812  0.005343
NM_002343.     0.002626  0.015052  0.041339  0.017257  0.029395  0.000000  0.014247  0.014438  0.009024  0.016624
 0.007696  0.045992  0.060527  0.039546  0.073768  0.028006  0.025748  0.025748  0.023171  0.025917  0.015958  0.
029607  0.027242  0.024052
X78902.1_C     0.014092  0.027967  0.052226  0.022401  0.034305  0.014247  0.000000  0.006240  0.012305  0.015824
 0.014168  0.056879  0.071414  0.044456  0.078678  0.032916  0.030658  0.030658  0.028081  0.030827  0.020868  0.
```

```
034517  0.032152  0.028962
AJ005203.1      0.014741  0.028158  0.054271  0.017781  0.029919  0.014438  0.006240  0.000000  0.014350  0.011438
 0.007928  0.058924  0.073459  0.045973  0.074292  0.028530  0.029933  0.026272  0.024221  0.027285  0.019371  0.
030131  0.027766  0.024576
X57084.1_B      0.008869  0.022744  0.039919  0.019049  0.023869  0.009024  0.012305  0.014350  0.000000  0.009699
 0.010816  0.044572  0.059107  0.034020  0.068242  0.022480  0.020222  0.020222  0.017645  0.020391  0.010432  0.
024081  0.026110  0.018526
AJ131674.1      0.016469  0.030344  0.049621  0.015851  0.022508  0.016624  0.015824  0.011438  0.009699  0.000000
 0.009016  0.054274  0.068809  0.041323  0.062854  0.018894  0.025283  0.020767  0.019571  0.022635  0.014721  0.
018693  0.018989  0.019141
M92089.1_S      0.009505  0.021328  0.049035  0.011447  0.023585  0.007696  0.014168  0.007928  0.010816  0.009016
 0.000000  0.053688  0.068223  0.040737  0.067958  0.022196  0.024697  0.020181  0.018985  0.022049  0.014135  0.
023797  0.021432  0.018555
AF092510.1      0.044183  0.032822  0.015419  0.043640  0.031766  0.045992  0.056879  0.058924  0.044572  0.054274
 0.053688  0.000000  0.014583  0.018869  0.033247  0.035380  0.035091  0.035501  0.034703  0.032836  0.040221  0.
035612  0.037578  0.035133
X54530.1_X      0.058718  0.047357  0.019188  0.058175  0.046301  0.060527  0.071414  0.073459  0.059107  0.068809
 0.068223  0.014583  0.000000  0.033452  0.045693  0.049915  0.049674  0.050084  0.049238  0.047419  0.054804  0.
050147  0.052113  0.049668
D88801.1_P      0.036920  0.025044  0.017977  0.030689  0.018815  0.039546  0.044456  0.045973  0.034020  0.041323
 0.040737  0.018869  0.033452  0.000000  0.043205  0.022429  0.016222  0.020556  0.021752  0.018688  0.026602  0.
022661  0.024627  0.022182
AH008271.2      0.071142  0.059266  0.045601  0.056511  0.046649  0.073768  0.078678  0.074292  0.068242  0.062854
 0.067958  0.033247  0.045693  0.043205  0.000000  0.046407  0.057185  0.053080  0.051595  0.052114  0.061543  0.
044814  0.046526  0.050280
L20313.1_S      0.025380  0.015217  0.032069  0.010749  0.003614  0.028006  0.032916  0.028530  0.022480  0.018894
 0.022196  0.035380  0.049915  0.022429  0.046407  0.000000  0.010778  0.006673  0.005188  0.005707  0.015136  0.
001600  0.005295  0.003954
D89091.1_S      0.023122  0.017347  0.034199  0.014649  0.010536  0.025748  0.030658  0.029933  0.020222  0.025283
 0.024697  0.035091  0.049674  0.016222  0.057185  0.010778  0.000000  0.004516  0.005712  0.005071  0.010562  0.
012371  0.016073  0.006905
D89088.1_S      0.023122  0.017757  0.034609  0.010133  0.006431  0.025748  0.030658  0.026272  0.020222  0.020767
 0.020181  0.035501  0.050084  0.020556  0.053080  0.006673  0.004516  0.000000  0.002865  0.002665  0.009790  0.
008266  0.011968  0.002869
D89090.1_S      0.020545  0.014892  0.031744  0.008937  0.006224  0.023171  0.028081  0.024221  0.017645  0.019571
 0.018985  0.034703  0.049238  0.021752  0.051595  0.005188  0.005712  0.002865  0.000000  0.003064  0.009948  0.
006781  0.010483  0.001315
D89089.1_S      0.023291  0.015092  0.031944  0.012001  0.005465  0.025917  0.030827  0.027285  0.020391  0.022635
 0.022049  0.032836  0.047419  0.018688  0.052114  0.005707  0.005071  0.002665  0.003064  0.000000  0.009959  0.
007300  0.011002  0.003494
D89086.2_O      0.013332  0.022477  0.039329  0.013370  0.014894  0.015958  0.020868  0.019371  0.010432  0.014721
 0.014135  0.040221  0.054804  0.026602  0.061543  0.015136  0.010562  0.009790  0.009948  0.009959  0.000000  0.
016729  0.020431  0.011263
D89083.1_O      0.026981  0.015456  0.032308  0.012350  0.003846  0.029607  0.034517  0.030131  0.024081  0.018693
 0.023797  0.035612  0.050147  0.022661  0.044814  0.001600  0.012371  0.008266  0.006781  0.007300  0.016729  0.
000000  0.004101  0.005555
D89084.1_O      0.024616  0.012740  0.032925  0.009985  0.005812  0.027242  0.032152  0.027766  0.026110  0.018989
 0.021432  0.037578  0.052113  0.024627  0.046526  0.005295  0.016073  0.011968  0.010483  0.011002  0.020431  0.
004101  0.000000  0.009168
D89085.1_O      0.021426  0.014888  0.031740  0.008507  0.005343  0.024052  0.028962  0.024576  0.018526  0.019141
 0.018555  0.035133  0.049668  0.022182  0.050280  0.003954  0.006905  0.002869  0.001315  0.003494  0.011263  0.
005555  0.009168  0.000000


58 genome datasets from different species

    58
V00662.1_H      0.000000  0.005647  0.004698  0.005499  0.007525  0.006900  0.010812  0.012034  0.015085  0.084152
 0.085775  0.085933  0.085828  0.085775  0.104468  0.104667  0.104945  0.105014  0.104424  0.112568  0.112586  0.
112617  0.112608  0.112709  0.112578  0.112571  0.112611  0.112598  0.112645  0.138241  0.140015  0.137790  0.
138763  0.140969  0.141050  0.137940  0.136992  0.135387  0.137783  0.139598  0.159249  0.153439  0.153723  0.
144747  0.135197  0.136078  0.133332  0.133501  0.132922  0.160170  0.120456  0.143490  0.188915  0.189660  0.
```

```
189417  0.191418  0.184313  0.188692
D38116.1_P     0.005647  0.000000  0.001945  0.004966  0.013172  0.004251  0.006713  0.017478  0.020529  0.078708
 0.080331  0.080489  0.080384  0.080331  0.099024  0.099223  0.099501  0.099570  0.098980  0.107124  0.107142  0.
107173  0.107164  0.107265  0.107134  0.107127  0.107167  0.107154  0.107201  0.142341  0.144115  0.141890  0.
142863  0.145069  0.145150  0.142040  0.141092  0.139487  0.141883  0.143698  0.163551  0.157741  0.158025  0.
149049  0.139499  0.140380  0.137634  0.137803  0.127478  0.154726  0.115012  0.138046  0.193217  0.193962  0.
193719  0.195720  0.188615  0.192994
D38113.1_P     0.004698  0.001945  0.000000  0.003145  0.012223  0.003105  0.008658  0.016404  0.019455  0.079781
 0.081404  0.081562  0.081457  0.081404  0.100097  0.100296  0.100574  0.100643  0.100053  0.108197  0.108215  0.
108246  0.108237  0.108338  0.108207  0.108200  0.108240  0.108227  0.108274  0.140396  0.142170  0.139945  0.
140918  0.143124  0.143205  0.140095  0.139147  0.137542  0.139938  0.141753  0.161730  0.155920  0.156204  0.
147228  0.137678  0.138559  0.135813  0.135982  0.128551  0.155799  0.116085  0.139119  0.191396  0.192141  0.
191898  0.193899  0.186794  0.191173
D38114.1_G     0.005499  0.004966  0.003145  0.000000  0.012396  0.005295  0.010848  0.017533  0.020584  0.078653
 0.080276  0.080434  0.080329  0.080276  0.098969  0.099168  0.099446  0.099515  0.098925  0.107069  0.107087  0.
107118  0.107109  0.107210  0.107079  0.107072  0.107112  0.107099  0.107146  0.138206  0.139980  0.137755  0.
138728  0.140934  0.141015  0.137905  0.136957  0.135352  0.137748  0.139563  0.158585  0.152775  0.153059  0.
144083  0.134533  0.135414  0.132668  0.132837  0.127423  0.154671  0.114957  0.137991  0.188251  0.188996  0.
188753  0.190754  0.183649  0.188028
X99256.1_H     0.007525  0.013172  0.012223  0.012396  0.000000  0.012080  0.015221  0.006854  0.009905  0.089332
 0.090955  0.091113  0.091008  0.090955  0.109648  0.109847  0.110125  0.110194  0.109604  0.117748  0.117766  0.
117797  0.117788  0.117889  0.117758  0.117751  0.117791  0.117778  0.117825  0.134964  0.136738  0.134513  0.
135486  0.137692  0.137773  0.134663  0.133715  0.132110  0.134506  0.136321  0.153627  0.147817  0.148101  0.
139125  0.129575  0.130456  0.127710  0.127879  0.138102  0.165350  0.125636  0.148670  0.183293  0.184038  0.
183795  0.185796  0.178691  0.183070
Y18001.1_P     0.006900  0.004251  0.003105  0.005295  0.012080  0.000000  0.006212  0.018934  0.021985  0.077252
 0.078875  0.079033  0.078928  0.078875  0.097568  0.097767  0.098045  0.098114  0.097524  0.105668  0.105686  0.
105717  0.105708  0.105809  0.105678  0.105671  0.105711  0.105698  0.105745  0.143501  0.145275  0.143050  0.
144023  0.146229  0.146310  0.143200  0.142252  0.140647  0.143043  0.144858  0.159300  0.153490  0.153774  0.
144798  0.135248  0.136129  0.133383  0.133552  0.126022  0.153270  0.113556  0.136590  0.188966  0.189711  0.
189468  0.191469  0.184364  0.188743
AY863426.1     0.010812  0.006713  0.008658  0.010848  0.015221  0.006212  0.000000  0.022075  0.025126  0.074111
 0.075734  0.075892  0.075787  0.075734  0.094427  0.094626  0.094904  0.094973  0.094383  0.102527  0.102545  0.
102576  0.102567  0.102668  0.102537  0.102530  0.102570  0.102557  0.102604  0.149054  0.150828  0.148603  0.
149576  0.151782  0.151863  0.148753  0.147805  0.146200  0.148596  0.150411  0.165512  0.159702  0.159986  0.
151010  0.141460  0.142341  0.139595  0.139764  0.122881  0.150129  0.116647  0.135121  0.195178  0.195923  0.
195680  0.197681  0.190576  0.194955
D38115.1_P     0.012034  0.017478  0.016404  0.017533  0.006854  0.018934  0.022075  0.000000  0.003051  0.096186
 0.097809  0.097967  0.097862  0.097809  0.116502  0.116701  0.116979  0.117048  0.116458  0.124602  0.124620  0.
124651  0.124642  0.124743  0.124612  0.124605  0.124645  0.124632  0.124679  0.134561  0.136335  0.134110  0.
135083  0.137289  0.137370  0.134260  0.133312  0.131707  0.134103  0.135918  0.158298  0.152488  0.152772  0.
143796  0.134246  0.135127  0.132381  0.132550  0.144956  0.172204  0.132490  0.155524  0.187964  0.188709  0.
188466  0.190467  0.183362  0.187741
NC_002083.     0.015085  0.020529  0.019455  0.020584  0.009905  0.021985  0.025126  0.003051  0.000000  0.099237
 0.100860  0.101018  0.100913  0.100860  0.119553  0.119752  0.120030  0.120099  0.119509  0.127653  0.127671  0.
127702  0.127693  0.127794  0.127663  0.127656  0.127696  0.127683  0.127730  0.133977  0.135751  0.133526  0.
134499  0.136705  0.136786  0.133676  0.132728  0.131123  0.133519  0.135334  0.158692  0.152882  0.153166  0.
144190  0.134640  0.135521  0.132775  0.132944  0.148007  0.175255  0.135541  0.158575  0.188358  0.189103  0.
188860  0.190861  0.183756  0.188135
FJ217161.1     0.084152  0.078708  0.079781  0.078653  0.089332  0.077252  0.074111  0.096186  0.099237  0.000000
 0.001623  0.001781  0.001676  0.001623  0.020316  0.020515  0.020793  0.020862  0.020272  0.042229  0.042552  0.
042326  0.042082  0.042183  0.042447  0.042441  0.042447  0.042453  0.042480  0.143934  0.145708  0.143483  0.
144456  0.146662  0.146743  0.143633  0.142685  0.141080  0.143476  0.145291  0.098747  0.092937  0.093221  0.
084245  0.074695  0.075576  0.072830  0.072999  0.062766  0.076018  0.069410  0.068356  0.128413  0.129158  0.
128915  0.130916  0.123811  0.128190
KC545393.1     0.085775  0.080331  0.081404  0.080276  0.090955  0.078875  0.075734  0.097809  0.100860  0.001623
 0.000000  0.000159  0.000053  0.000053  0.018693  0.018892  0.019170  0.019239  0.018649  0.041370  0.041693  0.
041467  0.041223  0.041324  0.041588  0.041582  0.041588  0.041594  0.041621  0.144481  0.146255  0.144030  0.
145003  0.147209  0.147290  0.144180  0.143232  0.141627  0.144023  0.145838  0.098531  0.092721  0.093005  0.
084029  0.074479  0.075360  0.072614  0.072783  0.063313  0.074395  0.069957  0.068140  0.128197  0.128942  0.
128699  0.130700  0.123595  0.127974
KC545395.1     0.085933  0.080489  0.081562  0.080434  0.091113  0.079033  0.075892  0.097967  0.101018  0.001781
```

```
 0.000159  0.000000  0.000159  0.000159  0.018535  0.018734  0.019012  0.019081  0.018491  0.041211  0.041534  0.
041308  0.041064  0.041165  0.041429  0.041423  0.041429  0.041435  0.041462  0.144534  0.146308  0.144083  0.
145056  0.147262  0.147343  0.144233  0.143285  0.141680  0.144076  0.145891  0.098584  0.092774  0.093058  0.
084082  0.074532  0.075413  0.072667  0.072836  0.063366  0.074237  0.070010  0.068193  0.128250  0.128995  0.
128752  0.130753  0.123648  0.128027
KC545394.1     0.085828  0.080384  0.081457  0.080329  0.091008  0.078928  0.075787  0.097862  0.100913  0.001676
 0.000053  0.000159  0.000000  0.000106  0.018640  0.018839  0.019117  0.019186  0.018596  0.041370  0.041693  0.
041467  0.041223  0.041324  0.041588  0.041582  0.041588  0.041594  0.041621  0.144481  0.146255  0.144030  0.
145003  0.147209  0.147290  0.144180  0.143232  0.141627  0.144023  0.145838  0.098478  0.092668  0.092952  0.
083976  0.074426  0.075307  0.072561  0.072730  0.063313  0.074342  0.069957  0.068087  0.128144  0.128889  0.
128646  0.130647  0.123542  0.127921
KC545396.1     0.085775  0.080331  0.081404  0.080276  0.090955  0.078875  0.075734  0.097809  0.100860  0.001623
 0.000053  0.000159  0.000106  0.000000  0.018693  0.018892  0.019170  0.019239  0.018649  0.041370  0.041693  0.
041467  0.041223  0.041324  0.041588  0.041582  0.041588  0.041594  0.041621  0.144534  0.146308  0.144083  0.
145056  0.147262  0.147343  0.144233  0.143285  0.141680  0.144076  0.145891  0.098584  0.092774  0.093058  0.
084082  0.074532  0.075413  0.072667  0.072836  0.063366  0.074395  0.070010  0.068193  0.128250  0.128995  0.
128752  0.130753  0.123648  0.128027
AF522874.1     0.104468  0.099024  0.100097  0.098969  0.109648  0.097568  0.094427  0.116502  0.119553  0.020316
 0.018693  0.018535  0.018640  0.018693  0.000000  0.000798  0.000477  0.001002  0.001154  0.029509  0.029893  0.
029366  0.029244  0.029244  0.029771  0.029916  0.029771  0.029806  0.029771  0.143324  0.145098  0.142873  0.
143846  0.146052  0.146133  0.143023  0.142075  0.140470  0.142866  0.144681  0.092220  0.086410  0.086694  0.
077718  0.068168  0.069049  0.066303  0.066472  0.062156  0.055702  0.068800  0.061829  0.121886  0.122631  0.
122388  0.124389  0.117284  0.121663
AB050936.1     0.104667  0.099223  0.100296  0.099168  0.109847  0.097767  0.094626  0.116701  0.119752  0.020515
 0.018892  0.018734  0.018839  0.018892  0.000798  0.000000  0.000405  0.000347  0.000700  0.028998  0.029382  0.
028855  0.028733  0.028733  0.029260  0.029405  0.029260  0.029295  0.029260  0.142813  0.144587  0.142362  0.
143335  0.145541  0.145622  0.142512  0.141564  0.139959  0.142355  0.144170  0.091422  0.085612  0.085896  0.
076920  0.067370  0.068251  0.065505  0.065674  0.061645  0.055503  0.068289  0.061031  0.121088  0.121833  0.
121590  0.123591  0.116486  0.120865
JX477166.1     0.104945  0.099501  0.100574  0.099446  0.110125  0.098045  0.094904  0.116979  0.120030  0.020793
 0.019170  0.019012  0.019117  0.019170  0.000477  0.000405  0.000000  0.000526  0.000894  0.029403  0.029787  0.
029260  0.029138  0.029138  0.029665  0.029810  0.029665  0.029700  0.029665  0.143218  0.144992  0.142767  0.
143740  0.145946  0.146027  0.142917  0.141969  0.140364  0.142760  0.144575  0.091744  0.085934  0.086218  0.
077242  0.067692  0.068573  0.065827  0.065996  0.062050  0.055225  0.068694  0.061353  0.121410  0.122155  0.
121912  0.123913  0.116808  0.121187
FJ621585.1     0.105014  0.099570  0.100643  0.099515  0.110194  0.098114  0.094973  0.117048  0.120099  0.020862
 0.019239  0.019081  0.019186  0.019239  0.001002  0.000347  0.000526  0.000000  0.000713  0.029128  0.029512  0.
028985  0.028863  0.028863  0.029390  0.029535  0.029390  0.029425  0.029390  0.142943  0.144717  0.142492  0.
143465  0.145671  0.145752  0.142642  0.141694  0.140089  0.142485  0.144300  0.091218  0.085408  0.085692  0.
076716  0.067166  0.068047  0.065301  0.065470  0.061775  0.055156  0.068419  0.060827  0.120884  0.121629  0.
121386  0.123387  0.116282  0.120661
FJ621583.1     0.104424  0.098980  0.100053  0.098925  0.109604  0.097524  0.094383  0.116458  0.119509  0.020272
 0.018649  0.018491  0.018596  0.018649  0.001154  0.000700  0.000894  0.000713  0.000000  0.029099  0.029483  0.
028956  0.028834  0.028834  0.029361  0.029506  0.029361  0.029396  0.029361  0.142914  0.144688  0.142463  0.
143436  0.145642  0.145723  0.142613  0.141665  0.140060  0.142456  0.144271  0.091066  0.085256  0.085540  0.
076564  0.067014  0.067895  0.065149  0.065318  0.061746  0.055746  0.068390  0.060675  0.120732  0.121477  0.
121234  0.123235  0.116130  0.120509
AY278488.2     0.112568  0.107124  0.108197  0.107069  0.117748  0.105668  0.102527  0.124602  0.127653  0.042229
 0.041370  0.041211  0.041370  0.041370  0.029509  0.028998  0.029403  0.029128  0.029099  0.000000  0.000384  0.
000189  0.000265  0.000265  0.000262  0.000407  0.000262  0.000297  0.000262  0.121003  0.121034  0.114219  0.
118530  0.117608  0.123598  0.119798  0.116978  0.119129  0.121269  0.118807  0.091967  0.086068  0.096884  0.
076915  0.079942  0.081257  0.081776  0.086847  0.035506  0.047602  0.039291  0.052051  0.112175  0.114112  0.
113101  0.114611  0.113835  0.111885
AY278741.1     0.112586  0.107142  0.108215  0.107087  0.117766  0.105686  0.102545  0.124620  0.127671  0.042552
 0.041693  0.041534  0.041693  0.041693  0.029893  0.029382  0.029787  0.029512  0.029483  0.000384  0.000000  0.
000527  0.000649  0.000649  0.000122  0.000117  0.000122  0.000099  0.000122  0.121021  0.121052  0.114237  0.
118548  0.117626  0.123616  0.119816  0.116996  0.119147  0.121287  0.118825  0.092290  0.086391  0.097207  0.
077238  0.080265  0.081580  0.082099  0.087170  0.035425  0.047584  0.038907  0.051970  0.112498  0.114435  0.
113424  0.114530  0.114158  0.111949
AY278491.2     0.112617  0.107173  0.108246  0.107118  0.117797  0.105717  0.102576  0.124651  0.127702  0.042326
 0.041467  0.041308  0.041467  0.041467  0.029366  0.028855  0.029260  0.028985  0.028956  0.000189  0.000527  0.
000000  0.000244  0.000143  0.000405  0.000550  0.000405  0.000440  0.000405  0.121052  0.121083  0.114268  0.
```

```
118579  0.117657  0.123647  0.119847  0.117027  0.119178  0.121318  0.118856  0.092064  0.086165  0.096981  0.
077012  0.080039  0.081354  0.081873  0.086944  0.035695  0.047553  0.039434  0.052240  0.112297  0.114209  0.
113198  0.114800  0.113932  0.112074
AY278554.2     0.112608  0.107164  0.108237  0.107109  0.117788  0.105708  0.102567  0.124642  0.127693  0.042082
 0.041223  0.041064  0.041223  0.041223  0.029244  0.028733  0.029138  0.028863  0.028834  0.000265  0.000649  0.
000244  0.000000  0.000101  0.000527  0.000672  0.000527  0.000562  0.000527  0.121043  0.121074  0.114259  0.
118570  0.117648  0.123638  0.119838  0.117018  0.119169  0.121309  0.118847  0.091820  0.085921  0.096737  0.
076768  0.079795  0.081110  0.081629  0.086700  0.035583  0.047562  0.039556  0.052128  0.112185  0.113965  0.
112954  0.114688  0.113688  0.111962
AY282752.2     0.112709  0.107265  0.108338  0.107210  0.117889  0.105809  0.102668  0.124743  0.127794  0.042183
 0.041324  0.041165  0.041324  0.041324  0.029244  0.028733  0.029138  0.028863  0.028834  0.000265  0.000649  0.
000143  0.000101  0.000000  0.000527  0.000672  0.000527  0.000562  0.000527  0.121144  0.121175  0.114360  0.
118671  0.117749  0.123739  0.119939  0.117119  0.119270  0.121410  0.118948  0.091921  0.086022  0.096838  0.
076869  0.079896  0.081211  0.081730  0.086801  0.035583  0.047461  0.039556  0.052128  0.112185  0.114066  0.
113055  0.114688  0.113789  0.111962
AY283794.1     0.112578  0.107134  0.108207  0.107079  0.117758  0.105678  0.102537  0.124612  0.127663  0.042447
 0.041588  0.041429  0.041588  0.041588  0.029771  0.029260  0.029665  0.029390  0.029361  0.000262  0.000122  0.
000405  0.000527  0.000527  0.000000  0.000145  0.000034  0.000049  0.000067  0.121013  0.121044  0.114229  0.
118540  0.117618  0.123608  0.119808  0.116988  0.119139  0.121279  0.118817  0.092185  0.086286  0.097102  0.
077133  0.080160  0.081475  0.081994  0.087065  0.035451  0.047592  0.039029  0.051996  0.112393  0.114330  0.
113319  0.114556  0.114053  0.111844
AY283795.1     0.112571  0.107127  0.108200  0.107072  0.117751  0.105671  0.102530  0.124605  0.127656  0.042441
 0.041582  0.041423  0.041582  0.041582  0.029916  0.029405  0.029810  0.029535  0.029506  0.000407  0.000117  0.
000550  0.000672  0.000672  0.000145  0.000000  0.000145  0.000110  0.000145  0.121006  0.121037  0.114222  0.
118533  0.117611  0.123601  0.119801  0.116981  0.119132  0.121272  0.118810  0.092179  0.086280  0.097096  0.
077127  0.080154  0.081469  0.081988  0.087059  0.035308  0.047599  0.038884  0.051853  0.112387  0.114324  0.
113313  0.114413  0.114047  0.111838
AY283796.1     0.112611  0.107167  0.108240  0.107112  0.117791  0.105711  0.102570  0.124645  0.127696  0.042447
 0.041588  0.041429  0.041588  0.041588  0.029771  0.029260  0.029665  0.029390  0.029361  0.000262  0.000122  0.
000405  0.000527  0.000527  0.000034  0.000145  0.000000  0.000035  0.000034  0.121046  0.121077  0.114262  0.
118573  0.117651  0.123641  0.119841  0.117021  0.119172  0.121312  0.118850  0.092185  0.086286  0.097102  0.
077133  0.080160  0.081475  0.081994  0.087065  0.035417  0.047559  0.039029  0.051962  0.112393  0.114330  0.
113319  0.114522  0.114053  0.111844
AY283797.1     0.112598  0.107154  0.108227  0.107099  0.117778  0.105698  0.102557  0.124632  0.127683  0.042453
 0.041594  0.041435  0.041594  0.041594  0.029806  0.029295  0.029700  0.029425  0.029396  0.000297  0.000099  0.
000440  0.000562  0.000562  0.000049  0.000110  0.000035  0.000000  0.000047  0.121033  0.121064  0.114249  0.
118560  0.117638  0.123628  0.119828  0.117008  0.119159  0.121299  0.118837  0.092191  0.086292  0.097108  0.
077139  0.080166  0.081481  0.082000  0.087071  0.035402  0.047572  0.038994  0.051947  0.112399  0.114336  0.
113325  0.114507  0.114059  0.111850
AY283798.2     0.112645  0.107201  0.108274  0.107146  0.117825  0.105745  0.102604  0.124679  0.127730  0.042480
 0.041621  0.041462  0.041621  0.041621  0.029771  0.029260  0.029665  0.029390  0.029361  0.000262  0.000122  0.
000405  0.000527  0.000527  0.000067  0.000145  0.000034  0.000047  0.000000  0.121080  0.121111  0.114296  0.
118607  0.117685  0.123675  0.119875  0.117055  0.119206  0.121346  0.118884  0.092218  0.086319  0.097135  0.
077166  0.080193  0.081508  0.082027  0.087098  0.035417  0.047525  0.039029  0.051962  0.112426  0.114363  0.
113352  0.114522  0.114086  0.111877
M73218.1_H     0.138241  0.142341  0.140396  0.138206  0.134964  0.143501  0.149054  0.134561  0.133977  0.143934
 0.144481  0.144534  0.144481  0.144534  0.143324  0.142813  0.143218  0.142943  0.142914  0.121003  0.121021  0.
121052  0.121043  0.121144  0.121013  0.121006  0.121046  0.121033  0.121080  0.000000  0.001774  0.006784  0.
003638  0.003889  0.002809  0.001205  0.004025  0.002853  0.000970  0.002667  0.073114  0.065418  0.074531  0.
086284  0.083973  0.084403  0.087467  0.090115  0.141357  0.168605  0.128891  0.151925  0.084805  0.086083  0.
085750  0.087511  0.086384  0.085743
D10330.1_H     0.140015  0.144115  0.142170  0.139980  0.136738  0.145275  0.150828  0.136335  0.135751  0.145708
 0.146255  0.146308  0.146255  0.146308  0.145098  0.144587  0.144992  0.144717  0.144688  0.121034  0.121052  0.
121083  0.121074  0.121175  0.121044  0.121037  0.121077  0.121064  0.121111  0.001774  0.000000  0.006815  0.
002504  0.003426  0.002564  0.002075  0.004056  0.004627  0.002232  0.002227  0.074888  0.067192  0.076305  0.
088058  0.085747  0.086177  0.089241  0.091889  0.141388  0.168636  0.128922  0.151956  0.084836  0.086114  0.
085781  0.087542  0.087300  0.085774
X99441.1_H     0.137790  0.141890  0.139945  0.137755  0.134513  0.143050  0.148603  0.134110  0.133526  0.143483
 0.144030  0.144083  0.144030  0.144083  0.142873  0.142362  0.142767  0.142492  0.142463  0.114219  0.114237  0.
114268  0.114259  0.114360  0.114229  0.114222  0.114262  0.114249  0.114296  0.006784  0.006815  0.000000  0.
004311  0.003389  0.009379  0.005579  0.002759  0.004910  0.007050  0.004588  0.072663  0.064967  0.074080  0.
085833  0.083522  0.083952  0.087016  0.089664  0.134573  0.161821  0.122107  0.145141  0.078021  0.079704  0.
```

```
078966  0.080727  0.085075  0.078959
AF076239.3     0.138763  0.142863  0.140918  0.138728  0.135486  0.144023  0.149576  0.135083  0.134499  0.144456
 0.145003  0.145056  0.145003  0.145056  0.143846  0.143335  0.143740  0.143465  0.143436  0.118530  0.118548  0.
118579  0.118570  0.118671  0.118540  0.118533  0.118573  0.118560  0.118607  0.003638  0.002504  0.004311  0.
000000  0.002876  0.005068  0.003742  0.001771  0.003837  0.003392  0.001530  0.073636  0.065940  0.075053  0.
086806  0.084495  0.084925  0.087989  0.090637  0.138884  0.166132  0.126418  0.149452  0.082332  0.083610  0.
083277  0.085038  0.086048  0.083270
AF051830.1     0.140969  0.145069  0.143124  0.140934  0.137692  0.146229  0.151782  0.137289  0.136705  0.146662
 0.147209  0.147262  0.147209  0.147262  0.146052  0.145541  0.145946  0.145671  0.145642  0.117608  0.117626  0.
117657  0.117648  0.117749  0.117618  0.117611  0.117651  0.117638  0.117685  0.003889  0.003426  0.003389  0.
002876  0.000000  0.005990  0.003993  0.003977  0.005581  0.003661  0.001371  0.075842  0.068146  0.077259  0.
089012  0.086701  0.087131  0.090195  0.092843  0.137962  0.165210  0.125496  0.148530  0.081410  0.082883  0.
082355  0.084116  0.088254  0.082348
AF185822.1     0.141050  0.145150  0.143205  0.141015  0.137773  0.146310  0.151863  0.137370  0.136786  0.146743
 0.147290  0.147343  0.147290  0.147343  0.146133  0.145622  0.146027  0.145752  0.145723  0.123598  0.123616  0.
123647  0.123638  0.123739  0.123608  0.123601  0.123641  0.123628  0.123675  0.002809  0.002564  0.009379  0.
005068  0.005990  0.000000  0.003800  0.006620  0.005662  0.003267  0.004791  0.075923  0.068227  0.077340  0.
089093  0.086782  0.087212  0.090276  0.092924  0.143952  0.171200  0.131486  0.154520  0.087400  0.088678  0.
088345  0.090106  0.088979  0.088338
AF459438.1     0.137940  0.142040  0.140095  0.137905  0.134663  0.143200  0.148753  0.134260  0.133676  0.143633
 0.144180  0.144233  0.144180  0.144233  0.143023  0.142512  0.142917  0.142642  0.142613  0.119798  0.119816  0.
119847  0.119838  0.119939  0.119808  0.119801  0.119841  0.119828  0.119875  0.001205  0.002075  0.005579  0.
003742  0.003993  0.003800  0.000000  0.002820  0.002552  0.001978  0.002771  0.072813  0.065117  0.074230  0.
085983  0.083672  0.084102  0.087166  0.089814  0.140152  0.167400  0.127686  0.150720  0.083600  0.084878  0.
084545  0.086306  0.085225  0.084538
D11092.1_H     0.136992  0.141092  0.139147  0.136957  0.133715  0.142252  0.147805  0.133312  0.132728  0.142685
 0.143232  0.143285  0.143232  0.143285  0.142075  0.141564  0.141969  0.141694  0.141665  0.116978  0.116996  0.
117027  0.117018  0.117119  0.116988  0.116981  0.117021  0.117008  0.117055  0.004025  0.004056  0.002759  0.
001771  0.003977  0.006620  0.002820  0.000000  0.002836  0.004291  0.002606  0.071865  0.064169  0.073282  0.
085035  0.082724  0.083154  0.086218  0.088866  0.137332  0.164580  0.124866  0.147900  0.080780  0.082058  0.
081725  0.083486  0.084277  0.081718
L25595.1_H     0.135387  0.139487  0.137542  0.135352  0.132110  0.140647  0.146200  0.131707  0.131123  0.141080
 0.141627  0.141680  0.141627  0.141680  0.140470  0.139959  0.140364  0.140089  0.140060  0.119129  0.119147  0.
119178  0.119169  0.119270  0.119139  0.119132  0.119172  0.119159  0.119206  0.002853  0.004627  0.004910  0.
003837  0.005581  0.005662  0.002552  0.002836  0.000000  0.002395  0.004210  0.070260  0.062564  0.071677  0.
083430  0.081119  0.081549  0.084613  0.087261  0.139483  0.166731  0.127017  0.150051  0.082931  0.084209  0.
083876  0.085637  0.084510  0.083869
L08816.1_H     0.137783  0.141883  0.139938  0.137748  0.134506  0.143043  0.148596  0.134103  0.133519  0.143476
 0.144023  0.144076  0.144023  0.144076  0.142866  0.142355  0.142760  0.142485  0.142456  0.121269  0.121287  0.
121318  0.121309  0.121410  0.121279  0.121272  0.121312  0.121299  0.121346  0.000970  0.002232  0.007050  0.
003392  0.003661  0.003267  0.001978  0.004291  0.002395  0.000000  0.002462  0.072656  0.064960  0.074073  0.
085826  0.083515  0.083945  0.087009  0.089657  0.141623  0.168871  0.129157  0.152191  0.085071  0.086349  0.
086016  0.087777  0.086650  0.086009
D11093.1_H     0.139598  0.143698  0.141753  0.139563  0.136321  0.144858  0.150411  0.135918  0.135334  0.145291
 0.145838  0.145891  0.145838  0.145891  0.144681  0.144170  0.144575  0.144300  0.144271  0.118807  0.118825  0.
118856  0.118847  0.118948  0.118817  0.118810  0.118850  0.118837  0.118884  0.002667  0.002227  0.004588  0.
001530  0.001371  0.004791  0.002771  0.002606  0.004210  0.002462  0.000000  0.074471  0.066775  0.075888  0.
087641  0.085330  0.085760  0.088824  0.091472  0.139161  0.166409  0.126695  0.149729  0.082609  0.083887  0.
083554  0.085315  0.086883  0.083547
NM_002343.     0.159249  0.163551  0.161730  0.158585  0.153627  0.159300  0.165512  0.158298  0.158692  0.098747
 0.098531  0.098584  0.098478  0.098584  0.092220  0.091422  0.091744  0.091218  0.091066  0.091967  0.092290  0.
092064  0.091820  0.091921  0.092185  0.092179  0.092185  0.092191  0.092218  0.073114  0.074888  0.072663  0.
073636  0.075842  0.075923  0.072813  0.071865  0.070260  0.072656  0.074471  0.000000  0.007696  0.009024  0.
015052  0.024052  0.023171  0.025917  0.025748  0.109463  0.119005  0.105571  0.098866  0.029666  0.030411  0.
030168  0.032169  0.025064  0.029443
M92089.1_S     0.153439  0.157741  0.155920  0.152775  0.147817  0.153490  0.159702  0.152488  0.152882  0.092937
 0.092721  0.092774  0.092668  0.092774  0.086410  0.085612  0.085934  0.085408  0.085256  0.086068  0.086391  0.
086165  0.085921  0.086022  0.086286  0.086280  0.086286  0.086292  0.086319  0.065418  0.067192  0.064967  0.
065940  0.068146  0.068227  0.065117  0.064169  0.062564  0.064960  0.066775  0.007696  0.000000  0.010816  0.
021328  0.018555  0.018985  0.022049  0.024697  0.103564  0.113106  0.099672  0.092967  0.035476  0.036221  0.
035978  0.037979  0.030874  0.035253
X57084.1_B     0.153723  0.158025  0.156204  0.153059  0.148101  0.153774  0.159986  0.152772  0.153166  0.093221
```

```
 0.093005  0.093058  0.092952  0.093058  0.086694  0.085896  0.086218  0.085692  0.085540  0.096884  0.097207  0.
096981  0.096737  0.096838  0.097102  0.097096  0.097102  0.097108  0.097135  0.074531  0.076305  0.074080  0.
075053  0.077259  0.077340  0.074230  0.073282  0.071677  0.074073  0.075888  0.009024  0.010816  0.000000  0.
022744  0.018526  0.017645  0.020391  0.020222  0.114380  0.123922  0.110488  0.103783  0.035192  0.035937  0.
035694  0.037695  0.030590  0.034969
S95936.1_t     0.144747  0.149049  0.147228  0.144083  0.139125  0.144798  0.151010  0.143796  0.144190  0.084245
 0.084029  0.084082  0.083976  0.084082  0.077718  0.076920  0.077242  0.076716  0.076564  0.076915  0.077238  0.
077012  0.076768  0.076869  0.077133  0.077127  0.077133  0.077139  0.077166  0.086284  0.088058  0.085833  0.
086806  0.089012  0.089093  0.085983  0.085035  0.083430  0.085826  0.087641  0.015052  0.021328  0.022744  0.
000000  0.014888  0.014892  0.015092  0.017347  0.094411  0.103953  0.090519  0.083814  0.044168  0.044913  0.
044670  0.046671  0.039566  0.043945
D89085.1_O     0.135197  0.139499  0.137678  0.134533  0.129575  0.135248  0.141460  0.134246  0.134640  0.074695
 0.074479  0.074532  0.074426  0.074532  0.068168  0.067370  0.067692  0.067166  0.067014  0.079942  0.080265  0.
080039  0.079795  0.079896  0.080160  0.080154  0.080160  0.080166  0.080193  0.083973  0.085747  0.083522  0.
084495  0.086701  0.086782  0.083672  0.082724  0.081119  0.083515  0.085330  0.024052  0.018555  0.018526  0.
014888  0.000000  0.001315  0.003494  0.006905  0.097438  0.106980  0.093546  0.086841  0.053718  0.054463  0.
054220  0.056221  0.049116  0.053495
D89090.1_S     0.136078  0.140380  0.138559  0.135414  0.130456  0.136129  0.142341  0.135127  0.135521  0.075576
 0.075360  0.075413  0.075307  0.075413  0.069049  0.068251  0.068573  0.068047  0.067895  0.081257  0.081580  0.
081354  0.081110  0.081211  0.081475  0.081469  0.081475  0.081481  0.081508  0.084403  0.086177  0.083952  0.
084925  0.087131  0.087212  0.084102  0.083154  0.081549  0.083945  0.085760  0.023171  0.018985  0.017645  0.
014892  0.001315  0.000000  0.003064  0.005712  0.098753  0.108295  0.094861  0.088156  0.052837  0.053582  0.
053339  0.055340  0.048235  0.052614
D89089.1_S     0.133332  0.137634  0.135813  0.132668  0.127710  0.133383  0.139595  0.132381  0.132775  0.072830
 0.072614  0.072667  0.072561  0.072667  0.066303  0.065505  0.065827  0.065301  0.065149  0.081776  0.082099  0.
081873  0.081629  0.081730  0.081994  0.081988  0.081994  0.082000  0.082027  0.087467  0.089241  0.087016  0.
087989  0.090195  0.090276  0.087166  0.086218  0.084613  0.087009  0.088824  0.025917  0.022049  0.020391  0.
015092  0.003494  0.003064  0.000000  0.005071  0.099272  0.108814  0.095380  0.088675  0.055583  0.056328  0.
056085  0.058086  0.050981  0.055360
D89091.1_S     0.133501  0.137803  0.135982  0.132837  0.127879  0.133552  0.139764  0.132550  0.132944  0.072999
 0.072783  0.072836  0.072730  0.072836  0.066472  0.065674  0.065996  0.065470  0.065318  0.086847  0.087170  0.
086944  0.086700  0.086801  0.087065  0.087059  0.087065  0.087071  0.087098  0.090115  0.091889  0.089664  0.
090637  0.092843  0.092924  0.089814  0.088866  0.087261  0.089657  0.091472  0.025748  0.024697  0.020222  0.
017347  0.006905  0.005712  0.005071  0.000000  0.104343  0.113885  0.100451  0.093746  0.055414  0.056159  0.
055916  0.057917  0.050812  0.055191
MF185111.1     0.132922  0.127478  0.128551  0.127423  0.138102  0.126022  0.122881  0.144956  0.148007  0.062766
 0.063313  0.063366  0.063313  0.063366  0.062156  0.061645  0.062050  0.061775  0.061746  0.035506  0.035425  0.
035695  0.035583  0.035583  0.035451  0.035308  0.035417  0.035402  0.035417  0.141357  0.141388  0.134573  0.
138884  0.137962  0.143952  0.140152  0.137332  0.139483  0.141623  0.139161  0.109463  0.103564  0.114380  0.
094411  0.097438  0.098753  0.099272  0.104343  0.000000  0.027248  0.012465  0.016545  0.129671  0.131608  0.
130597  0.130460  0.131331  0.129122
MF185115.1     0.160170  0.154726  0.155799  0.154671  0.165350  0.153270  0.150129  0.172204  0.175255  0.076018
 0.074395  0.074237  0.074342  0.074395  0.055702  0.055503  0.055225  0.055156  0.055746  0.047602  0.047584  0.
047553  0.047562  0.047461  0.047592  0.047599  0.047559  0.047572  0.047525  0.168605  0.168636  0.161821  0.
166132  0.165210  0.171200  0.167400  0.164580  0.166731  0.168871  0.166409  0.119005  0.113106  0.123922  0.
103953  0.106980  0.108295  0.108814  0.113885  0.027248  0.000000  0.039713  0.020139  0.139213  0.141150  0.
140139  0.140002  0.140873  0.138664
MF185117.1     0.120456  0.115012  0.116085  0.114957  0.125636  0.113556  0.116647  0.132490  0.135541  0.069410
 0.069957  0.070010  0.069957  0.070010  0.068800  0.068289  0.068694  0.068419  0.068390  0.039291  0.038907  0.
039434  0.039556  0.039556  0.039029  0.038884  0.039029  0.038994  0.039029  0.128891  0.128922  0.122107  0.
126418  0.125496  0.131486  0.127686  0.124866  0.127017  0.129157  0.126695  0.105571  0.099672  0.110488  0.
090519  0.093546  0.094861  0.095380  0.100451  0.012465  0.039713  0.000000  0.023033  0.125779  0.127716  0.
126705  0.126568  0.127439  0.125230
MF185119.1     0.143490  0.138046  0.139119  0.137991  0.148670  0.136590  0.135121  0.155524  0.158575  0.068356
 0.068140  0.068193  0.068087  0.068193  0.061829  0.061031  0.061353  0.060827  0.060675  0.052051  0.051970  0.
052240  0.052128  0.052128  0.051996  0.051853  0.051962  0.051947  0.051962  0.151925  0.151956  0.145141  0.
149452  0.148530  0.154520  0.150720  0.147900  0.150051  0.152191  0.149729  0.098866  0.092967  0.103783  0.
083814  0.086841  0.088156  0.088675  0.093746  0.016545  0.020139  0.023033  0.000000  0.119074  0.121011  0.
120000  0.119863  0.120734  0.118525
KY486224.1     0.188915  0.193217  0.191396  0.188251  0.183293  0.188966  0.195178  0.187964  0.188358  0.128413
 0.128197  0.128250  0.128144  0.128250  0.121886  0.121088  0.121410  0.120884  0.120732  0.112175  0.112498  0.
112297  0.112185  0.112185  0.112393  0.112387  0.112393  0.112399  0.112426  0.084805  0.084836  0.078021  0.
```

```
082332  0.081410  0.087400  0.083600  0.080780  0.082931  0.085071  0.082609  0.029666  0.035476  0.035192  0.
044168  0.053718  0.052837  0.055583  0.055414  0.129671  0.139213  0.125779  0.119074  0.000000  0.002471  0.
001370  0.002706  0.007842  0.000938
KY486226.1     0.189660  0.193962  0.192141  0.188996  0.184038  0.189711  0.195923  0.188709  0.189103  0.129158
 0.128942  0.128995  0.128889  0.128995  0.122631  0.121833  0.122155  0.121629  0.121477  0.114112  0.114435  0.
114209  0.113965  0.114066  0.114330  0.114324  0.114330  0.114336  0.114363  0.086083  0.086114  0.079704  0.
083610  0.082883  0.088678  0.084878  0.082058  0.084209  0.086349  0.083887  0.030411  0.036221  0.035937  0.
044913  0.054463  0.053582  0.056328  0.056159  0.131608  0.141150  0.127716  0.121011  0.002471  0.000000  0.
001100  0.001758  0.005371  0.002486
KY486227.1     0.189417  0.193719  0.191898  0.188753  0.183795  0.189468  0.195680  0.188466  0.188860  0.128915
 0.128699  0.128752  0.128646  0.128752  0.122388  0.121590  0.121912  0.121386  0.121234  0.113101  0.113424  0.
113198  0.112954  0.113055  0.113319  0.113313  0.113319  0.113325  0.113352  0.085750  0.085781  0.078966  0.
083277  0.082355  0.088345  0.084545  0.081725  0.083876  0.086016  0.083554  0.030168  0.035978  0.035694  0.
044670  0.054220  0.053339  0.056085  0.055916  0.130597  0.140139  0.126705  0.120000  0.001370  0.001100  0.
000000  0.002001  0.006471  0.001475
KY486214.1     0.191418  0.195720  0.193899  0.190754  0.185796  0.191469  0.197681  0.190467  0.190861  0.130916
 0.130700  0.130753  0.130647  0.130753  0.124389  0.123591  0.123913  0.123387  0.123235  0.114611  0.114530  0.
114800  0.114688  0.114688  0.114556  0.114413  0.114522  0.114507  0.114522  0.087511  0.087542  0.080727  0.
085038  0.084116  0.090106  0.086306  0.083486  0.085637  0.087777  0.085315  0.032169  0.037979  0.037695  0.
046671  0.056221  0.055340  0.058086  0.057917  0.130460  0.140002  0.126568  0.119863  0.002706  0.001758  0.
002001  0.000000  0.007105  0.002726
KY486215.1     0.184313  0.188615  0.186794  0.183649  0.178691  0.184364  0.190576  0.183362  0.183756  0.123811
 0.123595  0.123648  0.123542  0.123648  0.117284  0.116486  0.116808  0.116282  0.116130  0.113835  0.114158  0.
113932  0.113688  0.113789  0.114053  0.114047  0.114053  0.114059  0.114086  0.086384  0.087300  0.085075  0.
086048  0.088254  0.088979  0.085225  0.084277  0.084510  0.086650  0.086883  0.025064  0.030874  0.030590  0.
039566  0.049116  0.048235  0.050981  0.050812  0.131331  0.140873  0.127439  0.120734  0.007842  0.005371  0.
006471  0.007105  0.000000  0.007228
KY486216.1     0.188692  0.192994  0.191173  0.188028  0.183070  0.188743  0.194955  0.187741  0.188135  0.128190
 0.127974  0.128027  0.127921  0.128027  0.121663  0.120865  0.121187  0.120661  0.120509  0.111885  0.111949  0.
112074  0.111962  0.111962  0.111844  0.111838  0.111844  0.111850  0.111877  0.085743  0.085774  0.078959  0.
083270  0.082348  0.088338  0.084538  0.081718  0.083869  0.086009  0.083547  0.029443  0.035253  0.034969  0.
043945  0.053495  0.052614  0.055360  0.055191  0.129122  0.138664  0.125230  0.118525  0.000938  0.002486  0.
001475  0.002726  0.007228  0.000000
```

29 Escherichia/Shigella complete genomes

```
    29
CU928160.2     0.000000  0.000349  0.001084  0.002022  0.003423  0.004214  0.000763  0.000735  0.000732  0.001564
 0.000291  0.000415  0.000948  0.000526  0.000456  0.003017  0.002095  0.002243  0.002541  0.000838  0.001394  0.
001776  0.002060  0.001519  0.000868  0.002069  0.001145  0.001417  0.001736
AP009240.1     0.000349  0.000000  0.001433  0.002141  0.003542  0.004333  0.001020  0.001055  0.001052  0.001683
 0.000538  0.000563  0.001067  0.000674  0.000604  0.002736  0.002243  0.002391  0.002689  0.000986  0.001275  0.
001924  0.002208  0.001602  0.000749  0.002217  0.001293  0.001272  0.001884
CP000800.1     0.001084  0.001433  0.000000  0.002730  0.004131  0.004922  0.001625  0.001472  0.001744  0.002272
 0.001126  0.001207  0.001656  0.001218  0.001134  0.004101  0.001082  0.001230  0.001528  0.000772  0.000774  0.
000880  0.001047  0.000872  0.001282  0.001056  0.000843  0.000780  0.000953
CP000038.1     0.002022  0.002141  0.002730  0.000000  0.001401  0.002388  0.001258  0.001352  0.001795  0.001203
 0.001731  0.001636  0.001405  0.001619  0.001596  0.002746  0.002957  0.003252  0.002679  0.001957  0.003416  0.
003216  0.002310  0.003541  0.002890  0.003116  0.002004  0.002793  0.002739
CP000036.1     0.003423  0.003542  0.004131  0.001401  0.000000  0.001251  0.002659  0.002753  0.003196  0.002097
 0.003132  0.003037  0.002475  0.003020  0.002997  0.002115  0.004094  0.004653  0.003623  0.003358  0.004817  0.
004617  0.003639  0.004942  0.004291  0.004517  0.003405  0.004194  0.004140
CP001063.1     0.004214  0.004333  0.004922  0.002388  0.001251  0.000000  0.003450  0.003544  0.003987  0.002650
 0.003923  0.003828  0.003266  0.003811  0.003788  0.002538  0.005345  0.005444  0.004874  0.004149  0.005608  0.
005408  0.004698  0.005733  0.005082  0.005308  0.004196  0.004985  0.004931
CP000266.1     0.000763  0.001020  0.001625  0.001258  0.002659  0.003450  0.000000  0.000153  0.000536  0.001313
 0.000508  0.000892  0.001366  0.001003  0.000933  0.002476  0.002572  0.002720  0.003018  0.001315  0.002157  0.
002253  0.002537  0.002282  0.001631  0.002546  0.001622  0.002117  0.002290
AE014073.1     0.000735  0.001055  0.001472  0.001352  0.002753  0.003544  0.000153  0.000000  0.000443  0.001288
 0.000517  0.000888  0.001341  0.000993  0.000908  0.002629  0.002547  0.002695  0.002993  0.001290  0.002064  0.
002252  0.002512  0.002244  0.001538  0.002521  0.001622  0.002152  0.002325
```

```
AE005674.2     0.000732  0.001052  0.001744  0.001795  0.003196  0.003987  0.000536  0.000443  0.000000  0.001568
 0.000763  0.001147  0.001621  0.001258  0.001188  0.002791  0.002827  0.002975  0.003273  0.001570  0.001621  0.
002508  0.002792  0.002241  0.001095  0.002801  0.001877  0.002149  0.002468
CP000946.1     0.001564  0.001683  0.002272  0.001203  0.002097  0.002650  0.001313  0.001288  0.001568  0.000000
 0.001273  0.001178  0.000616  0.001161  0.001138  0.003720  0.002728  0.002794  0.002257  0.001499  0.002958  0.
002758  0.002081  0.003083  0.002432  0.002686  0.001546  0.002335  0.002281
CP000802.1     0.000291  0.000538  0.001126  0.001731  0.003132  0.003923  0.000508  0.000517  0.000763  0.001273
 0.000000  0.000384  0.000858  0.000495  0.000425  0.002975  0.002064  0.002212  0.002510  0.000807  0.001685  0.
001745  0.002029  0.001810  0.001159  0.002038  0.001114  0.001635  0.001808
U00096.3_E     0.000415  0.000563  0.001207  0.001636  0.003037  0.003828  0.000892  0.000888  0.001147  0.001178
 0.000384  0.000000  0.000562  0.000110  0.000230  0.003299  0.001892  0.001936  0.002125  0.000621  0.001780  0.
001580  0.001644  0.001905  0.001254  0.001850  0.000734  0.001264  0.001437
AP009048.1     0.000948  0.001067  0.001656  0.001405  0.002475  0.003266  0.001366  0.001341  0.001621  0.000616
 0.000858  0.000562  0.000000  0.000545  0.000599  0.003773  0.002261  0.002305  0.001790  0.000990  0.002342  0.
002142  0.001614  0.002467  0.001816  0.002219  0.000930  0.001719  0.001665
CP000948.1     0.000526  0.000674  0.001218  0.001619  0.003020  0.003811  0.001003  0.000993  0.001258  0.001161
 0.000495  0.000110  0.000545  0.000000  0.000335  0.003410  0.001903  0.001947  0.002015  0.000632  0.001797  0.
001597  0.001534  0.001922  0.001271  0.001861  0.000629  0.001174  0.001332
CP001396.1     0.000456  0.000604  0.001134  0.001596  0.002997  0.003788  0.000933  0.000908  0.001188  0.001138
 0.000425  0.000230  0.000599  0.000335  0.000000  0.003340  0.001662  0.001787  0.002085  0.000391  0.001820  0.
001620  0.001604  0.001945  0.001294  0.001620  0.000964  0.001494  0.001667
CP000034.1     0.003017  0.002736  0.004101  0.002746  0.002115  0.002538  0.002476  0.002629  0.002791  0.003720
 0.002975  0.003299  0.003773  0.003410  0.003340  0.000000  0.004979  0.005127  0.005425  0.003722  0.003777  0.
004660  0.004944  0.004270  0.003256  0.004953  0.004029  0.004008  0.004620
CP001846.1     0.002095  0.002243  0.001082  0.002957  0.004094  0.005345  0.002572  0.002547  0.002827  0.002728
 0.002064  0.001892  0.002261  0.001903  0.001662  0.004979  0.000000  0.000723  0.000585  0.001271  0.001857  0.
000583  0.000647  0.000908  0.002365  0.000500  0.001528  0.001291  0.000907
AE005174.2     0.002243  0.002391  0.001230  0.003252  0.004653  0.005444  0.002720  0.002695  0.002975  0.002794
 0.002212  0.001936  0.002305  0.001947  0.001787  0.005127  0.000723  0.000000  0.001204  0.001405  0.002005  0.
000467  0.001014  0.001000  0.002513  0.000223  0.001572  0.001335  0.000951
BA000007.2     0.002541  0.002689  0.001528  0.002679  0.003623  0.004874  0.003018  0.002993  0.003273  0.002257
 0.002510  0.002125  0.001790  0.002015  0.002085  0.005425  0.000585  0.001204  0.000000  0.001703  0.002303  0.
001168  0.000481  0.001493  0.002811  0.001068  0.001396  0.001417  0.000805
CU928163.2     0.000838  0.000986  0.000772  0.001957  0.003358  0.004149  0.001315  0.001290  0.001570  0.001499
 0.000807  0.000621  0.000990  0.000632  0.000391  0.003722  0.001271  0.001405  0.001703  0.000000  0.001458  0.
001258  0.001222  0.001583  0.001108  0.001231  0.000611  0.001141  0.001314
CU928164.2     0.001394  0.001275  0.000774  0.003416  0.004817  0.005608  0.002157  0.002064  0.001621  0.002958
 0.001685  0.001780  0.002342  0.001797  0.001820  0.003777  0.001857  0.002005  0.002303  0.001458  0.000000  0.
001538  0.001822  0.001005  0.000526  0.001831  0.001412  0.000886  0.001498
CP000970.1     0.001776  0.001924  0.000880  0.003216  0.004617  0.005408  0.002253  0.002252  0.002508  0.002758
 0.001745  0.001580  0.002142  0.001597  0.001620  0.004660  0.000583  0.000467  0.001168  0.001258  0.001538  0.
000000  0.000978  0.000533  0.002046  0.000293  0.001212  0.000975  0.000591
FM180568.1     0.002060  0.002208  0.001047  0.002310  0.003639  0.004698  0.002537  0.002512  0.002792  0.002081
 0.002029  0.001644  0.001614  0.001534  0.001604  0.004944  0.000647  0.001014  0.000481  0.001222  0.001822  0.
000978  0.000000  0.001303  0.002330  0.000878  0.000915  0.000936  0.000501
CP000247.1     0.001519  0.001602  0.000872  0.003541  0.004942  0.005733  0.002282  0.002244  0.002241  0.003083
 0.001810  0.001905  0.002467  0.001922  0.001945  0.004270  0.000908  0.001000  0.001493  0.001583  0.001005  0.
000533  0.001303  0.000000  0.001513  0.000826  0.001537  0.000775  0.000802
CU928162.2     0.000868  0.000749  0.001282  0.002890  0.004291  0.005082  0.001631  0.001538  0.001095  0.002432
 0.001159  0.001254  0.001816  0.001271  0.001294  0.003256  0.002365  0.002513  0.002811  0.001108  0.000526  0.
002046  0.002330  0.001513  0.000000  0.002339  0.001415  0.001394  0.002006
AE014075.1     0.002069  0.002217  0.001056  0.003116  0.004517  0.005308  0.002546  0.002521  0.002801  0.002686
 0.002038  0.001850  0.002219  0.001861  0.001620  0.004953  0.000500  0.000223  0.001068  0.001231  0.001831  0.
000293  0.000878  0.000826  0.002339  0.000000  0.001486  0.001249  0.000865
CU928161.2     0.001145  0.001293  0.000843  0.002004  0.003405  0.004196  0.001622  0.001622  0.001877  0.001546
 0.001114  0.000734  0.000930  0.000629  0.000964  0.004029  0.001528  0.001572  0.001396  0.000611  0.001412  0.
001212  0.000915  0.001537  0.001415  0.001486  0.000000  0.000789  0.000735
CP000243.1     0.001417  0.001272  0.000780  0.002793  0.004194  0.004985  0.002117  0.002152  0.002149  0.002335
 0.001635  0.001264  0.001719  0.001174  0.001494  0.004008  0.001291  0.001335  0.001417  0.001141  0.000886  0.
000975  0.000936  0.000775  0.001394  0.001249  0.000789  0.000000  0.000612
CP000468.1     0.001736  0.001884  0.000953  0.002739  0.004140  0.004931  0.002290  0.002325  0.002468  0.002281
 0.001808  0.001437  0.001665  0.001332  0.001667  0.004620  0.000907  0.000951  0.000805  0.001314  0.001498  0.
```

000591 0.000501 0.000802 0.002006 0.000865 0.000735 0.000612 0.000000

9 mammalian X chromosomes

```
    9
Human_X_ch     0.000000  0.000923  0.001189  0.004122  0.002227  0.001907  0.008808  0.017529  0.001013
Monkey_X_c     0.000923  0.000000  0.000292  0.004501  0.001534  0.001289  0.009187  0.018147  0.000093
Gorilla_X_     0.001189  0.000292  0.000000  0.004651  0.001358  0.000997  0.009337  0.018439  0.000321
Dog_X_chro     0.004122  0.004501  0.004651  0.000000  0.005881  0.005227  0.004686  0.014329  0.004594
Horse_X_ch     0.002227  0.001534  0.001358  0.005881  0.000000  0.000953  0.010567  0.019604  0.001560
Mouse_X_ch     0.001907  0.001289  0.000997  0.005227  0.000953  0.000000  0.009614  0.019436  0.001318
Opossum_X_     0.008808  0.009187  0.009337  0.004686  0.010567  0.009614  0.000000  0.011243  0.009280
Platypus_X     0.017529  0.018147  0.018439  0.014329  0.019604  0.019436  0.011243  0.000000  0.018118
Chimpanzee     0.001013  0.000093  0.000321  0.004594  0.001560  0.001318  0.009280  0.018118  0.000000
```
