# Supplementary material for: Alignment-free method for DNA sequence clustering using Fuzzy integral similarity
Source: Sci Rep. 2019 Mar 6;9:3753. doi: 10.1038/s41598-019-40452-6 (PMC6403383; doi:10.1038/s41598-019-40452-6)
Supplement: Supplementary file 5 — Dataset 5 [file 41598_2019_40452_MOESM5_ESM.zip › run/media/snandi/NEW VOLUME/DNA submission/MODIFIED_sci.Rep_26_07_18/collection_of_mts/readme.docx]

**README FILE**

**Fuzzy integral similarity for Alignment-free sequence comparison through a Markov chain**

We used MEGA7 tools for opening “.mts” .
